# Selective synthesis of *gem*-dihalopiperidines and 4-halo-1,2,3,6-tetrahydropyridines from halogen substituted homoallylic benzenesulfonamides and aldehydes†

**DOI:** 10.1039/d5ra03630e

**Published:** 2025-06-23

**Authors:** Surjya Kumar Bora, Anil K. Saikia

**Affiliations:** a Department of Chemistry, Indian Institute of Technology Guwahati Guwahati-781039 Assam India asaikia@iitg.ac.in

## Abstract

An efficient synthesis of *gem*-dihalopiperidines and 4-halo-1,2,3,6-tetrahydropyridines *via* aza-Prins cyclization reaction of homoallylic benzenesulfonamides and aldehydes has been described. The reaction proceeds *via* aza-Prins followed by base-mediated elimination reaction, giving moderate to good yields. The reaction is highly diastereo- and regio-selective. Furthermore, the *gem*-dihalopiperidines can be easily converted to 2-substituted-1-tosylpiperidin-4-one and pyridine in good yields. Additionally, 4-halo-1,2,3,6-tetrahydropyridines can be employed to afford their corresponding Sonogashira coupling products in good yield.

## Abstract 2

A diastereo- and regioselective synthesis of *gem*-dihalopiperidines and 4-halo-1,2,3,6-tetrahydropyridines has been unveiled. Post synthetic modifications of these compounds provide piperidin-4-ones, pyridines, and Sonogashira coupling products.

## Graphical abstract

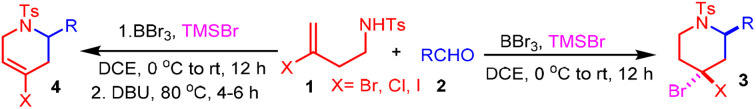

## Introduction

**Fig. 1:**
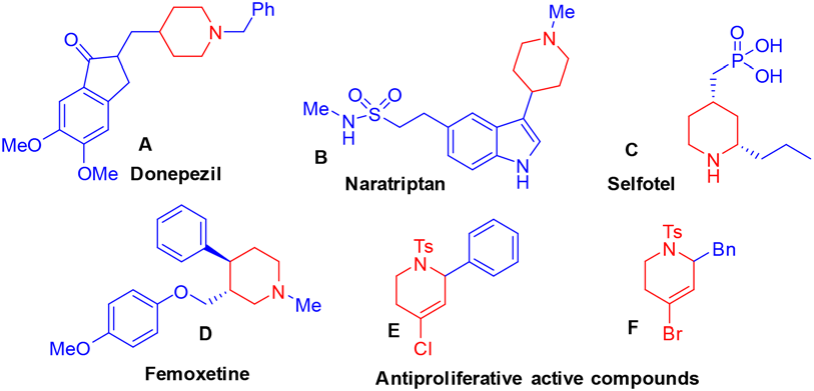
Representative examples of biologically active molecules.

Six-membered nitrogen heterocycles are key structural units in organic synthesis, as they are found in a wide range of natural products and bioactive compounds.^1^ Among these, the piperidine ring is highly prevalent in alkaloids,^2^ and serves as a fundamental scaffold in drug discovery and development,^3^*e.g.*, donepezil (A), a widely prescribed medication, is commonly used for the treatment of Alzheimer's disease (AD).^4^ Naratriptan (B) is utilized for the treatment of migraine headaches,^1*d*^ while selfotel (C) functions as a competitive *N*-methyl-d-aspartate (NMDA) receptor agonist.^5^ Femoxetine (D) belongs to a significant class of serotonin reuptake inhibitors.^6^ Similarly, tetrahydropyridine compounds E and F show antiproliferative activity in solid tumour cell lines (Fig. 1).^7^ Due to their broad spectrum of biological activity in both pharmaceuticals and natural products, the synthesis of these compounds continues to be of significant interest to synthetic chemists. As a result, considerable research efforts have been made towards developing novel and efficient synthetic methodologies for their preparation.

The aza-Prins cyclization is a well-recognized method for synthesizing nitrogen-containing heterocyclic compounds.^8^ Similarly, the halo-aza-Prins cyclization, where a halide ion serves as a nucleophile to produce mono-halogenated compounds, is also well documented.^9^ However, the synthesis of *gem*-dihalopiperidines and 4-halo-1,2,3,6-tetrahydropyridines has been rarely reported in the literature.

**Scheme 1:**
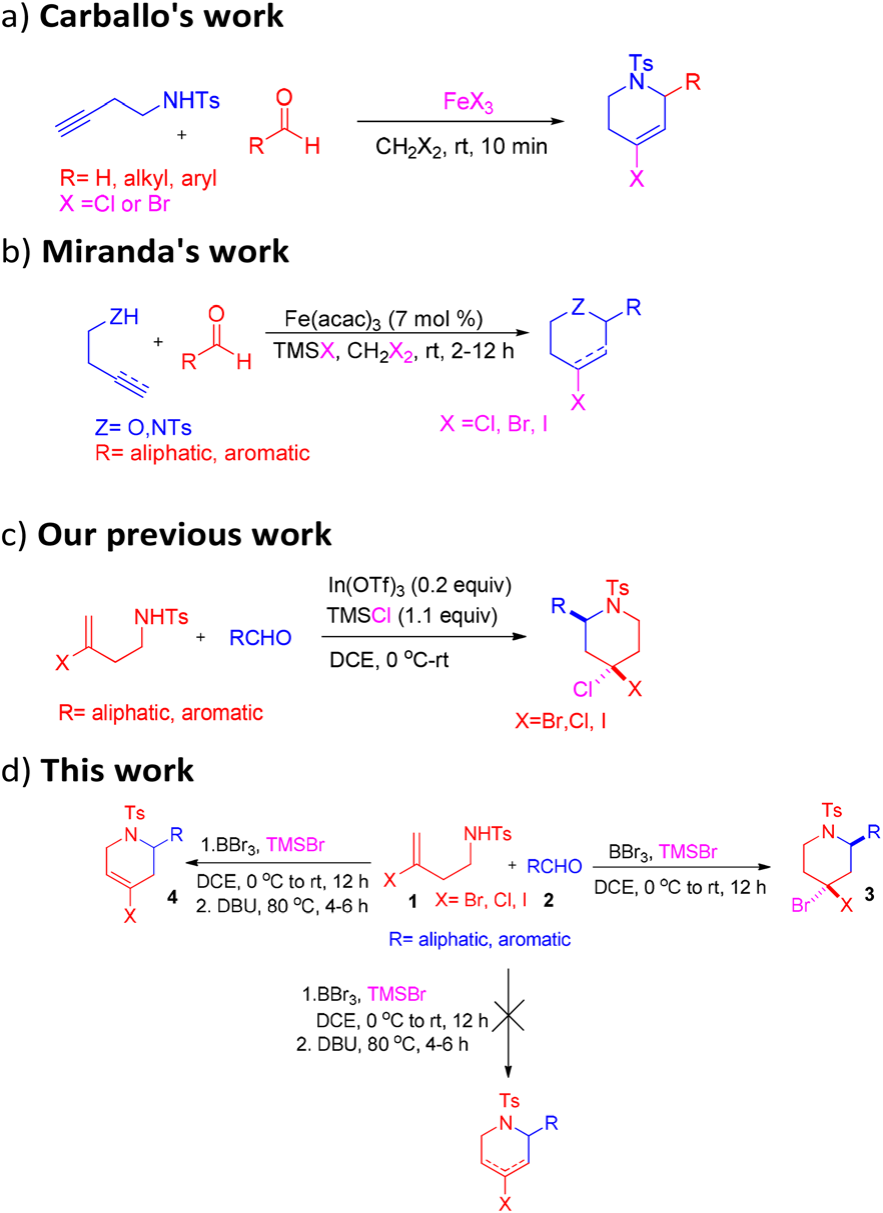
Previous and present work.

In 2006, Carballo *et al.* reported the synthesis of tetrahydro-pyridines using iron(iii) halides as a reagent (Scheme 1a).^10^ In another study, Miranda *et al.* reported a synthetic strategy of oxa- and azacycles through the combination of an iron(iii) source with the corresponding trimethylsilyl halide (Scheme 1b).^11^ Recently, our group introduced a new approach for synthesizing 4,4-dihalopiperidines *via* halo-aza-Prins cyclization reaction, wherein chlorine acts as nucleophile (Scheme 1c).^12^ Despite these advancements, the rapid and efficient synthesis of *gem*-dihalopiperidines and tetrahydropyridine derivatives remains challenging. Therefore, the development of more efficient synthetic strategies is necessary to enhance the practicality of this approach. Herein, we established a new methodology for the synthesis of *gem*-dihalopiperidines and 4-halo-1,2,3,6-tetrahydropyridines *via* halo-aza-Prins cyclization reaction of halogen-substituted homoallylic benzenesulfonamide and aldehyde (Scheme 1d). Notably, the position of the double bond of tetrahydropyridines 4 in the present case differs from that of the products reported by Carballo^10^ and Miranda.^11^ Also, in *gem*-dihalopiperidines, Br (axial) acts as a nucleophile, which differs from our previous report where Cl (axial) acted as a nucleophile. Consequently, the two cases give products with different stereochemistry, particularly in the case of 4-bromo-4-chloro derivatives.

## Results and discussion

Initially, *N*-(3-bromobut-3-en-1-yl)-4-methylbenzenesulfon-amide (1a) was reacted with 4-chloro-benzaldehyde (2c) using 1.1 equiv. of boron trifluoride etherate (BF_3_·OEt_2_) in dichloromethane (DCM) at 0 °C to room temperature under a nitrogen atmosphere for 1.5 hours (Table 1, entry 1). However, this led to the decomposition of the starting material. When the reagent was changed to boron tribromide (BBr_3_), it gave 18% of product 3ac with unreacted starting material (Table 1, entry 2).

**Table 1:** Optimization of the reaction^a^

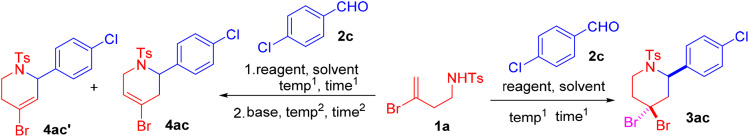							
S. no	Reagents (equiv.)	Base (eq.) (temp^2^) (time^2^)	Solvent	Temp^1^	Time^1^/h	3ac	4ac : 4ac′
						% yield^b^	% yield (4ac : 4ac′)
1	BF_3_·OEt_2_ (1.1)	—	DCM	0 °C-rt	1.5	—c	—
2	BBr_3_ (1.1)	—	DCM	0 °C	12	18	—
3	InBr_3_ (1.1)	—	DCM	0 °C-rt	12	NR	
4	BBr_3_ (1.1)	—	DCM	40 °C	2	—c	—
5	BBr_3_ (0.25) + TMSBr (1.2)	—	DCM	0 °C-rt	12	80	—
6	In(OTf)_3_ (0.25) + TMSBr (1.2)	—	DCM	0 °C-rt	12	NR	—
7	Sc(OTf)_3_ (0.25) + TMSBr (1.2)	—	DCM	0 °C-rt	12	NR	—
8	TMSBr (1.2)	—	DCM	0 °C-rt	12	9	—
9	BBr_3_ (0.1) + TMSBr (1.2)	—	DCM	0 °C-rt	12	76	—
10	BBr_3_ (0.3) + TMSBr (1.2)	—	DCM	0 °C-rt	12	78	—
**11**	**BBr**_**3**_ **(0.25) + TMSBr (1.2)**	**—**	**DCE**	**0 °C-rt**	**12**	**83**	—
12	BBr_3_(0.25) + TMSBr (1.2)	—	Toluene	0 °C-rt	12	71	—
13	BBr_3_(0.25) + TMSBr (1.2)	—	CH_3_CN	0 °C-rt	12	NR	—
14	BBr_3_ (0.25) + TMSBr (1.2)	DBU (1.0)	DCE	0 °C-rt	12	51	20 (1 : 2)
		(rt) (12 h)					
15	BBr_3_ (0.25) + TMSBr (1.2)	KO^*t*^Bu (1.0)	DCE	0 °C-rt	12	74	NR
		(rt) (12 h)					
16	BBr_3_ (0.25) + TMSBr (1.2)	DABCO (1.0)	DCE	0 °C-rt	12	—	—d
		(rt) (4 h)					
17	BBr_3_ (0.25) + TMSBr (1.2)	Piperidine (1.0)	DCE	0 °C-rt	12	—	—d
		(rt) (4 h)					
18	BBr_3_ (0.25) + TMSBr (1.2)	DBU (10.0)	DCE	0 °C-rt	12	—	68 (1 : 2)
		(80) (4 h)					
19	BBr_3_ (0.25) + TMSBr (1.2)	DBU (20.0)	DCE	0 °C-rt	12	—	69 (1 : 1)
		(80) (4 h)					
20	BBr_3_ (0.25) + TMSBr (1.2)	DBU (40.0)	DCE	0 °C-rt	12	—	70 (1 : 0)
		(80) (4 h)					

a Reaction conditions: all reactions were carried out under a nitrogen atmosphere, 1a (0.6 mmol) and 2c (0.66 mmol), solvent (3.0 mL).

b Isolated yield.

c Decomposed. NR = no reaction.

d Complex mixture. Regioselectivity was determined by ^1^H NMR spectroscopy.

Changing BBr_3_ to indium tribromide (InBr_3_) did not yield any product (Table 1, entry 3). Therefore, in order to increase the yield, the reaction was performed at 40 °C with BBr_3_, which led to the decomposition of the product (Table 1, entry 4). Fortunately, when the reaction was performed using 0.25 equiv. of BBr_3_ with 1.2 equiv. of trimethylsilyl bromide (TMSBr) as an additive, the yield of 3ac increased to 80% (Table 1, entry 5). Other combinations, such as indium triflate (In(OTf)_3_) and scandium triflate (Sc(OTf)_3_) with TMSBr, failed to produce 3ac (Table 1, entries 6 and 7). When the reaction was performed using only 1.2 equiv. of TMSBr resulted in a mere 9% yield of the product with unreacted starting material (Table 1, entry 8). Additionally, decreasing the BBr_3_ loading to 0.1 equiv. or increasing it to 0.3 equiv. did not improve the yield (Table 1, entries 9 and 10). However, changing the solvent to 1,2-dichloroethane (DCE) resulted in an improved yield of 83% (Table 1, entry 11). It may be due to its higher polarity than DCM which dissolves both organic substrates and Lewis acids effectively to promote the transformation. Other solvents, such as toluene and acetonitrile, did not improve the yield of product 3ac (Table 1, entries 12 and 13). Thus, 0.25 equiv. of BBr_3_ and 1.2 equiv. of TMSBr in DCE at 0 °C to rt were the optimal conditions for the product 3ac.

After confirming the formation of product 3ac by thin layer chromatography (TLC), 1.0 equiv. of 1,8-diazabicyclo-[5.4.0]undec-7-ene (DBU) was added to the reaction mixture at rt and stirred for 12 hours. Interestingly, this resulted in 20% yield of regioisomeric products 4ac and 4ac′ with a regioselectivity 1 : 2, along with unreacted 3ac (Table 1, entry 14). In order to improve the yield and regioselectivity, various reaction conditions were examined (Table 1). However, the use of inorganic base potassium tertiary butoxide (KO^*t*^Bu) failed to produce 4ac, possibly due to solubility issues (Table 1, entry 15). Other organic bases such as 1,4-diazabicyclo[2.2.2]octane (DABCO) and piperidine led to the formation of a complex mixture (Table 1, entries 16–17). Increasing the temperature to 80 °C and using 10.0 equiv. of DBU improved the yield to 68%, but the regioselectivity remains same (Table 1, entry 18). Notably, increasing the loading of DBU to 20.0 equiv. provided 4ac and 4ac′ with a ratio of 1 : 1 (Table 1, entry 19). Finally, employing 40.0 equiv. of DBU resulted in the formation of a single regioselective product 4ac (Table 1, entry 20). Therefore, it was concluded that 0.25 equiv. of BBr_3_ and 1.2 equiv. of TMSBr in DCE at 0 °C to rt for 12 h, followed by treatment with 40.0 equiv. of DBU at 80 °C were the optimal conditions for the product 4ac. The high concentration of DBU may be required to abstract the proton from the less hindered site of the *gem*-dihalopiperidine effectively as DBU is bulky molecule.

**Scheme 2:**
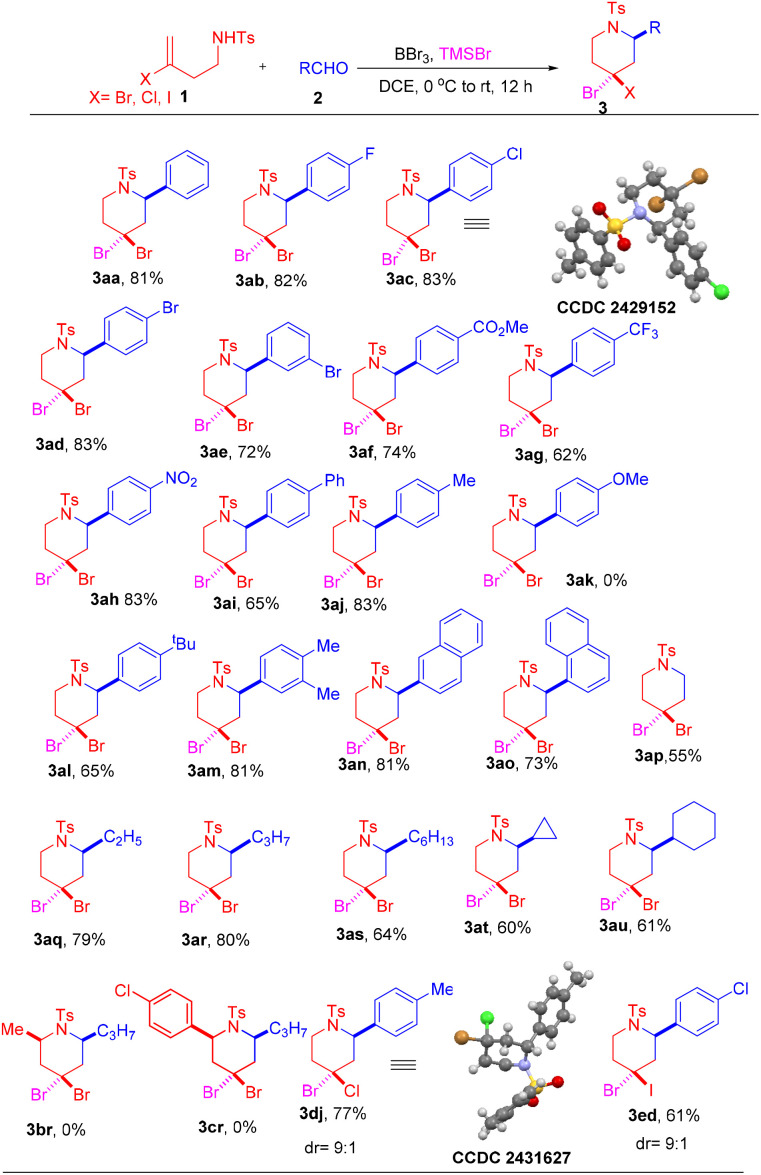
Synthesis of *gem*-dihalopiperidines.^a^ The formula of the major isomer is shown in the scheme. ^a^ Reaction conditions: 1 (0.6 mmol), 2 (0.66 mmol), BBr_3_ (0.15 mmol), TMSBr (0.72 mmol), DCE (3 mL), 0 °C-rt, N_2_ atmosphere. Diastereoselectivity was determined by ^1^H NMR spectroscopy.

**Scheme 3:**
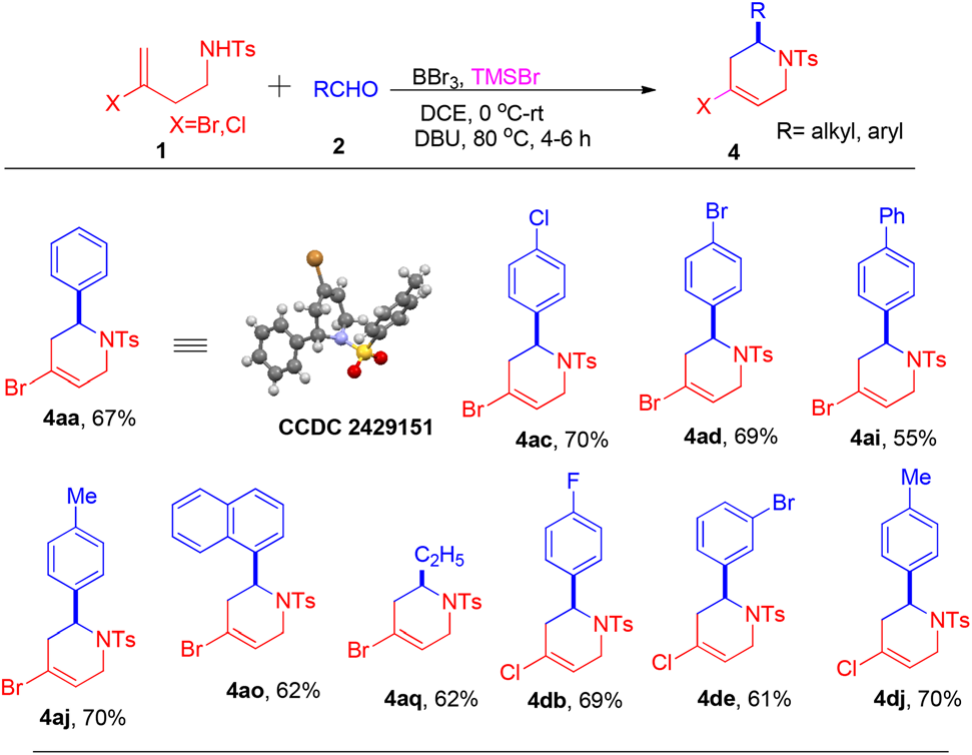
Synthesis of 4-halo-1,2,3,6-tetrahydropyridines.^a^ Reaction conditions: 1 (0.6 mmol), 2 (0.66 mmol), BBr_3_ (0.15 mmol), TMSBr (0.72 mmol), DCE (3 mL), 0 °C-rt, DBU (24.0 mmol), N_2_ atmosphere.

Under the first established optimal conditions, the reaction was screened with different aldehydes, as presented in Scheme 2. Aldehydes bearing moderately electron withdrawing groups at *para* and *meta* positions of the aromatic ring such as –F, –Cl and –Br (Scheme 2, 3ab–3ae), as well as those with strongly electron-withdrawing groups, including such as –CO_2_Me, –CF_3_ and –NO_2_ (Scheme 2, 3af–3ah), gave good to excellent yields. Similarly, when the reaction was carried out with aldehydes containing a moderately electron-donating group such as –Me, 3,4-dimethyl or a bulky electron-donating *tert*-butyl group at the *para* positions gave the products 3aj, 3am and 3al in 83%, 81% and 65% yields, respectively. However, a highly electron-donating methoxy group on the aromatic ring of aldehyde 2k led to decomposition, similar to many Prins cyclisation reactions.^12^ Intriguingly, biphenyl aldehyde 2i gave 3ai in 65% yield, and bulky substrate such as 2-naphthaldehyde 2n and sterically-hindered 1-naphthaldehyde 2o provided corresponding products 3an and 3ao in 81% and 73% yields, respectively. Aliphatic aldehydes, including –C_2_H_5_, –C_3_H_7_, and –C_6_H_13_ groups, also furnished their corresponding products in decent yields (3aq–3au). Unfortunately, secondary amide *N*-(4-bromopent-4-en-2-yl)-4-methylbenzenesulfonamide (1b) and *N*-(3-bromo-1-(4-chlorophenyl)but-3-en-1-yl)-4-methyl-benzenesulfonamide (1c) did not yield the desired products. Under the same optimized reaction conditions, the reaction also proceeded efficiently with halogen-substituted homoallylic benzenesulfonamides bearing chlorine (Cl) or iodine (I), specifically, *N*-(3-chlorobut-3-en-1-yl)-4-methylbenzene-sulfonamide (1d) and *N*-(3-iodobut-3-en-1-yl)-4-methylbenzenesulfonamide (1e), giving products 3dj–3ed in moderate to good yields (Scheme 2). The structure and stereochemistry of all compounds (3aa–3aj, 3al–3au, 3dj, 3ed) were determined by ^1^H NMR, ^13^C{^1^H} NMR, mass spectrometry, and ultimately by X-ray crystallographic analysis of compounds 3ac and 3dj. Likewise, under the second set of optimized reaction conditions, the reaction was explored with different aldehydes as shown in Scheme 3. Substrates with electron-withdrawing groups such as –Cl, –Br on the aromatic ring, provided products 4ac and 4ad in good to moderate yields. Electron-donating group in the aromatic ring of the aldehyde provided a 70% yield of their corresponding product 4aj. Moreover, biphenyl and sterically hindered 1-naphthyl groups were well tolerated under the reaction conditions. The substrate with an aliphatic group gave 62% of their desired product 4aq. The reaction of halogen-substituted homoallylic benzenesulfonamides containing chlorine (Cl) with electron-donating and electron-withdrawing groups at the *para* and *meta* positions of the aromatic group of the aldehyde gave their corresponding products 4db, 4de, and 4dj in moderate yields (Scheme 3). The structure of all compounds (4aa–4dj) was determined by ^1^H NMR, ^13^C{^1^H} NMR, and mass spectrometry. The stereochemistry of the compounds was determined by comparison with the reported ^1^H NMR data.^10,11^ For example, the olefinic proton of 4aa resonates at 5.92 ppm, whereas the olefinic proton of the corresponding regioisomer of Carballo's group^10^ resonates at 6.22 ppm. Finally, the structure and stereochemistry of compounds were determined by X-ray crystallographic analysis of compound 4aa.

**Scheme 4:**
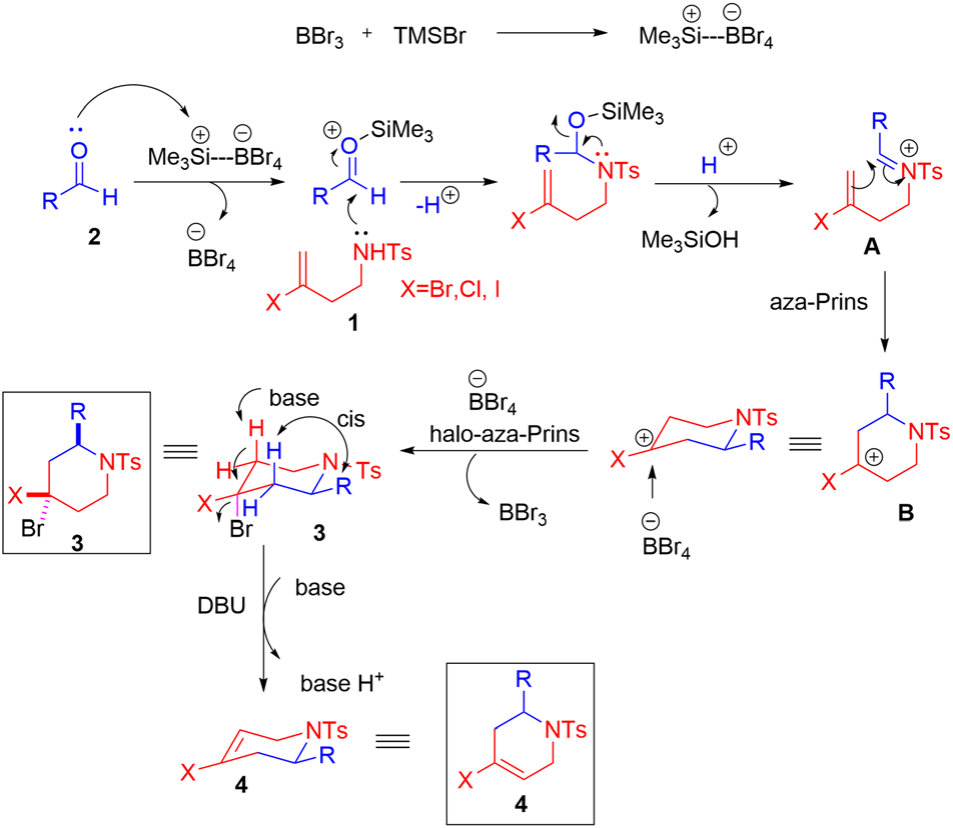
Mechanism of the reaction.

A plausible mechanism is proposed in Scheme 4. First, BBr_3_ reacts with TMSBr to generate Me_3_Si^+^–BBr_4_^−^ species.^13^ In the presence of these species, the halogen-substituted homoallylic benzenesulfonamide 1 reacts with aldehyde 2 to form the iminium ion intermediate (A), which then undergoes aza-Prins cyclisation to produce the carbocation intermediate (B). Further axial attack by bromide ion from BBr_4_^−^ gives product 3. After the addition of organic base DBU, its abstract proton from compound 3 to give the final product 4. The formation of a single regioisomer may be attributed to the selective abstraction of a proton from C-5 of the piperidine ring. The proton at C-3 is not in a position to be abstracted by bulky DBU as it is sterically hindered due to its *cis* configuration with the bulky substituent “R” at the C-2 position.

**Scheme 5:**
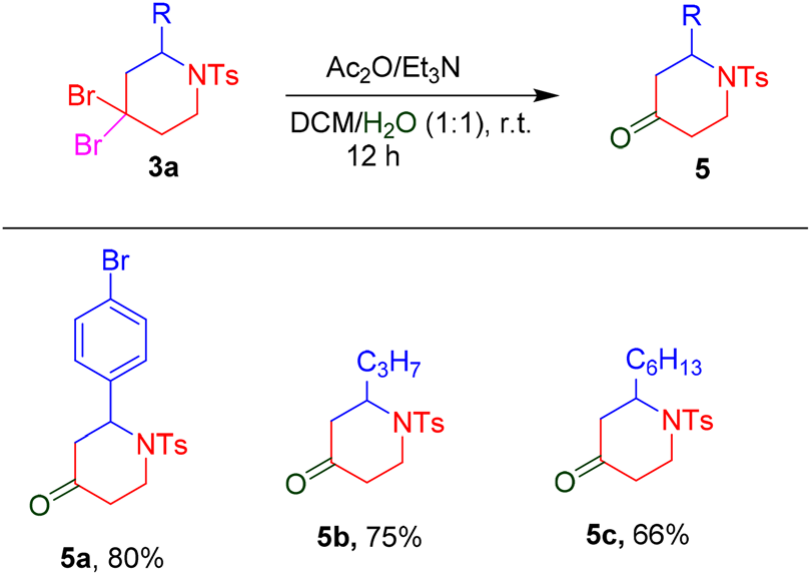
Synthesis of 2-substituted-1-tosylpiperidin-4-one.

**Scheme 6:**
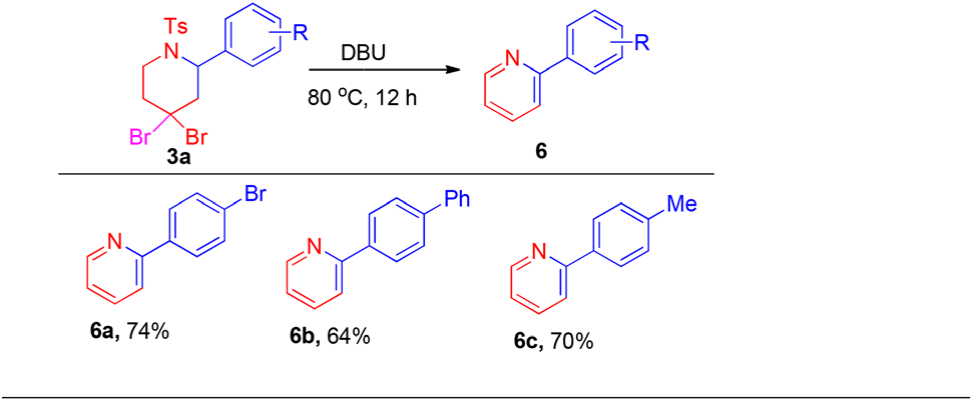
Synthesis of pyridine.

**Scheme 7:**
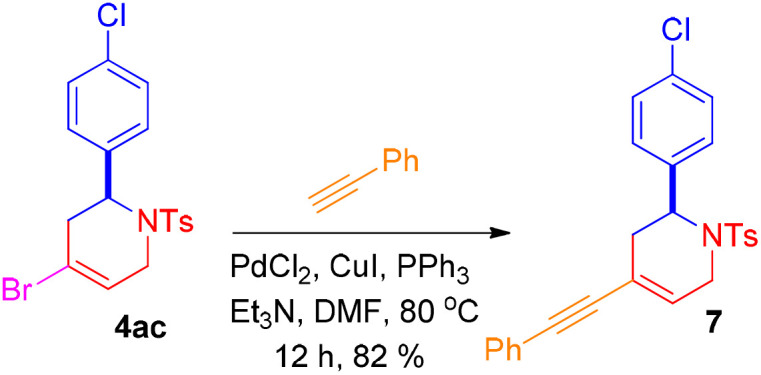
Synthesis of 2-(4-chlorophenyl)-4-(phenylethynyl)-1-tosyl-1,2,3,6-tetrahydropyridine.

To investigate the utility of this methodology, several reactions were carried out as shown in Schemes 5–7. The *gem*-dihalogen compounds 3ad, 3ar and 3as were treated with triethylamine and acetic anhydride in DCM/H_2_O (1 : 1), to give corresponding piperidinone compounds 2-(4-bromophenyl)-1-tosylpiperidin-4-one (5a), 2-propyl-1-tosylpiperidin-4-one (5b) and 2-hexyl-1-tosylpiperidin-4-one (5c) in 80%, 75% and 66% yields, respectively, under a previously reported procedure (Scheme 5).^14^ Furthermore, treatment with DBU of *gem*-dihalocompounds 3ad, 3ai and 3aj gave their corresponding pyridine derivatives 6a–6c in 74%, 64% and 70% yields, respectively (Scheme 6).^12^

The reaction of 4-halo-1,2,3,6-tetrahydropyridines gives Sonogashira product using literature precedents.^15^ Thus, the reaction of 4-bromo-2-(4-chlorophenyl)-1-tosyl-1,2,3,6- tetrahydropyridine (4ac) with phenyl acetylene in the presence of PdCl_2_, CuI, PPh_3_ and Et_3_N provided the corresponding Sonogashira product 2-(4-chlorophenyl)-4-(phenylethynyl)-1-tosyl-1,2,3,6-tetrahydropyridine (7) (Scheme 7).

**Scheme 8:**
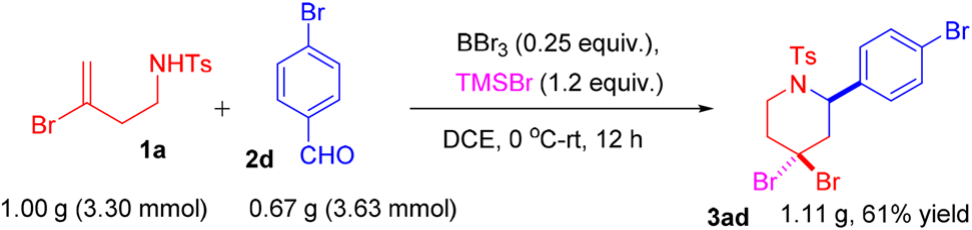
Gram-scale of the reaction.

To evaluate the scalability of this methodology, a gram-scale reaction was performed between *N*-(3-bromobut-3-en-1-yl)-4-methylbenzenesulfonamide 1a (1.00 g, 3.30 mmol) and 4-bromobenzaldehyde 2d (0.67 g, 3.63 mmol), which gave 1.11 g of the product 3ad with 61% yield (Scheme 8).

## Conclusions

In conclusion, an efficient methodology has been developed that is useful not only for the synthesis of *gem*-dihalopiperidine but also for the synthesis of 4-halo-1,2,3,6-tetrahydropyridine derivatives from alkene sulfonamides and aldehydes in good to moderate yields. The selectivity of the reaction is particularly notable, as the first pathway gives a diastereoselective product, while the second provides a regioselective product. *Gem*-dihalopiperidines can be extended to the synthesis of pyridine and piperidinone derivatives in moderate to good yields, whereas 4-halo-1,2,3,6-tetrahydropyridine can be transformed into its corresponding Sonogashira product. However, the enantioselective or chiral induction strategies for the synthesis of its chiral counterpart are a limitation of the current approach and will be explored in the future. Furthermore, the scalability of the reaction is investigated using a gram-scale experiment, and it shows its potential for large-scale applications with industrial relevance.

## Experimental

### General information

All the reagents were of reagent grade (AR grade) and were used as purchased without further purification. Silica gel (60–120 mesh size) was used for column chromatography. Reactions were monitored by TLC on silica gel GF254 (0.25 mm). Melting points were recorded in an open capillary tube and are uncorrected. Fourier transform-infrared (FT-IR) spectra were recorded as neat liquid or KBr pellets. NMR spectra were recorded in CDCl_3_ with tetramethylsilane as the internal standard for ^1^H (600 MHz, 500 MHz and 400 MHz) or ^13^C (150 MHz, 125 MHz and 100 MHz) NMR. Chemical shifts (*δ*) are reported in ppm and spin–spin coupling constants (*J*) are given in Hz. Structural assignments were made with additional information from single-crystal XRD experiments. HRMS spectra were recorded using a Q-TOF mass spectrometer.

The starting material *N*-(3-bromobut-3-en-1-yl)-4-methylbenzenesulfonamide^16*a*^ (1a), *N*-(4-bromopent-4-en-2-yl)-4-methylbenzenesulfonamide^12^ (1b), *N*-(3-bromo-1-(4-chlorophenyl)but-3-en-1-yl)-4-methylbenzenesulfonamide^12^ (1c), *N*-(3-chlorobut-3-en-1-yl)-4-methylbenzenesulfonamide^12^ (1d) and *N*-(3-iodobut-3-en-1-yl)-4-methylbenzenesulfonamide^16*b*^ (1e) was synthesized according to the reported literature. The spectroscopic data of the above compound are in good agreement with the literature. The experimental procedure and the characterization data of all compounds are given as follows.

#### General procedure for the synthesis of (3aa–3ad)

A solution of *N*-(3-bromobut-3-en-1-yl)-4-methylbenzenesulfonamide (0.6 mmol, 1.0 equiv.) and the aldehyde (0.66 mmol, 1.1 equiv.) in dry 1,2-dichloroethane (DCE) (3 mL) was added boron tribromide (BBr_3_) (1 M in DCM) (0.15 mmol, 0.25 equiv.) and trimethylsilyl bromide (TMSBr) (0.72 mmol, 1.2 equiv.) at 0 °C under the nitrogen atmosphere. The reaction mixture was then stirred at room temperature overnight, and the progress of the reaction was monitored by thin-layer chromatography (TLC) (ethyl acetate : hexane = 1 : 9). Upon completion of the reaction, it was quenched with a saturated sodium bicarbonate solution. A brine solution was then added, and the mixture was extracted with ethyl acetate (3 × 10 mL). The combined organic layer was dried over anhydrous sodium sulfate (Na_2_SO_4_), filtered, and concentrated under reduced pressure using a rotary evaporator. The crude product was purified by silica gel column chromatography, employing a mixture of ethyl acetate and hexane (1 : 9, v/v) as the eluent to obtain the final product.

#### 4,4-Dibromo-2-phenyl-1-tosylpiperidine (3aa)

Colorless solid; *R*_f_ (hexane/EtOAc, 9 : 1) 0.51; mp 136 °C, yield 230 mg, 81%; IR (KBr, neat) *ν* 2924, 1598, 1495, 1450, 1344, 1158, 1094, 950, 719, 660, 559 cm^−1^; ^1^H NMR (400 MHz, CDCl_3_) *δ* 7.50 (d, *J* = 8.4 Hz, 2H), 7.24–7.17 (m, 7H), 4.78 (t, *J* = 5.7 Hz, 1H), 3.84–3.78 (m, 1H), 3.62–3.56 (m, 1H), 3.19 (dd, *J* = 14.8, 6.4 Hz, 1H), 2.81 (ddd, *J* = 14.8, 4.8, 1.1 Hz, 1H), 2.66–2.62 (m, 2H), 2.42 (s, 3H). ^13^C{^1^H} NMR (150 MHz, CDCl_3_) *δ* 143.8, 137.7, 136.6, 129.8, 128.4, 127.8, 127.6, 127.5, 62.4, 59.0, 52.8, 47.7, 43.9, 21.8. HRMS (ESI) calcd for C_18_H_20_Br_2_NO_2_S (M + H)^+^ 473.9556, found 473.9571.

#### 4,4-Dibromo-2-(4-fluorophenyl)-1-tosylpiperidine (3ab)

Colorless solid; *R*_f_ (hexane/EtOAc, 9 : 1) 0.52; mp 143 °C, yield 242 mg, 82%; IR (KBr, neat) *ν* 2944, 1602, 1510, 1443, 1375, 1162, 1095, 1039, 918, 744, 660, 553 cm^−1^; ^1^H NMR (400 MHz, CDCl_3_) *δ* 7.48–7.45 (m, 2H), 7.23 (d, *J* = 8.0 Hz, 2H), 7.15–7.12 (m, 2H), 6.91–6.87 (m, 2H), 4.71 (t, *J* = 5.7 Hz, 1H), 3.86–3.79 (m, 1H), 3.59–3.53 (m, 1H), 3.15–3.09 (m, 1H), 2.80–2.75 (m, 1H), 2.67–2.62 (m, 2H), 2.42 (s, 3H). ^13^C{^1^H} NMR (125 MHz, CDCl_3_) *δ* 162.4 (d, *J* = 245.3 Hz), 144.0, 136.6, 133.3 (d, *J* = 3.2 Hz), 129.8, 129.4 (d, *J* = 8.1 Hz), 127.6, 115.3 (d, *J* = 21.5 Hz), 62.2, 58.7, 53.0, 47.7, 44.1, 21.8. ^19^F NMR (470 MHz, C_6_F_6_/CDCl_3_) *δ* −114.50. HRMS (ESI) calcd for C_18_H_19_Br_2_FNO_2_S (M + H)^+^ 491.9462, found 491.9441.

#### 4,4-Dibromo-2-(4-chlorophenyl)-1-tosylpiperidine (3ac)

Colorless solid; *R*_f_ (hexane/EtOAc, 9 : 1) 0.52; mp 159 °C, yield 252 mg, 83%; IR (KBr, neat) *ν* 2925, 2855, 1597, 1492, 1345, 1162, 1093, 1015, 916, 712, 656, 551 cm^−1^; ^1^H NMR (500 MHz, CDCl_3_) *δ* 7.48 (d, *J* = 7.8 Hz, 2H), 7.24 (d, *J* = 7.9 Hz, 2H), 7.17 (d, *J* = 8.8 Hz, 2H), 7.10 (d, *J* = 8.1 Hz, 2H), 4.73 (t, *J* = 5.6 Hz, 1H), 3.80–3.76 (m, 1H), 3.60–3.55 (m, 1H), 3.12 (dd, *J* = 14.8, 6.5 Hz, 1H), 2.78 (dd, *J* = 14.8, 4.7 Hz, 1H), 2.65–2.61 (m, 2H), 2.43 (s, 3H). ^13^C{^1^H} NMR (125 MHz, CDCl_3_) *δ* 144.1, 136.5, 136.2, 133.7, 129.9, 129.0, 128.5, 127.6, 62.0, 58.5, 52.6, 47.6, 43.8, 21.8. HRMS (ESI) calcd for C_18_H_19_Br_2_ClNO_2_S (M + H)^+^ 507.9166, found 507.9174.

#### 4,4-Dibromo-2-(4-bromophenyl)-1-tosylpiperidine (3ad)

Colorless solid; *R*_f_ (hexane/EtOAc, 9 : 1) 0.52; mp 149 °C, yield 274 mg, 83%; IR (KBr, neat) *ν* 2924, 1597, 1488, 1341, 1162, 1094, 1011, 915, 709, 661, 549 cm^−1^; ^1^H NMR (400 MHz, CDCl_3_) *δ* 7.48 (d, *J* = 8.3 Hz, 2H), 7.32 (d, *J* = 8.6 Hz, 2H), 7.24 (d, *J* = 8.1 Hz, 2H), 7.04 (d, *J* = 8.3 Hz, 2H), 4.72 (t, *J* = 5.6 Hz, 1H), 3.81–3.75 (m, 1H), 3.61–3.55 (m, 1H), 3.15–3.09 (m, 1H), 2.78 (dd, *J* = 14.8, 4.8, 1.2 Hz, 1H), 2.65–2.61 (m, 2H), 2.43 (s, 3H). ^13^C{^1^H} NMR (150 MHz, CDCl_3_) *δ* 144.1, 136.7, 136.5, 131.5, 129.9, 129.3, 127.6, 121.9, 61.9, 58.5, 52.5, 47.6, 43.8, 21.8. HRMS (ESI) calcd for C_18_H_19_Br_3_NO_2_S (M + H)^+^ 551.8661, found 551.8637.

#### 4,4-Dibromo-2-(3-bromophenyl)-1-tosylpiperidine (3ae)

Colorless gum; *R*_f_ (hexane/EtOAc, 9 : 1) 0.52; yield 238 mg, 72%; IR (KBr, neat) *ν* 2924, 1596, 1475, 1341, 1160, 1091, 1011, 923, 808, 725, 659, 570 cm^−1^; ^1^H NMR (600 MHz, CDCl_3_) *δ* 7.47 (d, *J* = 8.1 Hz, 2H), 7.32–7.30 (m, 1H), 7.22 (d, *J* = 8.0 Hz, 2H), 7.18–7.16 (m, 1H), 7.15–7.10 (m, 2H), 4.76 (t, *J* = 5.7 Hz, 1H), 3.81–3.77 (m, 1H), 3.63–3.59 (m, 1H), 3.12 (dd, *J* = 14.7, 6.6 Hz, 1H), 2.80 (ddd, *J* = 14.8, 4.8, 1.2 Hz, 1H), 2.66–2.60 (m, 2H), 2.42 (s, 3H). ^13^C{^1^H} NMR (150 MHz, CDCl_3_) *δ* 144.2, 139.9, 136.6, 130.9, 130.8, 130.0, 129.9, 127.4, 126.4, 122.5, 61.9, 58.4, 52.5, 47.6, 43.8, 21.8. HRMS (ESI) calcd for C_18_H_19_Br_3_NO_2_S (M + H)^+^ 551.8661, found 551.8655.

#### Methyl 4-(4,4-dibromo-1-tosylpiperidin-2-yl)benzoate (3af)

Colorless solid; *R*_f_ (hexane/EtOAc, 9 : 1) 0.48; mp 169 °C, yield 236 mg, 74%; IR (KBr, neat) *ν* 2926, 1718, 1611, 1435, 1344, 1278, 1158, 1094, 1017, 950, 917, 868, 771, 662, 550 cm^−1^; ^1^H NMR (400 MHz, CDCl_3_) *δ* 7.91 (d, *J* = 8.5 Hz, 2H), 7.55 (d, *J* = 8.4 Hz, 2H), 7.28–7.24 (m, 4H), 4.90 (t, *J* = 5.5 Hz, 1H), 3.91 (s, 3H), 3.77–3.70 (m, 1H), 3.68–3.62 (m, 1H), 3.20 (ddd, *J* = 14.8, 5.6, 1.1 Hz, 1H), 2.84 (ddd, *J* = 14.7, 5.2, 0.9 Hz, 1H), 2.65–2.56 (m, 2H), 2.43 (s, 3H). ^13^C{^1^H} NMR (150 MHz, CDCl_3_) *δ* 166.9, 144.3, 143.2, 136.3, 130.0, 129.8, 129.6, 127.6, 127.2, 61.6, 58.3, 52.4, 52.2, 47.4, 43.4, 21.8. HRMS (ESI) calcd for C_20_H_22_Br_2_NO_4_S (M + H)^+^ 531.9611, found 531.9610.

#### 4,4-Dibromo-1-tosyl-2-(4-(trifluoromethyl)phenyl)piperidine (3ag)

Colorless gum; *R*_f_ (hexane/EtOAc, 9 : 1) 0.53; yield 201 mg, 62%; IR (KBr, neat) *ν* 2926, 1620, 1598, 1324, 1160, 1116, 1123, 1069, 1017, 951, 715, 664, 583, 549 cm^−1^; ^1^H NMR (600 MHz, CDCl_3_) *δ* 7.49–7.45 (m, 4H), 7.30 (d, *J* = 8.1 Hz, 2H), 7.22 (d, *J* = 8.0 Hz, 2H), 4.84 (t, *J* = 5.8 Hz, 1H), 3.81–3.77 (m, 1H), 3.64–3.60 (m, 1H), 3.17 (dd, *J* = 14.7, 6.4 Hz, 1H), 2.82 (ddd, *J* = 14.8, 4.9, 1.3 Hz, 1H), 2.68–2.59 (m, 2H), 2.42 (s, 3H). ^13^C{^1^H} NMR (125 MHz, CDCl_3_) *δ* 144.2, 141.8, 136.5, 130.1 (q, *J* = 32.2 Hz), 130.0, 127.9, 127.5, 125.3 (q, *J* = 3.7 Hz), 124.2 (q, *J* = 270.4 Hz), 61.6, 58.5, 52.4, 47.5, 43.7, 21.8. ^19^F NMR (470 MHz, CDCl_3_/C_6_F_6_) *δ* −62.50. HRMS (ESI) calcd for C_19_H_19_Br_2_F_3_NO_2_S (M + H)^+^ 541.9430, found 541.9431.

#### 4,4-Dibromo-2-(4-nitrophenyl)-1-tosylpiperidine (3ah)

Colorless solid; *R*_f_ (hexane/EtOAc, 9 : 1) 0.46; mp 193 °C, yield 258 mg, 83%; IR (KBr, neat) *ν* 2927, 1597, 1522, 1349, 1159, 1092, 721, 663, 550 cm^−1^; ^1^H NMR (500 MHz, CDCl_3_) *δ* 8.11 (d, *J* = 8.8 Hz, 2H), 7.57–7.56 (m, 2H), 7.40 (d, *J* = 8.3 Hz, 2H), 7.29 (d, *J* = 7.9 Hz, 2H), 4.96–4.94 (m, 1H), 3.72–3.64 (m, 2H), 3.18 (dd, *J* = 15.0, 5.4 Hz, 1H), 2.84 (dd, *J* = 14.9, 5.3 Hz, 1H), 2.65–2.60 (m, 1H), 2.57–2.52 (m, 1H), 2.44 (s, 3H). ^13^C{^1^H} NMR (125 MHz, CDCl_3_) *δ* 147.4, 145.8, 144.6, 136.0, 130.1, 128.0, 127.5, 123.7, 60.9, 57.9, 52.0, 47.1, 43.3, 21.8. HRMS (ESI) calcd for C_18_H_19_Br_2_N_2_O_4_S (M + H)^+^ 518.9407, found 518.9379.

#### 2-([1,1′-Biphenyl]-4-yl)-4,4-dibromo-1-tosylpiperidine (3ai)

Yellow solid; *R*_f_ (hexane/EtOAc, 9 : 1) 0.53; mp 152 °C, yield 214 mg, 65%; IR (KBr, neat) *ν* 3030, 2924, 1598, 1488, 1343, 1262, 1157, 1093, 1008, 949, 866, 758, 728, 697, 550 cm^−1^; ^1^H NMR (400 MHz, CDCl_3_) *δ* 7.56–7.54 (m, 2H), 7.50–7.421 (m, 6H), 7.37–7.33 (m, 1H), 7.25–7.19 (m, 4H), 4.82–4.79 (m, 1H), 3.91–3.85 (m, 1H), 3.64–3.58 (m, 1H), 3.22 (dd, *J* = 14.7, 6.9 Hz, 1H), 2.85 (ddd, *J* = 14.8, 4.6, 1.1 Hz, 1H), 2.70–2.66 (m, 2H), 2.40 (s, 3H). ^13^C{^1^H} NMR (125 MHz, CDCl_3_) *δ* 143.7, 140.7, 136.8, 136.5, 129.7, 129.0, 128.3, 127.7, 127.6, 127.2, 127.0, 62.6, 59.1, 52.8, 47.8, 44.1, 21.8. HRMS (ESI) calcd for C_24_H_24_Br_2_NO_2_S (M + H)^+^ 549.9869, found 549.9863.

#### 4,4-Dibromo-2-(*p*-tolyl)-1-tosylpiperidine (3aj)

Brown solid; *R*_f_ (hexane/EtOAc, 9 : 1) 0.52; mp 127 °C, yield 242 mg, 83%; IR (KBr, neat) *ν* 2923, 1598, 1511, 1343, 1161, 812, 726, 673, 567, 546 cm^−1^; ^1^H NMR (600 MHz, CDCl_3_) *δ* 7.48 (d, *J* = 8.0 Hz, 2H), 7.21 (d, *J* = 8.1 Hz, 2H), 7.05 (d, *J* = 8.0 Hz, 2H), 7.01 (d, *J* = 8.0 Hz, 2H), 4.68 (t, *J* = 5.6 Hz, 1H), 3.85–3.81 (m, 1H), 3.56–3.52 (m, 1H), 3.15 (dd, *J* = 14.7, 6.8 Hz, 1H), 2.78 (ddd, *J* = 14.8, 4.6, 1.3 Hz, 1H), 2.68–2.62 (m, 2H), 2.42 (s, 3H), 2.30 (s, 3H). ^13^C{^1^H} NMR (150 MHz, CDCl_3_) *δ* 143.7, 137.6, 136.6, 134.6, 129.7, 129.0, 127.7, 127.6, 62.7, 59.1, 53.1, 47.8, 44.1, 21.8, 21.3. HRMS (ESI) calcd for C_19_H_22_Br_2_NO_2_S (M + H)^+^ 487.9713, found 487.9707.

#### 4,4-Dibromo-2-(4-(*tert*-butyl)phenyl)-1-tosylpiperidine (3al)

Pale yellow solid; *R*_f_ (hexane/EtOAc, 9 : 1) 0.52; mp 137 °C, yield 206 mg, 65%; IR (KBr, neat) *ν* 2961, 1512, 1494, 1345, 1322, 1155, 1092, 1017, 714, 656, 547 cm^−1^; ^1^H NMR (400 MHz, CDCl_3_) *δ* 7.38 (d, *J* = 8.4 Hz, 2H), 7.16–7.12 (m, 4H), 7.06–7.04 (m, 2H), 4.67 (dd, *J* = 7.8, 4.1 Hz, 1H), 3.95–3.89 (m, 1H), 3.57–3.51 (m, 1H), 3.16 (dd, *J* = 14.7, 7.8 Hz, 1H), 2.81–2.76 (m, 1H), 2.70–2.67 (m, 2H), 2.38 (s, 3H), 1.27 (s, 9H). ^13^C{^1^H} NMR (125 MHz, CDCl_3_) *δ* 150.9, 143.3, 137.2, 133.9, 129.5, 127.9, 127.6, 125.1, 63.2, 59.5, 53.1, 47.9, 44.5, 34.6, 31.5, 21.7. HRMS (ESI) calcd for C_22_H_28_Br_2_NO_2_S (M + H)^+^ 530.0182, found 530.0175.

#### 4,4-Dibromo-2-(3,4-dimethylphenyl)-1-tosylpiperidine (3am)

Colorless gum; *R*_f_ (hexane/EtOAc, 9 : 1) 0.52; yield 243 mg, 81%; IR (KBr, neat) *ν* 2922, 1597, 1504, 1451, 1342, 1321, 1156, 1092, 1017, 920, 813, 723, 659, 546, 449 cm^−1^; ^1^H NMR (400 MHz, CDCl_3_) *δ* 7.43 (d, *J* = 8.3 Hz, 2H), 7.17 (d, *J* = 8.0 Hz, 2H), 6.97 (d, *J* = 7.8 Hz, 1H), 6.92 (dd, *J* = 7.8, 2.0 Hz, 1H), 6.80–6.79 (m, 1H), 4.65–4.62 (m, 1H), 3.90–3.84 (m, 1H), 3.57–3.51 (m, 1H), 3.15 (dd, *J* = 14.7, 7.4 Hz, 1H), 2.82–2.76 (m, 1H), 2.69–2.66 (m, 2H), 2.40 (s, 3H), 2.19 (s, 3H), 2.09 (s, 3H). ^13^C{^1^H} NMR (125 MHz, CDCl_3_) *δ* 143.5, 136.8, 136.3, 136.2, 134.6, 129.5, 129.4, 129.2, 127.6, 125.4, 63.0, 59.3, 53.2, 47.9, 44.3, 21.7, 19.9, 19.6. HRMS (ESI) calcd for C_20_H_24_Br_2_NO_2_S (M + H)^+^ 501.9869, found 501.9869.

#### 4,4-Dibromo-2-(naphthalen-2-yl)-1-tosylpiperidine (3an)

Colorless solid; *R*_f_ (hexane/EtOAc, 9 : 1) 0.50; mp 159 °C, yield 254 mg, 81%; IR (KBr, neat) *ν* 3057, 2925, 1598, 1438, 1343, 1158, 1093, 1016, 816, 662, 559, 478 cm^−1^; ^1^H NMR (400 MHz, CDCl_3_) *δ* 7.79–7.77 (m, 1H), 7.69 (d, *J* = 8.6 Hz, 1H), 7.65–7.62 (m, 1H), 7.49–7.42 (m, 5H), 7.33 (dd, *J* = 8.6, 1.9 Hz, 1H), 7.15–7.04 (m, 2H), 4.91–4.88 (m, 1H), 3.94–3.89 (m, 1H), 3.68–3.62 (m, 1H), 3.30 (dd, *J* = 14.8, 6.8 Hz, 1H), 2.89 (dd, *J* = 14.7, 4.6 Hz, 1H), 2.72–2.70 (m, 2H), 2.35 (s, 3H). ^13^C{^1^H} NMR (125 MHz, CDCl_3_) *δ* 143.9, 136.7, 135.0, 133.2, 133.0, 129.7, 128.1, 128.0, 127.8, 127.6, 126.7, 126.4, 126.3, 125.6, 62.5, 59.5, 53.0, 47.8, 44.1, 21.7. HRMS (ESI) calcd for C_22_H_22_Br_2_NO_2_S (M + H)^+^ 523.9713, found 523.9699.

#### 4,4-Dibromo-2-(naphthalen-1-yl)-1-tosylpiperidine (3ao)

Colorless solid; *R*_f_ (hexane/EtOAc, 9 : 1) 0.51; mp 149 °C, yield 229 mg, 73%; IR (KBr, neat) *ν* 3051, 1598, 1510, 1494, 1439, 1342, 1316, 1264, 1152, 1088, 1007, 777, 694, 571, 531, 481 cm^−1^; ^1^H NMR (400 MHz, CDCl_3_) *δ* 7.75–7.73 (m, 1H), 7.66 (d, *J* = 8.2 Hz, 1H), 7.62–7.58 (m, 1H), 7.53 (d, *J* = 7.2 Hz, 1H), 7.40–7.31 (m, 3H), 6.88 (d, *J* = 8.0 Hz, 2H), 6.57 (d, *J* = 8.0 Hz, 2H), 5.20 (dd, *J* = 10.9, 2.6 Hz, 1H), 4.34 (dt, *J* = 14.2, 3.7 Hz, 1H), 3.75 (dd, *J* = 14.2, 10.8 Hz, 1H), 3.64 (ddd, *J* = 14.1, 10.9, 2.9 Hz, 1H), 2.98–2.90 (m, 2H), 2.86 (dq, *J* = 14.6, 2.8 Hz, 1H), 2.10 (s, 3H). ^13^C{^1^H} NMR (100 MHz, CDCl_3_) *δ* 142.4, 135.9, 133.5, 131.7, 131.5, 129.6, 128.7, 128.3, 127.3, 127.0, 126.5, 125.5, 125.0, 123.2, 65.8, 57.5, 51.9, 48.5, 47.0, 21.4. HRMS (ESI) calcd for C_22_H_22_Br_2_NO_2_S (M + H)^+^ 523.9713, found 523.9715.

#### 4,4-Dibromo-1-tosylpiperidine (3ap)

Colorless solid; *R*_f_ (hexane/EtOAc, 9 : 1) 0.51; mp 149 °C, yield 131 mg, 55%; IR (KBr, neat) *ν* 2927, 1598, 1350, 1163, 933, 715, 546 cm^−1^; ^1^H NMR (400 MHz, CDCl_3_) *δ* 7.65 (d, *J* = 8.3 Hz, 2H), 7.34 (d, *J* = 8.0 Hz, 2H), 3.17 (t, *J* = 5.3 Hz, 4H), 2.63 (t, *J* = 5.3 Hz, 4H), 2.44 (s, 3H). ^13^C{^1^H} NMR (125 MHz, CDCl_3_) *δ* 144.2, 133.6, 130.1, 127.7, 64.6, 47.5, 44.7, 21.8. HRMS (ESI) calcd for C_12_H_16_Br_2_NO_2_S (M + H)^+^ 397.9243, found 397.9240.

#### 4,4-Dibromo-2-ethyl-1-tosylpiperidine (3aq)

Colorless solid; *R*_f_ (hexane/EtOAc, 9 : 1) 0.52; mp 127 °C, yield 201 mg, 79%; IR (KBr, neat) *ν* 2970, 2931, 2876, 1597, 1494, 1455, 1341, 1319, 1156, 1092, 1051, 957, 814, 718, 648, 552 cm^−1^; ^1^H NMR (600 MHz, CDCl_3_) *δ* 7.70 (d, *J* = 8.3 Hz, 2H), 7.31 (d, *J* = 8.4 Hz, 2H), 3.88–3.84 (m, 1H), 3.68 (dtd, *J* = 14.8, 3.9, 1.4 Hz, 1H), 3.45–3.41 (m, 1H), 2.81 (dt, *J* = 15.0, 2.3 Hz, 1H), 2.65 (dd, *J* = 15.0, 6.3 Hz, 1H), 2.62–2.58 (m, 1H), 2.47–2.44 (m, 1H), 2.43 (s, 3H), 1.89 (p, *J* = 7.5 Hz, 2H), 0.86 (t, *J* = 7.4 Hz, 3H). ^13^C{^1^H} NMR (150 MHz, CDCl_3_) *δ* 143.9, 138.1, 130.1, 127.3, 62.3, 57.1, 48.5, 48.1, 40.7, 24.6, 21.8, 11.6. HRMS (ESI) calcd for C_14_H_20_Br_2_NO_2_S (M + H)^+^ 425.9556, found 425.9557.

#### 4,4-Dibromo-2-propyl-1-tosylpiperidine (3ar)

Brown solid; *R*_f_ (hexane/EtOAc, 9 : 1) 0.52; mp 126 °C, yield 211 mg, 80%; IR (KBr, neat) *ν* 2960, 2931, 2872, 1597, 1494, 1456, 1320, 1157, 1092, 1062, 926, 815, 710, 650, 552 cm^−1^; ^1^H NMR (600 MHz, CDCl_3_) *δ* 7.70 (d, *J* = 8.2 Hz, 2H), 7.31 (d, *J* = 8.0 Hz, 2H), 3.99–3.95 (m, 1H), 3.67 (dtd, *J* = 14.8, 3.9, 1.4 Hz, 1H), 3.47–3.42 (m, 1H), 2.78 (dt, *J* = 14.9, 2.2 Hz, 1H), 2.65 (dd, *J* = 15.0, 6.3 Hz, 1H), 2.61–2.58 (m, 1H), 2.43 (s, 3H), 2.42–2.41 (m, 1H), 1.85–1.81 (m, 2H), 1.32–1.25 (m, 2H), 0.87 (t, *J* = 7.4 Hz, 3H). ^13^C{^1^H} NMR (150 MHz, CDCl_3_) *δ* 143.9, 138.1, 130.1, 127.3, 62.3, 55.3, 48.9, 48.1, 40.7, 33.7, 21.8, 20.2, 13.9. HRMS (ESI) calcd for C_15_H_22_Br_2_NO_2_S (M + H)^+^ 439.9713, found 439.9712.

#### 4,4-Dibromo-2-hexyl-1-tosylpiperidine (3as)

Pale yellow gum; *R*_f_ (hexane/EtOAc, 9 : 1) 0.48; yield 184 mg, 64%; IR (KBr, neat) *ν* 2927, 15 967, 1494, 1322, 1157, 812, 651 cm^−1^; ^1^H NMR (400 MHz, CDCl_3_) *δ* 7.70 (d, *J* = 8.3 Hz, 2H), 7.30 (d, *J* = 8.0 Hz, 2H), 3.96–3.90 (m, 1H), 3.69 (dtd, *J* = 14.8, 3.9, 1.3 Hz, 1H), 3.47–3.40 (m, 1H), 2.79 (dt, *J* = 15.0, 2.3 Hz, 1H), 2.67 (dd, *J* = 15.0, 6.2 Hz, 1H), 2.63–2.58 (m, 1H), 2.49–2.45 (m, 1H), 2.42 (s, 3H), 1.87–1.77 (m, 2H), 1.25–1.17 (m, 8H), 0.86 (t, *J* = 6.9 Hz, 3H). ^13^C{^1^H} NMR (100 MHz, CDCl_3_) *δ* 143.9, 138.1, 130.1, 127.3, 62.4, 55.6, 48.9, 48.2, 40.7, 31.8, 31.5, 29.0, 27.0, 22.8, 21.8, 14.3. HRMS (ESI) calcd for C_18_H_28_Br_2_NO_2_S (M + H)^+^ 482.0182, found 482.0181.

#### 4,4-Dibromo-2-cyclopropyl-1-tosylpiperidine (3at)

Pale yellow solid; *R*_f_ (hexane/EtOAc, 9 : 1) 0.53; mp 136 °C, yield 157 mg, 60%; IR (KBr, neat) *ν* 2962, 1597, 1493, 1383, 1255, 1155, 1093, 755, 711, 649, 553 cm^−1^; ^1^H NMR (400 MHz, CDCl_3_) *δ* 7.71 (d, *J* = 8.1 Hz, 2H), 7.32 (d, *J* = 7.9 Hz, 2H), 4.03–3.95 (m, 1H), 3.71 (dt, *J* = 14.8, 3.6 Hz, 1H), 3.47–3.42 (m, 1H), 3.38 (t, *J* = 6.2 Hz, 2H), 2.75 (dt, *J* = 15.1, 2.3 Hz, 1H), 2.65 (dd, *J* = 15.1, 6.4 Hz, 1H), 2.60–2.54 (m, 1H), 2.43 (s, 3H), 2.40–2.33 (m, 1H), 2.20–2.10 (m, 1H), 1.89–1.81 (m, 2H). ^13^C{^1^H} NMR (125 MHz, CDCl_3_) *δ* 144.2, 137.7, 130.3, 127.3, 61.6, 54.6, 49.2, 47.7, 40.5, 33.3, 30.4, 30.1, 21.8. HRMS (ESI) calcd for C_15_H_20_Br_2_NO_2_S (M + H)^+^ 437.9556, found 437.9544.

#### 4,4-Dibromo-2-cyclohexyl-1-tosylpiperidine (3au)

Colorless gum; *R*_f_ (hexane/EtOAc, 9 : 1) 0.51; yield 175 mg, 61%; IR (KBr, neat) *ν* 2926, 1599, 1494, 1342, 1156, 658, 612 cm^−1^; ^1^H NMR (400 MHz, CDCl_3_) *δ* 7.71 (d, *J* = 8.3 Hz, 2H), 7.32 (d, *J* = 8.0 Hz, 2H), 3.73–3.63 (m, 2H), 3.35–3.28 (m, 1H), 3.03 (dt, *J* = 15.3, 1.9 Hz, 1H), 2.53–2.44 (m, 2H), 2.43 (s, 3H), 2.37–2.33 (m, 1H), 2.31–2.25 (m, 1H), 1.96–1.91 (m, 1H), 1.79–1.70 (m, 3H), 1.66–1.58 (m, 2H), 1.15–1.07 (m, 2H), 0.86–0.72 (m, 2H). ^13^C{^1^H} NMR (150 MHz, CDCl_3_) *δ* 143.9, 138.1, 130.1, 127.4, 62.3, 60.6, 47.6, 45.1, 40.4, 36.2, 31.1, 30.3, 26.3, 26.2, 26.0, 21.8. HRMS (ESI) calcd for C_18_H_26_Br_2_NO_2_S (M + H)^+^ 480.0026, found 480.0025.

#### 4-Bromo-4-chloro-2-(*p*-tolyl)-1-tosylpiperidine (diastereomers, 9 : 1) (3dj)

Colorless solid; *R*_f_ (hexane/EtOAc, 9 : 1) 0.51; mp 102 °C, yield 204 mg, 77%; IR (KBr, neat) *ν* 2924, 1597, 1512, 1344, 1154, 734, 660, 556 cm^−1^; ^1^H NMR (400 MHz, CDCl_3_) *δ* 7.55 (d, *J* = 8.3 Hz, 2H, major), 7.46 (d, *J* = 8.4 Hz, 2H, minor), 7.24 (d, *J* = 8.0 Hz, 2H), 7.08–7.01 (m, 4H), 4.86 (t, *J* = 5.3 Hz, 1H, major), 4.67 (dd, *J* = 7.4, 4.5 Hz, 1H, minor), 3.84–3.77 (m, 1H), 3.67–3.61 (m, 1H), 3.13–3.08 (m, 1H), 2.77–2.72 (m, 1H), 2.56–2.49 (m, 2H), 2.42 (s, 3H), 2.30 (s, 3H). ^13^C{^1^H} NMR (125 MHz, CDCl_3_) *δ* 143.8, 137.3, 136.9, 134.7, 129.8, 129.1, 127.5, 127.1, 76.0, 74.7, 58.6, 58.1, 51.0, 46.5, 46.4, 43.7, 42.8, 21.8, 21.2. HRMS (ESI) calcd for C_19_H_22_BrClNO_2_S (M + H)^+^ 442.0238, found 442.0227.

#### 4-Bromo-2-(4-chlorophenyl)-4-iodo-1-tosylpiperidine (diastereomers, 9 : 1) (3ed)

Colorless gum; *R*_f_ (hexane/EtOAc, 9 : 1) 0.52; yield 202 mg, 61%; IR (KBr, neat) *ν* 2925, 1597, 1491, 1343, 1161, 712, 666, 551 cm^−1^; ^1^H NMR (400 MHz, CDCl_3_) *δ* 7.65 (d, *J* = 7.6 Hz, 2H, minor), 7.50 (d, *J* = 8.2 Hz, 2H, minor), 7.41 (d, *J* = 8.2 Hz, 2H, major), 7.22 (d, *J* = 8.1 Hz, 2H, major), 7.17 (d, *J* = 8.6 Hz, 2H), 7.10 (d, *J* = 8.6 Hz, 2H), 4.60 (dd, *J* = 5.5, 5.5 Hz, 1H, minor), 4.52 (dd, *J* = 8.0, 4.1 Hz, 1H, major), 3.66–3.60 (m, 1H), 3.45–3.41 (m, 1H), 3.17 (dd, *J* = 14.8, 7.9 Hz, 1H), 2.89–2.82 (m, 1H), 2.76–2.71 (m, 1H), 2.65–2.59 (m, 1H), 2.42 (s, 3H). ^13^C{^1^H} NMR (100 MHz, CDCl_3_) *δ* 144.0, 136.0, 135.8, 133.9, 129.7, 129.6, 128.5, 127.7, 59.4, 56.0, 50.3, 50.0, 45.4, 45.1, 29.3, 21.8. HRMS (ESI) calcd for C_18_H_19_BrClINO_2_S (M + H)^+^ 553.9048, found 553.9027.

#### General procedure for the synthesis of (4aa–4dj)

A solution of *N*-(3-bromobut-3-en-1-yl)-4-methylbenzene-sulfonamide (0.6 mmol, 1.0 equiv.) and the aldehyde (0.66 mmol, 1.1 equiv.) in dry 1,2-dichloroethane (DCE) (3 mL) was cooled to 0 °C under a nitrogen atmosphere. To this solution, boron tribromide (BBr_3_) (1 M in DCM) (0.15 mmol, 0.25 equiv.) and trimethylsilyl bromide (TMSBr) (0.72 mmol, 1.2 equiv.) were added. The reaction mixture was then stirred at room temperature for overnight, and progress of the reaction was monitored by thin-layer chromatography (TLC) (ethyl acetate: hexane = 1 : 9). Once the starting material was fully consumed, 1,8-diazabicyclo[5.4.0]undec-7-ene (DBU) (24 mmol, 40.0 equiv.) was added, and the mixture was stirred in an oil bath at 80 °C for 4 to 6 hours. The progress of the reaction was monitored by TLC (ethyl acetate : hexane = 1 : 9). After completion, it was allowed to cool to room temperature, brine was added, and the organic layer was extracted with EtOAc (3 × 10 mL). The combined organic layers were dried over anhydrous sodium sulfate (Na_2_SO_4_), filtered, and concentrated under reduced pressure by rotary evaporator and purified by column chromatography over silica gel using hexane/ethyl acetate (9 : 1, v/v) as eluent to get the products.

#### 4-Bromo-2-phenyl-1-tosyl-1,2,3,6-tetrahydropyridine (4aa)

Colorless solid; *R*_f_ (hexane/EtOAc, 9 : 1) 0.52; mp 116 °C, yield 157 mg, 67%; IR (KBr, neat) *ν* 2926, 2851, 1663, 1597, 1493, 1441, 1343, 1160, 1093, 1011, 912, 811, 731, 571, 492, 444 cm^−1^; ^1^H NMR (500 MHz, CDCl_3_) *δ* 7.70 (d, *J* = 8.0 Hz, 2H), 7.32–7.28 (m, 7H), 5.92 (s, 1H), 5.30 (d, *J* = 5.9 Hz, 1H), 4.17–4.12 (m, 1H), 3.34 (dt, *J* = 18.5, 2.1 Hz, 1H), 2.74–2.68 (m, 2H), 2.44 (s, 3H). ^13^C{^1^H} NMR (125 MHz, CDCl_3_) *δ* 143.9, 138.0, 137.5, 130.0, 128.9, 128.2, 127.4, 127.2, 125.1, 118.5, 54.8, 42.6, 35.9, 21.8. HRMS (ESI) calcd for C_18_H_19_BrNO_2_S (M + H)^+^ 392.0315, found 392.0307.

#### 4-Bromo-2-(4-chlorophenyl)-1-tosyl-1,2,3,6-tetrahydro-pyridine (4ac)

Brown gum; *R*_f_ (hexane/EtOAc, 9 : 1) 0.54; yield 176 mg, 69%; IR (KBr, neat) *ν* 2925, 2852, 1660, 1597, 1493, 1443, 1346, 1160, 1093, 1015, 911, 814, 733, 711, 654, 573, 491, 444 cm^−1^; ^1^H NMR (400 MHz, CDCl_3_) *δ* 7.69 (d, *J* = 8.0 Hz, 2H), 7.31–7.28 (m, 4H), 7.25–7.24 (m, 2H), 5.93–5.92 (m, 1H), 5.26 (t, *J* = 3.9 Hz, 1H), 4.14 (dd, *J* = 18.6, 4.7 Hz, 1H), 3.32 (dq, *J* = 18.4, 3.0 Hz, 1H), 2.70–2.68 (m, 2H), 2.44 (s, 3H). ^13^C{^1^H} NMR (100 MHz, CDCl_3_) *δ* 144.1, 137.3, 136.5, 134.1, 130.1, 129.1, 128.9, 127.2, 125.2, 118.1, 54.2, 42.6, 35.8, 21.8. HRMS (ESI) calcd for C_18_H_18_BrClNO_2_S (M + H)^+^ 425.9925, found 425.9899.

#### 4-Bromo-2-(4-bromophenyl)-1-tosyl-1,2,3,6-tetrahydro-pyridine (4ad)

Colorless solid; *R*_f_ (hexane/EtOAc, 9 : 1) 0.54; mp 119 °C, yield 198 mg, 70%; IR (KBr, neat) *ν* 2922, 2851, 1660, 1596, 1489, 1442, 1344, 1159, 1095, 909, 814, 708, 696, 544, 481, 410 cm^−1^; ^1^H NMR (400 MHz, CDCl_3_) *δ* 7.71–7.68 (m, 2H), 7.45–7.42 (m, 2H), 7.30 (d, *J* = 8.0 Hz, 2H), 7.18 (d, *J* = 8.4 Hz, 2H), 5.93–5.91 (m, 1H), 5.24 (t, *J* = 3.9 Hz, 1H), 4.17–4.11 (m, 1H), 3.35–3.28 (m, 1H), 2.70–2.68 (m, 2H), 2.44 (s, 3H). ^13^C{^1^H} NMR (100 MHz, CDCl_3_) *δ* 144.1, 137.3, 137.0, 132.0, 130.1, 129.2, 127.2, 125.2, 122.3, 118.1, 54.2, 42.6, 35.7, 21.8. HRMS (ESI) calcd for C_18_H_18_Br_2_NO_2_S (M + H)^+^ 471.9400, found 471.9387.

#### 2-([1,1′-Biphenyl]-4-yl)-4-bromo-1-tosyl-1,2,3,6-tetrahydro-pyridine (4ai)

Brown solid; *R*_f_ (hexane/EtOAc, 9 : 1) 0.55; mp 114 °C, yield 154 mg, 55%; IR (KBr, neat) *ν* 3030, 2922, 2851, 1658, 1598, 1442, 1344, 1159, 1094, 1008, 909, 814, 766, 720, 658, 568, 543 cm^−1^; ^1^H NMR (400 MHz, CDCl_3_) *δ* 7.73 (d, *J* = 8.3 Hz, 2H), 7.58–7.54 (m, 4H), 7.46–7.42 (m, 2H), 7.40–7.35 (m, 3H), 7.30 (d, *J* = 8.1 Hz, 2H), 5.96–5.95 (m, 1H), 5.35–5.33 (m, 1H), 4.20–4.14 (m, 1H), 3.44–3.38 (m, 1H), 2.83–2.70 (m, 2H), 2.44 (s, 3H). ^13^C{^1^H} NMR (100 MHz, CDCl_3_) *δ* 143.9, 141.1, 140.6, 137.5, 137.0, 130.0, 129.1, 127.9, 127.7, 127.6, 127.3, 127.2, 125.2, 118.4, 54.6, 42.7, 35.9, 21.8. HRMS (ESI) calcd for C_24_H_23_BrNO_2_S (M + H)^+^ 468.0628, found 468.0603.

#### 4-Bromo-2-(*p*-tolyl)-1-tosyl-1,2,3,6-tetrahydropyridine (4aj)

Pale yellow solid; *R*_f_ (hexane/EtOAc, 9 : 1) 0.54; mp 144 °C, yield 170 mg, 70%; IR (KBr, neat) *ν* 2922, 1659, 1597, 1514, 1345, 1159, 1095, 909, 813, 725, 657, 575, 550 cm^−1^; ^1^H NMR (400 MHz, CDCl_3_) *δ* 7.70 (d, *J* = 8.3 Hz, 2H), 7.29 (d, *J* = 8.1 Hz, 2H), 7.20 (d, *J* = 8.2 Hz, 2H), 7.12 (d, *J* = 8.0 Hz, 2H), 5.92–5.89 (m, 1H), 5.26 (d, *J* = 5.7 Hz, 1H), 4.16–4.09 (m, 1H), 3.37–3.30 (m, 1H), 2.72–2.66 (m, 2H), 2.44 (s, 3H), 2.32 (s, 3H). ^13^C{^1^H} NMR (100 MHz, CDCl_3_) *δ* 143.8, 138.0, 137.6, 134.9, 130.0, 129.5, 127.3, 127.2, 125.1, 118.6, 54.5, 42.5, 35.9, 21.8, 21.3. HRMS (ESI) calcd for C_18_H_21_BrNO_2_S (M + H)^+^ 406.0471, found 406.0470.

#### 4-Bromo-2-(naphthalen-1-yl)-1-tosyl-1,2,3,6-tetrahydro-pyridine (4ao)

Colorless solid; *R*_f_ (hexane/EtOAc, 9 : 1) 0.52; mp 146 °C, yield, 164 mg, 62%; IR (KBr, neat) *ν* 3051, 2924, 1656, 1598, 1511, 1439, 1340, 1317, 1159, 1092, 1049, 779, 717, 664, 572, 542 cm^−1^; ^1^H NMR (500 MHz, CDCl_3_) *δ* 8.53 (d, *J* = 8.6 Hz, 1H), 7.79 (d, *J* = 8.2 Hz, 1H), 7.76–7.74 (m, 1H), 7.69 (d, *J* = 7.9 Hz, 2H), 7.55 (t, *J* = 7.8 Hz, 1H), 7.45 (t, *J* = 7.6 Hz, 1H), 7.32–7.29 (m, 2H), 7.20 (d, *J* = 8.1 Hz, 2H), 6.03 (d, *J* = 7.2 Hz, 1H), 5.88 (s, 1H), 3.93 (dd, *J* = 17.5, 4.3 Hz, 1H), 3.13 (d, *J* = 19.3 Hz, 1H), 2.90–2.84 (m, 1H), 2.70 (d, *J* = 18.4 Hz, 1H), 2.36 (s, 3H). ^13^C{^1^H} NMR (125 MHz, CDCl_3_) *δ* 144.2, 136.8, 134.3, 133.3, 131.7, 129.9, 129.8, 128.9, 127.9, 127.1, 126.3, 125.4, 124.9, 124.5, 124.4, 119.5, 52.0, 42.8, 36.5, 21.8. HRMS (ESI) calcd for C_22_H_21_BrNO_2_S (M + H)^+^ 442.0471, found 442.0446.

#### 4-Bromo-2-ethyl-1-tosyl-1,2,3,6-tetrahydropyridine (4aq)

Pale yellow gum; *R*_f_ (hexane/EtOAc, 9 : 1) 0.54; yield 128 mg, 62%; IR (KBr, neat) *ν* 2968, 2933, 2876, 1654, 1598, 1494, 1454, 1335, 1266, 1155, 1092, 1042, 951, 882, 814, 717, 648, 540 cm^−1^; ^1^H NMR (400 MHz, CDCl_3_) *δ* 7.62 (d, *J* = 8.3 Hz, 2H), 7.21 (d, *J* = 8.1 Hz, 2H), 5.96–5.94 (m, 1H), 4.22–4.17 (m, 1H), 3.80–3.75 (m, 1H), 3.20–3.12 (m, 1H), 2.35 (s, 3H), 2.09–1.96 (m, 2H), 1.59–1.51 (m, 2H), 0.91 (t, *J* = 7.4 Hz, 3H). ^13^C{^1^H} NMR (125 MHz, CDCl_3_) *δ* 143.7, 138.1, 129.9, 129.2, 127.1, 119.5, 57.3, 39.7, 32.9, 27.9, 21.7, 10.9. HRMS (ESI) calcd for C_14_H_19_BrNO_2_S (M + H)^+^ 344.0315, found 344.0316.

#### 4-Chloro-2-(4-fluorophenyl)-1-tosyl-1,2,3,6-tetrahydro-pyridine (4db)

Colorless solid; *R*_f_ (hexane/EtOAc, 9 : 1) 0.55; mp 77 °C, yield, 151 mg, 69%; IR (KBr, neat) *ν* 2962, 1603, 1510, 1343, 1159, 1484, 1442, 1093, 740, 661, 484, 403 cm^−1^; ^1^H NMR (400 MHz, CDCl_3_) *δ* 7.69 (d, *J* = 8.4 Hz, 2H), 7.31–7.27 (m, 4H), 7.02–6.98 (m, 2H), 5.72–5.70 (m, 1H), 5.31 (d, *J* = 6.1 Hz, 1H), 4.22–4.16 (m, 1H), 3.37–3.31 (m, 1H), 2.67–2.56 (m, 2H), 2.43 (s, 3H). ^13^C{^1^H} NMR (125 MHz, CDCl_3_) *δ* 162.5 (d, *J* = 245.7 Hz), 144.0, 137.4, 133.8 (d, *J* = 3.2 Hz), 130.1, 129.2 (d, *J* = 8.2 Hz), 129.0, 127.2, 121.1, 115.7 (d, *J* = 21.2 Hz), 53.4, 41.4, 33.9, 21.8. ^19^F NMR (470 MHz, CDCl_3_/C_6_F_6_) *δ* −114.01. HRMS (ESI) calcd for C_18_H_18_ClFNO_2_S (M + H)^+^ 366.0726, found 366.0708.

#### 2-(3-Bromophenyl)-4-chloro-1-tosyl-1,2,3,6-tetrahydro-pyridine (4de)

Colorless solid; *R*_f_ (hexane/EtOAc, 9 : 1) 0.54; mp 118 °C, yield 156 mg, 61%; IR (KBr, neat) *ν* 2924, 1659, 1596, 1488, 1441, 1344, 1159, 1093, 906, 814, 707, 481, 409 cm^−1^; ^1^H NMR (500 MHz, CDCl_3_) *δ* 7.69 (d, *J* = 7.8 Hz, 2H), 7.40 (d, *J* = 7.9 Hz, 1H), 7.35 (s, 1H), 7.30 (d, *J* = 7.9 Hz, 2H), 7.24 (d, *J* = 7.8 Hz, 1H), 7.19 (t, *J* = 7.8 Hz, 1H), 5.73 (s, 1H), 5.29 (d, *J* = 6.4 Hz, 1H), 4.23 (d, *J* = 18.2 Hz, 1H), 3.41–3.36 (m, 1H), 2.67–2.61 (m, 1H), 2.56 (d, *J* = 17.7 Hz, 1H), 2.44 (s, 3H). ^13^C{^1^H} NMR (125 MHz, CDCl_3_) *δ* 144.1, 140.4, 137.3, 131.4, 130.6, 130.5, 130.1, 128.8, 127.2, 125.9, 123.0, 121.0, 53.7, 41.6, 33.9, 21.8. HRMS (ESI) calcd for C_18_H_18_BrClNO_2_S (M + H)^+^ 425.9925, found 425.9904.

#### 4-Chloro-2-(*p*-tolyl)-1-tosyl-1,2,3,6-tetrahydropyridine (4dj)

Pale yellow solid; *R*_f_ (hexane/EtOAc, 9 : 1) 0.53; mp 130 °C, yield 152 mg, 70%; IR (KBr, neat) *ν* 2923, 1658, 1597, 1514, 1342, 1159, 1095, 906, 811, 575, 551 cm^−1^; ^1^H NMR (400 MHz, CDCl_3_) *δ* 7.70 (d, *J* = 8.3 Hz, 2H), 7.28 (d, *J* = 7.9 Hz, 2H), 7.19 (d, *J* = 8.2 Hz, 2H), 7.11 (d, *J* = 8.0 Hz, 2H), 5.69–5.67 (m, 1H), 5.31–5.29 (m, 1H), 4.20–4.14 (m, 1H), 3.40–3.33 (m, 1H), 2.61–2.58 (m, 2H), 2.43 (s, 3H), 2.32 (s, 3H). ^13^C{^1^H} NMR (125 MHz, CDCl_3_) *δ* 143.8, 137.9, 137.6, 135.0, 130.0, 129.5, 129.3, 127.3, 127.2, 121.0, 53.8, 41.5, 33.9, 21.8, 21.3. HRMS (ESI) calcd for C_19_H_21_ClNO_2_S (M + H)^+^ 362.0977, found 362.0953.

#### General procedure for the synthesis of (5a–5c)

A mixture of 4,4-dibromo-1-tosylpiperidine derivatives (0.22 mmol, 1 equiv.) in dichloromethane (DCM) (1.0 mL) and water (1.0 mL) was treated with acetic anhydride (Ac_2_O) (4.1 mmol, 19.0 equiv.) and triethylamine (Et_3_N) (5.7 mmol, 30.0 equiv.) at room temperature under an air atmosphere. The reaction mixture was vigorously stirred overnight at room temperature. After completion of the reaction, H_2_O was added, and the organic layer was extracted with DCM (3 × 10 mL). The combined organic layers were dried over anhydrous sodium sulfate (Na_2_SO_4_), filtered, and concentrated under reduced pressure by rotary evaporator and purified by column chromatography over silica gel using hexane/ethyl acetate (4 : 1, v/v) as eluent to get the products.

#### 2-(4-Bromophenyl)-1-tosylpiperidin-4-one (5a)

Brown solid; *R*_f_ (hexane/EtOAc, 4 : 1) 0.48; mp 113 °C, yield 72 mg, 80%; IR (KBr, neat) *ν* 2924, 1721, 1596, 1339, 1153, 1091, 1009, 930, 815, 709, 548 cm^−1^; ^1^H NMR (400 MHz, CDCl_3_) *δ* 7.81 (d, *J* = 8.2 Hz, 2H), 7.42 (d, *J* = 8.5 Hz, 2H), 7.35 (d, *J* = 8.1 Hz, 2H), 7.12 (d, *J* = 8.3 Hz, 2H), 5.56 (d, *J* = 7.0 Hz, 1H), 4.04–3.98 (m, 1H), 3.16–3.08 (m, 1H), 2.88–2.84 (m, 1H), 2.68 (dd, *J* = 15.3, 7.0 Hz, 1H), 2.45 (s, 3H), 2.42–2.38 (m, 1H), 2.26–2.21 (m, 1H). ^13^C{^1^H} NMR (100 MHz, CDCl_3_) *δ* 206.2, 144.5, 137.6, 137.4, 132.2, 130.4, 129.3, 127.3, 122.5, 56.2, 43.4, 40.6, 40.4, 21.8. HRMS (ESI) calcd for C_18_H_19_BrNO_3_S (M + H)^+^ 408.0264, found 408.0235.

#### 2-Propyl-1-tosylpiperidin-4-one (5b)^12^

Brown oil; *R*_f_ (hexane/EtOAc, 4 : 1) 0.47; yield 49 mg, 75%; ^1^H NMR (500 MHz, CDCl_3_) *δ* 7.77 (d, *J* = 7.8 Hz, 2H), 7.32 (d, *J* = 7.8 Hz, 2H), 4.42–4.37 (m, 1H), 4.15–4.10 (m, 1H), 3.30–3.23 (m, 1H), 2.55–2.51 (m, 1H), 2.43 (s, 3H), 2.41–2.36 (m, 1H), 2.22 (d, *J* = 14.3 Hz, 2H), 1.39–1.32 (m, 2H), 1.26–1.22 (m, 2H), 0.85 (q, *J* = 7.2, 3H). ^13^C{^1^H} NMR (125 MHz, CDCl_3_) *δ* 206.9, 144.1, 137.8, 130.2, 127.3, 54.6, 45.6, 40.5, 40.3, 34.6, 21.8, 19.3, 13.7.

#### 2-Hexyl-1-tosylpiperidin-4-one (5c)^12^

Colorless oil; *R*_f_ (hexane/EtOAc, 4 : 1) 0.46; yield 49 mg, 66%; ^1^H NMR (400 MHz, CDCl_3_) *δ* 7.77 (d, *J* = 8.3 Hz, 2H), 7.32 (d, *J* = 8.0 Hz, 2H), 4.39–4.33 (m, 1H), 4.18–4.12 (m, 1H), 3.29–3.22 (m, 1H), 2.54 (dd, *J* = 14.3, 6.5 Hz, 1H), 2.43 (s, 3H), 2.41–2.36 (m, 1H), 2.25–2.21 (m, 2H), 1.41–1.33 (m, 3H), 1.22–1.15 (m, 7H), 0.85 (t, *J* = 7.0 Hz, 3H). ^13^C{^1^H} NMR (100 MHz, CDCl_3_) *δ* 207.0, 144.0, 137.8, 130.2, 127.3, 54.9, 45.7, 40.6, 40.2, 32.4, 31.8, 28.9, 26.0, 22.7, 21.8, 14.3.

#### General procedure for the synthesis of (6a–6c)

A solution of 4,4-dibromo-1-tosylpiperidine derivatives (0.22 mmol, 1 equiv.) and 1,8-diazabicyclo[5.4.0]undec-7-ene (DBU) (10 mmol, 45.45 equiv.) was stirred in an oil bath at 80 °C for 12 hours. After completion of the reaction, it was allowed to cool to room temperature, brine was added, and the organic layer was extracted with EtOAc (3 × 10 mL). The combined organic layers were dried over anhydrous sodium sulfate (Na_2_SO_4_), filtered, and concentrated under reduced pressure by rotary evaporator and purified by column chromatography over silica gel using hexane/ethyl acetate (9 : 1, v/v) as eluent to get the products.

#### 2-(4-Bromophenyl)pyridine (6a)

Colorless gum; *R*_f_ (hexane/EtOAc, 9 : 1) 0.53; yield 38 mg, 74%; IR (KBr, neat) *ν* 3052, 3008, 2924, 1586, 1463, 1432, 1393, 1153, 1070, 1006, 839, 771 cm^−1^; ^1^H NMR (600 MHz, CDCl_3_) *δ* 8.72 (d, *J* = 4.8 Hz, 1H), 7.90 (d, *J* = 8.3 Hz, 2H), 7.81–7.78 (m, 1H), 7.73 (d, *J* = 8.0 Hz, 1H), 7.63 (d, *J* = 8.2 Hz, 2H), 7.30–7.28 (m, 1H). ^13^C{^1^H} NMR (150 MHz, CDCl_3_) *δ* 156.5, 149.9, 138.4, 137.2, 132.2, 128.7, 123.8, 122.7, 120.6. HRMS (ESI) calcd for C_11_H_9_BrN (M + H)^+^ 233.9913, found 233.9909.

#### 2-([1,1′-Biphenyl]-4-yl)pyridine (6b)^12^

Yellow solid; *R*_f_ (hexane/EtOAc, 9 : 1) 0.53; mp 133 °C, yield 33 mg, 64%; ^1^H NMR (400 MHz, CDCl_3_) *δ* 8.72 (dt, *J* = 4.8, 1.5 Hz, 1H), 8.10–8.06 (m, 2H), 7.79–7.77 (m, 2H), 7.73–7.71 (m, 2H), 7.67–7.65 (m, 2H), 7.49–7.45 (m, 2H), 7.39–7.35 (m, 1H), 7.26–7.24 (m, 1H). ^13^C{^1^H} NMR (100 MHz, CDCl_3_) *δ* 157.2, 149.8, 142.0, 140.8, 138.3, 137.2, 129.1, 127.8, 127.7, 127.6, 127.3, 122.4, 120.8.

#### 2-(*p*-Tolyl)pyridine (6c)^12^

Colorless oil; *R*_f_ (hexane/EtOAc, 9 : 1) 0.53; yield 26 mg, 70%; ^1^H NMR (500 MHz, CDCl_3_) *δ* 8.68 (t, *J* = 4.1 Hz, 1H), 7.90–7.88 (m, 2H), 7.74–7.70 (m, 2H), 7.30–7.26 (m, 2H), 7.22–7.20 (m, 1H), 2.41 (s, 3H). ^13^C {^1^H} NMR (125 MHz, CDCl_3_) *δ* 157.7, 149.7, 139.3, 137.0, 136.7, 129.7, 127.0, 122.1, 120.6, 21.5.

#### Experimental procedure for the synthesis of 7

A dried reaction flask was charged with 4-bromo-2-(4-chlorophenyl)-1-tosyl-1,2,3,6-tetrahydropyridine 4ac (100 mg, 0.23 mmol, 1.0 equiv.). Triphenylphosphine (PPh_3_) (0.02 mmol, 0.05 equiv.), palladium(ii) chloride (PdCl_2_) (0.02 mmol, 0.05 equiv.), and copper(i) iodide (CuI) (0.01 mmol, 0.01 equiv.) were then added, and the flask was evacuated under reduced pressure and subsequently purged with nitrogen gas. Subsequently, anhydrous dimethylformamide (DMF) (3 mL), triethylamine (Et_3_N) (4.14 mmol, 18.0 equiv.), and phenylacetylene (0.37 mmol, 1.6 equiv.) were added to the reaction flask under a nitrogen atmosphere. The reaction mixture was heated at 80 °C in an oil bath for 12 h. After completion of the reaction, it was allowed to cool to room temperature and then quenched with a saturated sodium bicarbonate solution. A brine solution was then added, and the mixture was extracted with ethyl acetate (3 × 10 mL). The combined organic layer was dried over anhydrous sodium sulfate (Na_2_SO_4_), filtered, and concentrated under reduced pressure by rotary evaporator. The crude was purified by silica gel column chromatography with ethyl acetate/hexane (1 : 9, v/v) as the eluent to yield the product.

#### 2-(4-Chlorophenyl)-4-(phenylethynyl)-1-tosyl-1,2,3,6-tetra-hydropyridine (7)

Pale yellow gum; *R*_f_ (hexane/EtOAc, 9 : 1) 0.32; yield 88 mg, 82%; IR (KBr, neat) *ν* 2921, 2851, 2278, 1659, 1597, 1493, 1441, 1344, 1160, 1093, 1012, 911, 812, 733, 491 cm^−1^; ^1^H NMR (400 MHz, CDCl_3_) *δ* 7.63–7.61 (m, 2H), 7.35–7.32 (m, 2H), 7.24–7.19 (m, 9H), 5.92–5.90 (m, 1H), 5.24 (dd, *J* = 5.8, 2.0 Hz, 1H), 4.20–4.13 (m, 1H), 3.43–3.36 (m, 1H), 2.54–2.46 (m, 2H), 2.34 (s, 3H). ^13^C{^1^H} NMR (150 MHz, CDCl_3_) *δ* 143.8, 137.5, 137.1, 133.8, 131.7, 130.0, 129.5, 129.0, 128.9, 128.7, 128.6, 127.1, 122.9, 118.2, 89.6, 88.8, 52.4, 41.4, 30.5, 21.8. HRMS (ESI) calcd for C_26_H_22_ClNNaO_2_S (M + Na)^+^ 470.0952, found 470.0925.

#### Experimental procedure for the gram-scale reaction

A solution of *N*-(3-bromobut-3-en-1-yl)-4-methylbenzenesulfonamide (1.0 g, 3.30 mmol, 1.0 equiv.) and 4-bromobenzaldehyde (0.67 g, 3.63 mmol, 1.1 equiv.) in anhydrous 1,2-dichloroethane (DCE) (15 mL) was cooled to 0 °C under a nitrogen atmosphere. To this solution boron tribromide (BBr_3_) (1 M in DCM) (0.21 g, 0.83 mmol, 0.25 equiv.) and trimethylsilyl bromide (TMSBr) (0.61 g, 3.96 mmol, 1.2 equiv.) were added. The reaction mixture was stirred at room temperature overnight, and the progress was monitored by thin-layer chromatography (TLC) using ethyl acetate/hexane (1 : 9, v/v) as the eluent. After completion of the reaction, it was quenched with a saturated sodium bicarbonate solution. A brine solution was then added, and the mixture was extracted with ethyl acetate (3 × 10 mL). The combined organic layers were dried over anhydrous sodium sulfate (Na_2_SO_4_), filtered, and concentrated under reduced pressure by rotary evaporator. The desired product 3ad was obtained (1.11 g, colorless solid) in 61% yield by silica gel column chromatography using hexane/ethyl acetate (9 : 1, v/v) as the eluent.

## Conflicts of interest

There are no conflicts to declare.

## Supplementary Material

RA-015-D5RA03630E-s001

RA-015-D5RA03630E-s002

RA-015-D5RA03630E-s003

## Data Availability

The data supporting the findings of this study are available within the article and/or its ESI.† Supporting data for this article are provided in the ESI,† which includes copies of the ^1^H NMR and ^13^C{^1^H} NMR spectra for all newly synthesized compounds, along with single-crystal X-ray data for compounds 3ac (CCDC 2429152), 3dj (CCDC 2431627), and 4aa (CCDC 2429151).
