# Supplementary material for: Selective synthesis of gem-dihalopiperidines and 4-halo-1,2,3,6-tetrahydropyridines from halogen substituted homoallylic benzenesulfonamides and aldehydes
Source: RSC Adv. 2025 Jun 23;15(27):21257–68. doi: 10.1039/d5ra03630e (PMC12184090; doi:10.1039/d5ra03630e)
Supplement: RA-015-D5RA03630E-s001 [file RA-015-D5RA03630E-s001.pdf]

Supplementary Information (SI) for RSC Advances.
This journal is © The Royal Society of Chemistry 2025

Supporting Information for Publication

Selective Synthesis of gem-Dihalopiperidines and 4-Halo-1,2,3,6-Tetrahydropyridines from Halogen Substituted Homoallylic Benzenesulfonamides and Aldehydes

Surjya Kumar Bora and Anil K. Saikia*

Department of Chemistry, Indian Institute of Technology Guwahati, Guwahati 781039, India

E-mail: asaikia@iitg.ac.in

Table of Contents:

1. **^{1}H, $^{13}C\{^{1}H\}$ and ^{19}F spectra of all new compounds** **S2-S43**
2. **X-ray crystallographic data of compound 3ac, 3cj and 4aa** **S44-S50**

^{1}H (400 MHz, $CDCl_3$) and ^{13}C{^{1}H} (150 MHz, $CDCl_3$) spectra of 3aa:

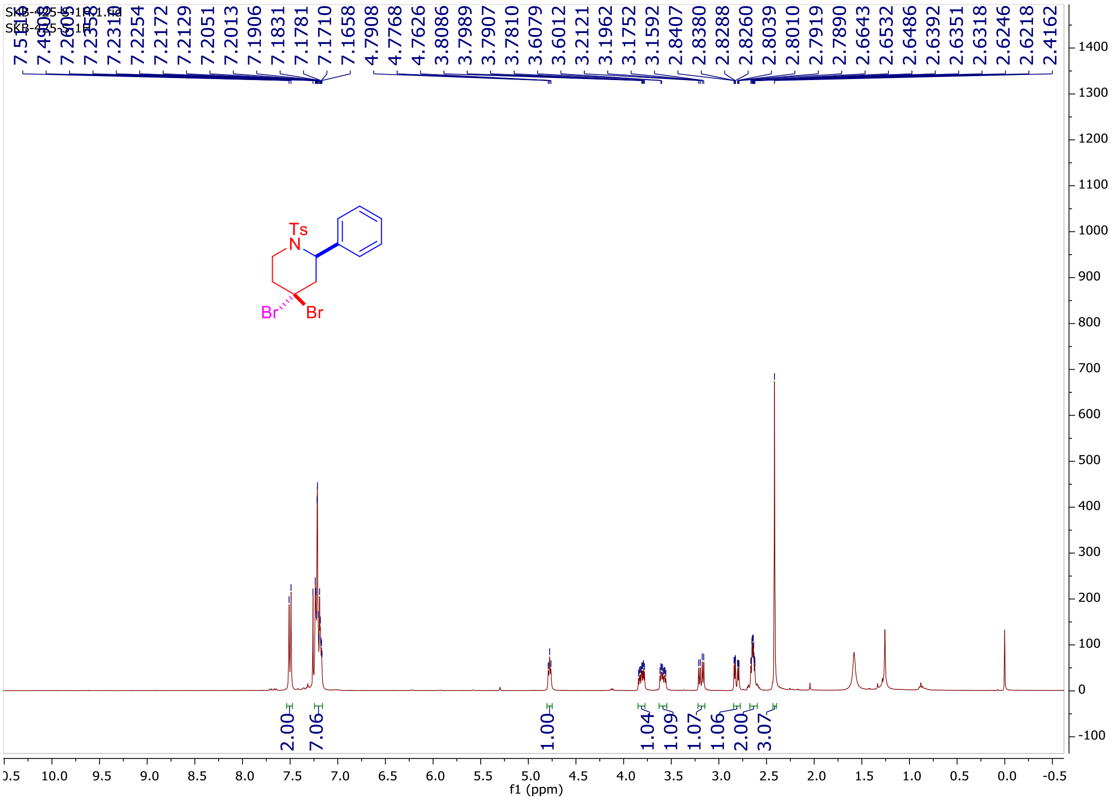

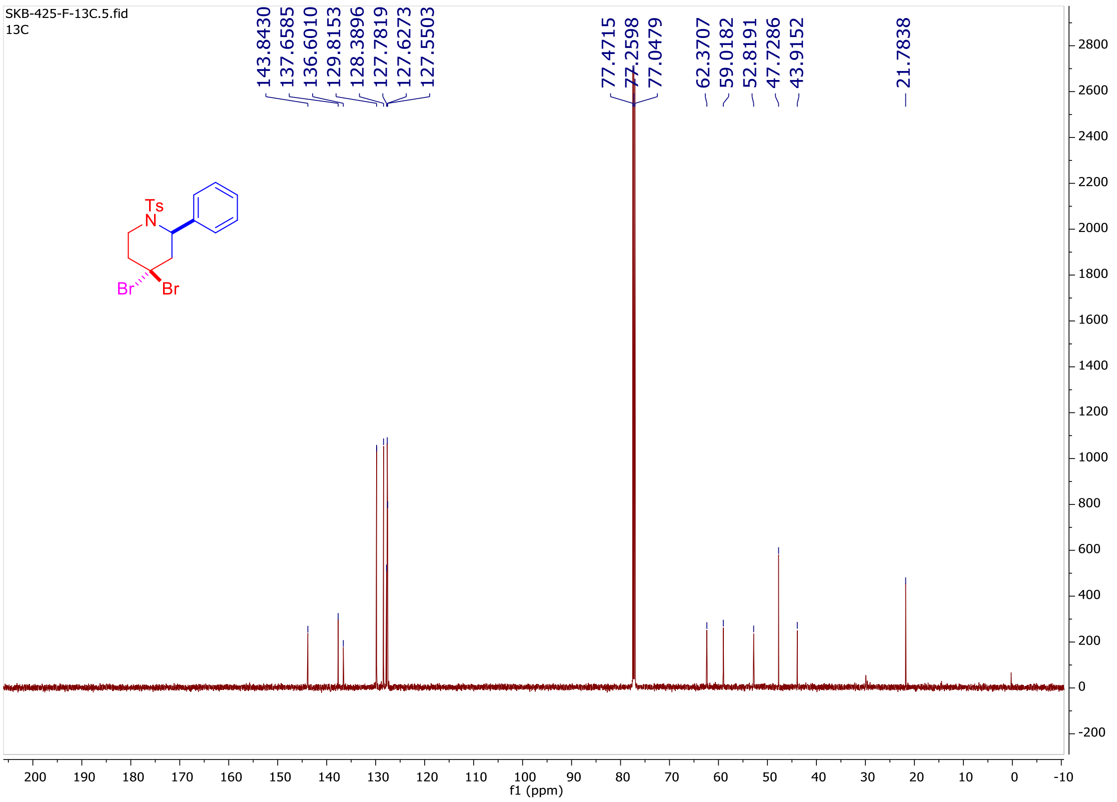

^{1}H (400 MHz, $CDCl_3$) and $^{13}C\{^1H\}$ (125 MHz, $CDCl_3$) spectra of 3ab:

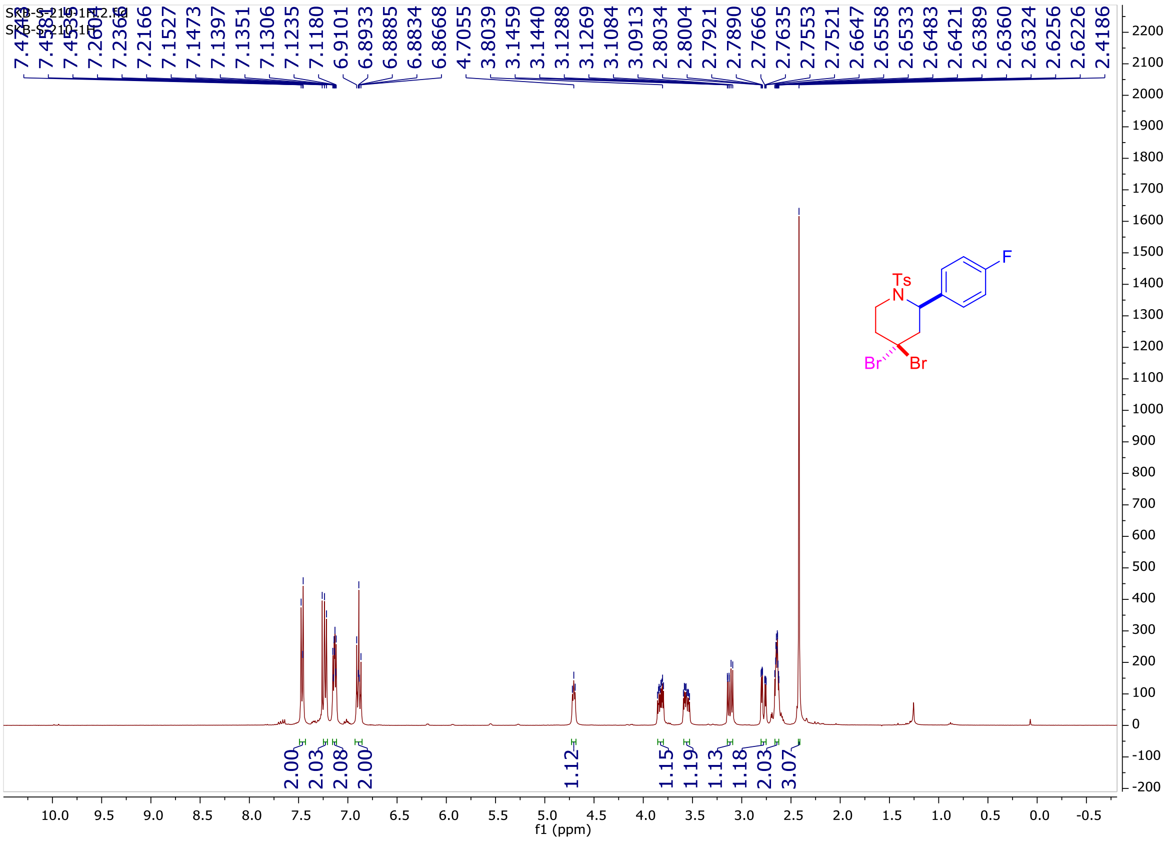

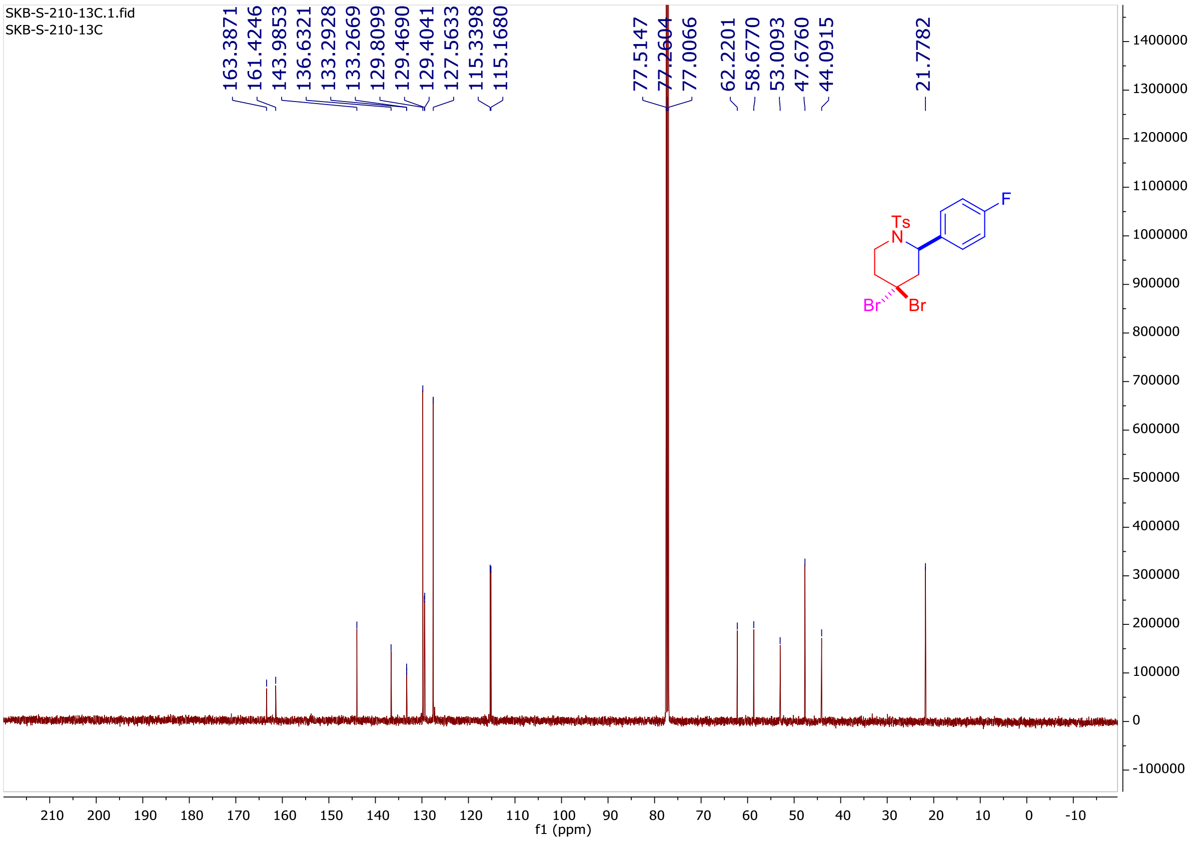

^{19}F (470 MHz, $C_6F_6/CDCl_3$) spectrum of 3ab:

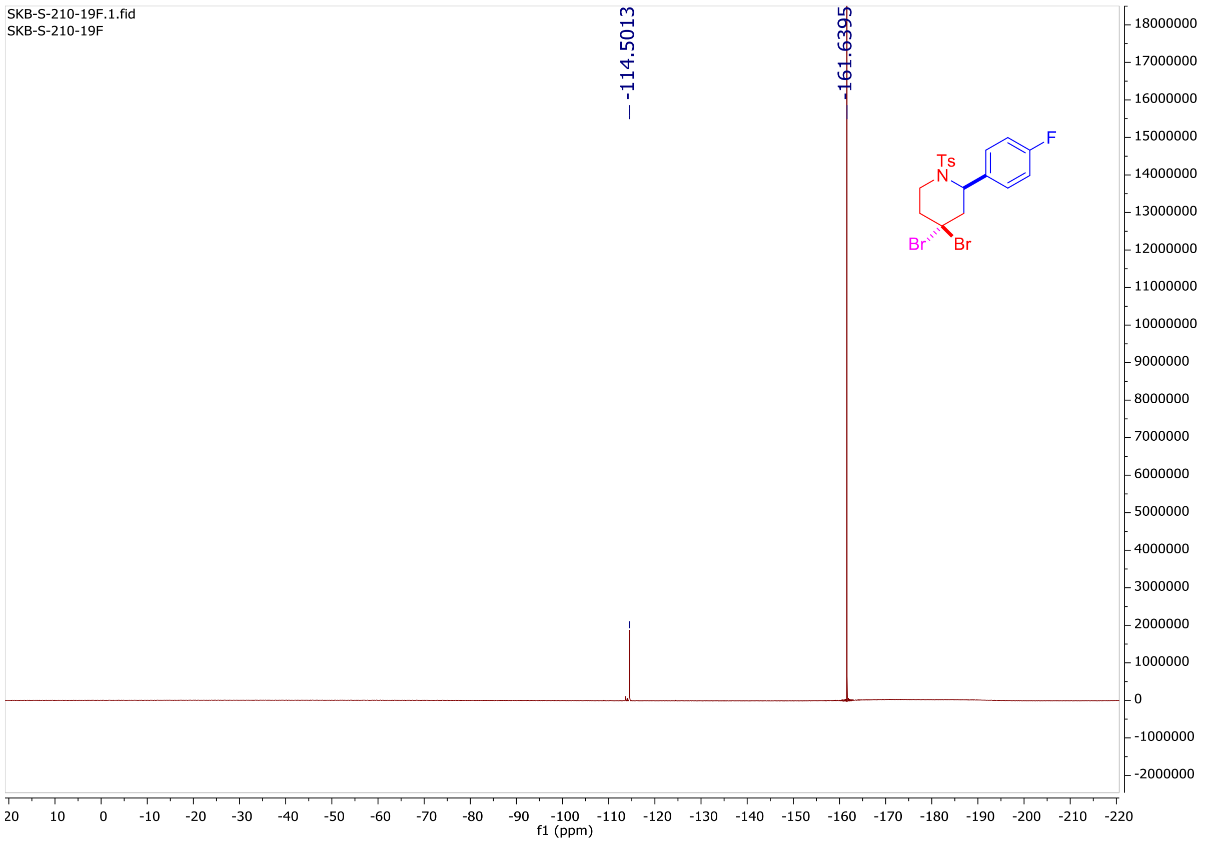

^{1}H (500 MHz, $CDCl_3$) and $^{13}C\{^{1}H\}$ (125 MHz, $CDCl_3$) spectra of 3ac:

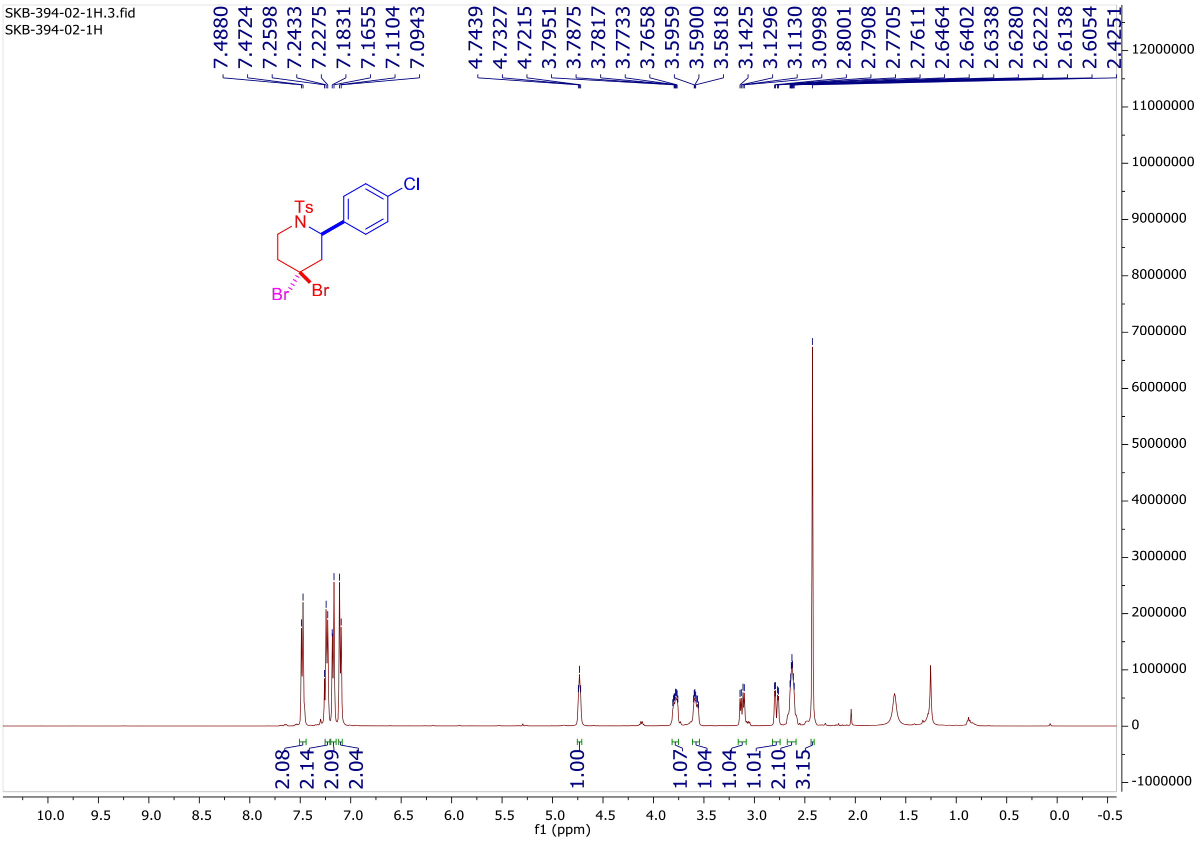

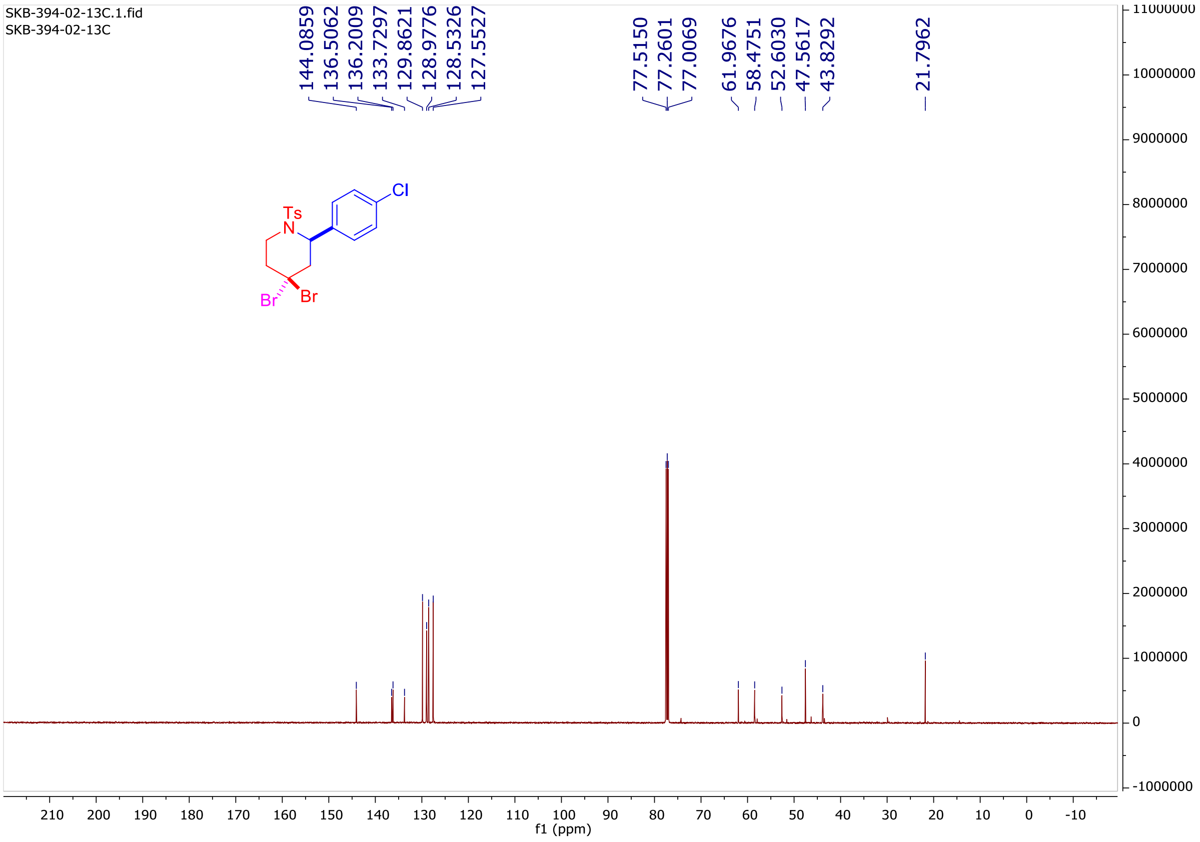

^{1}H (400 MHz, $CDCl_3$) and $^{13}C\{^{1}H\}$ (150 MHz, $CDCl_3$) spectra of 3ad:

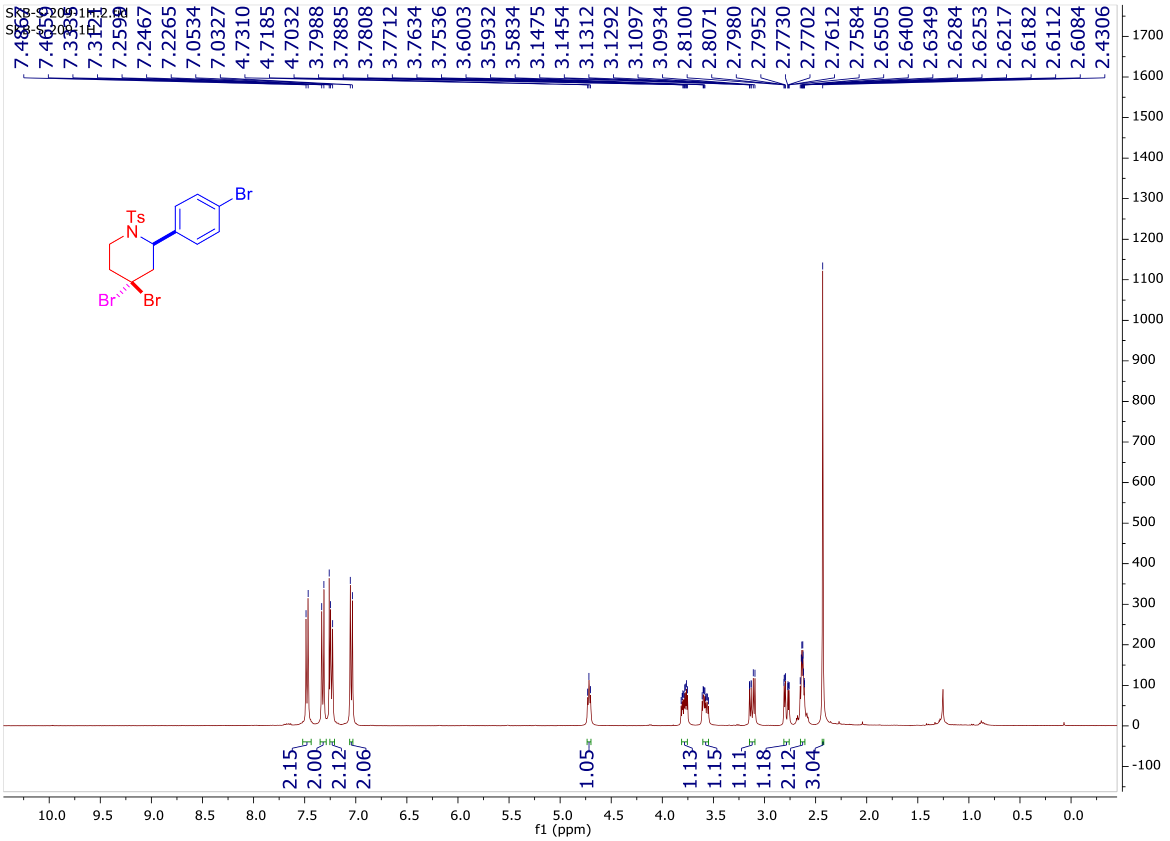

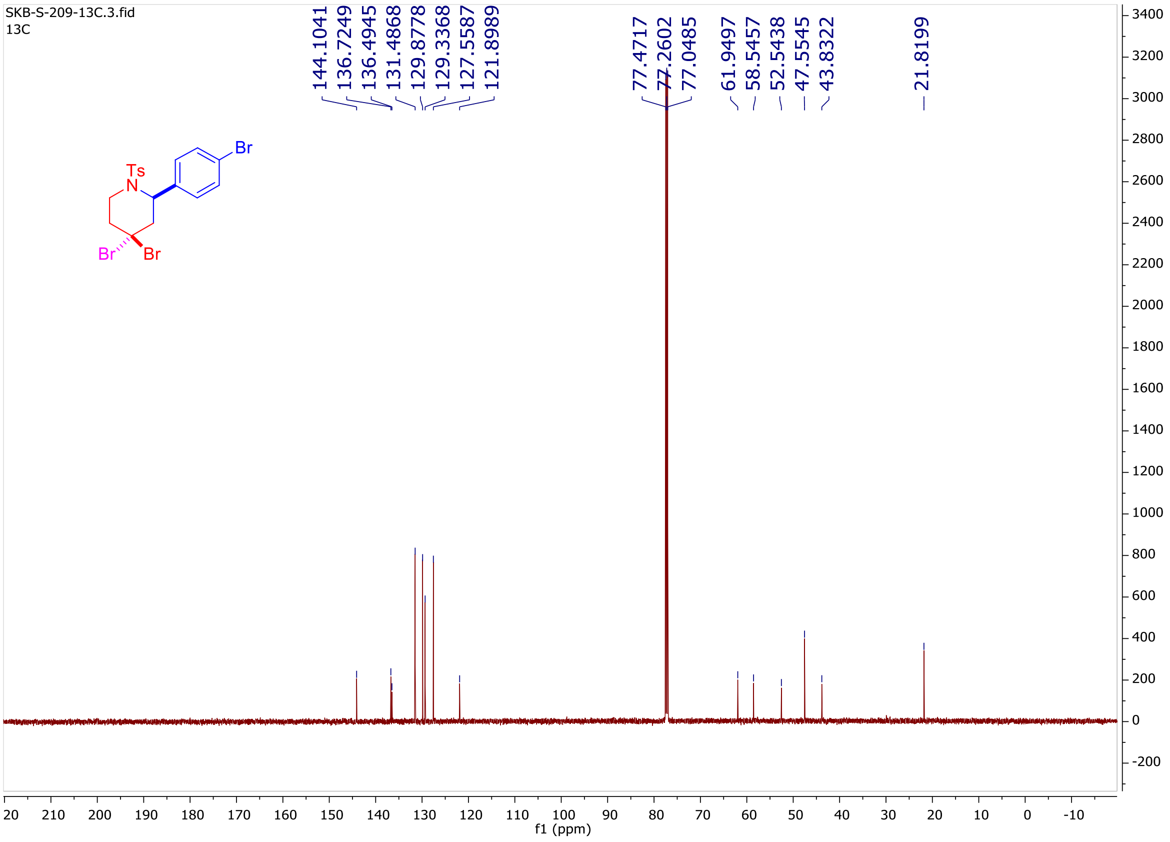

^{1}H (600 MHz, $CDCl_3$) and $^{13}C\{^{1}H\}$ (150 MHz, $CDCl_3$) spectra of 3ae:

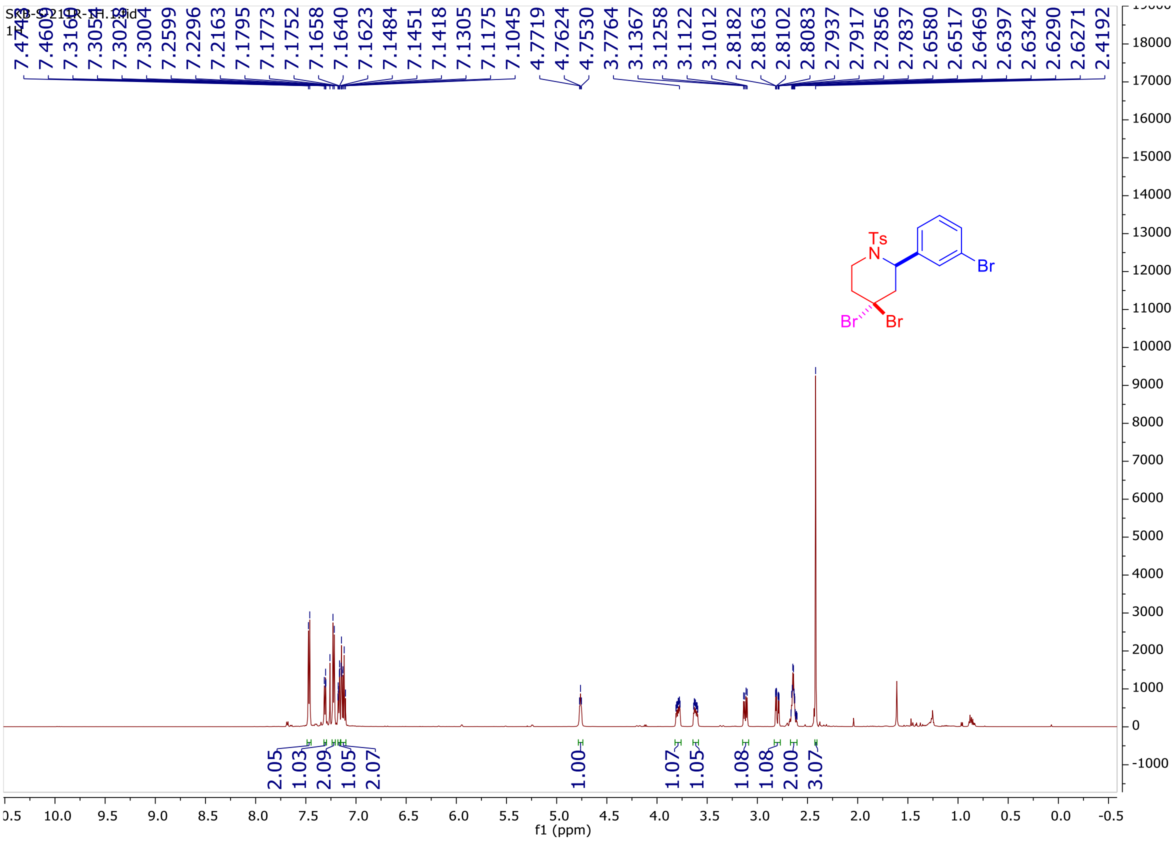

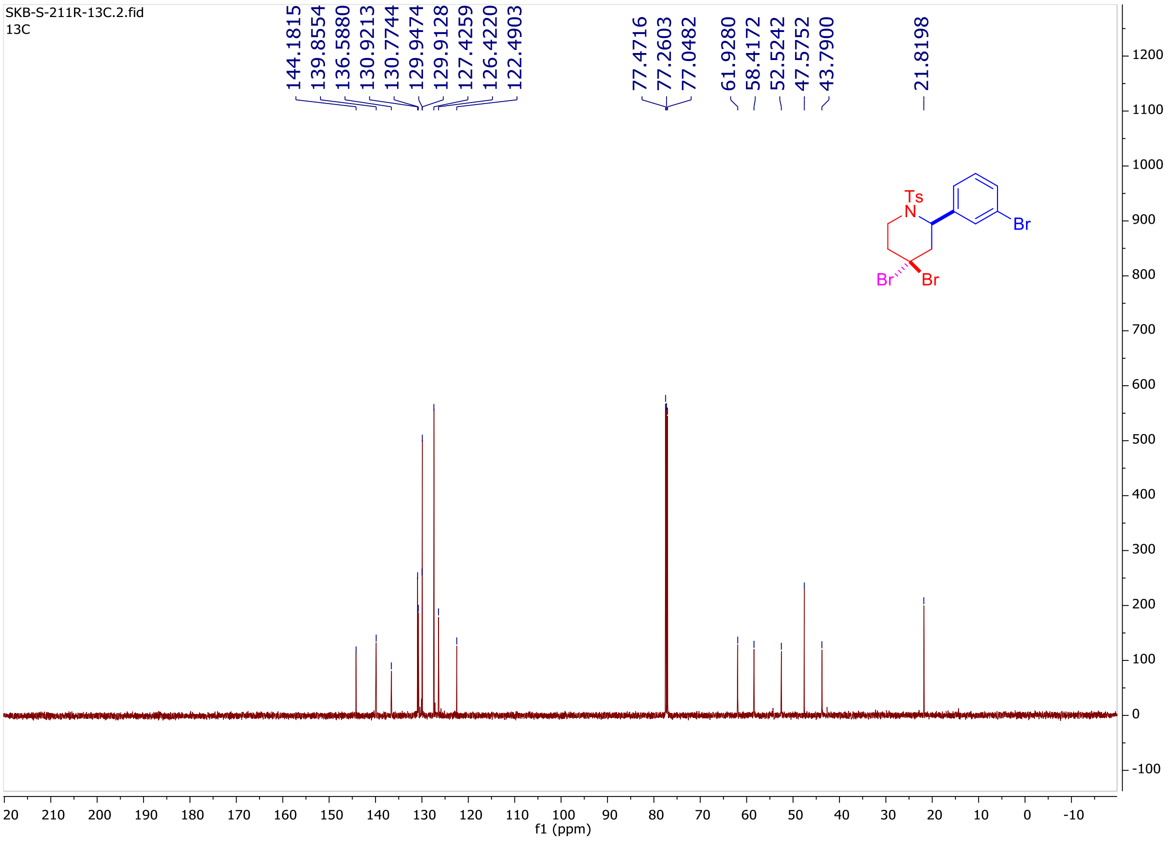

^1H (400 MHz, $CDCl_3$) and $^{13}C\{^1H\}$ (150 MHz, $CDCl_3$) spectra of 3af:

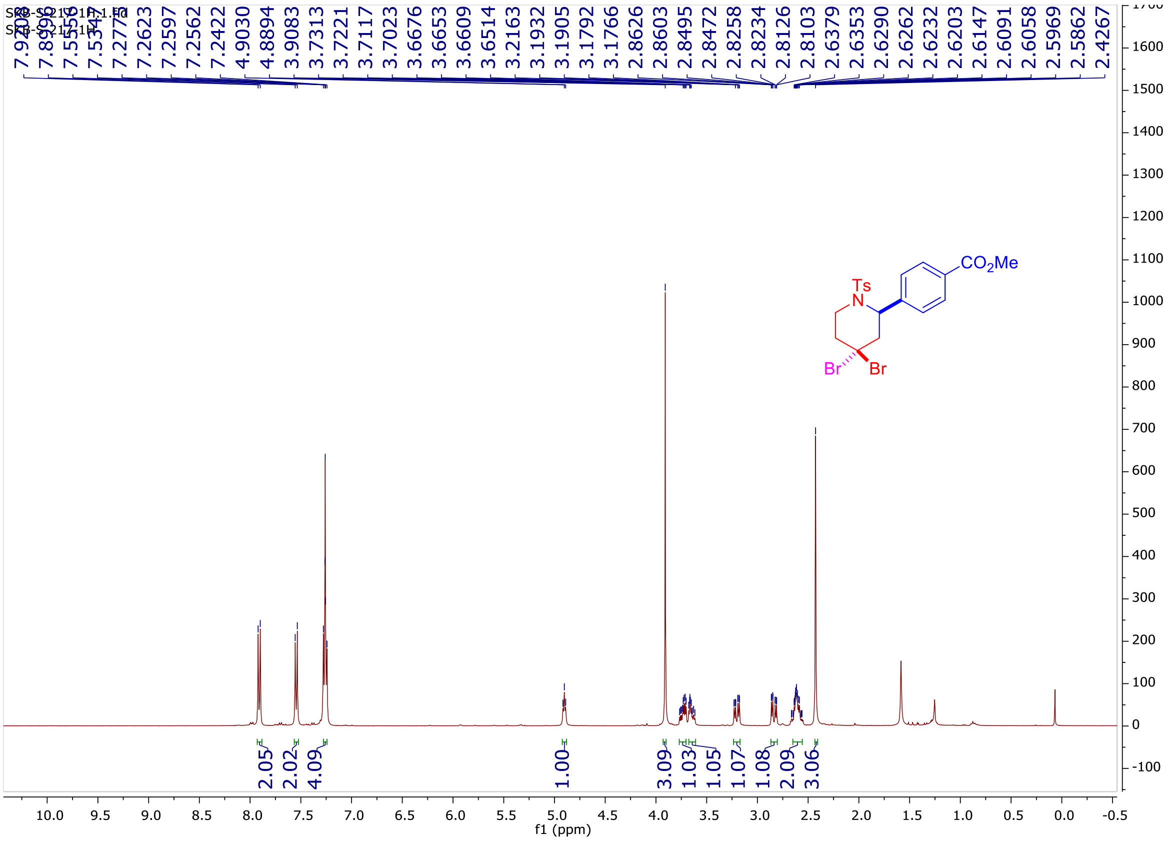

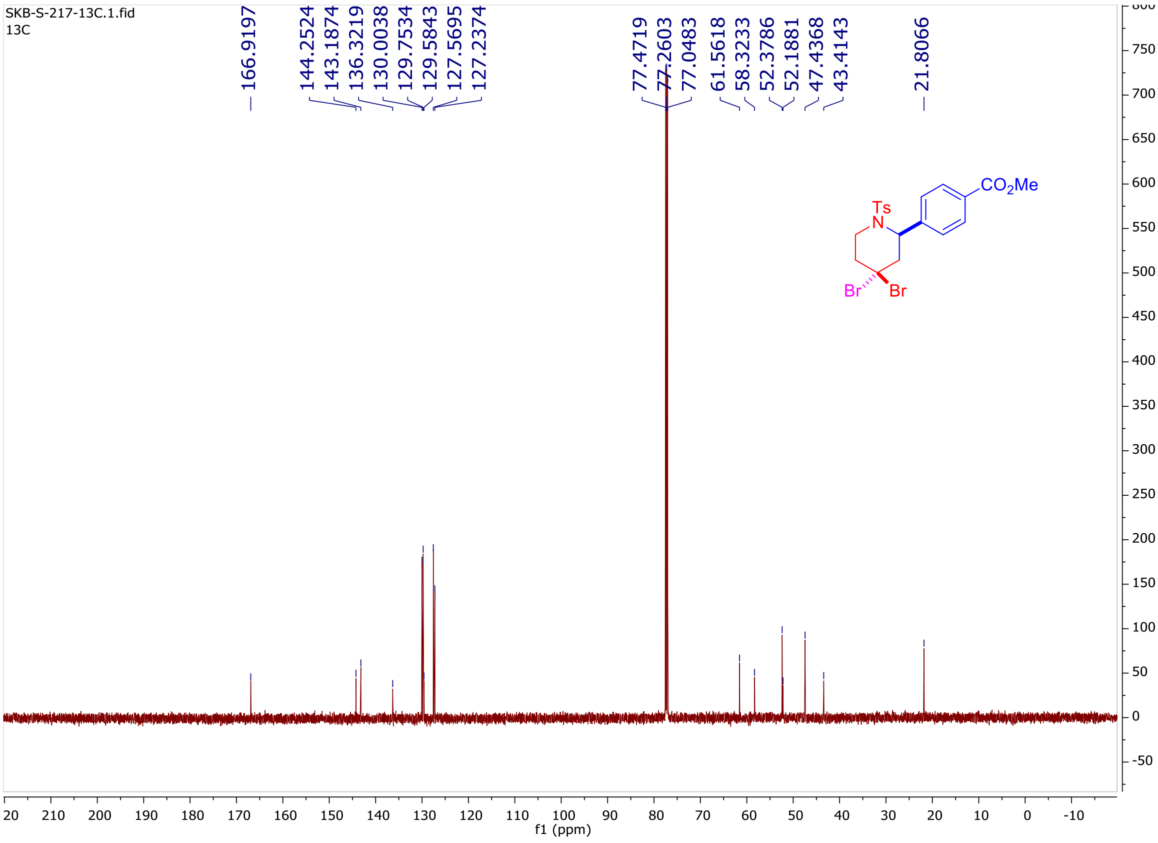

^{1}H (600 MHz, $CDCl_3$) and $^{13}C\{^{1}H\}$ (125 MHz, $CDCl_3$) spectra of 3ag:

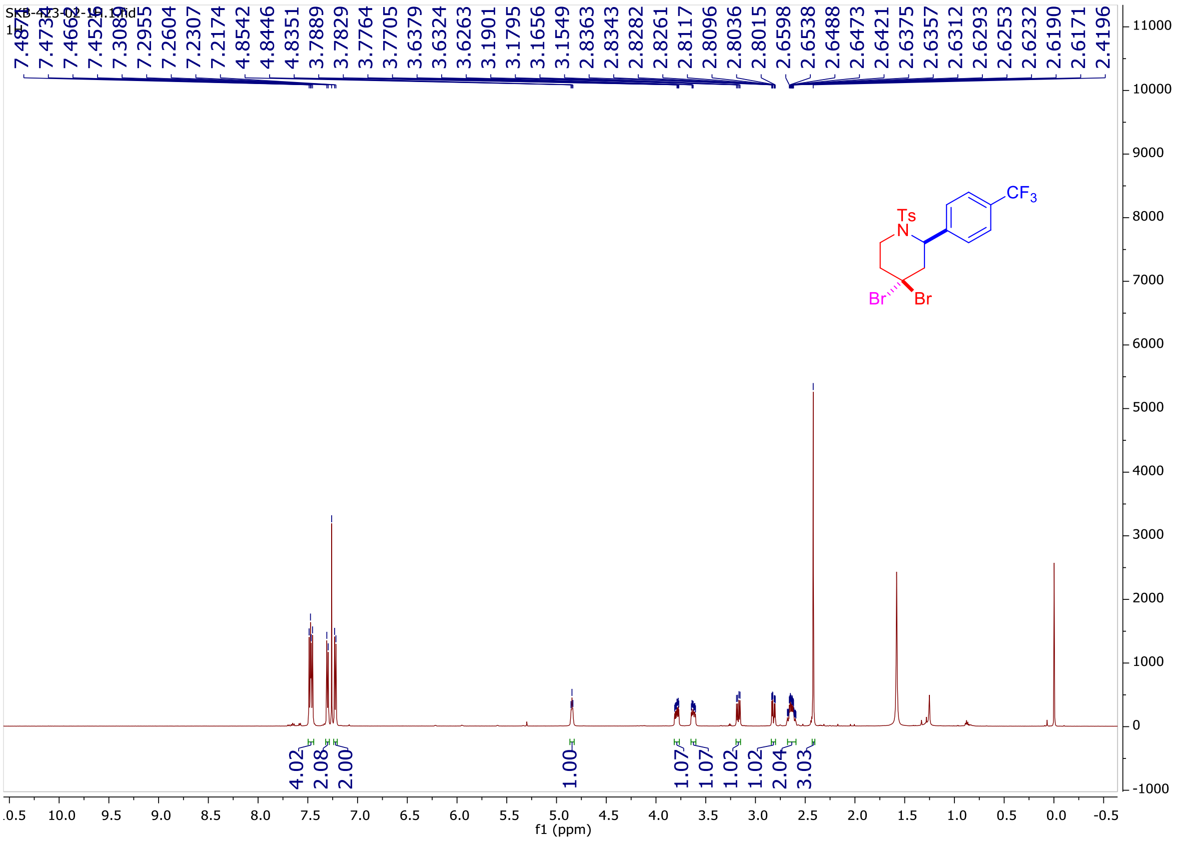

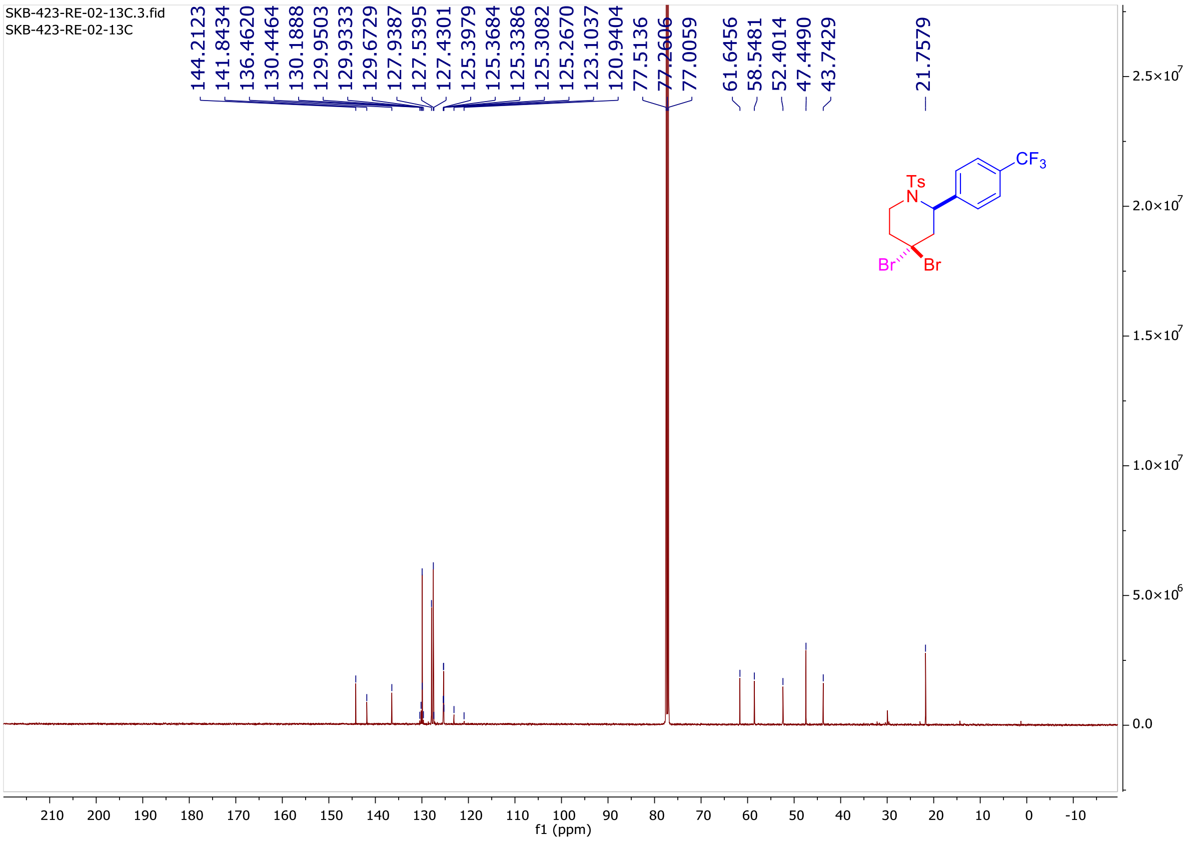

^{19}F (470 MHz, $C_6F_6/CDCl_3$) spectrum of 3ag:

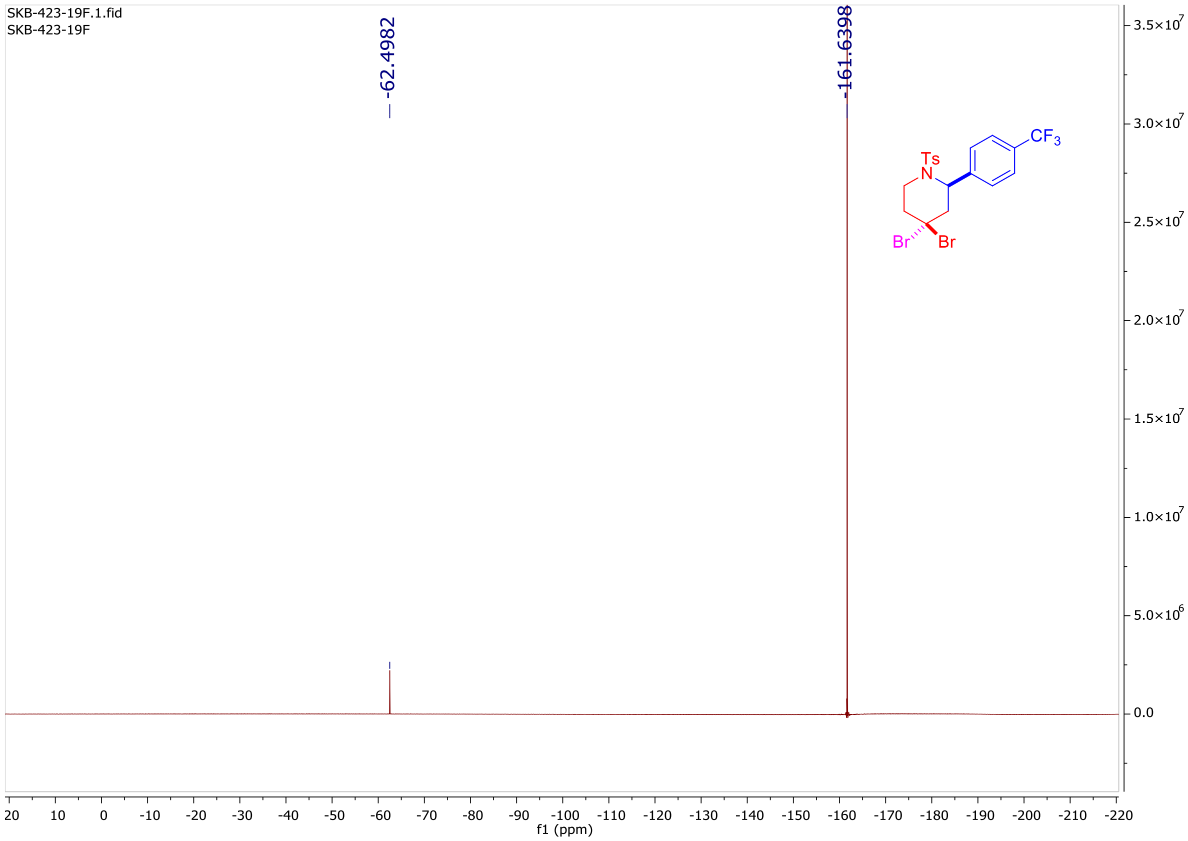

^{1}H (500 MHz, CDCl3) and ^{13}C{^{1}H} (125 MHz, CDCl3) spectra of 3ah:

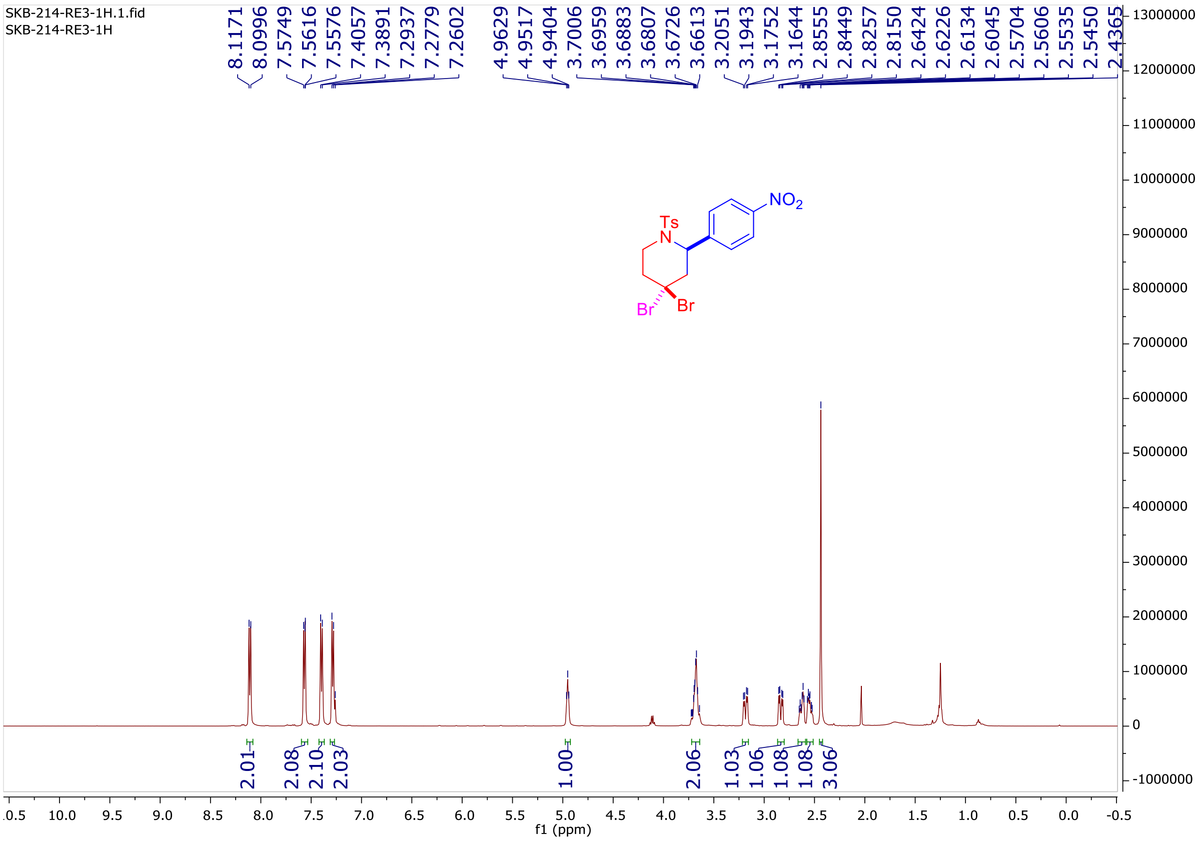

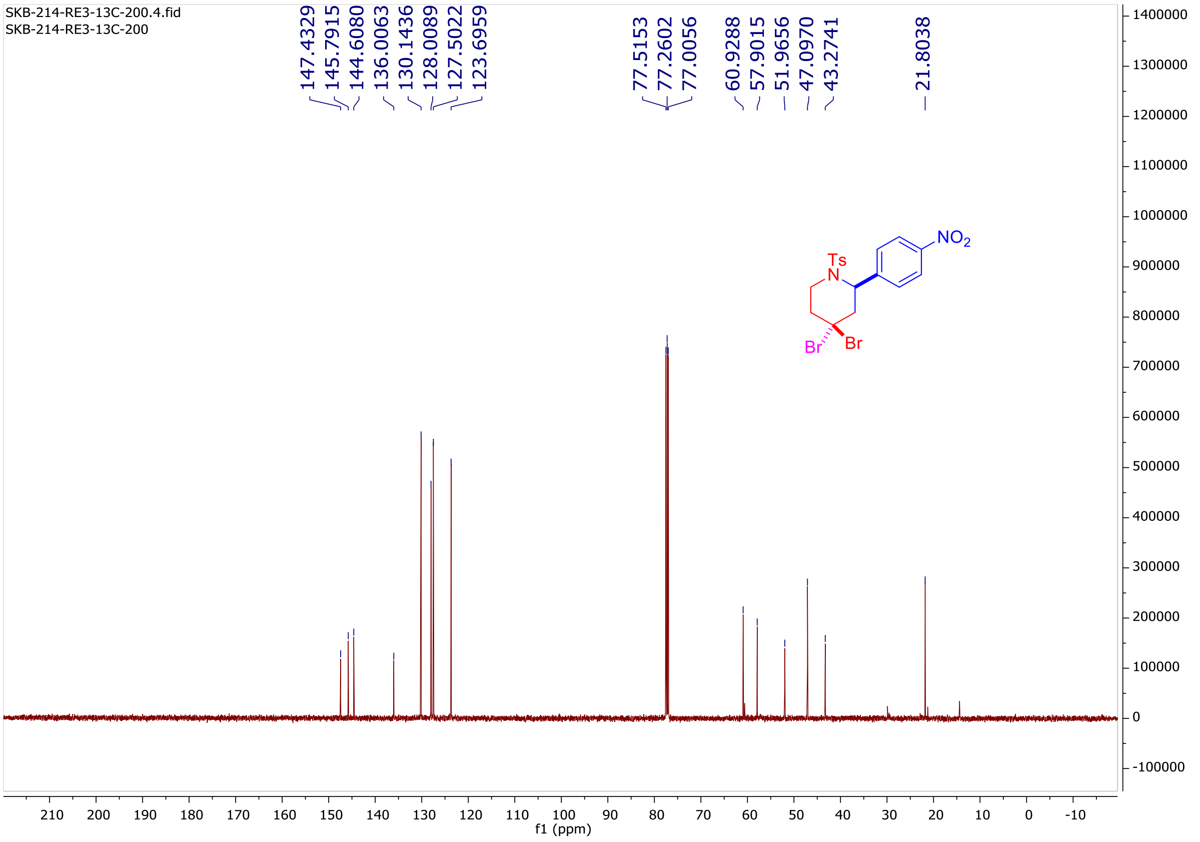

^{1}H (400 MHz, $CDCl_3$) and $^{13}C\{^{1}H\}$ (125 MHz, $CDCl_3$) spectra of 3ai:

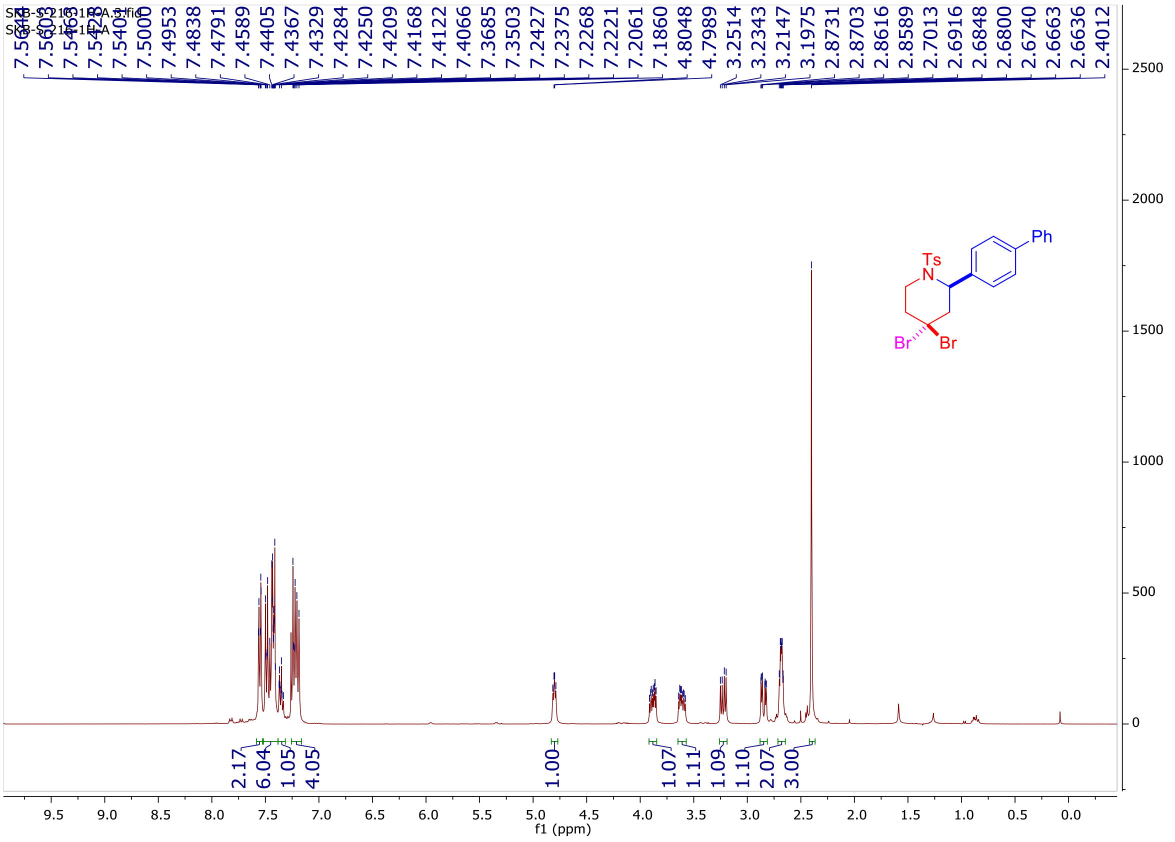

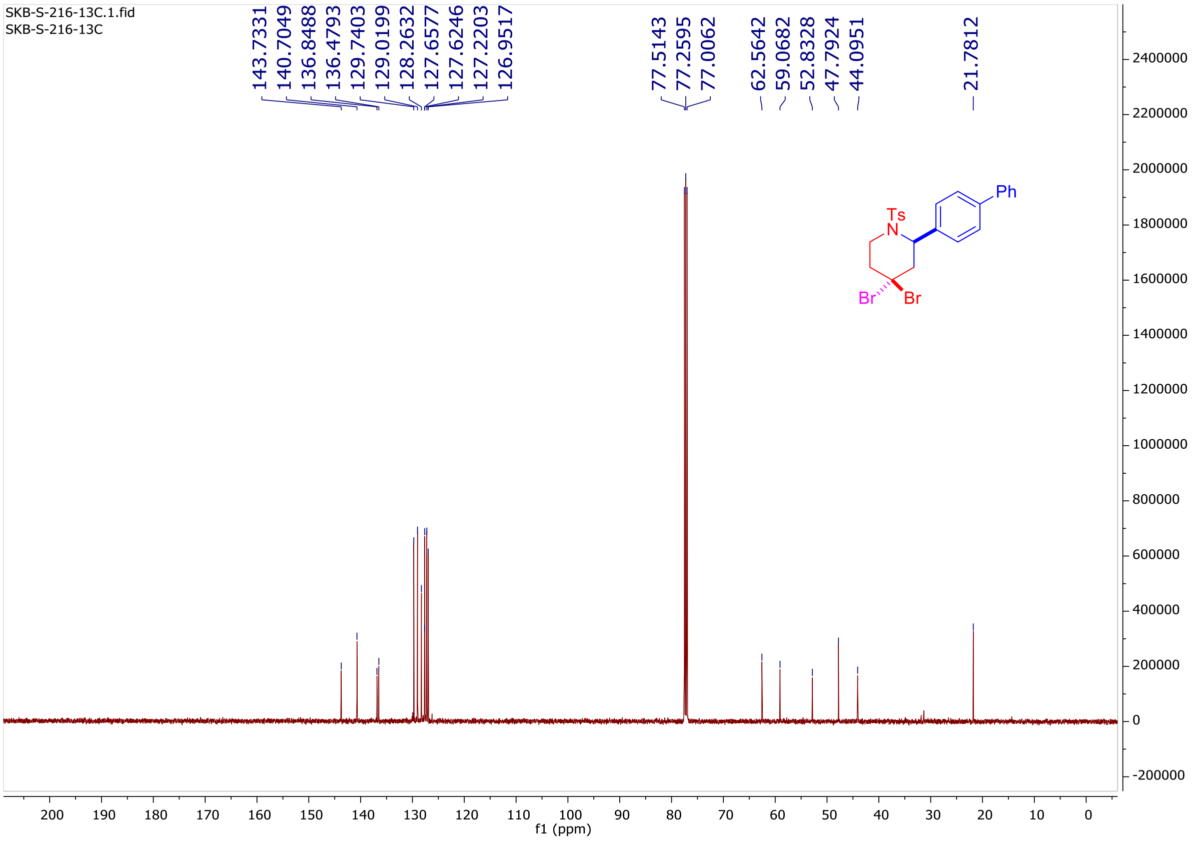

^{1}H (600 MHz, $CDCl_3$) and $^{13}C\{^{1}H\}$ (150 MHz, $CDCl_3$) spectra of 3aj:

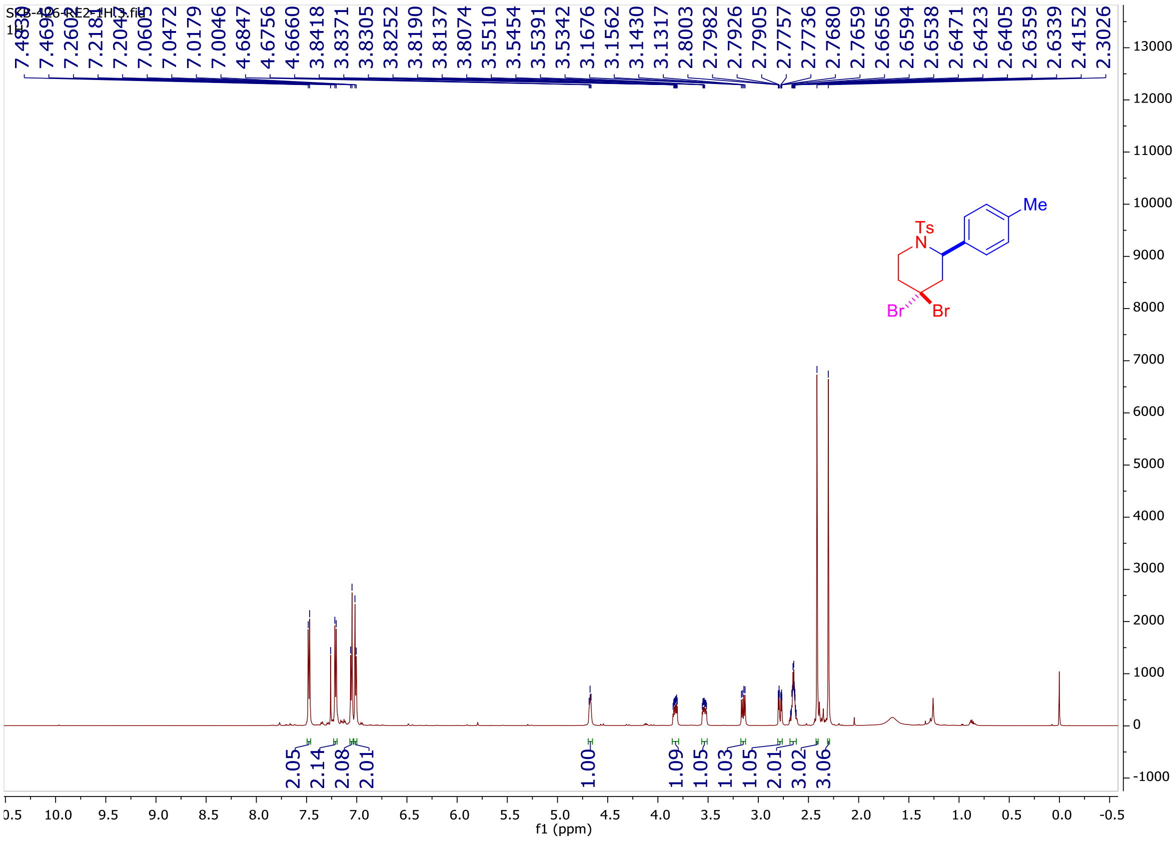

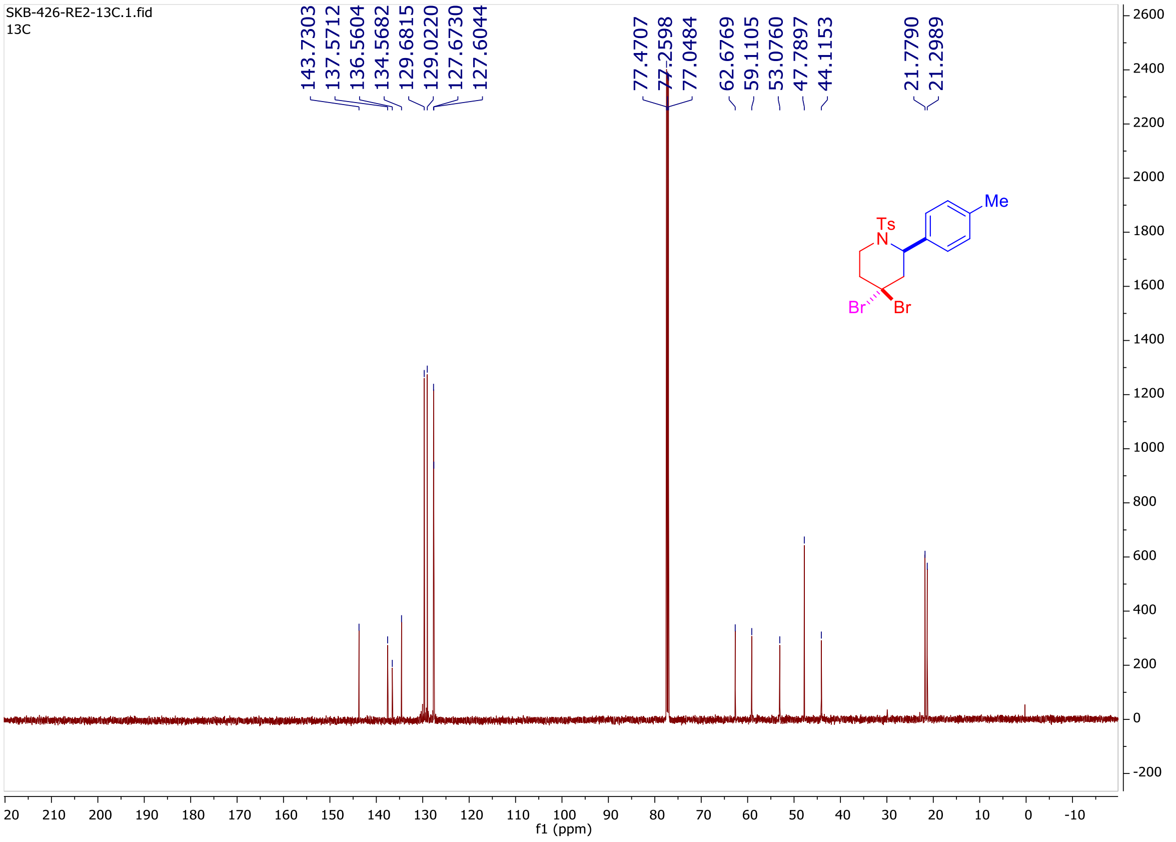

^{1}H (400 MHz, $CDCl_3$) and ^{13}C{^{1}H} (125 MHz, $CDCl_3$) spectra of 3al:

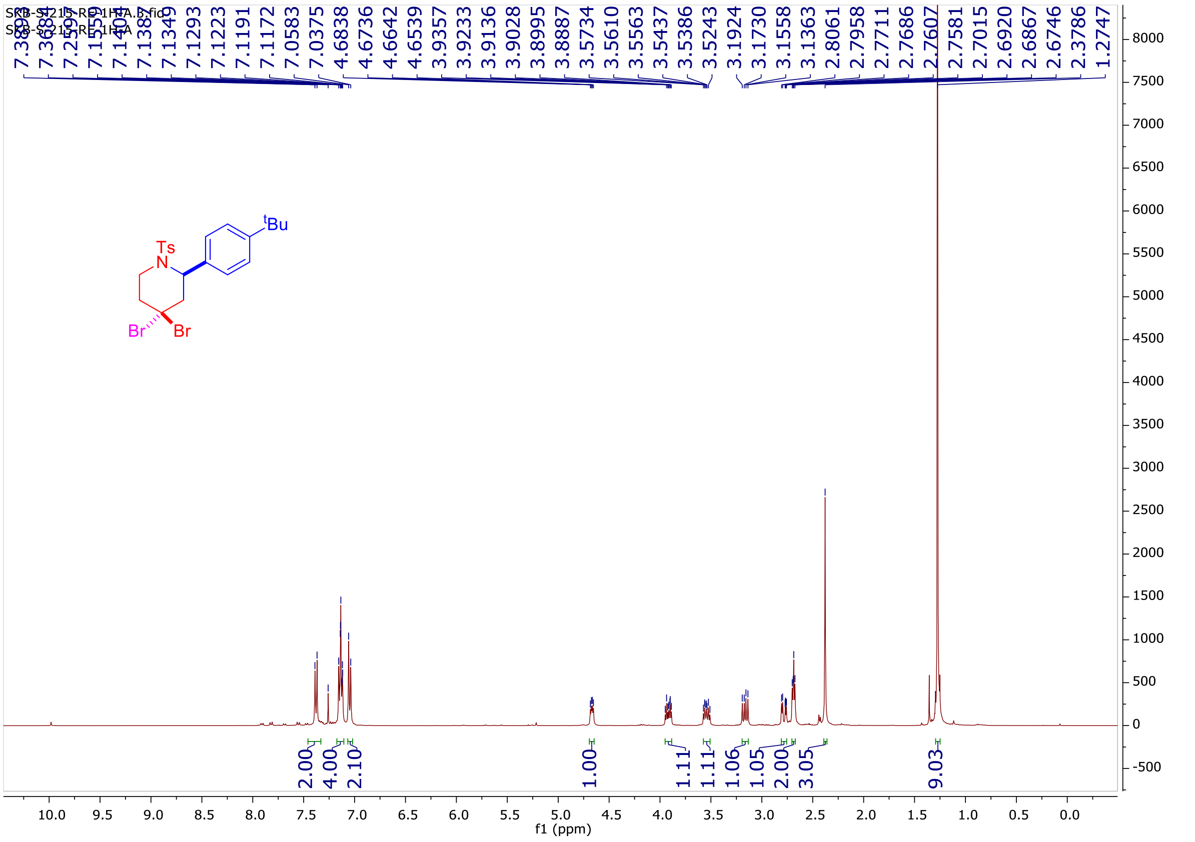

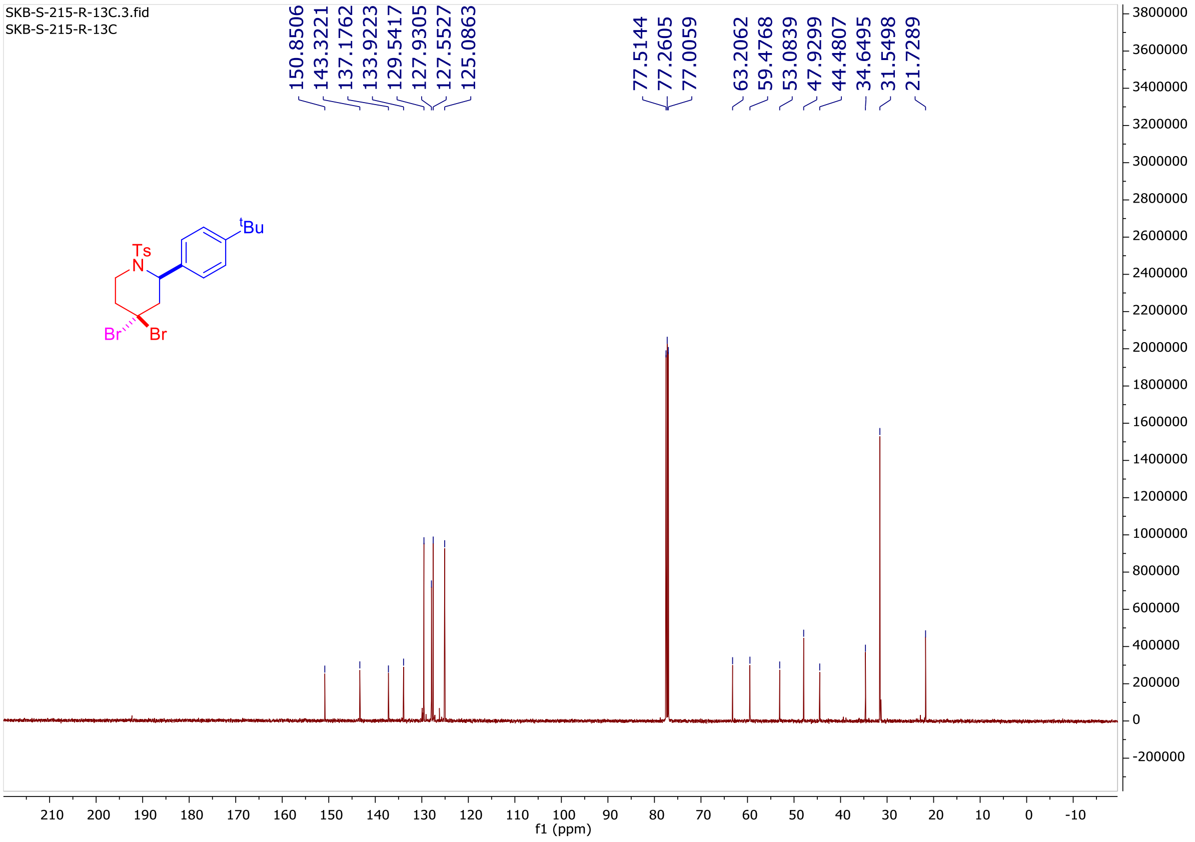

^{1}H (400 MHz, CDCl$_3$) and ^{13}C{^{1}H} (125 MHz, CDCl$_3$) spectra of 3am:

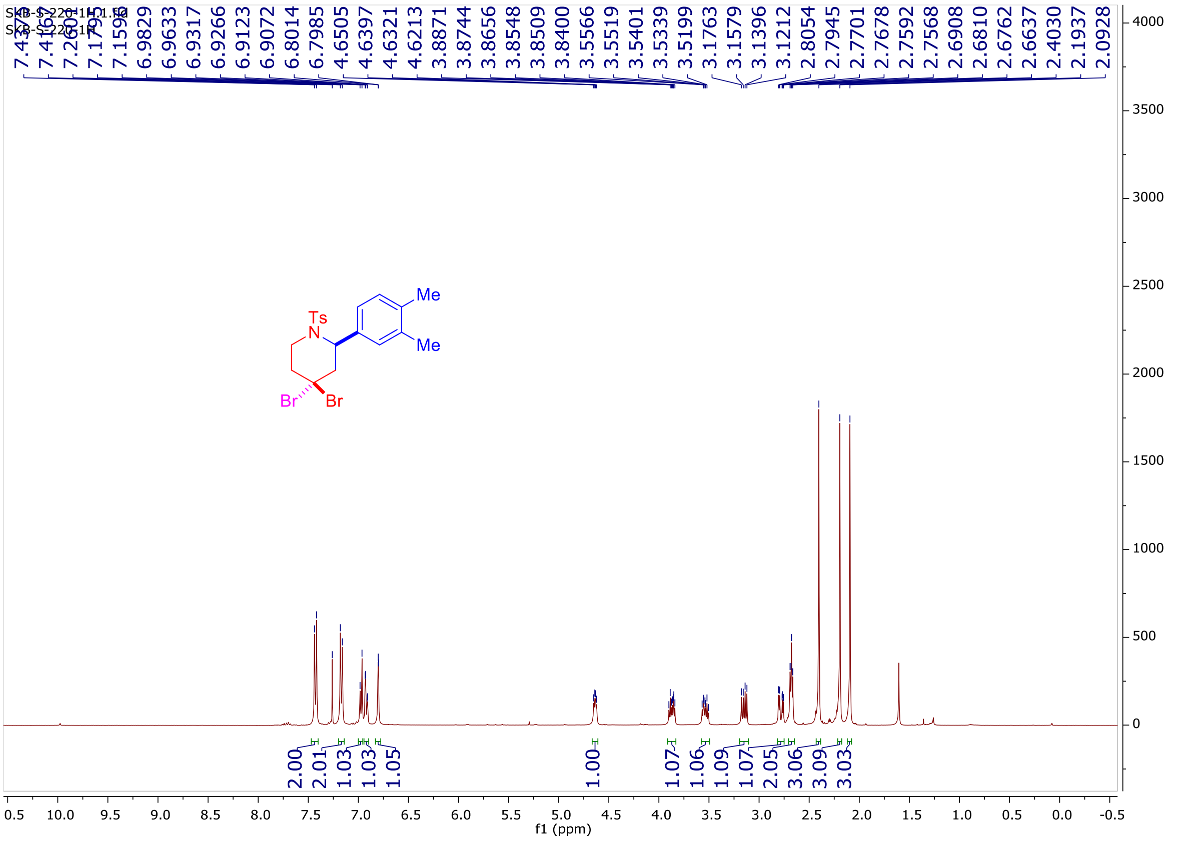

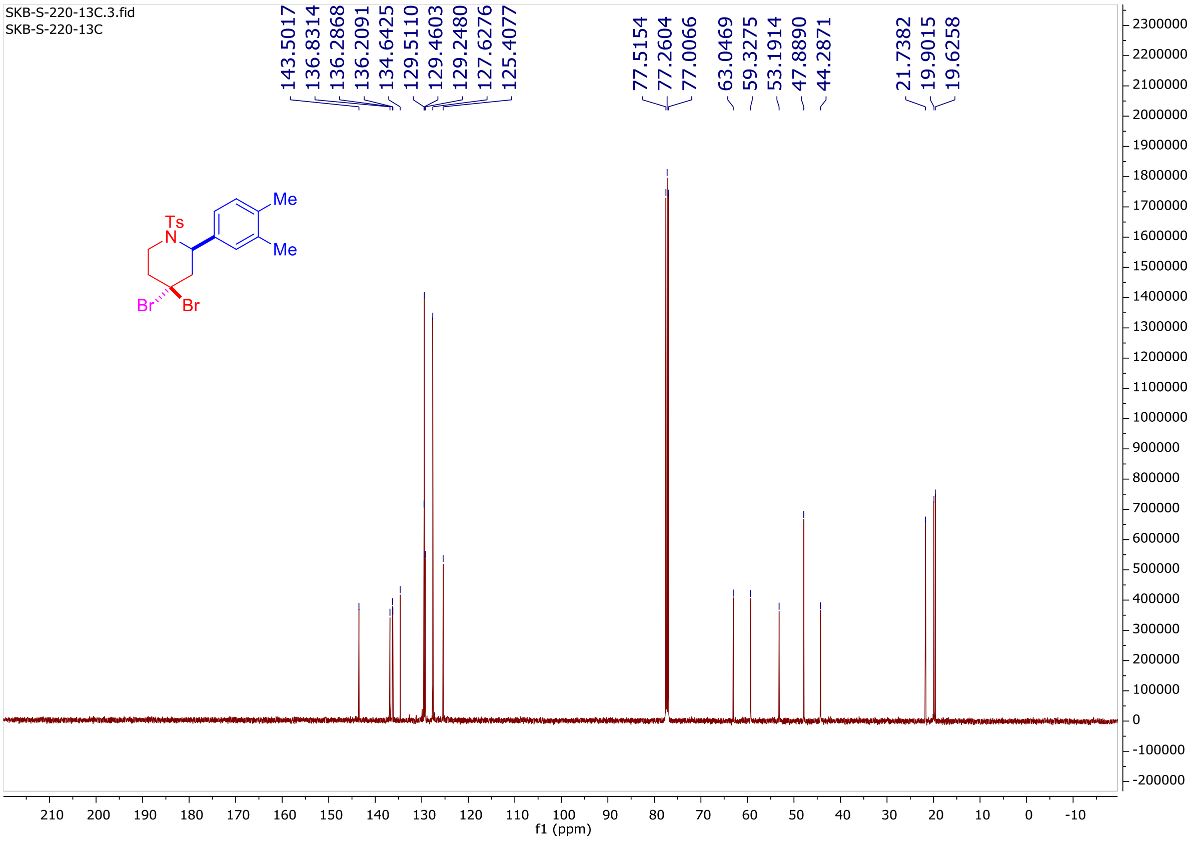

^{1}H (400 MHz, $CDCl_3$) and $^{13}C\{^{1}H\}$ (125 MHz, $CDCl_3$) spectra of 3an:

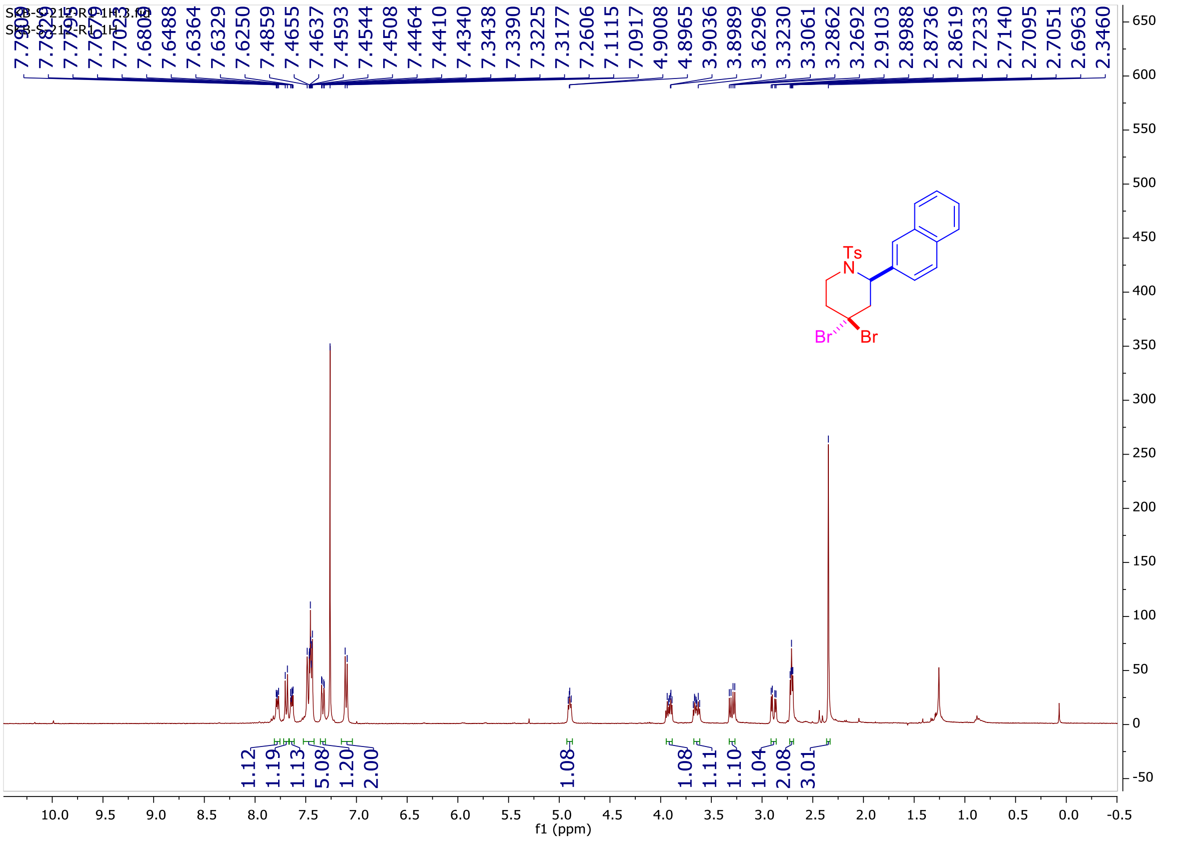

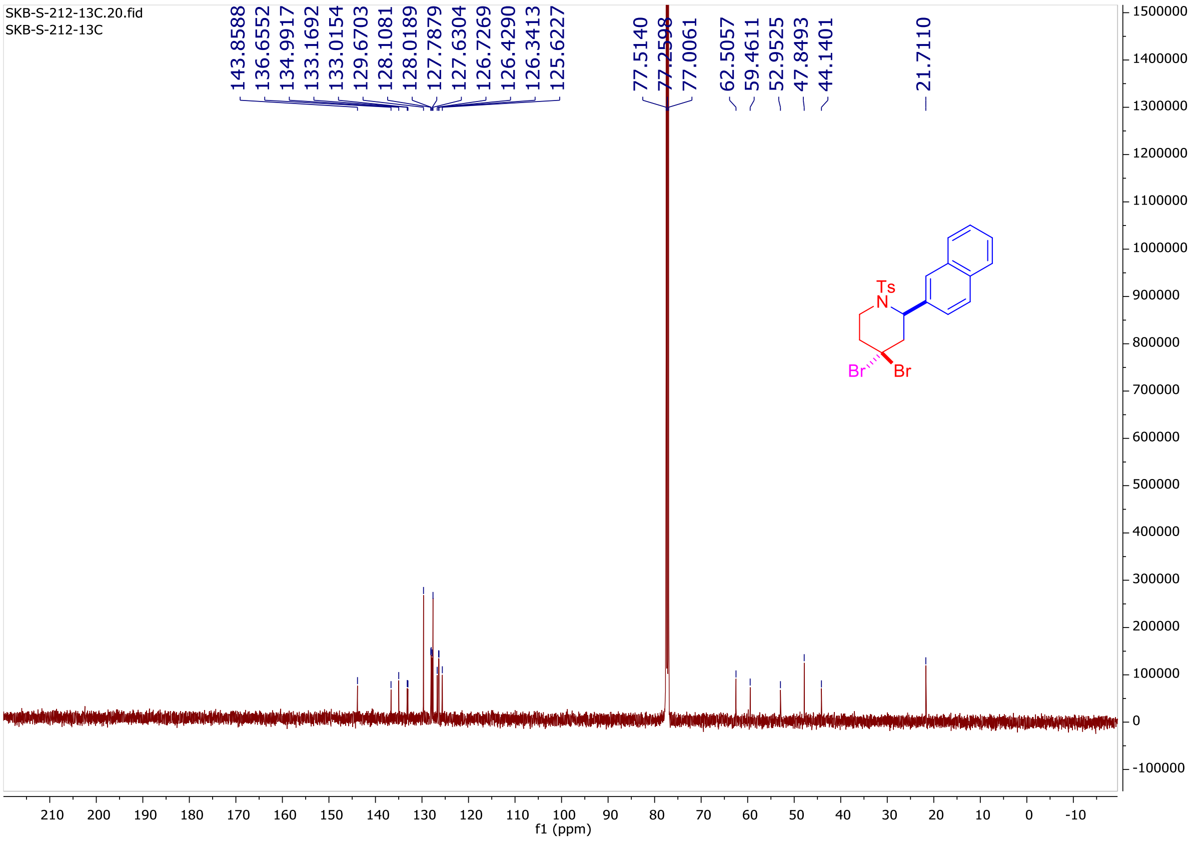

^{1}H (400 MHz, CDCl3) and ^{13}C{^{1}H} (100 MHz, CDCl3) spectra of 3ao:

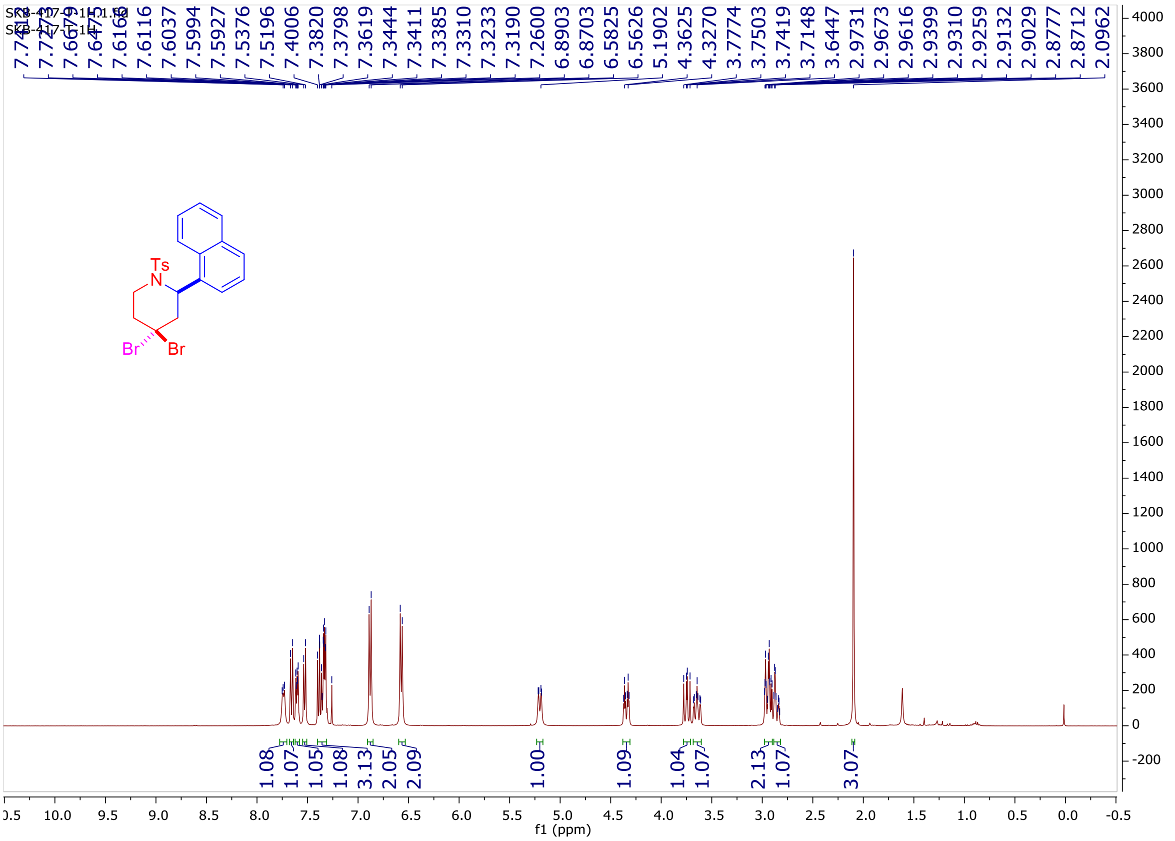

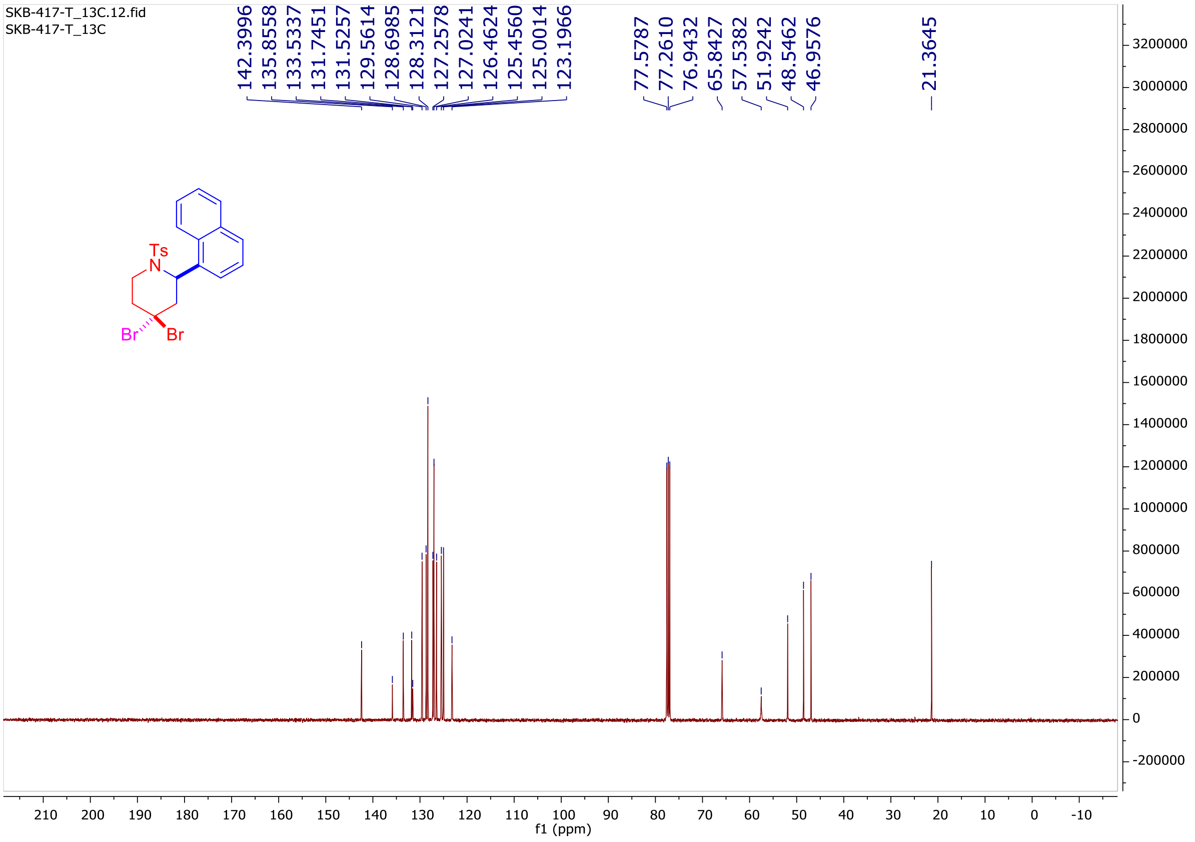

^{1}H (400 MHz, $CDCl_3$) and ^{13}C{^{1}H} (125 MHz, $CDCl_3$) spectra of 3ap:

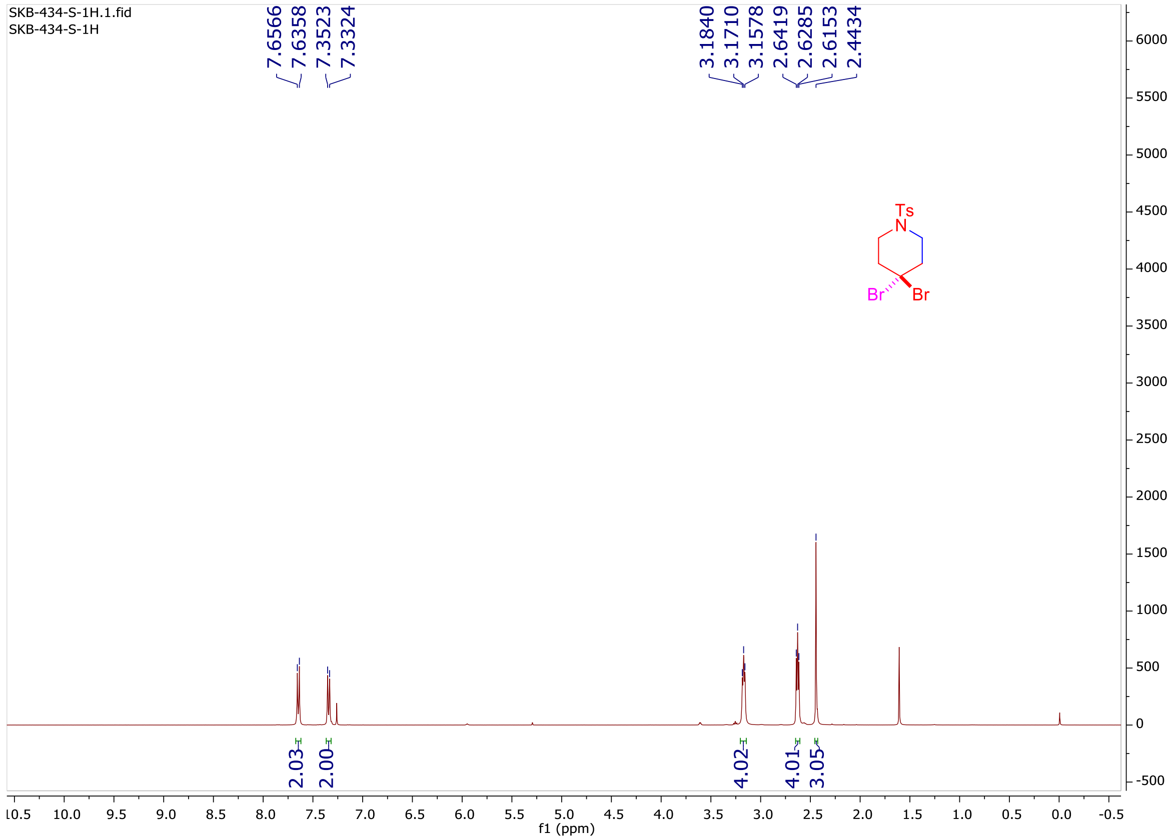

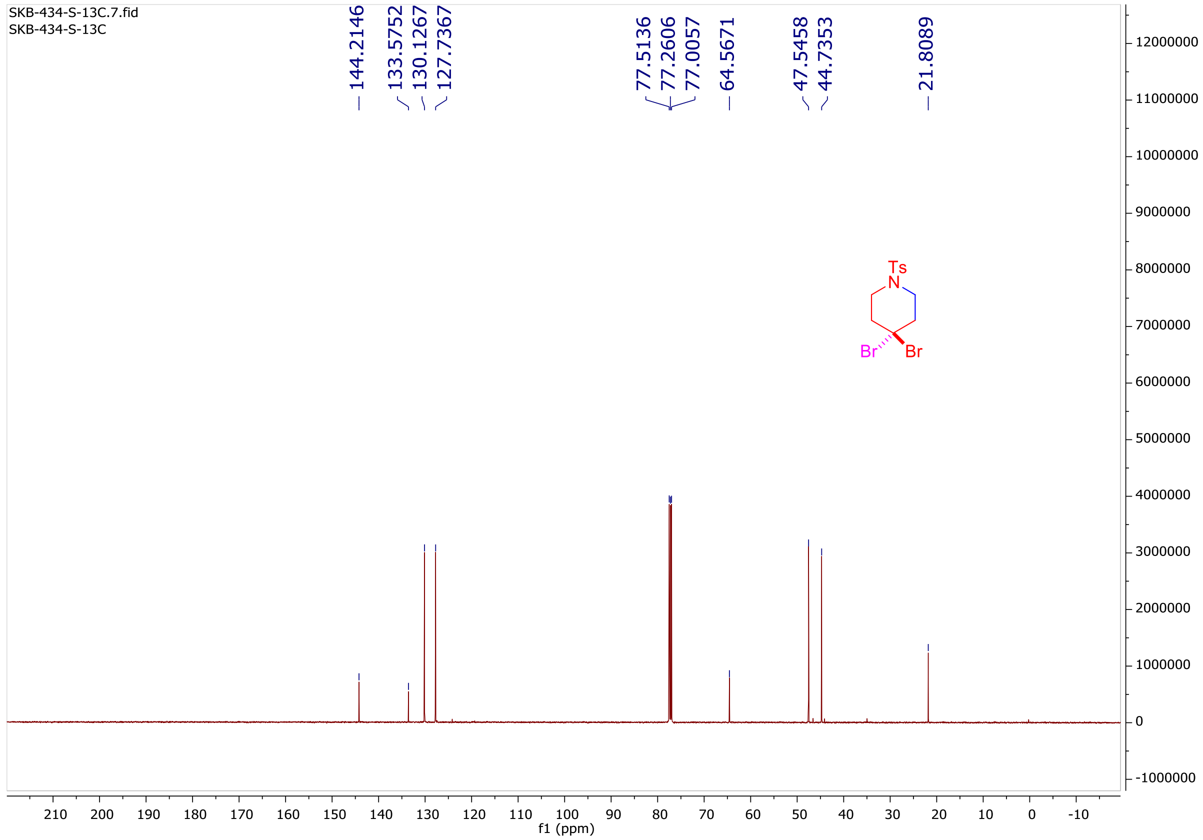

^{1}H (600 MHz, $CDCl_3$) and ^{13}C{^{1}H} (150 MHz, $CDCl_3$) spectra of 3aq:

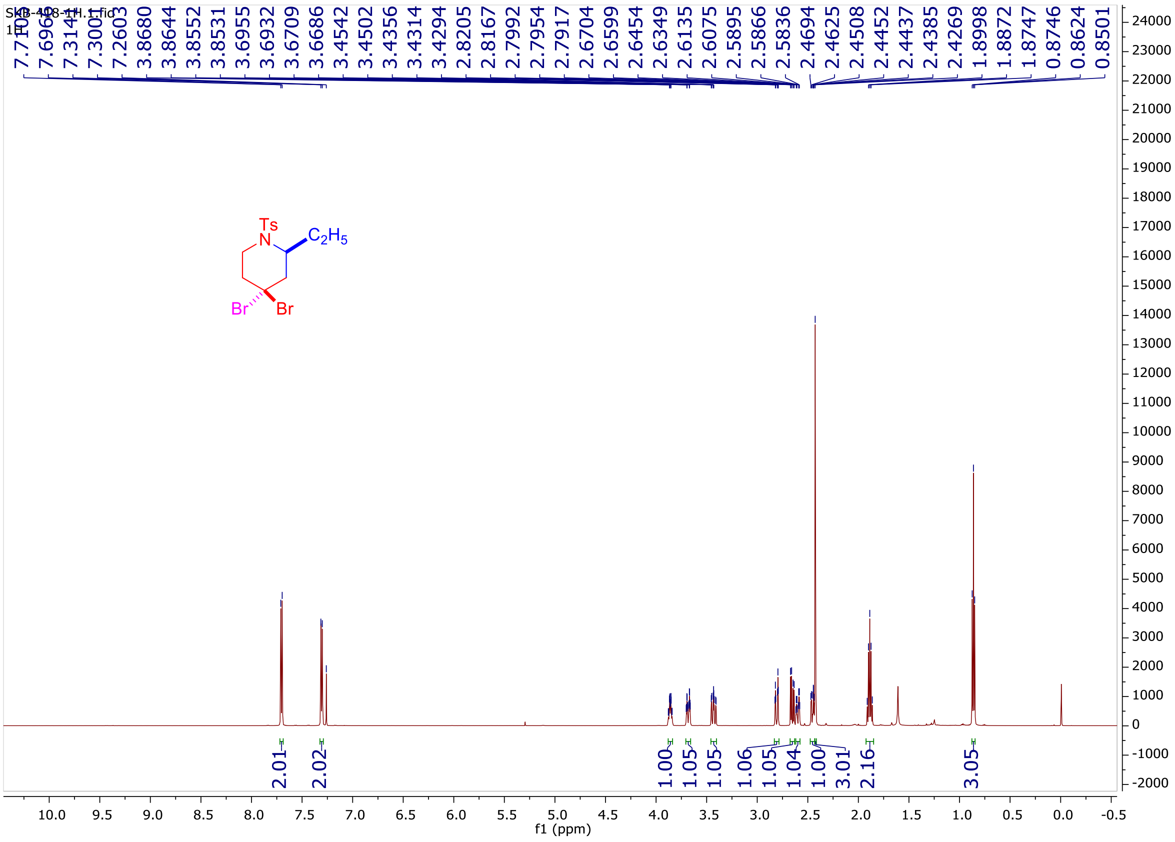

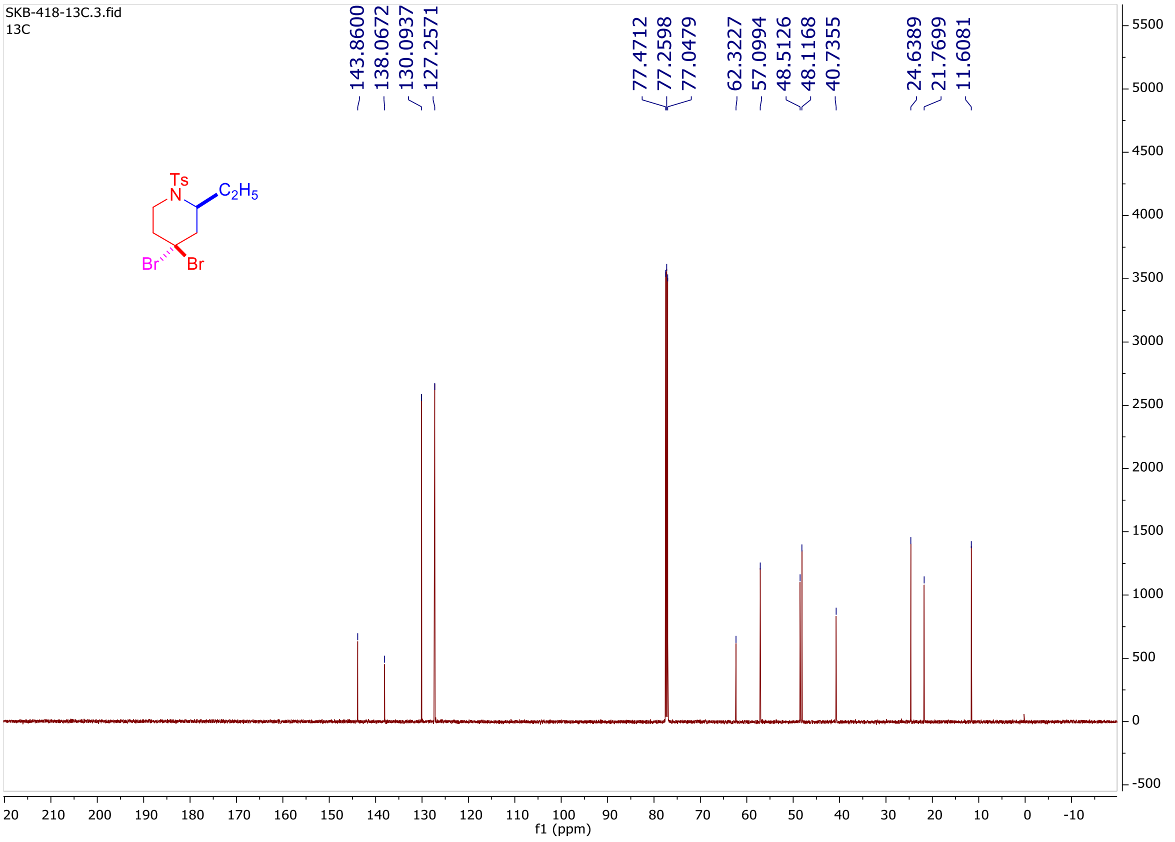

^{1}H (600 MHz, $CDCl_3$) and $^{13}C\{^{1}H\}$ (150 MHz, $CDCl_3$) spectra of 3ar:

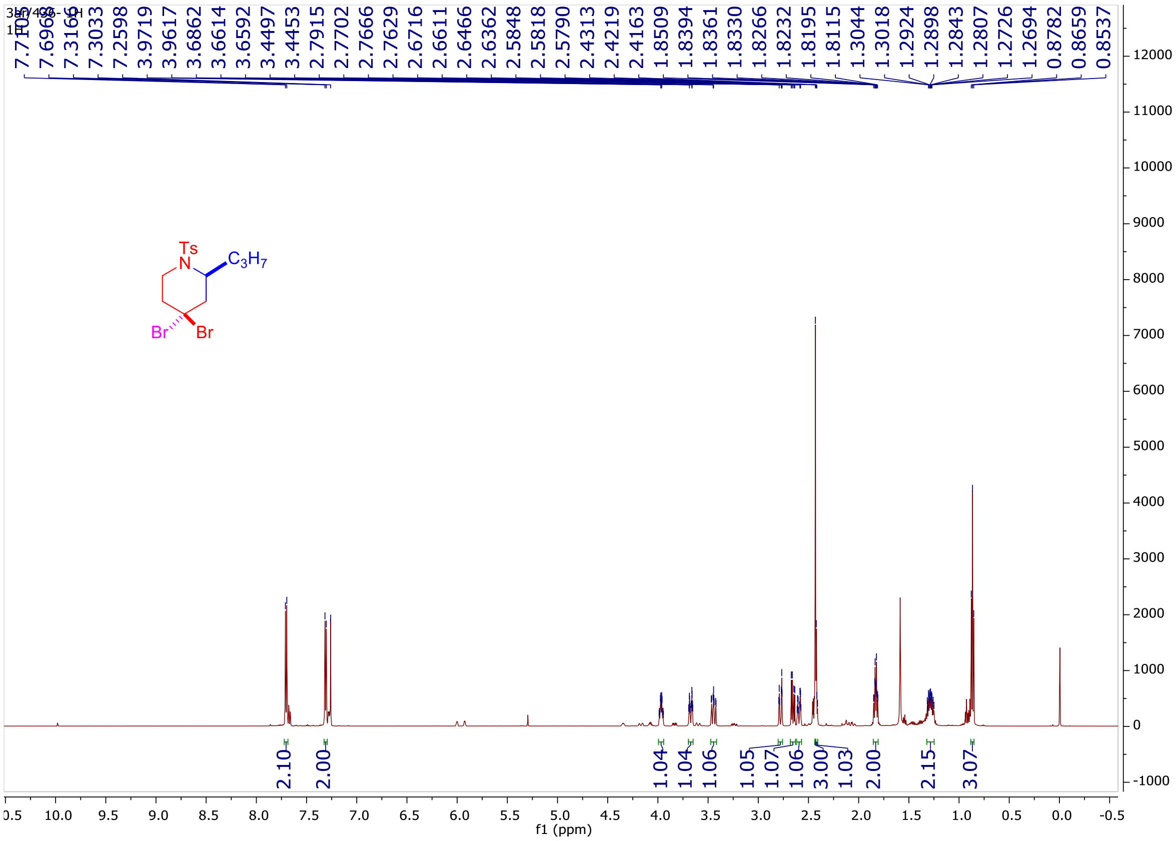

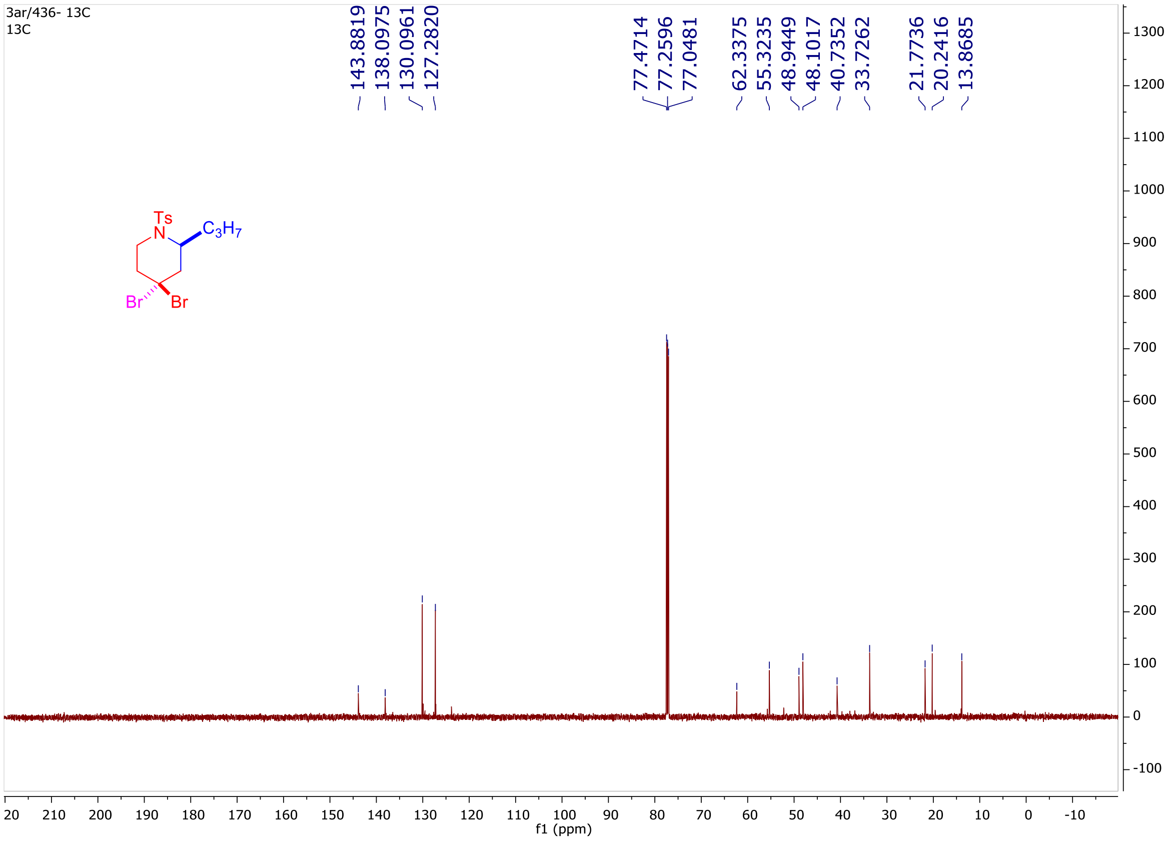

^{1}H (400 MHz, CDCl$_3$) and ^{13}C{^{1}H} (100 MHz, CDCl$_3$) spectra of 3as:

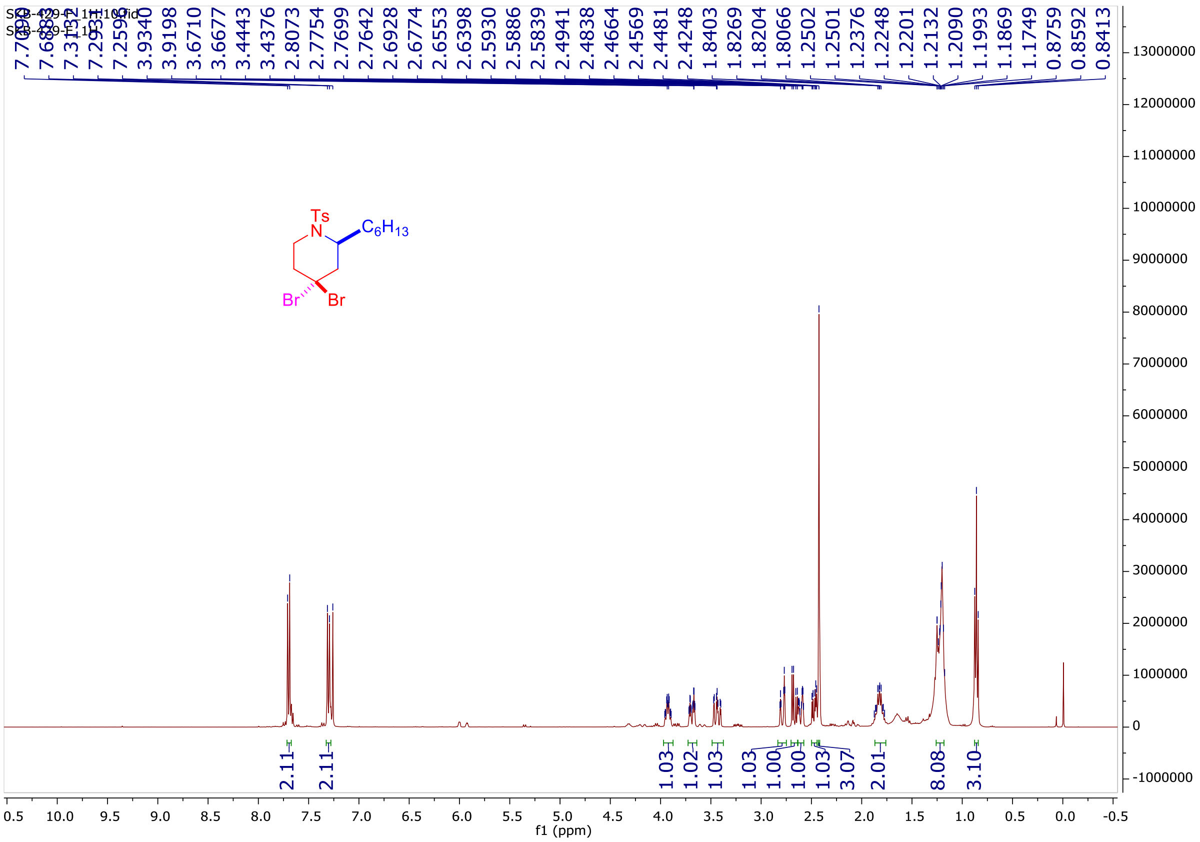

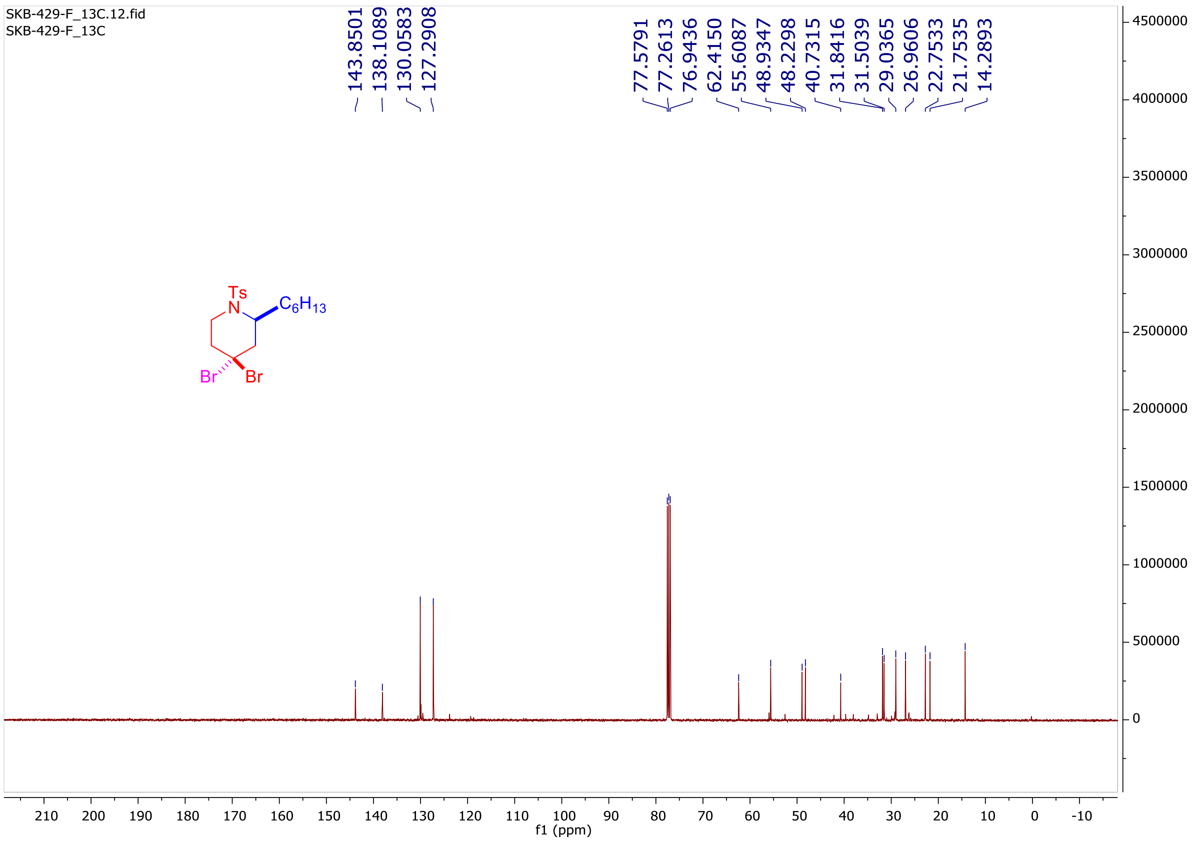

^{1}H (400 MHz, $CDCl_3$) and $^{13}C\{^{1}H\}$ (125 MHz, $CDCl_3$) spectra of 3at:

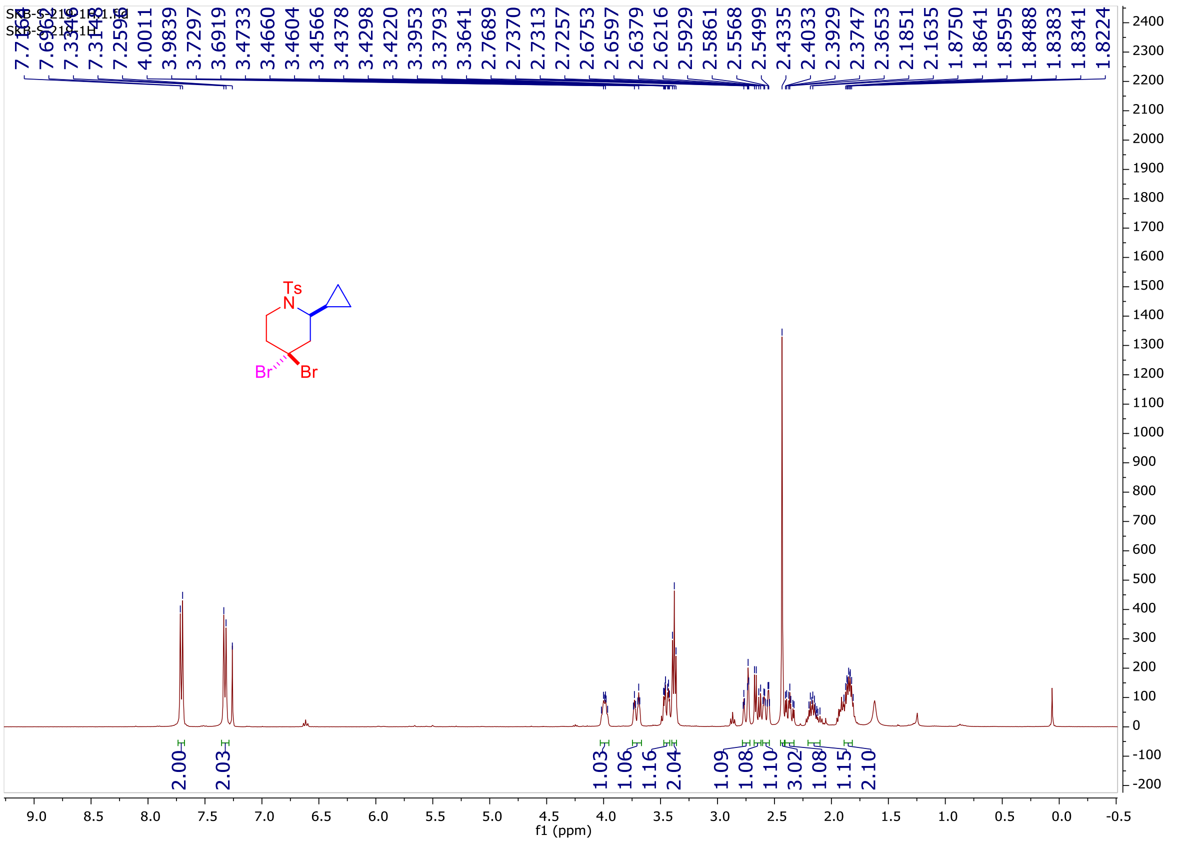

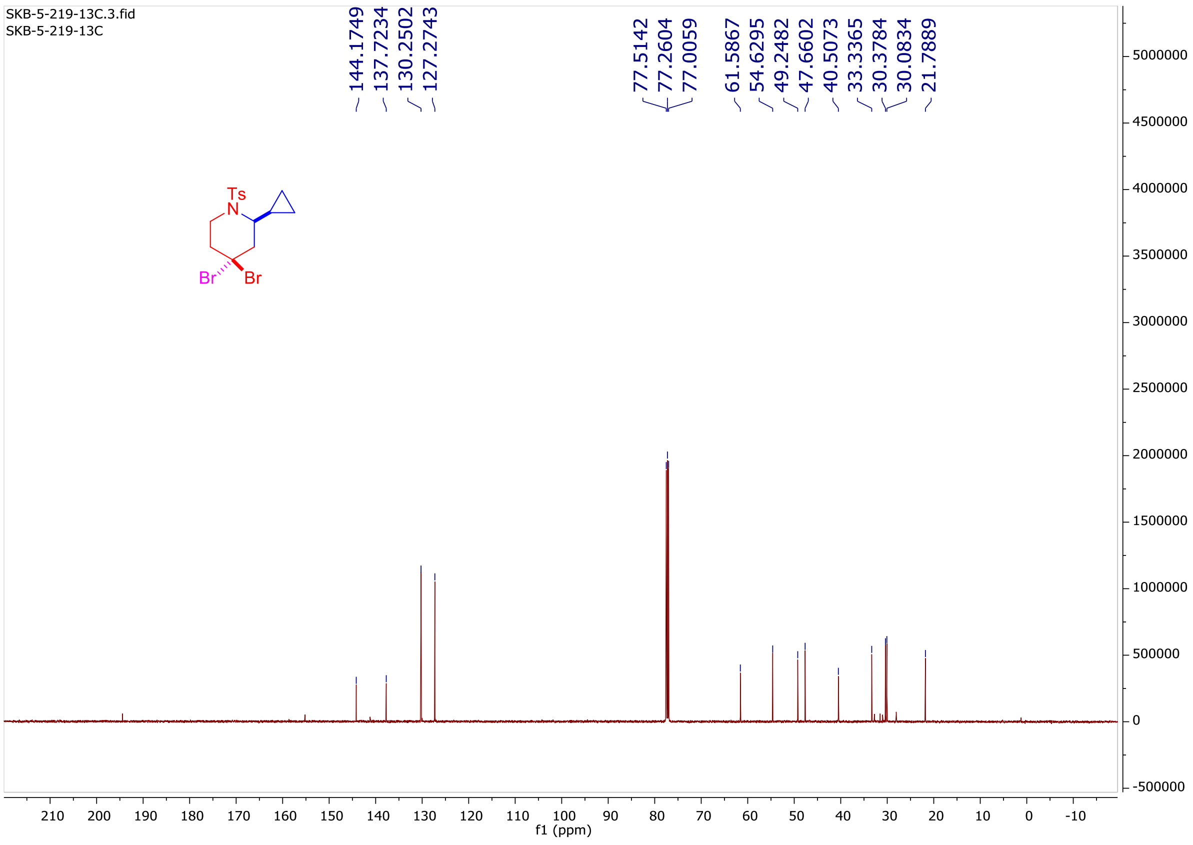

^{1}H (400 MHz, CDCl3) and ^{13}C{^{1}H} (150 MHz, CDCl3) spectra of 3au:

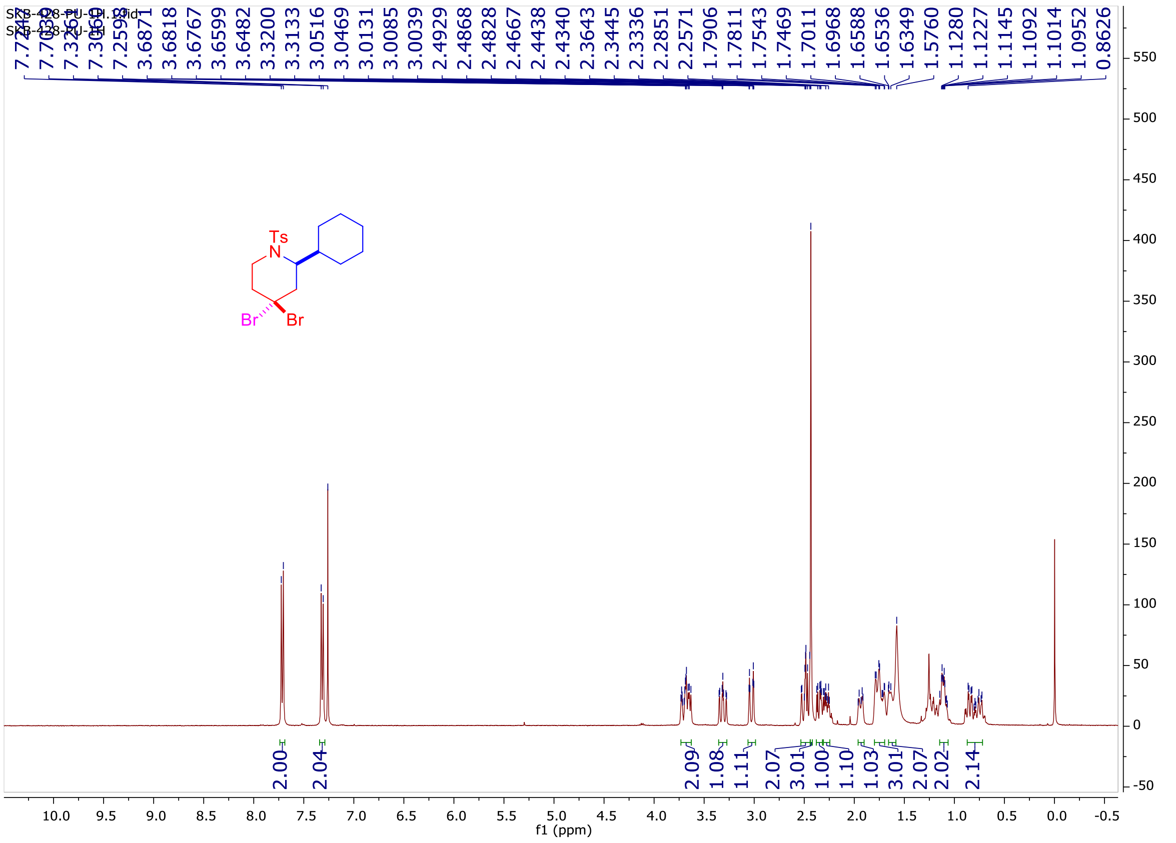

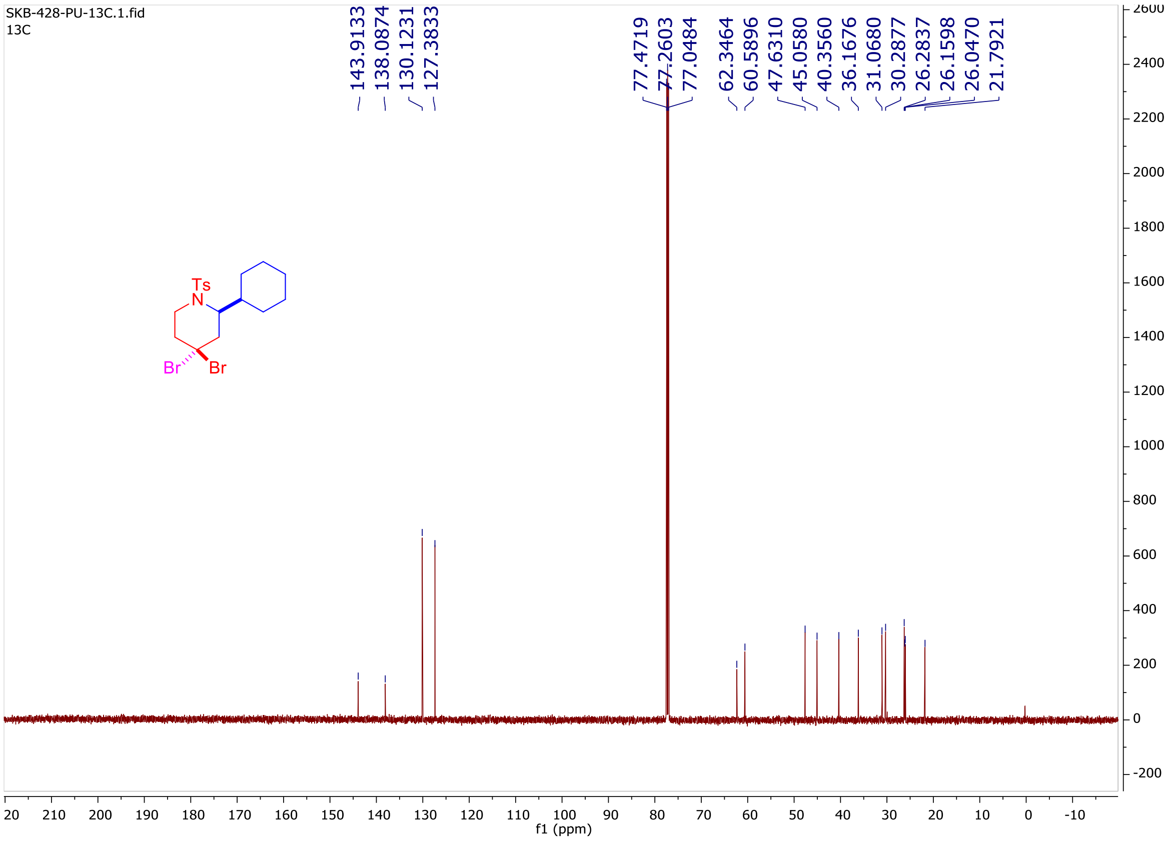

^{1}H (400 MHz, $CDCl_3$) and $^{13}C\{^{1}H\}$ (125 MHz, $CDCl_3$) spectra of 3dj:

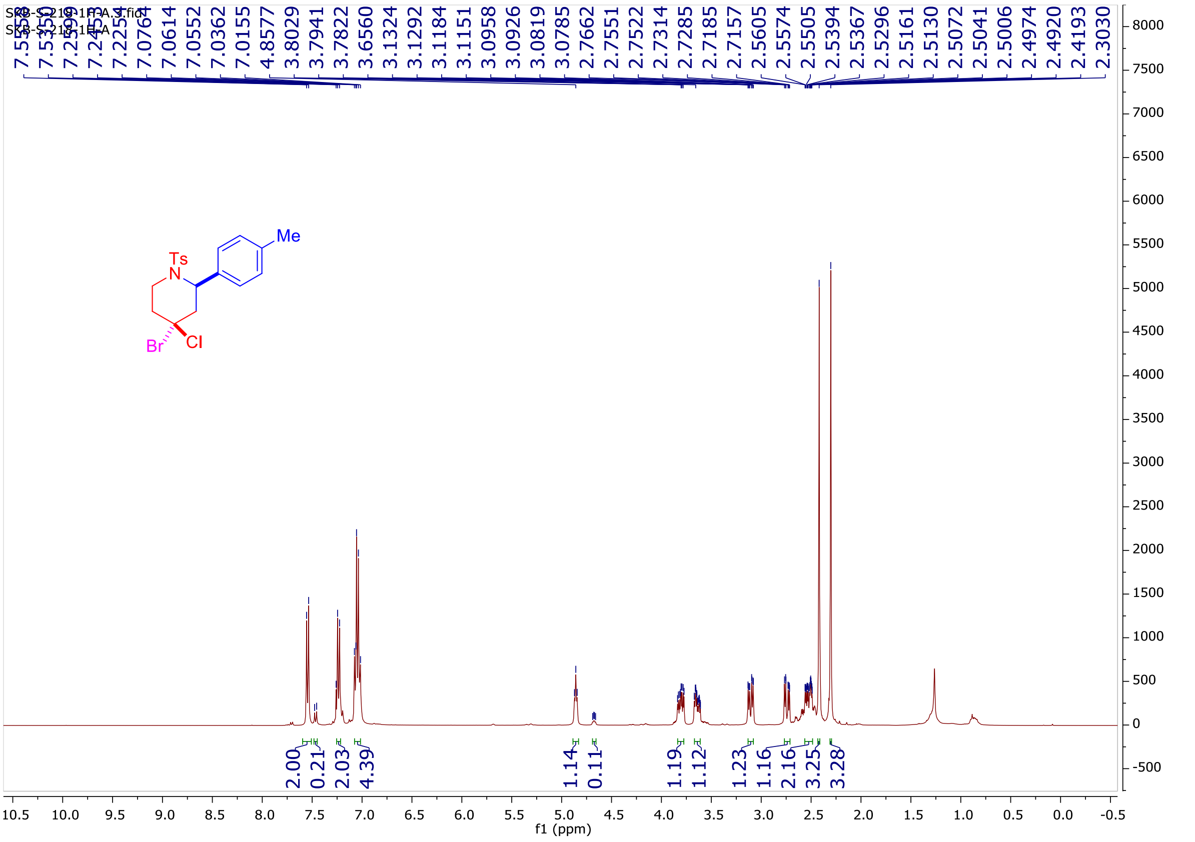

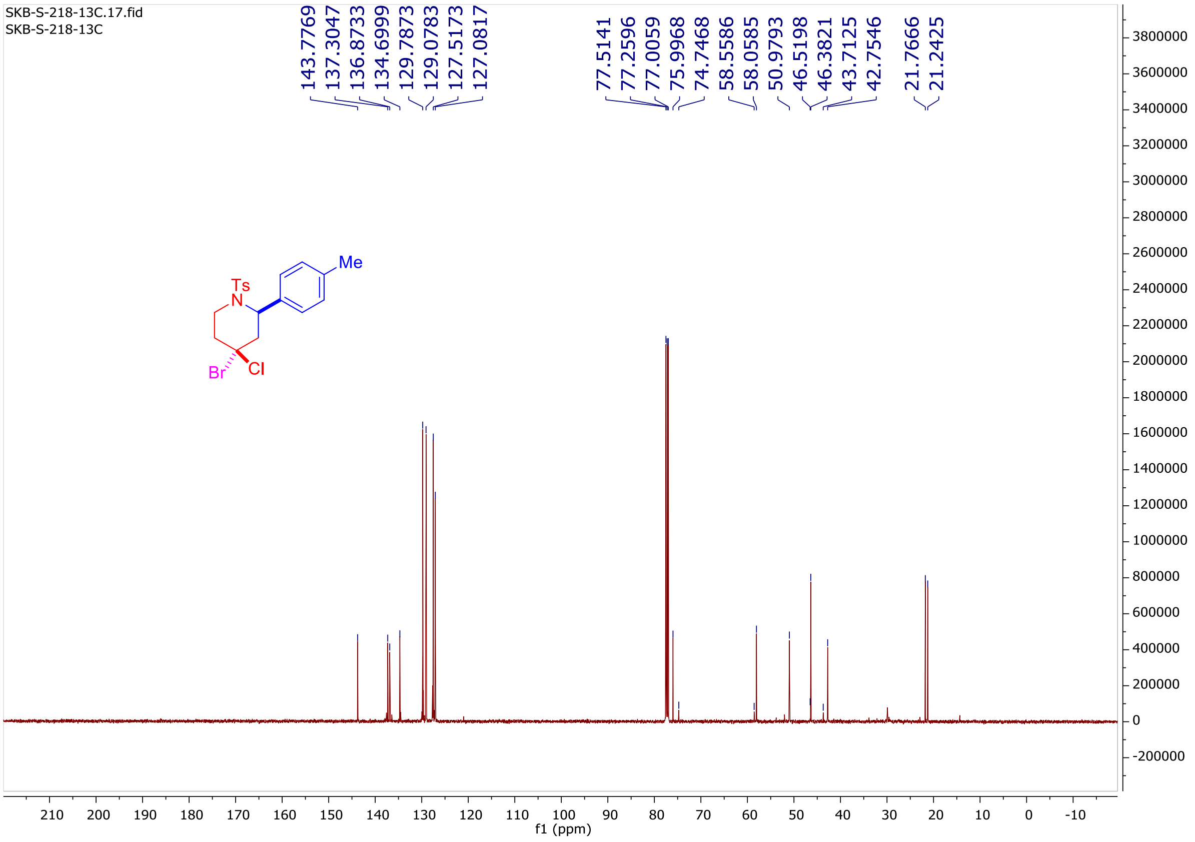

^{1}H (400 MHz, CDCl3) and ^{13}C{^{1}H} (100 MHz, CDCl3) spectra of 3ed:

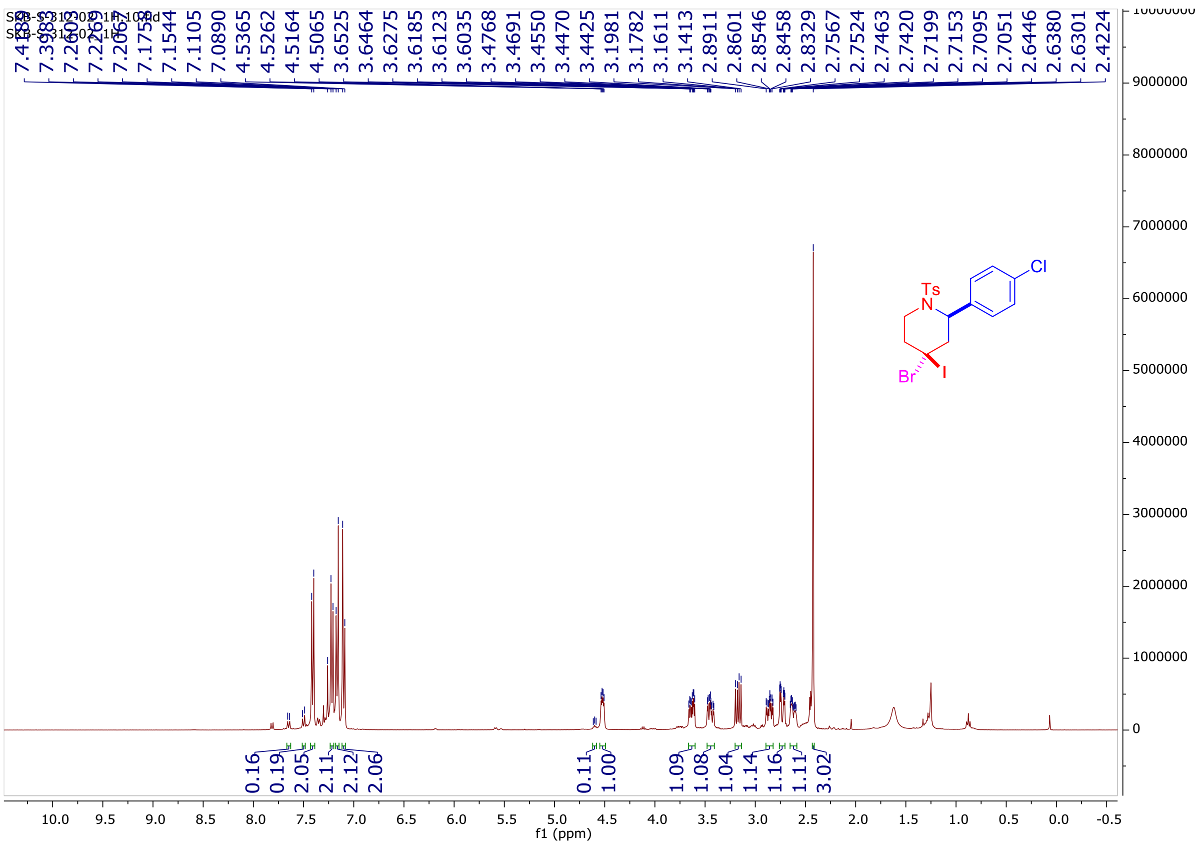

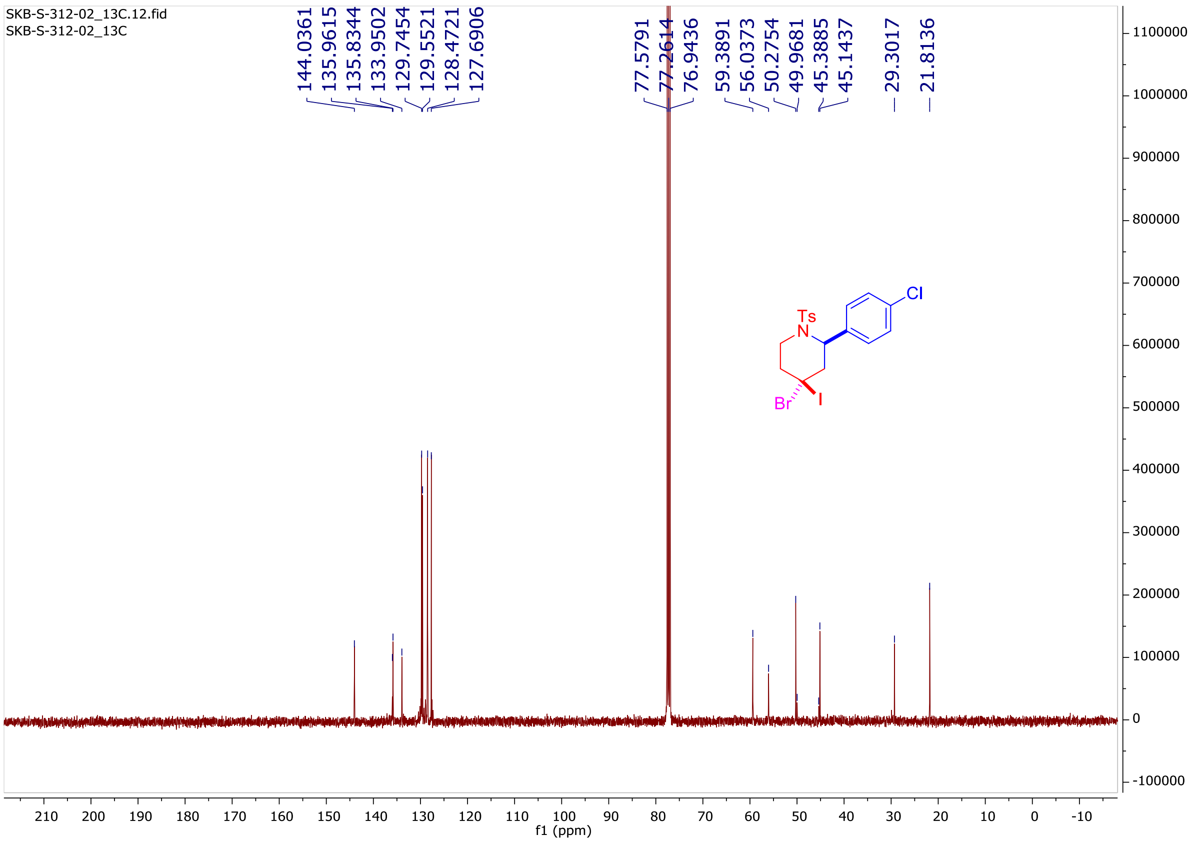

^{1}H (500 MHz, $CDCl_3$) and $^{13}C\{^{1}H\}$ (125 MHz, $CDCl_3$) spectra of 4aa:

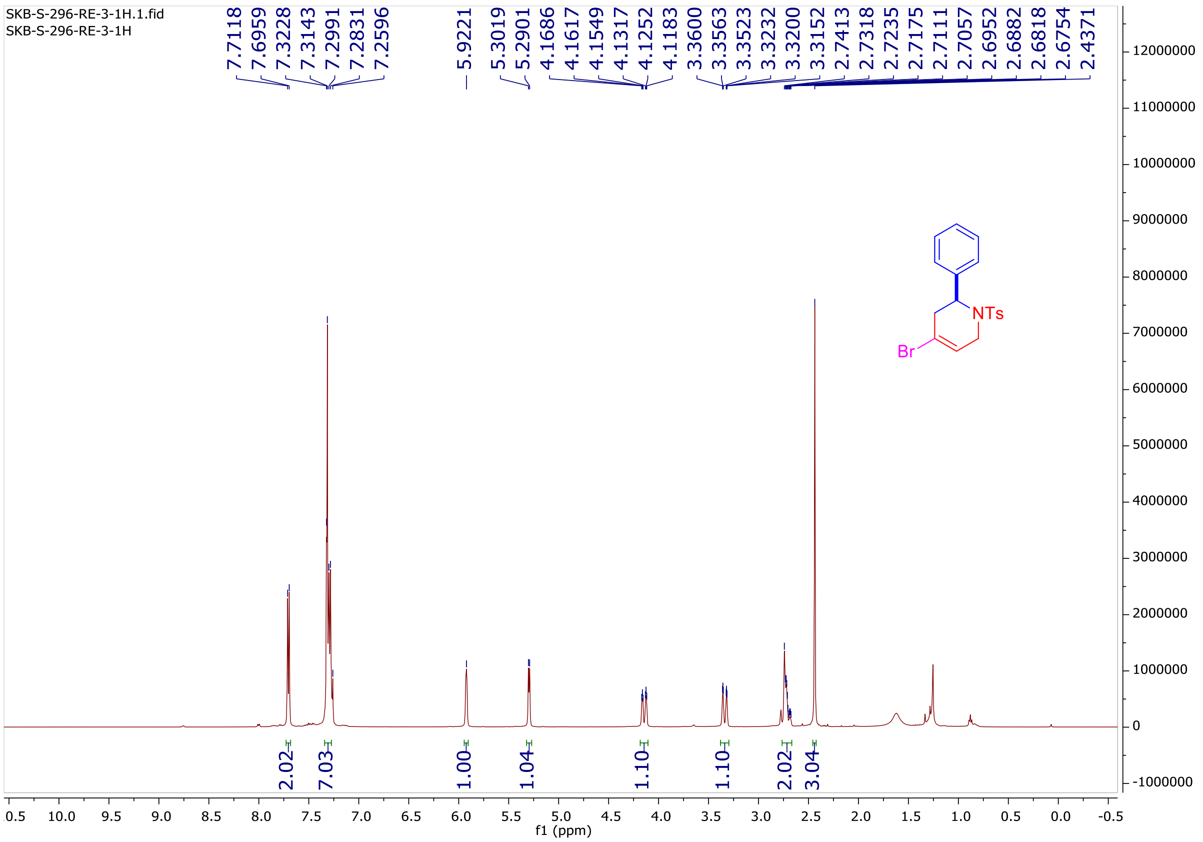

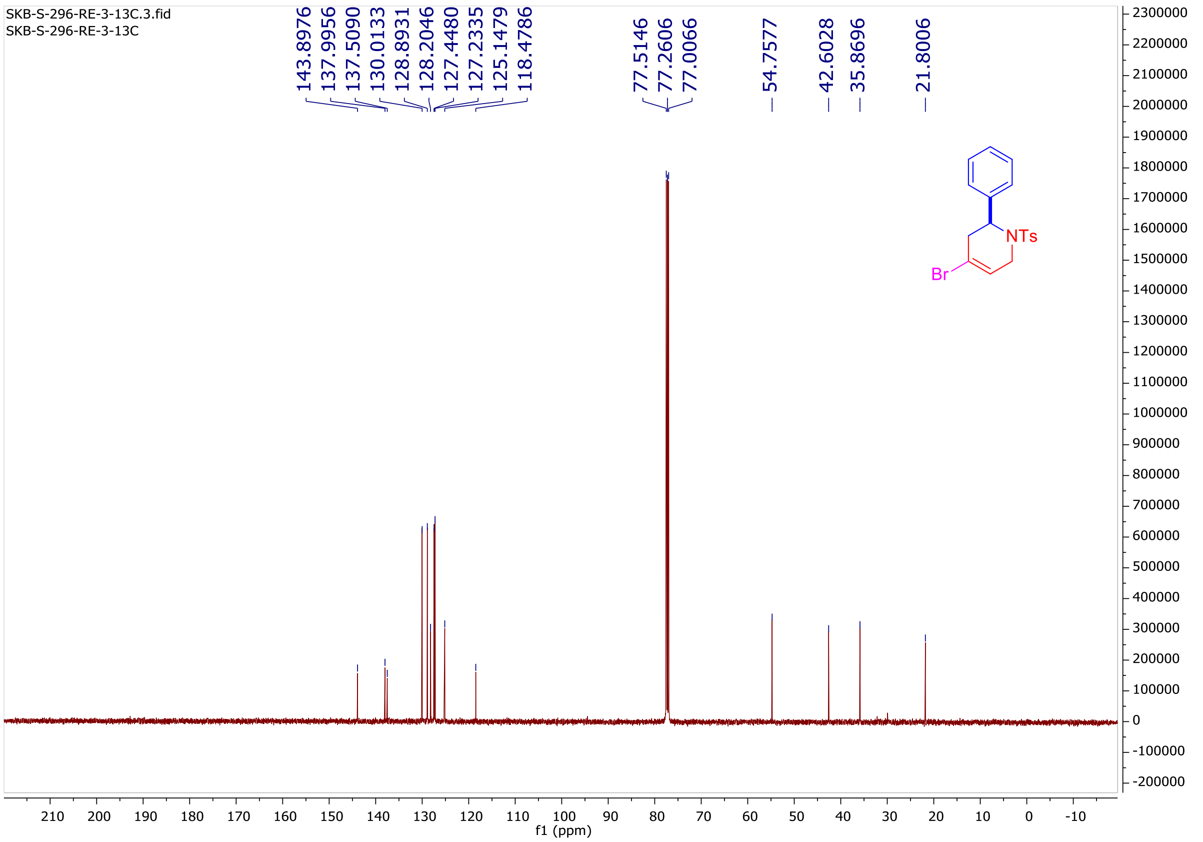

^{1}H (400 MHz, CDCl$_3$) and ^{13}C{^{1}H} (100 MHz, CDCl$_3$) spectra of 4ac:

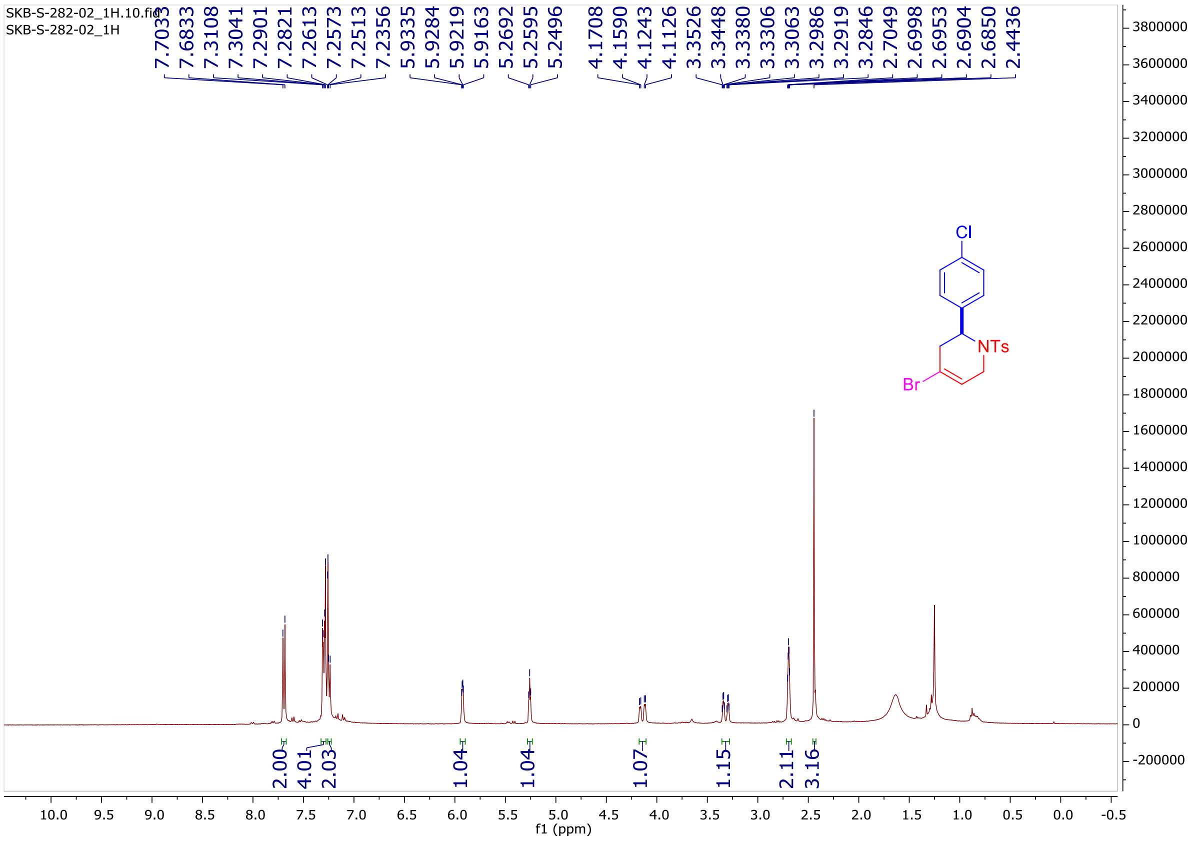

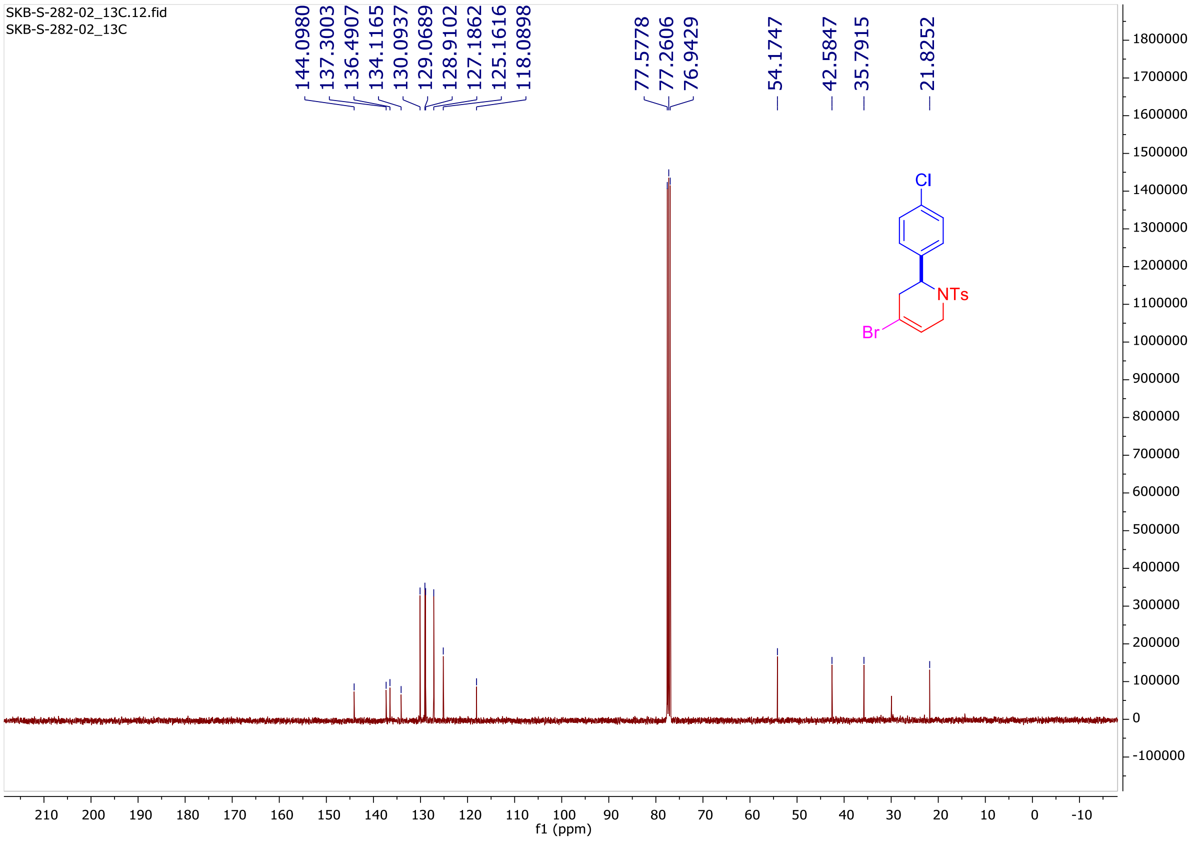

^{1}H (400 MHz, $CDCl_3$) and $^{13}C\{^{1}H\}$ (100 MHz, $CDCl_3$) spectra of 4ad:

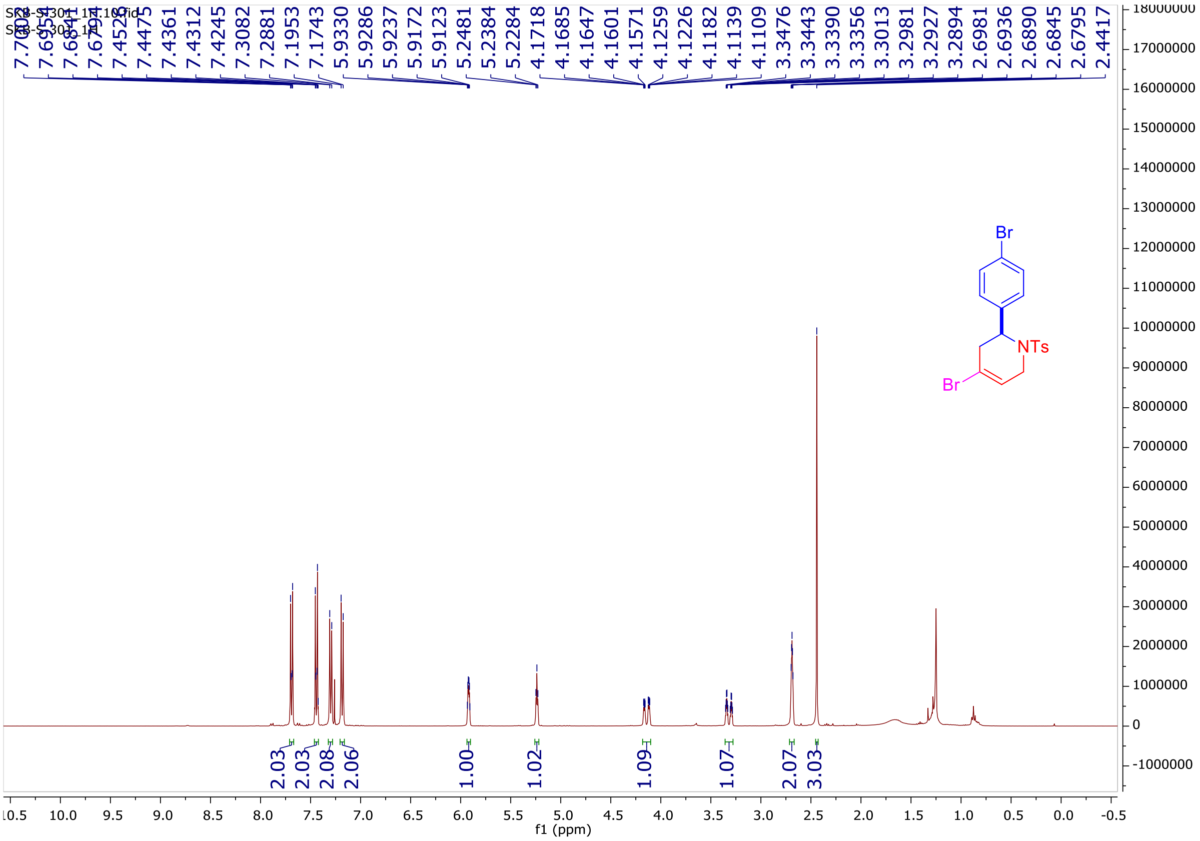

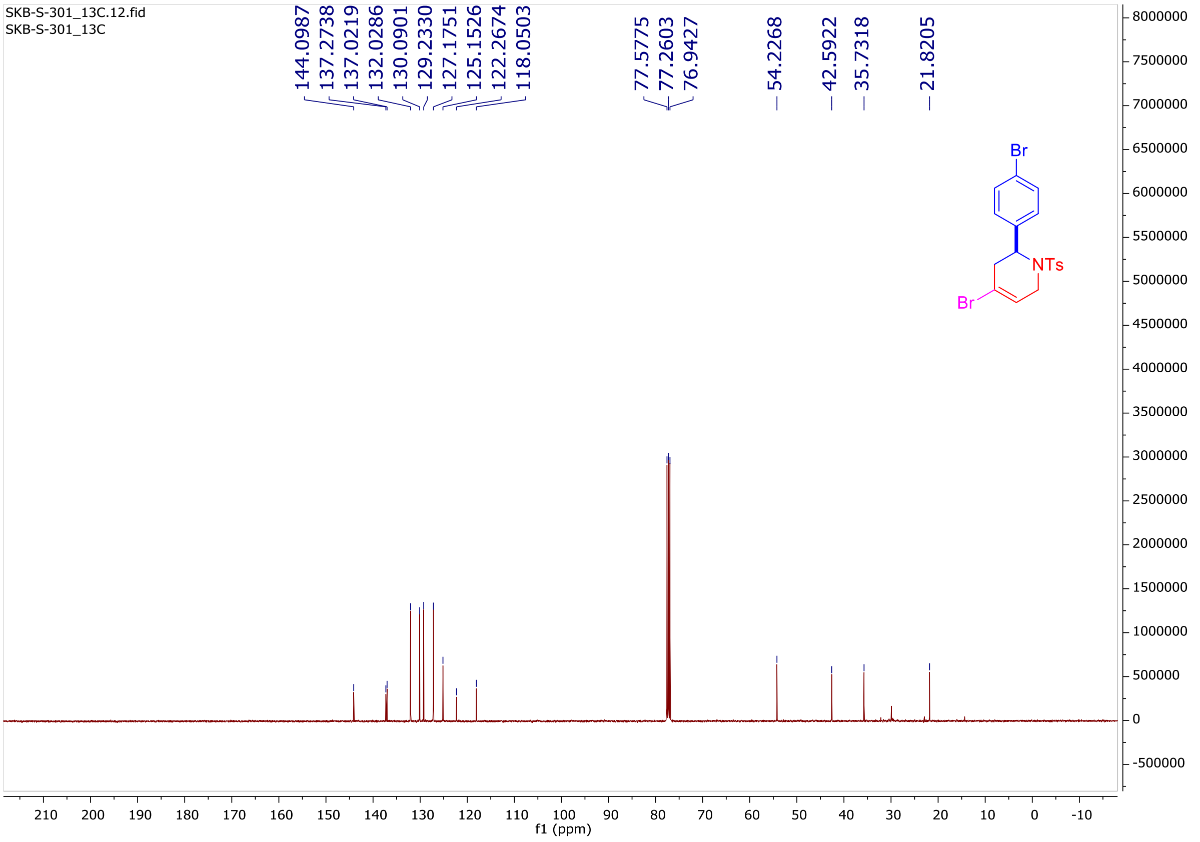

^{1}H (400 MHz, $CDCl_3$) and $^{13}C\{^{1}H\}$ (100 MHz, $CDCl_3$) spectra of 4ai:

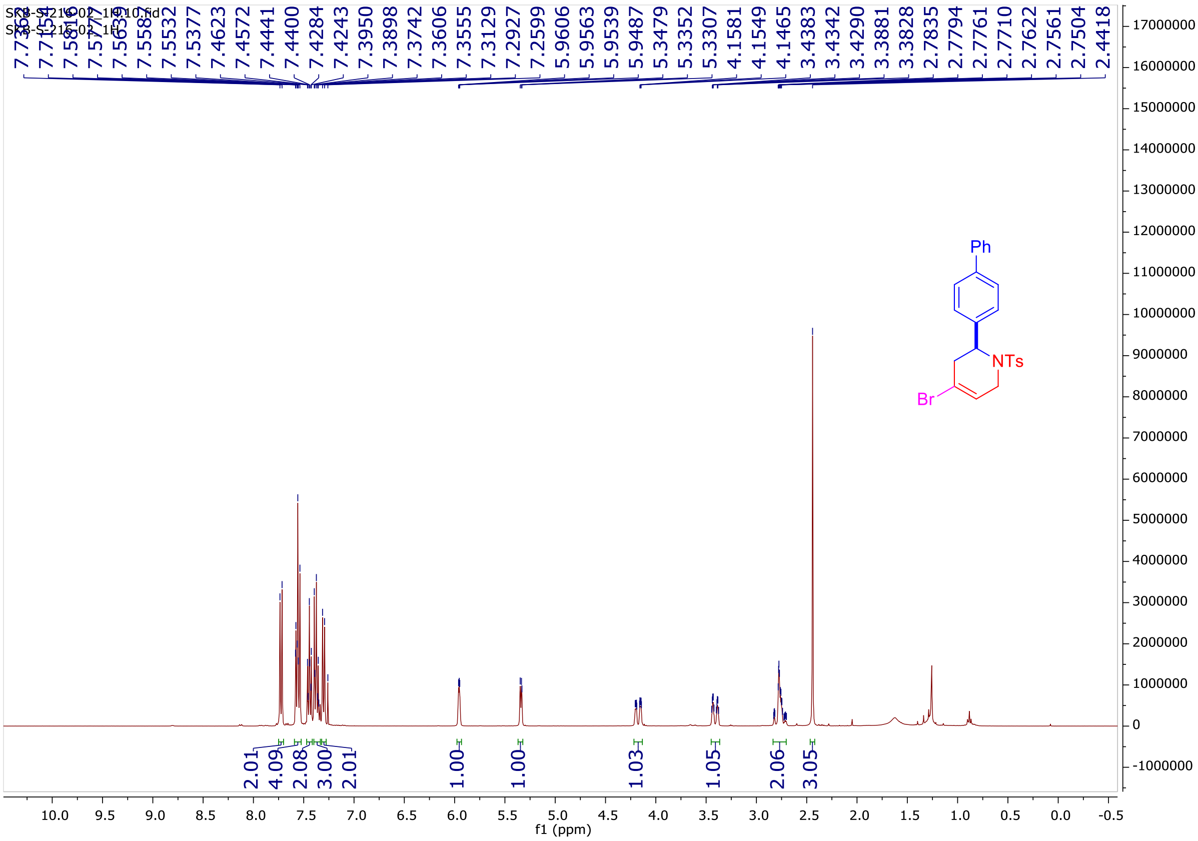

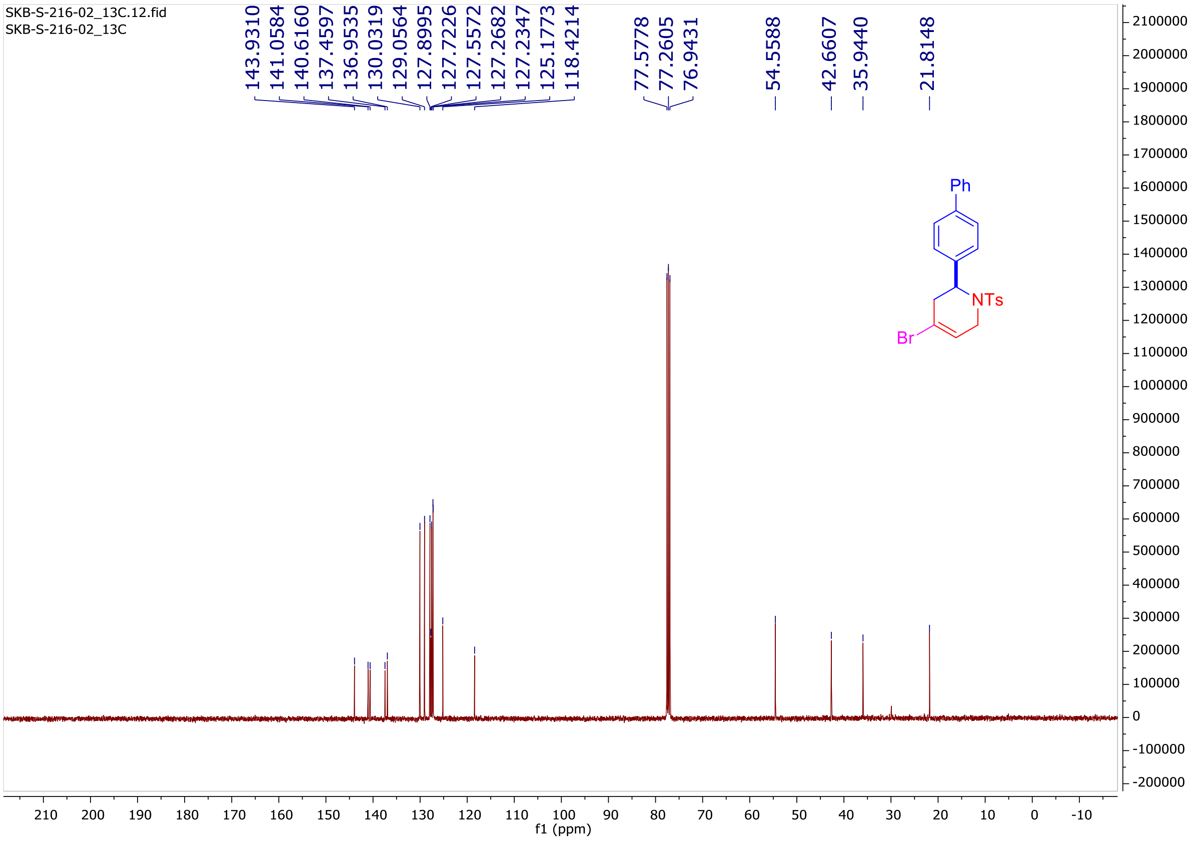

^{1}H (400 MHz, $CDCl_3$) and $^{13}C\{^{1}H\}$ (100 MHz, $CDCl_3$) spectra of 4aj:

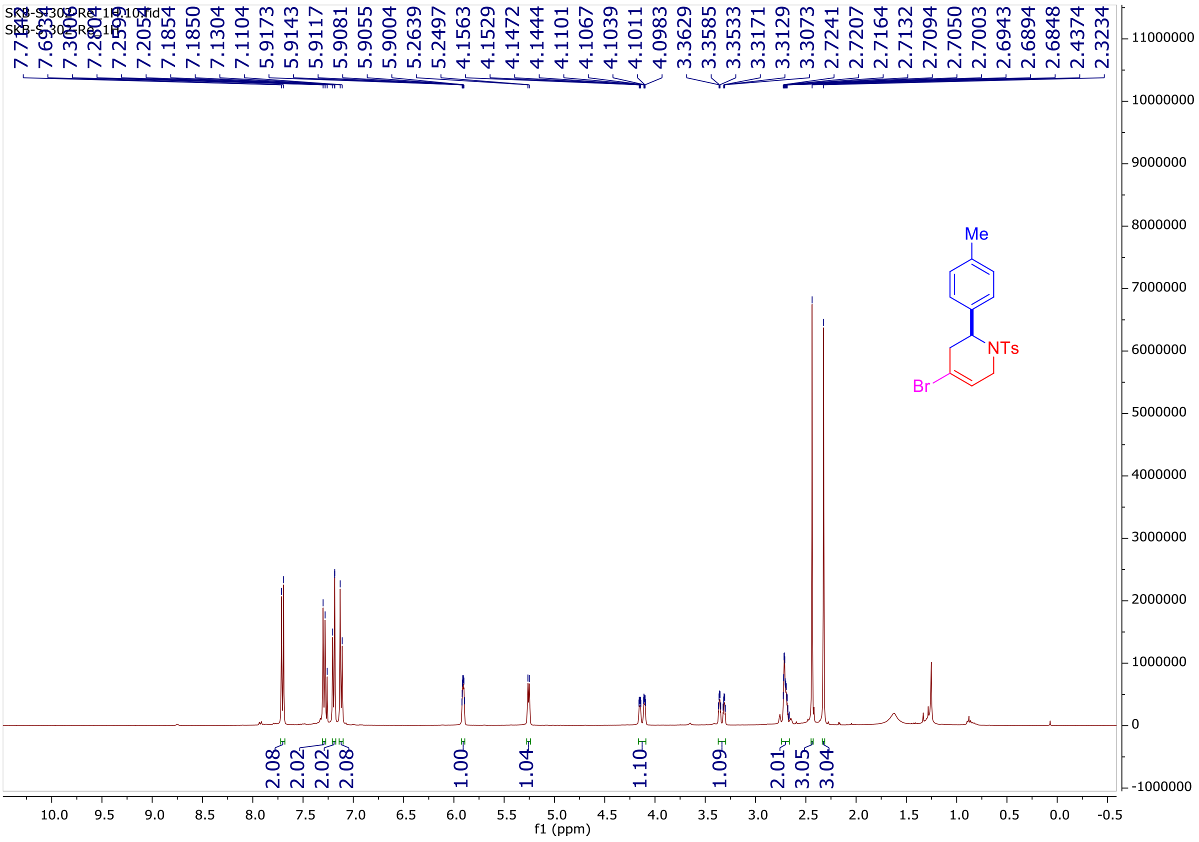

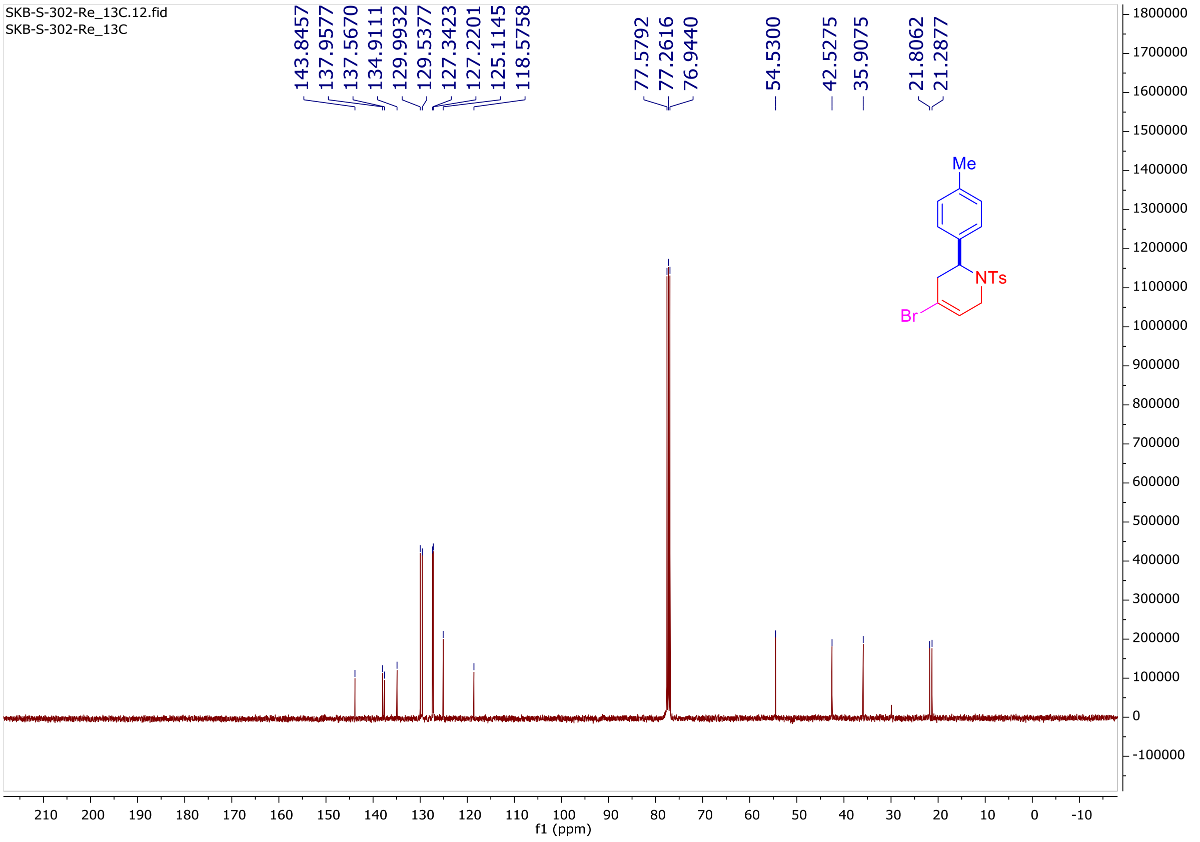

^{1}H (500 MHz, CDCl$_3$) and ^{13}C{^{1}H} (125 MHz, CDCl$_3$) spectra of 4ao:

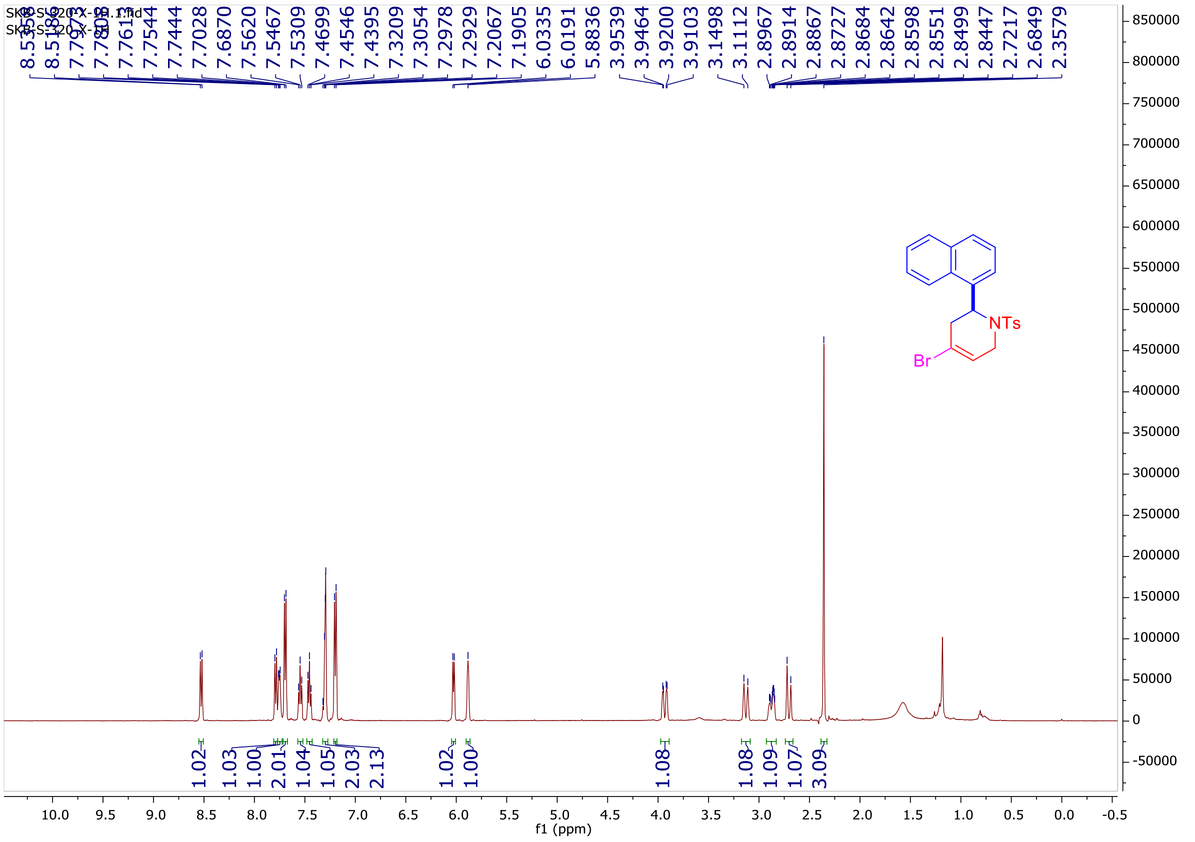

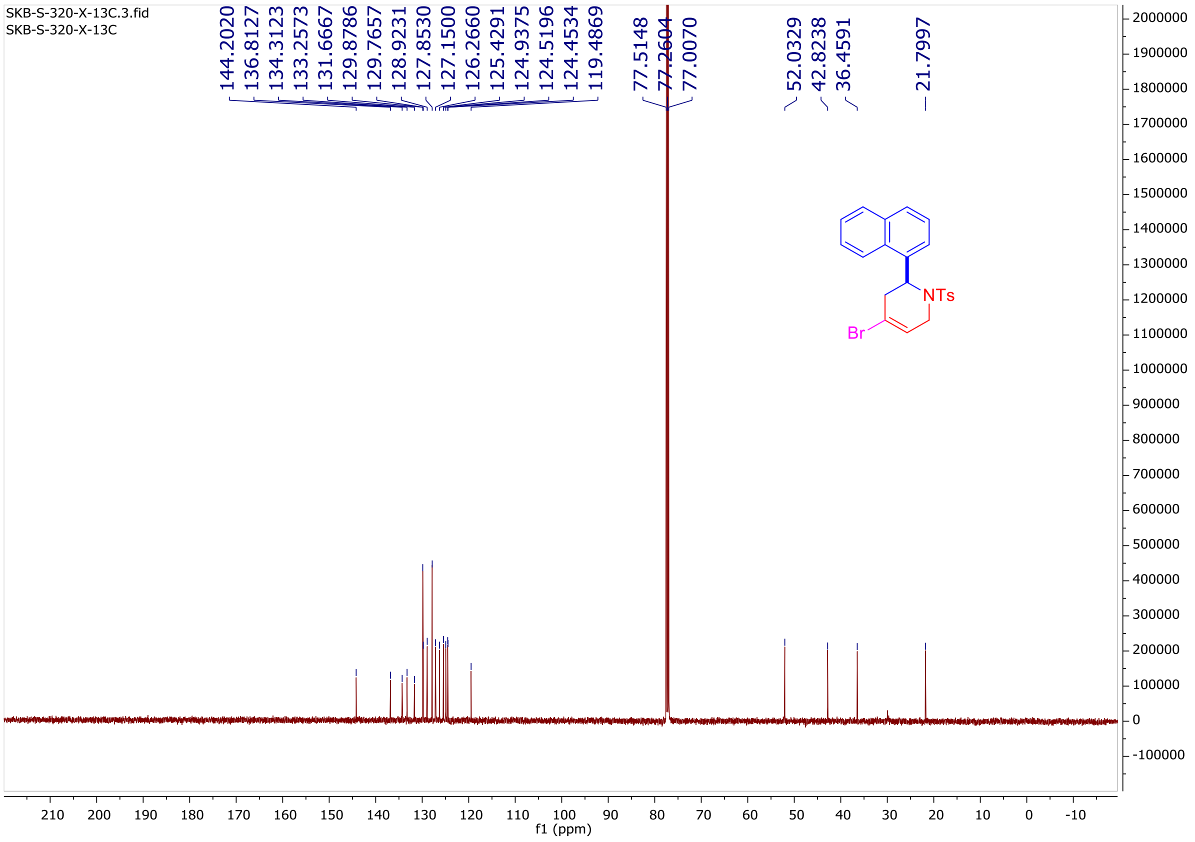

^{1}H (400 MHz, $CDCl_3$) and ^{13}C{^{1}H} (125 MHz, $CDCl_3$) spectra of 4aq:

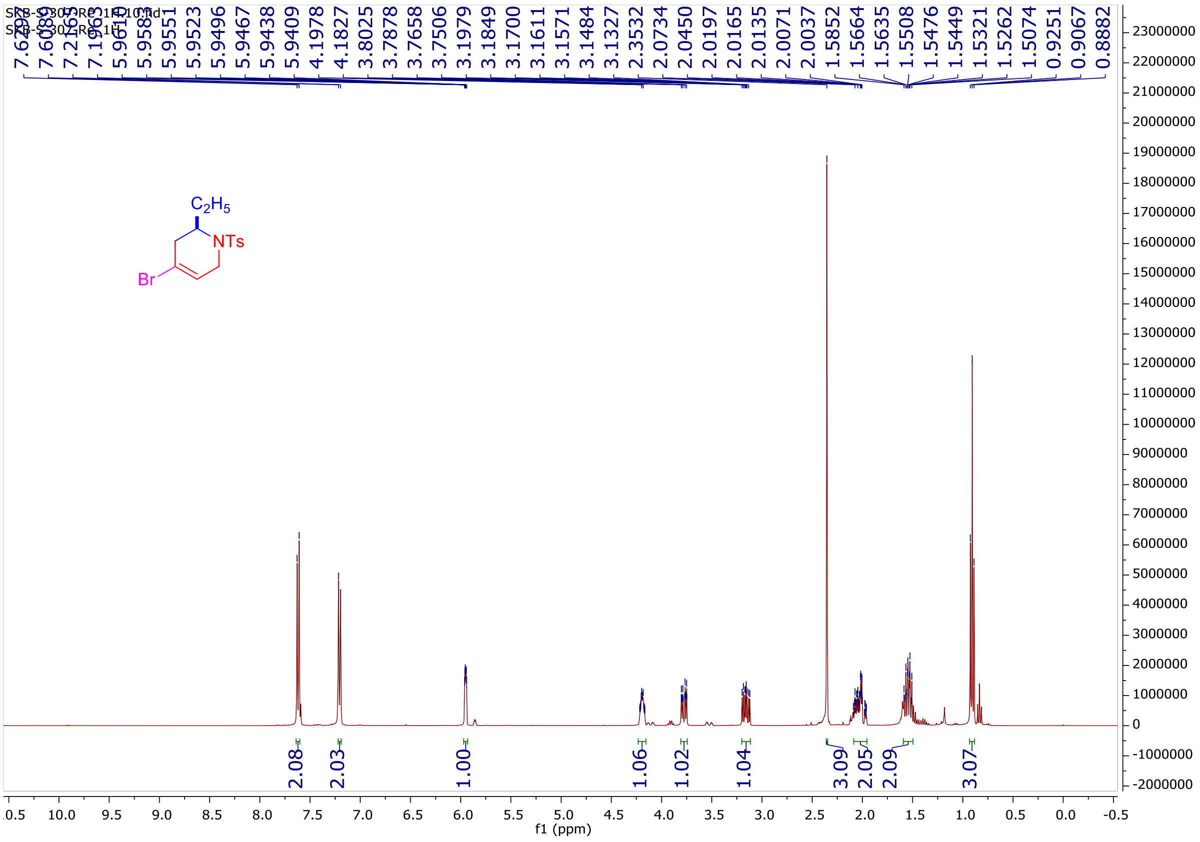

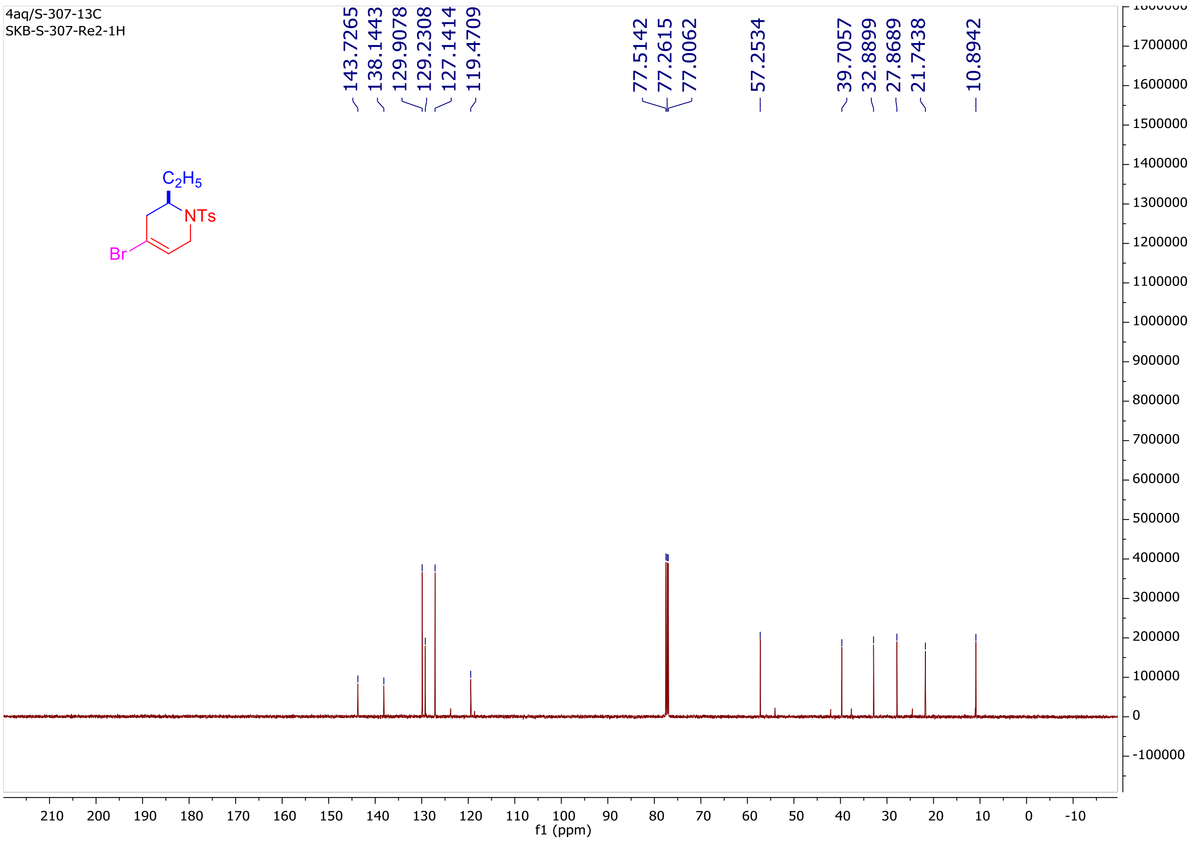

^{1}H (400 MHz, $CDCl_3$) and $^{13}C\{^{1}H\}$ (125 MHz, $CDCl_3$) spectra of 4db:

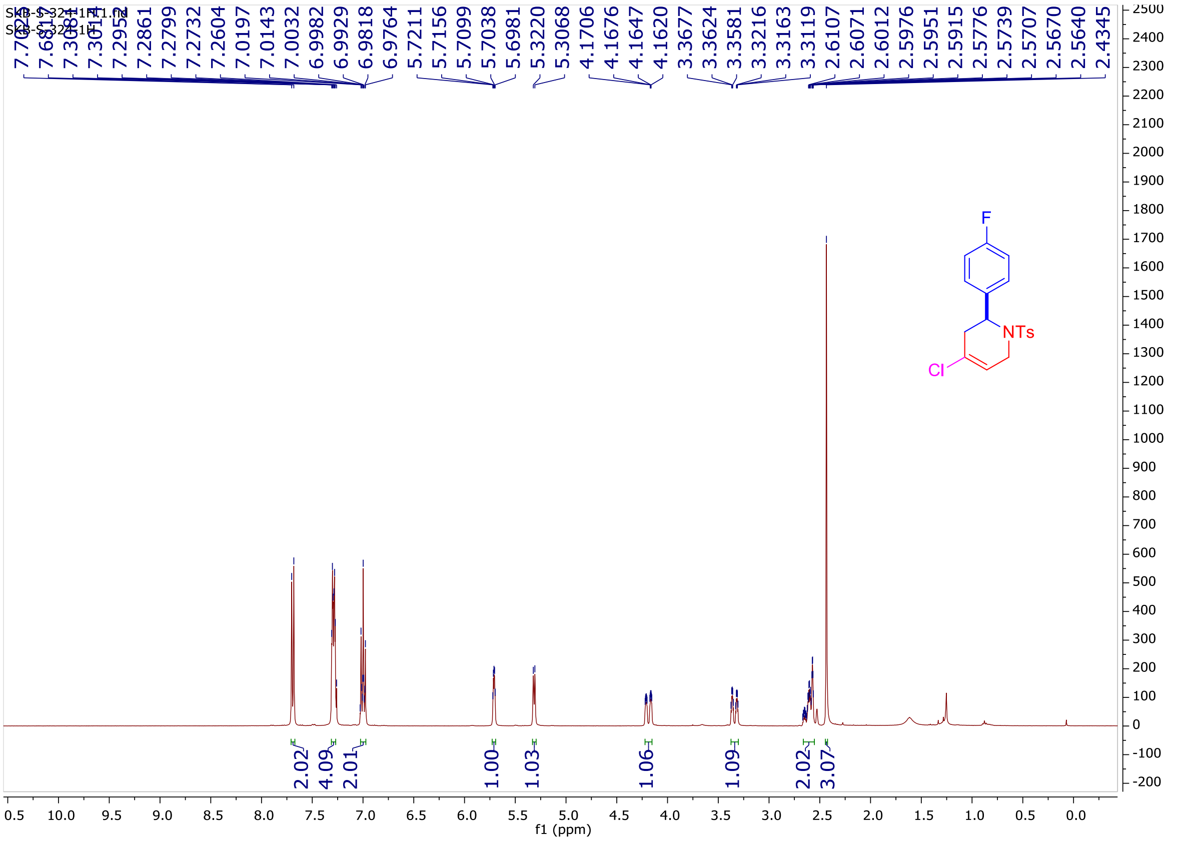

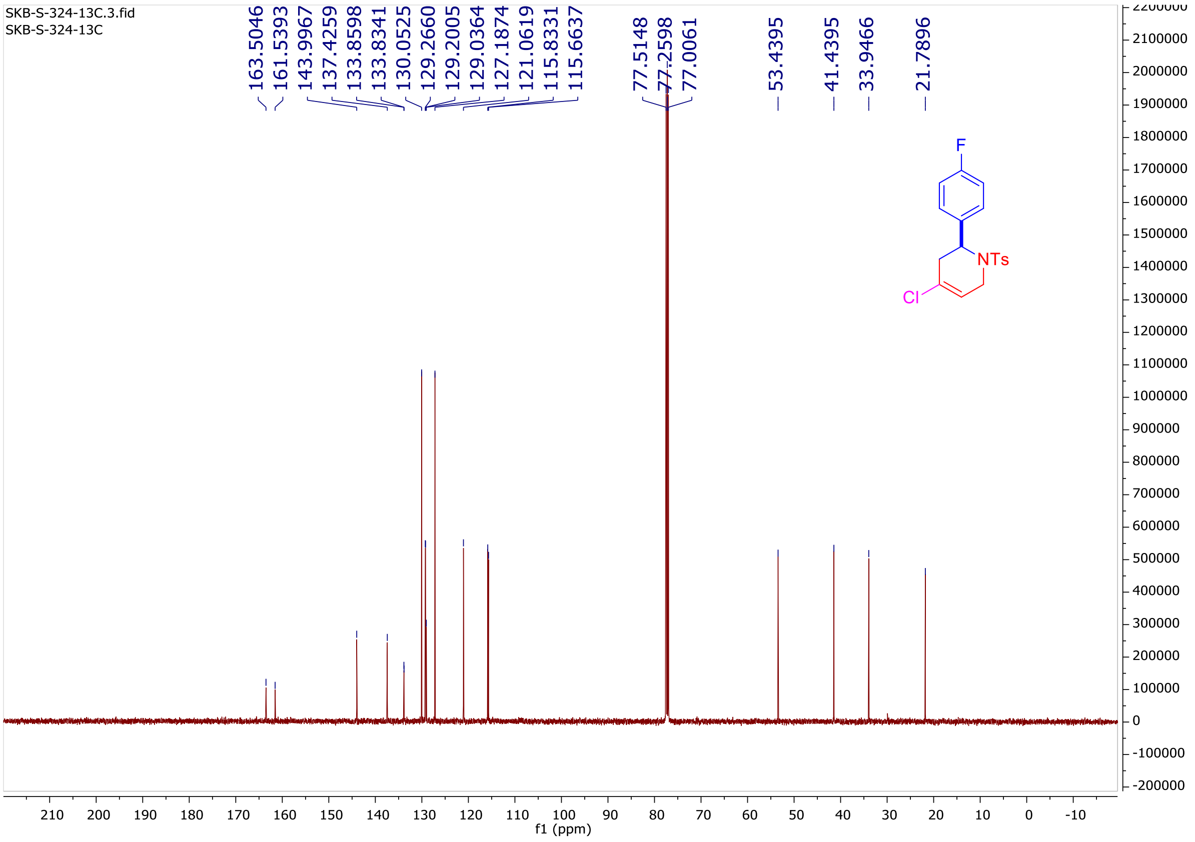

^{19}F (470 MHz, $C_6F_6/CDCl_3$) spectrum of 4db:

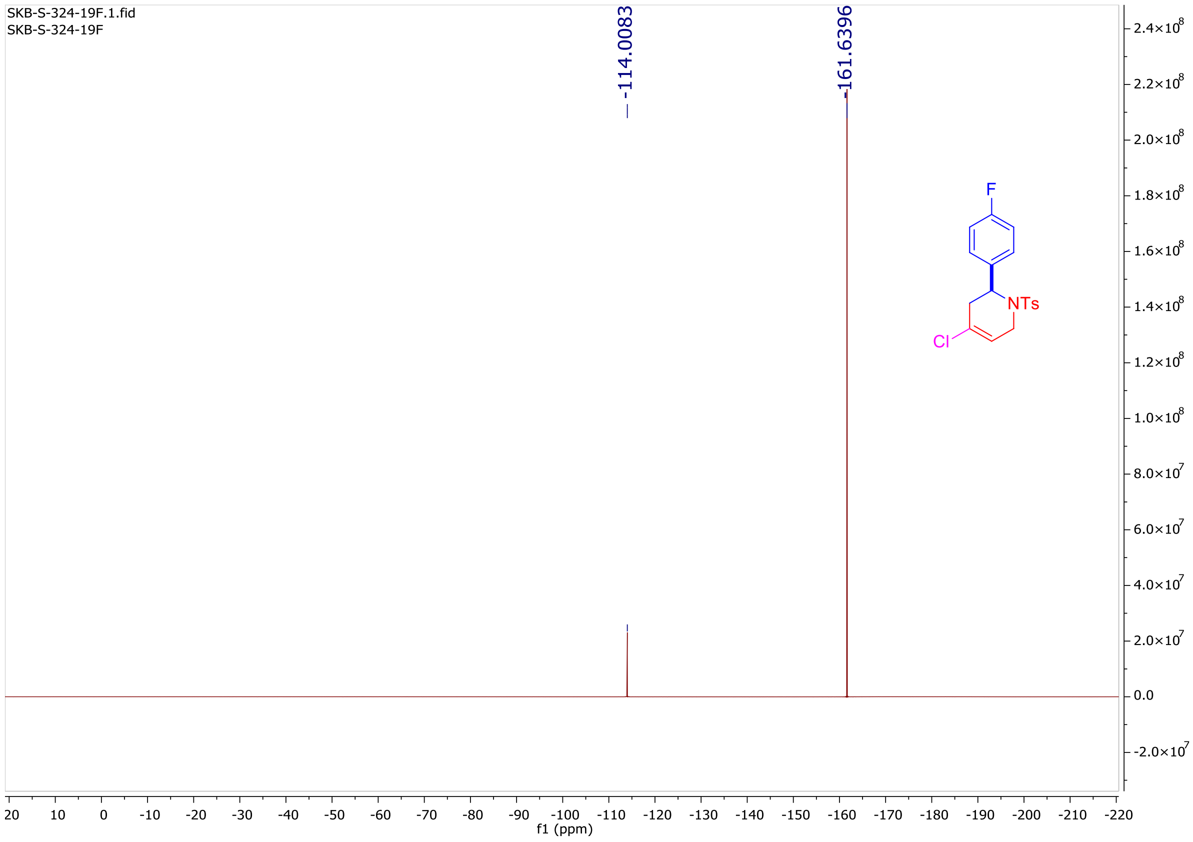

^{1}H (500 MHz, $CDCl_3$) and $^{13}C\{^{1}H\}$ (125 MHz, $CDCl_3$) spectra of 4de:

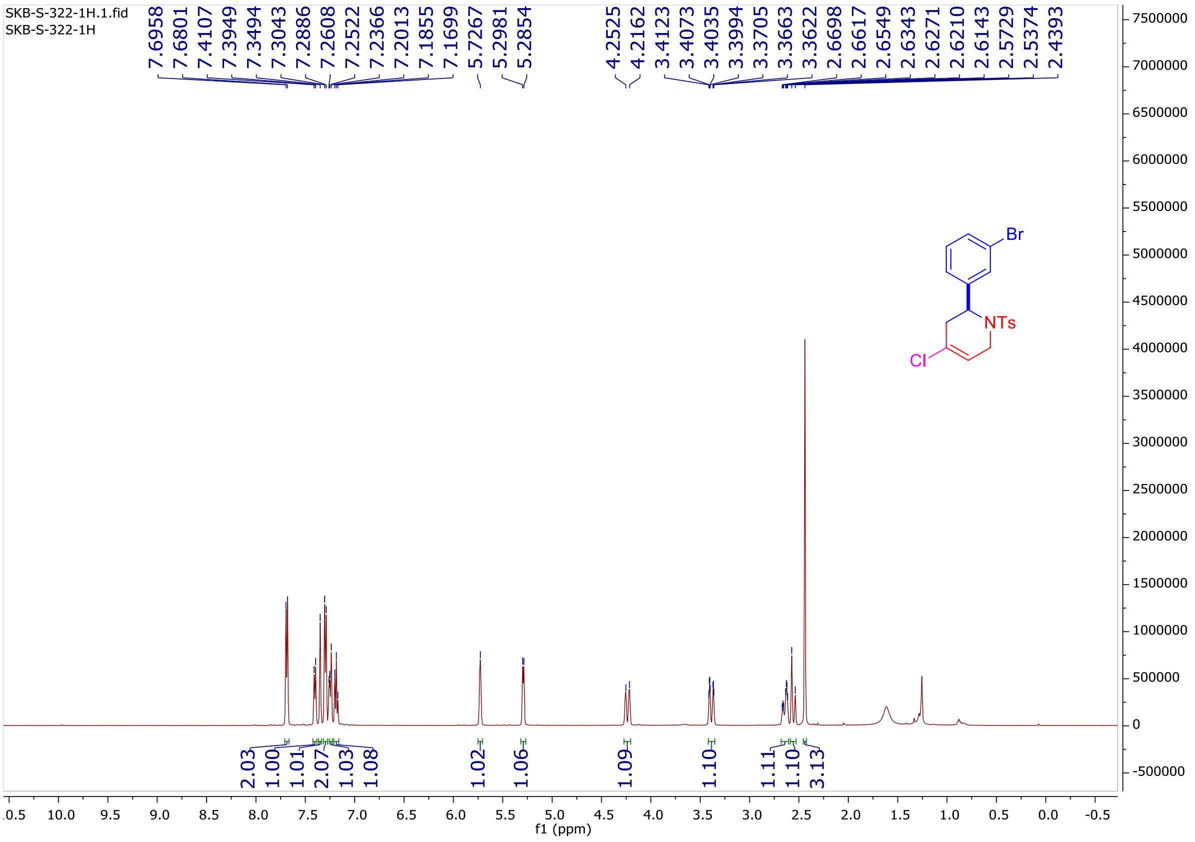

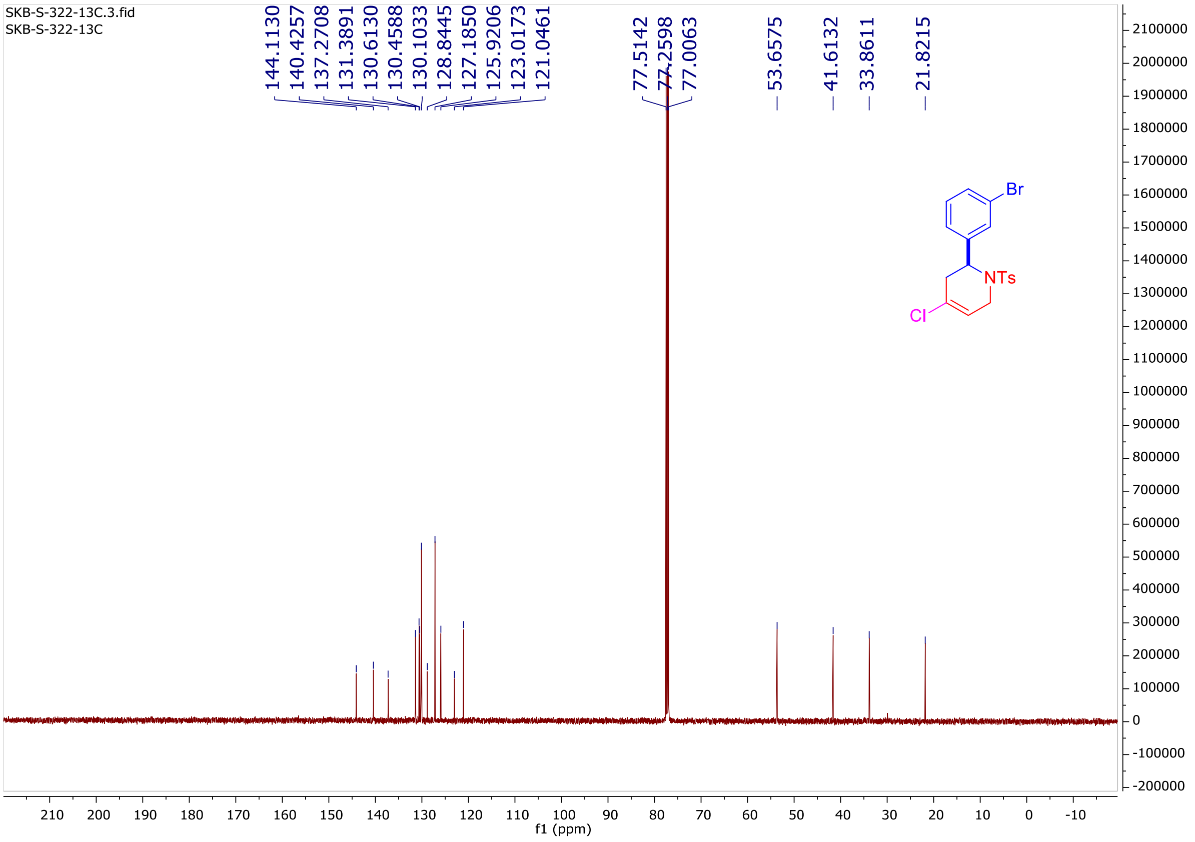

^{1}H (400 MHz, $CDCl_3$) and $^{13}C\{^{1}H\}$ (125 MHz, $CDCl_3$) spectra of 4dj:

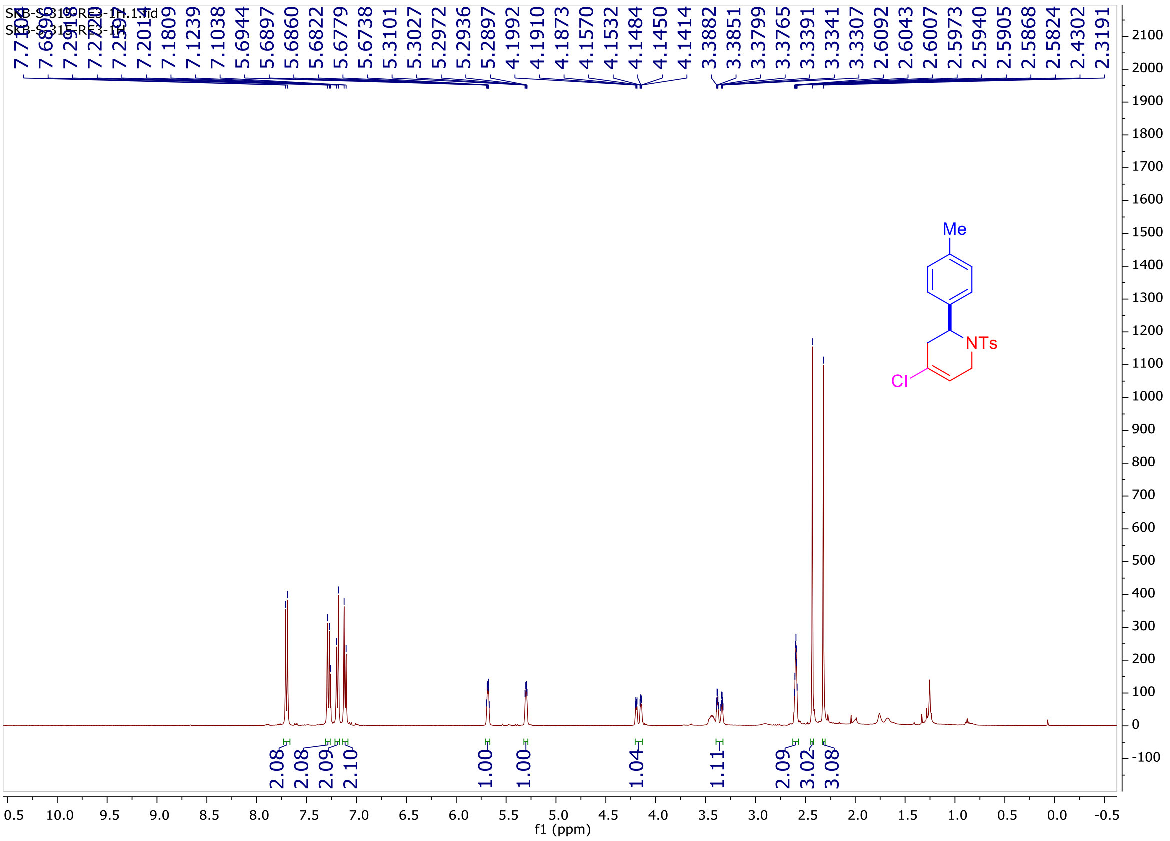

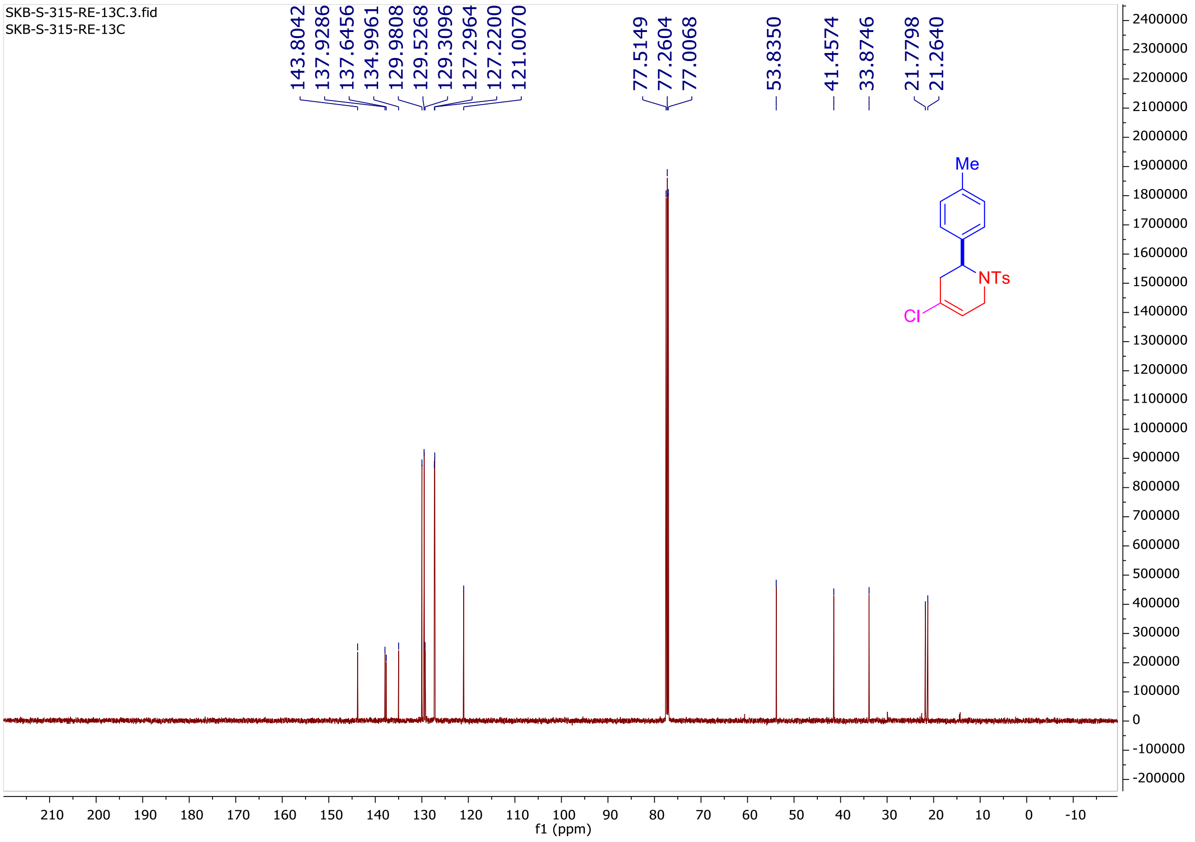

^{1}H (400 MHz, $CDCl_3$) and $^{13}C\{^{1}H\}$ (100 MHz, $CDCl_3$) spectra of 5a:

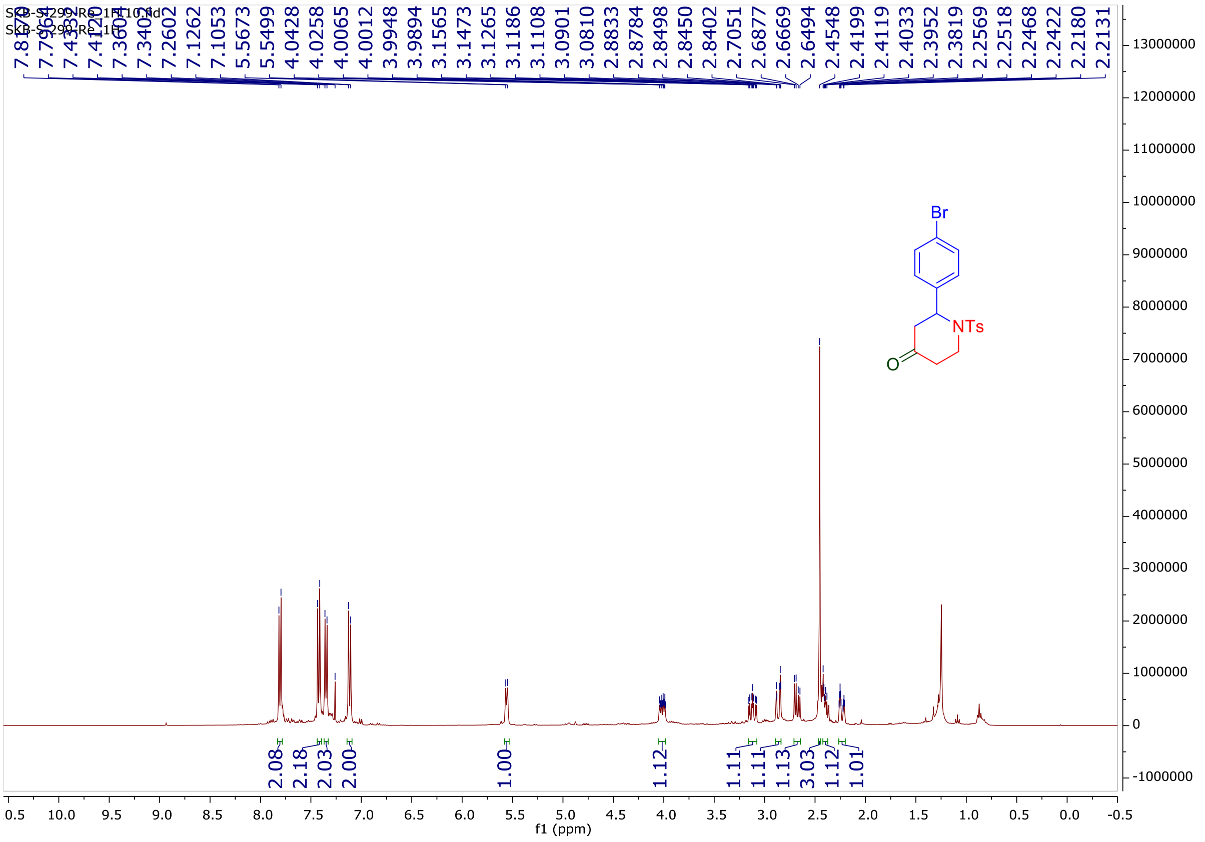

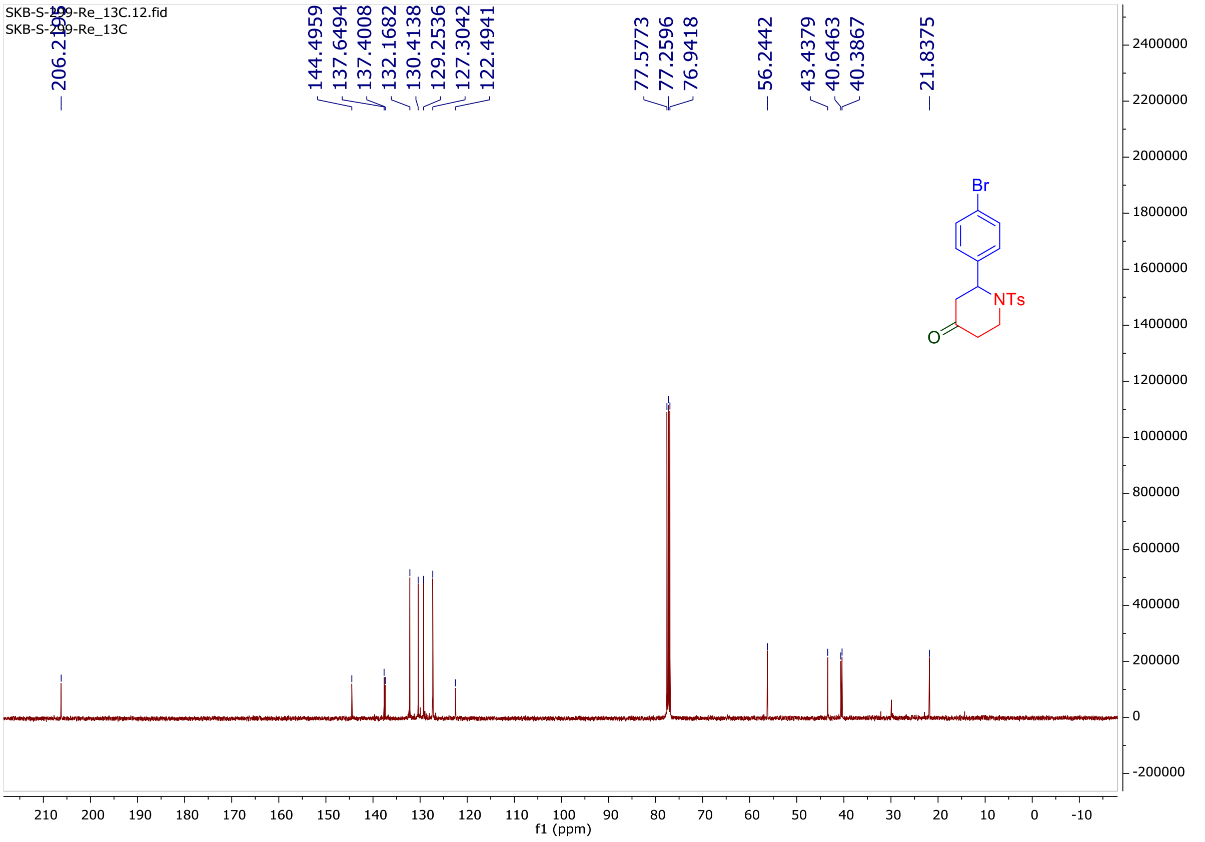

^{1}H (500 MHz, CDCl3) and ^{13}C{^{1}H} (125 MHz, CDCl3) spectra of 5b:

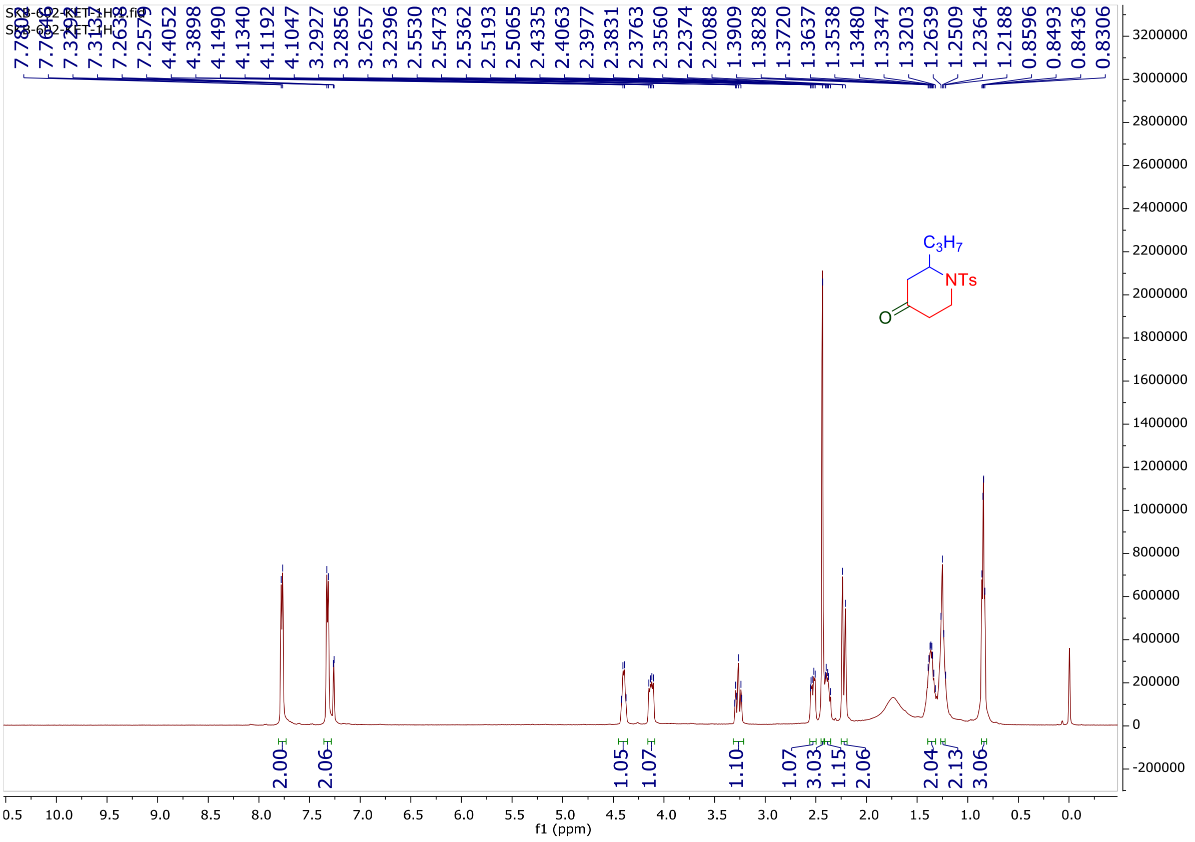

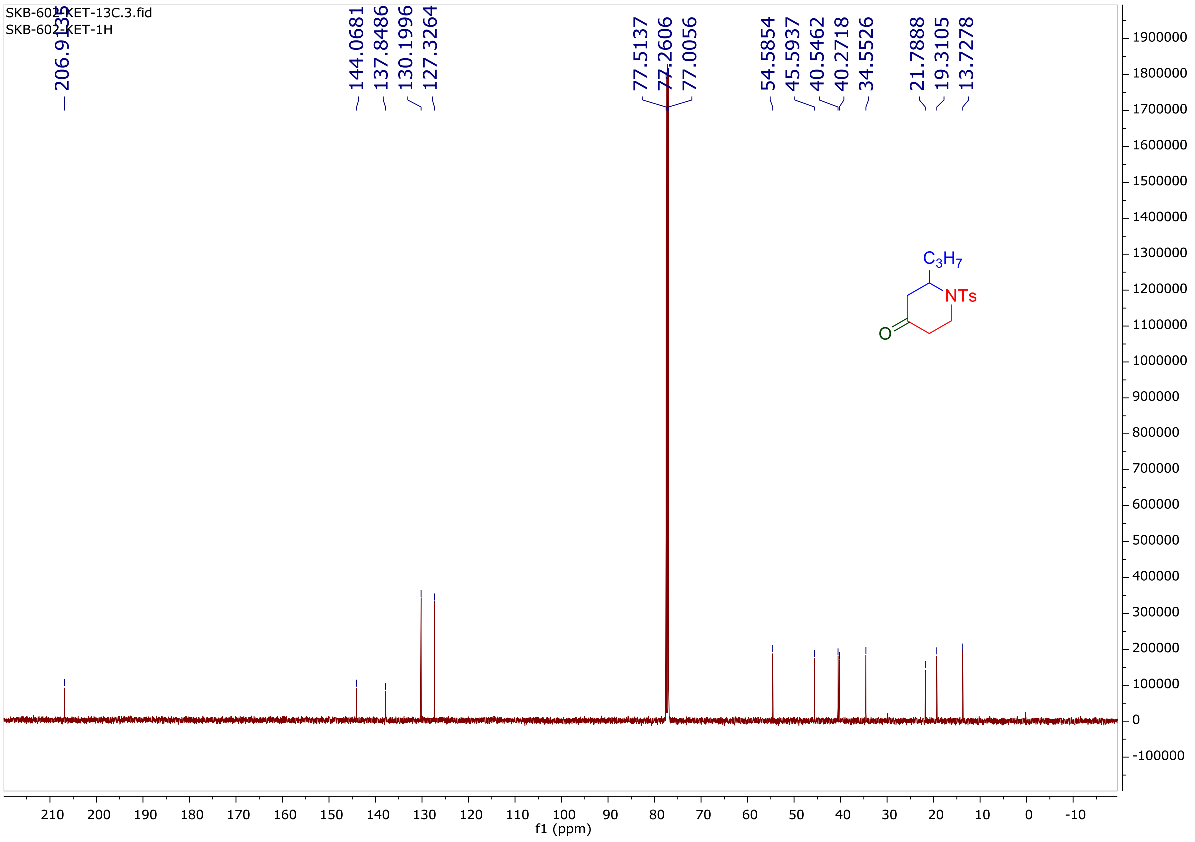

^{1}H (400 MHz, $CDCl_3$) and $^{13}C\{^{1}H\}$ (100 MHz, $CDCl_3$) spectra of 5c:

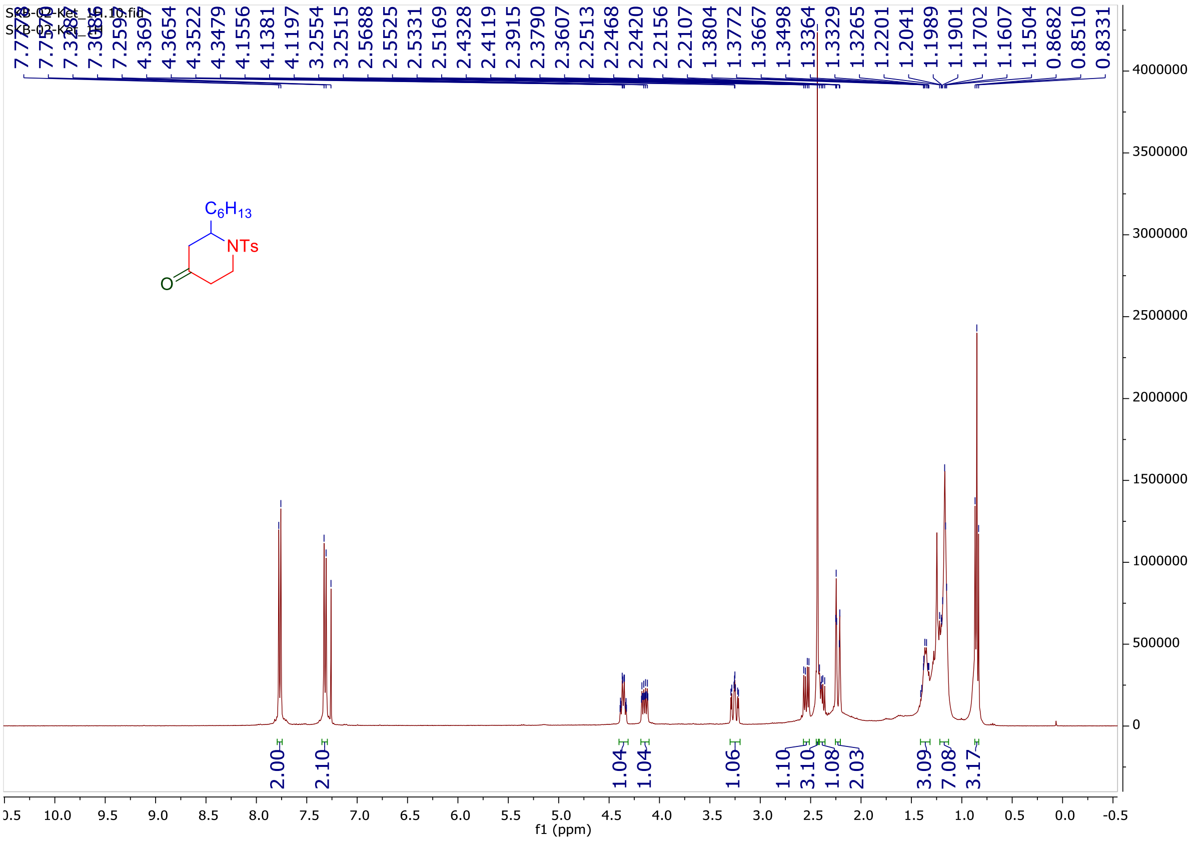

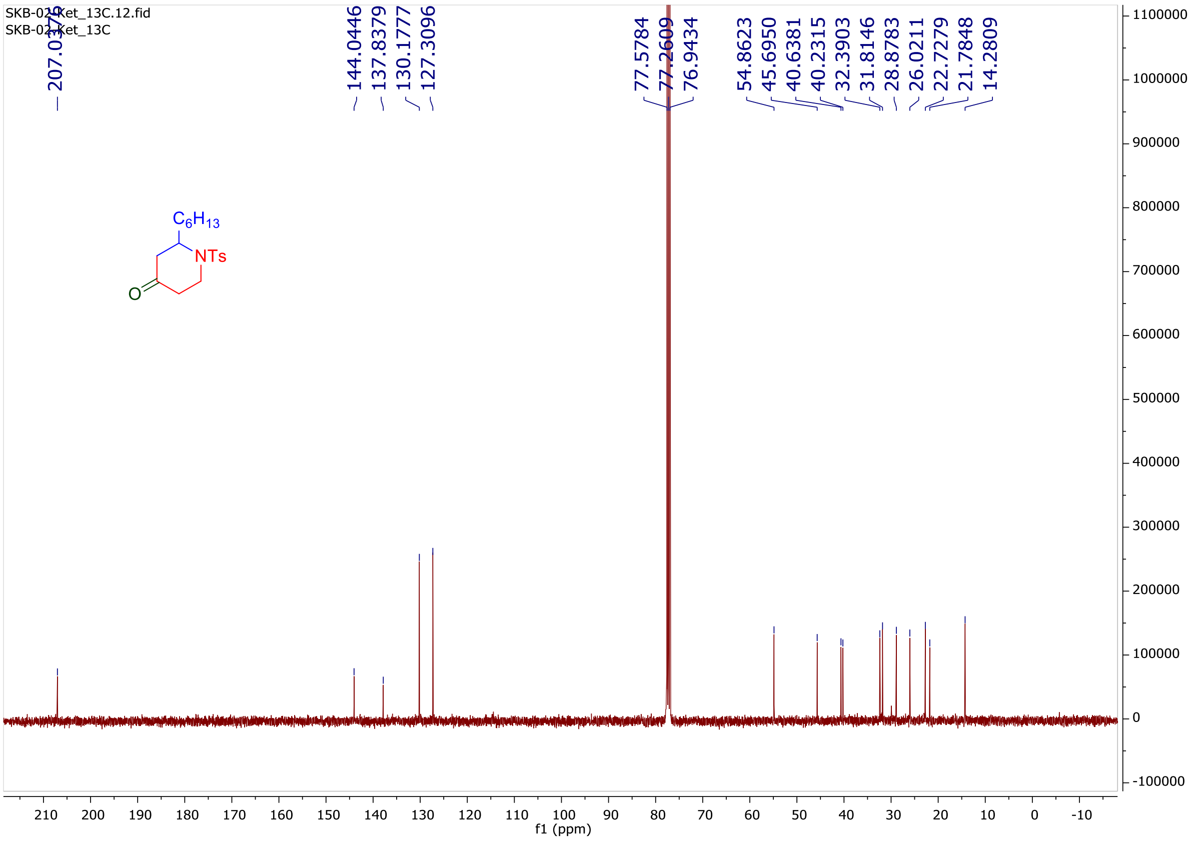

^{1}H (600 MHz, $CDCl_3$) and ^{13}C{^{1}H} (150 MHz, $CDCl_3$) spectra of 6a:

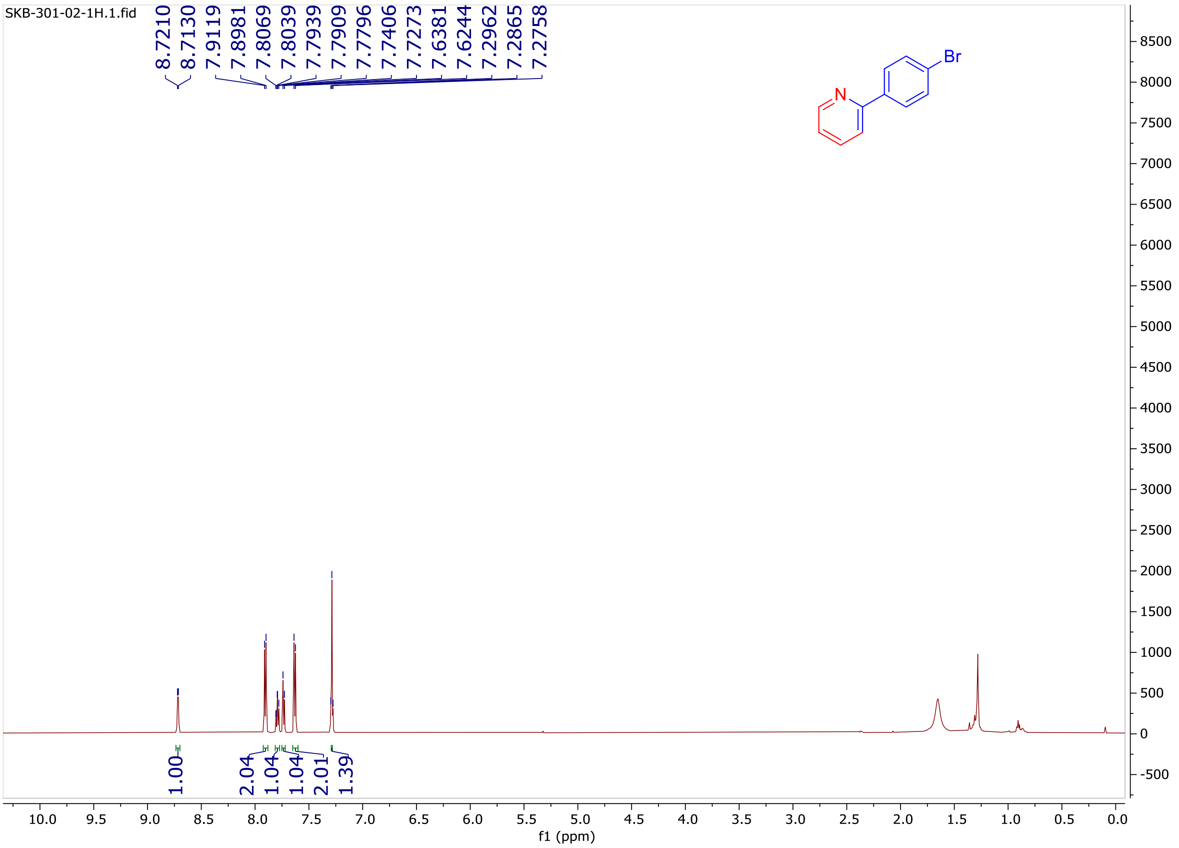

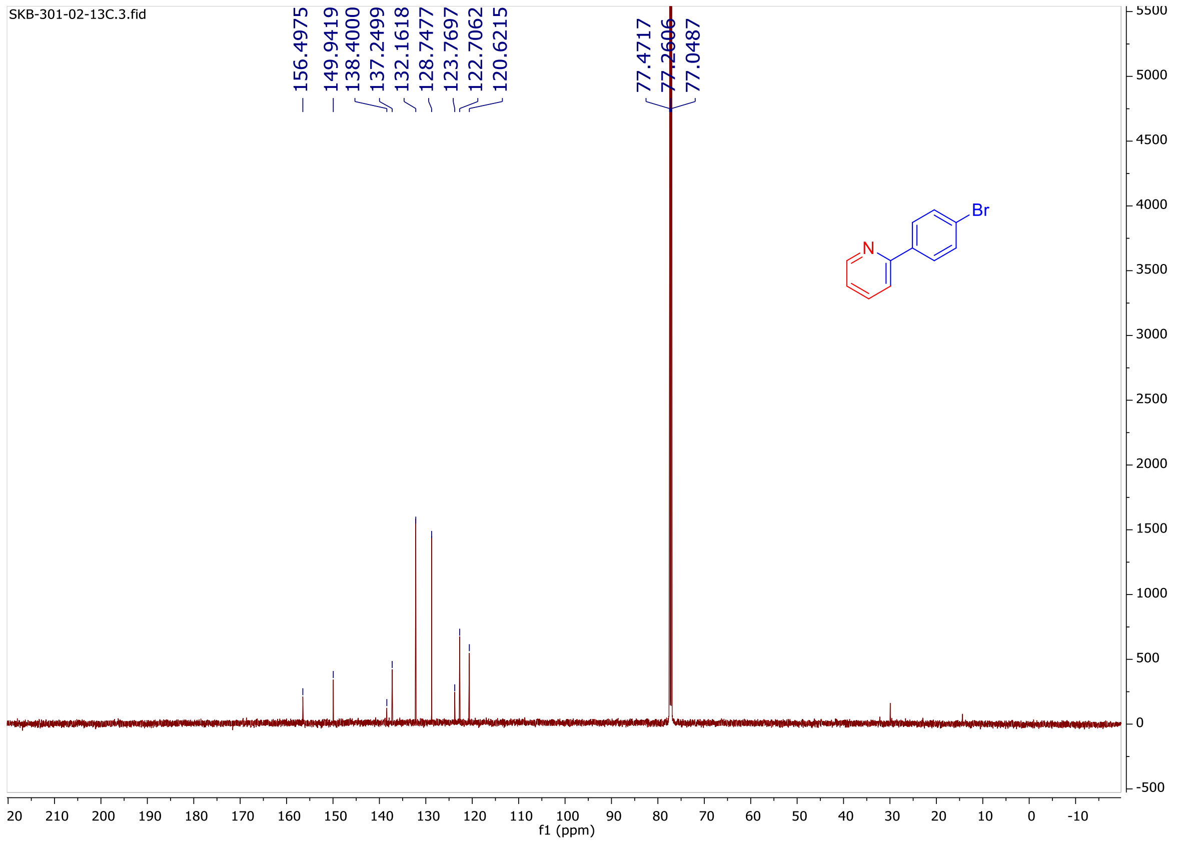

^{1}H (400 MHz, CDCl3) and ^{13}C{^{1}H} (100 MHz, CDCl3) spectra of 6b:

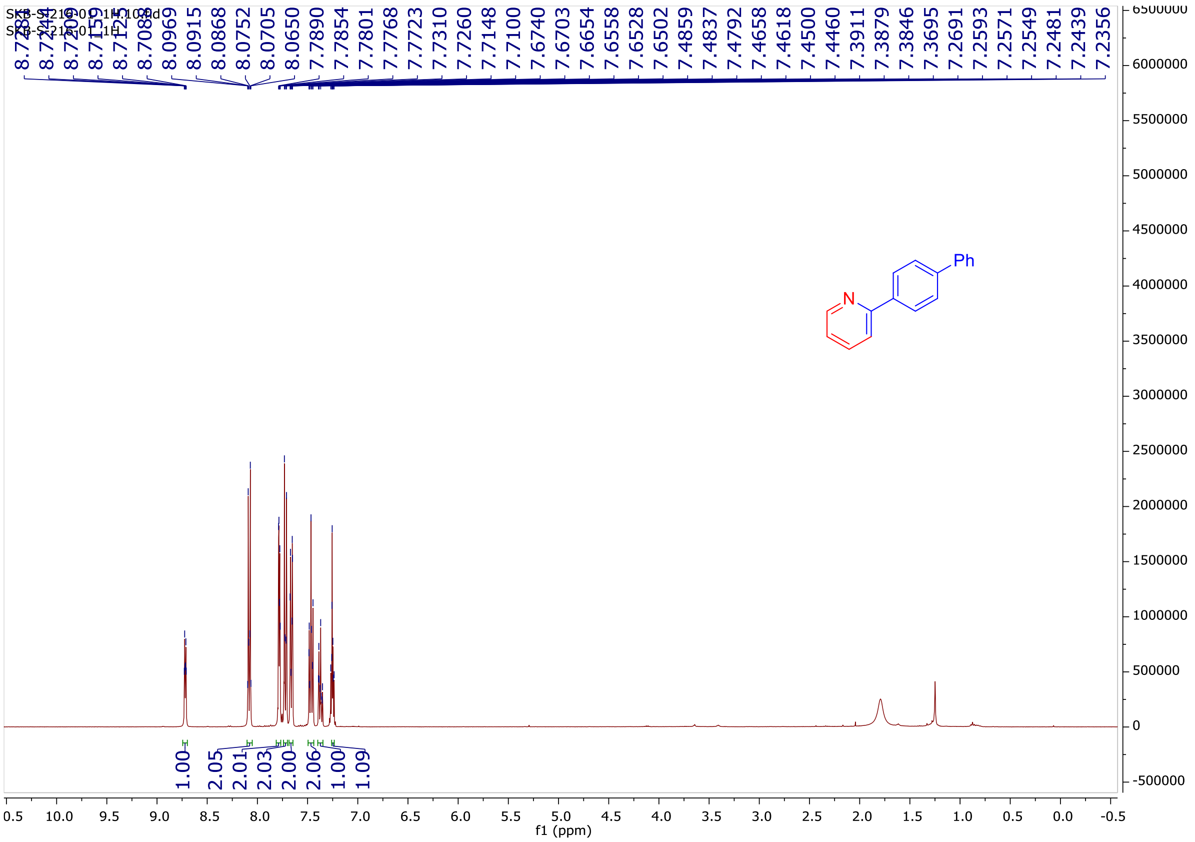

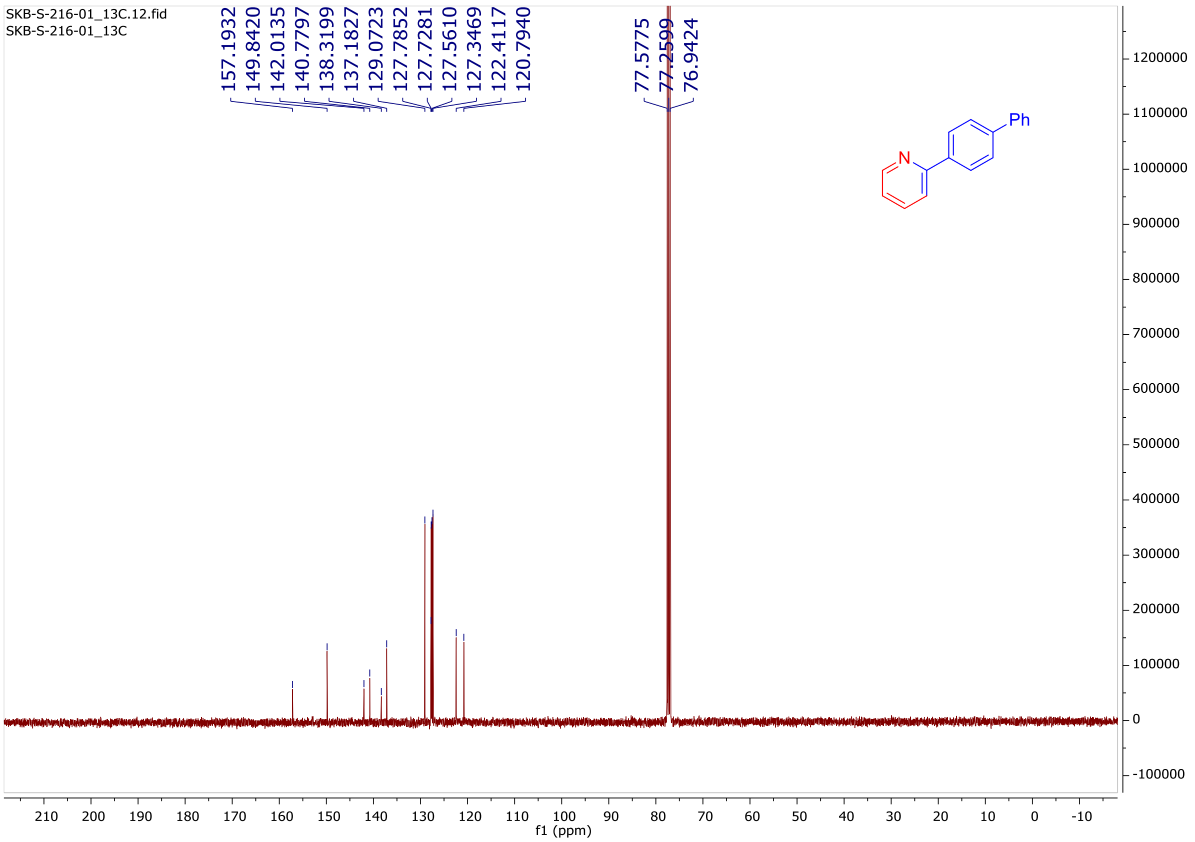

^{1}H (500 MHz, CDCl$_3$) and ^{13}C{^{1}H} (125 MHz, CDCl$_3$) spectra of 6c:

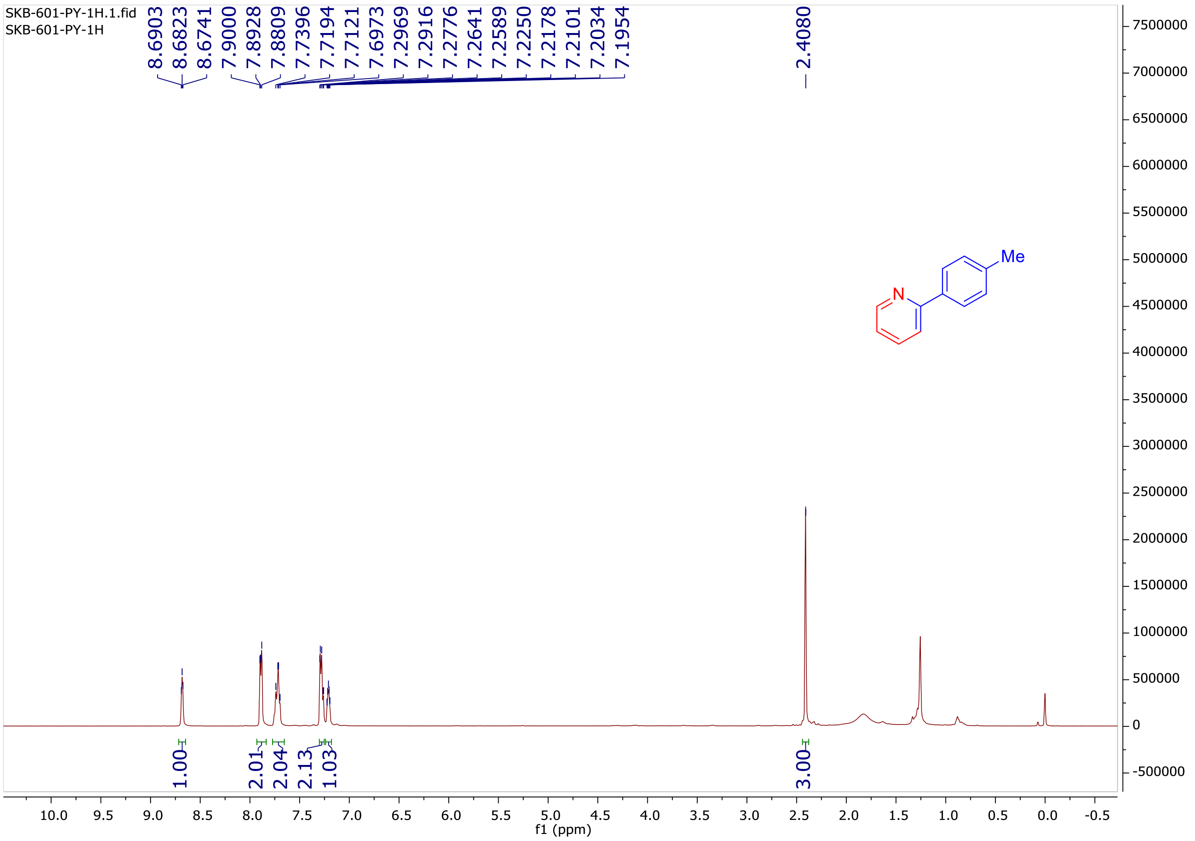

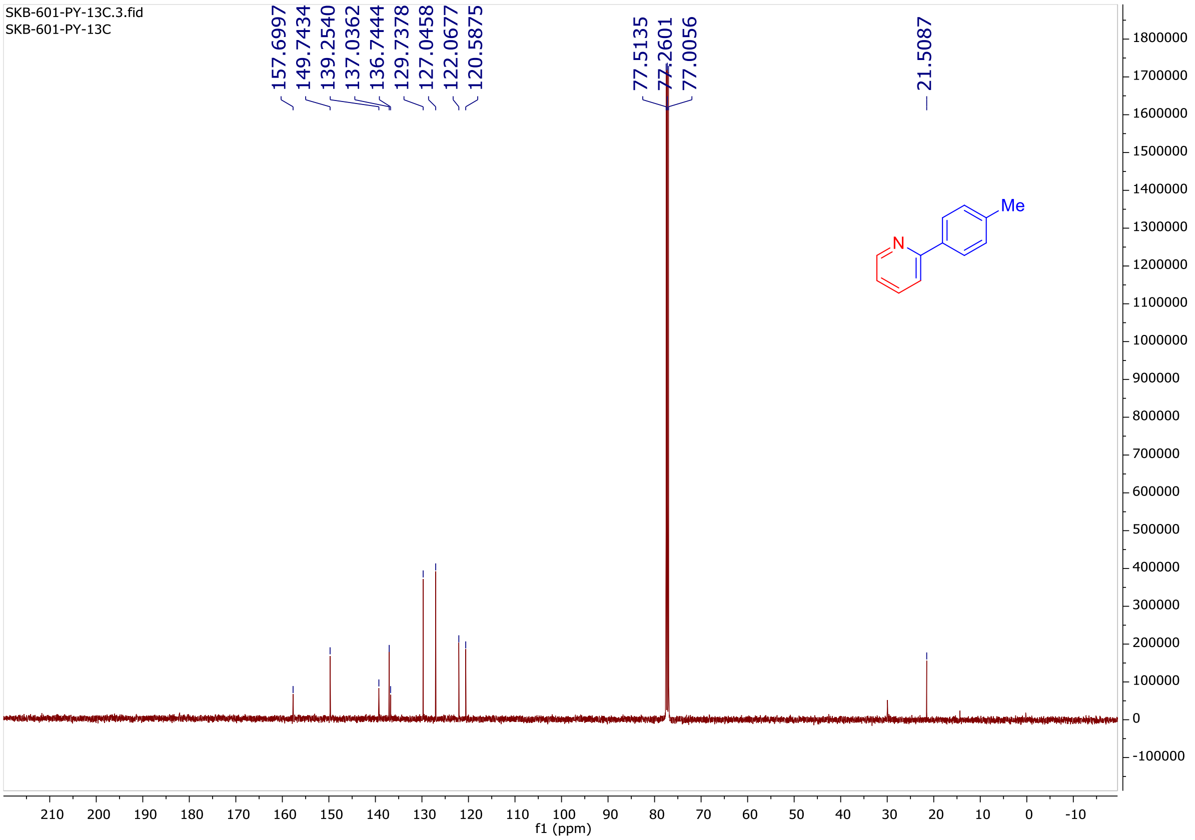

^{1}H (400 MHz, $CDCl_3$) and $^{13}C\{^{1}H\}$ (150 MHz, $CDCl_3$) spectra of 7:

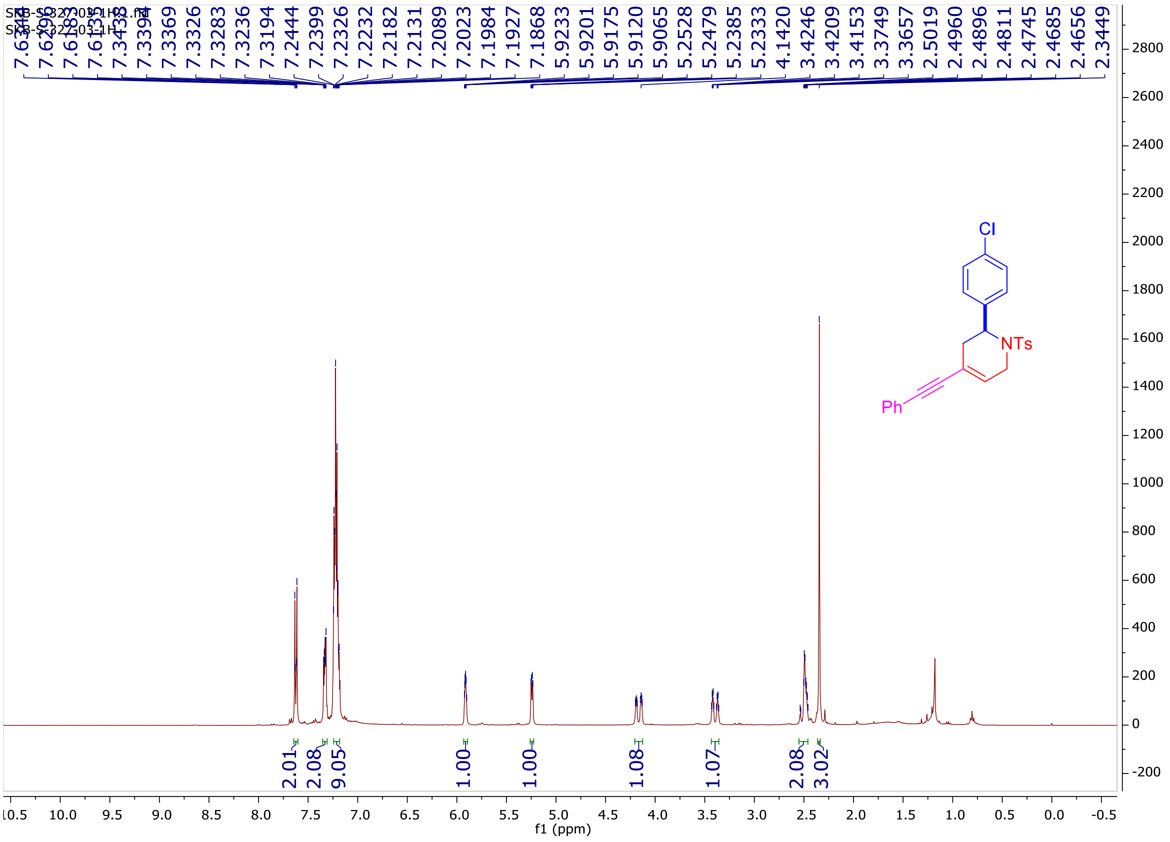

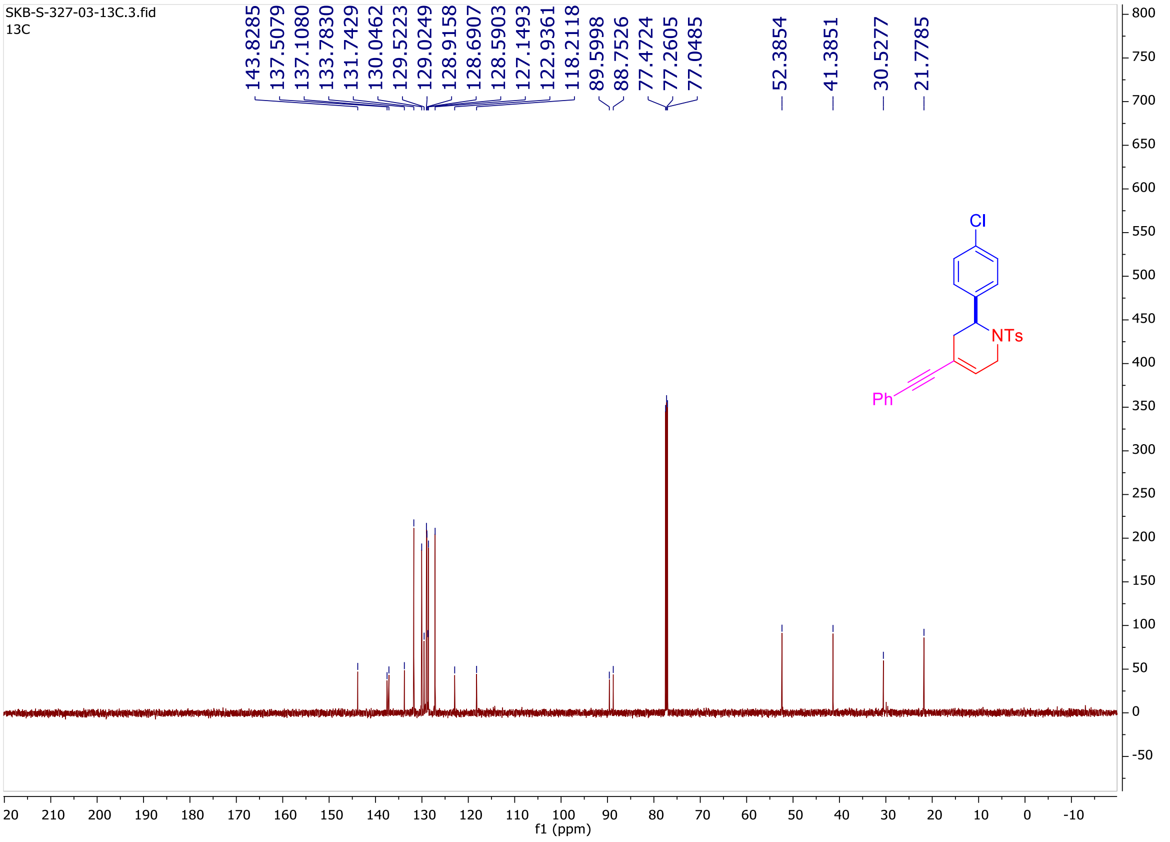

Single crystal X-ray diffraction:

Single crystals of compound **3ac, 3dj** and **4aa** were obtained by slow evaporation of hexane and ethyl acetate solution (9:1). The Bruker SMART APEX-II CCD diffractometer was used to collect the intensity data. The instrument is equipped with a fine focus 1.75 kW sealed tube Mo Kα radiation (λ = 0.71073 Å) at 293(3) K, with increasing ω (width of 0.3° per frame) at a scan speed of 3 s/frame. The data acquisition was done with the SMART software. The SAINT and XPREP software were implemented for data integration and reduction.[1] Multiscan empirical absorption corrections were employed to the data using the program SADABS.[2] Structures were solved by direct methods using SHELXS- 2016 and refined with full-matrix least-squares on F2 using SHELXL- 2016/6.[3] Structural illustrations have been drawn with ORTEP-3 for Windows.[4] The detailed data collection and structure refinement are summarized in Table 1-3. CCDC- 2429152 (for **3ac**), 2431627 (for **3dj**) and CCDC- 2429151 (for **4aa**) contained supplementary crystallographic data for this paper.

Ref. 1) SMART; SAINT; XPREP; Siemens Analytical X-ray Instruments Inc.: Madison, WI, 1995.

2) G. M. Sheldrick, SADABS: Software for Empirical Absorption Correction University of Gottingen, Institut fur Anorganiche Chemieder Universitat: Gottingen, Germany, 1999.

3) G. M. Sheldrick, SHELXS-2014, Program for the crystal structure solution; University of Göttingen: Göttingen, Germany, 2014.

4) L. J. Farrugia, XRDIFF: simulation of X-ray diffraction patterns, *J. Appl. Crystallogr*. 1997, **30**, 565.

Table S1: The crystal parameters of compound **3ac**

	CCDC 2429152
Formula	$C_{18}H_{18}Br_2ClNO_2S$
Formula weight	507.66
T/K	293(2)
Crystal system	monoclinic
Space group	P21/c
• *a*/Å	11.4025(12)
• *b*/Å	15.3012(15)
• *c*/Å	11.8426(12)
• *α*/°	90
• *β*/°	112.487(4)
• *γ*/°	90
• *V*/$Å^3$	1909.1(3)
• Z	4
Abs. Coeff./mm^{-1}	4.507
Abs. Correction	‘none’
GOF on F^2	1.058
Final *R* indices [*I*> 2σ(I)]	R_1 = 0.0299<br>*wR2* = 0.0688
R indices [all data]	*R1* = 0.0406<br>*wR2* = 0.0726

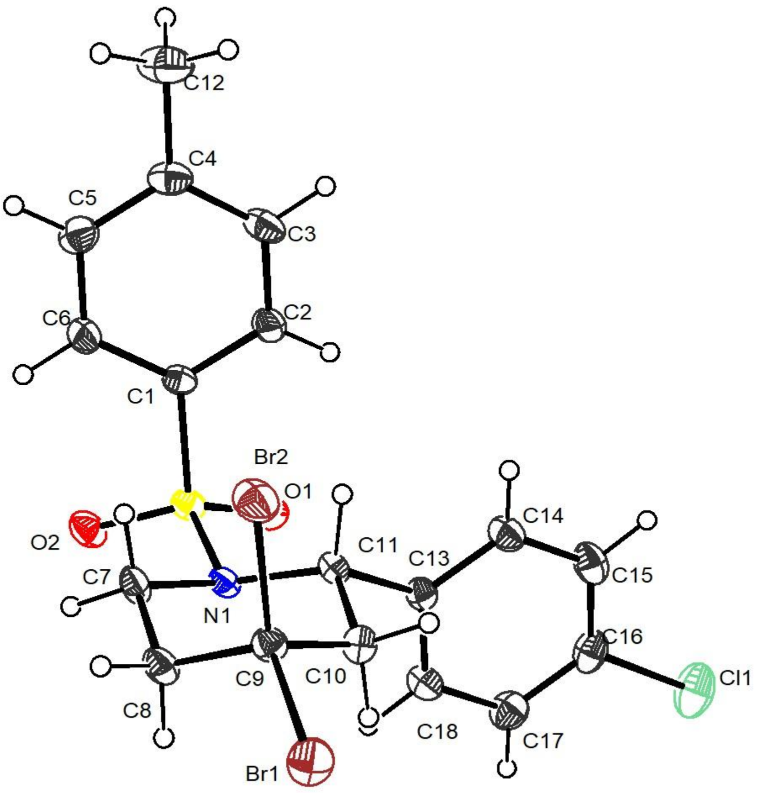

Figure S1: ORTEP diagram of compound 3ac with 30% probability:

Table S2: The crystal parameters of compound **3dj**

	CCDC 2431627
Formula	$C_{19}H_{21}BrClNO_2S$
Formula weight	442.79
T/K	295.00
Crystal system	monoclinic
Space group	Cc
• *a*/Å	20.319(4)
• *b*/Å	10.440(2)
• *c*/Å	9.3782(19)
• *α*/°	90
• *β*/°	100.424(6)
• *γ*/°	90
• *V*/Å^3	1956.6(7)
• Z	4
Abs. Coeff./mm^{-1}	2.356
Abs. Correction	‘none’
GOF on F^2	1.025
Final *R* indices [I> 2σ(I)]	R_1 = 0.0534<br>*wR2* = 0.1373
R indices [all data]	*R1* = 0.0649<br>*wR2* = 0.1460

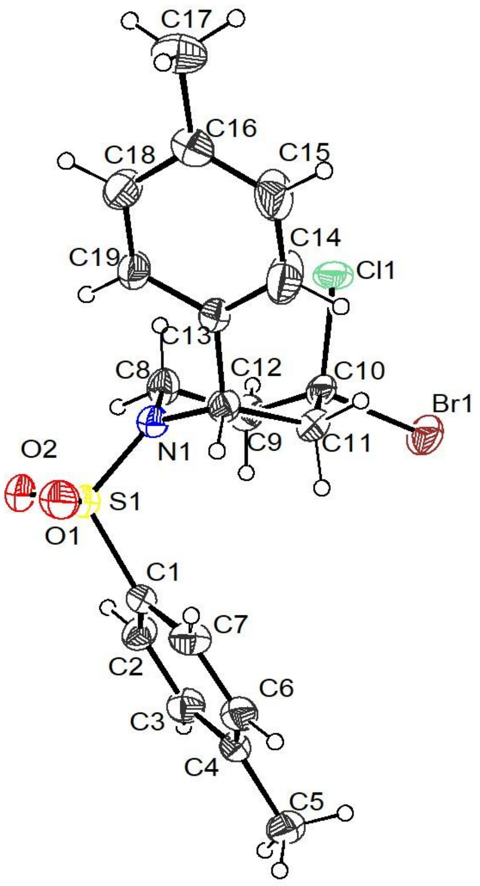

Figure S2: ORTEP diagram of compound 3dj with 30% probability:

Table S3: The crystal parameters of compound **4aa**

	CCDC 2429151
Formula	$C_{18}H_{18}BrNO_2S$
Formula weight	392.30
T/K	295(2)
Crystal system	monoclinic
Space group	P21/c
• *a*/Å	11.378(2)
• *b*/Å	8.0513(15)
• *c*/Å	19.674(4)
• *α*/°	90
• *β*/°	106.635(5)
• *γ*/°	90
• *V*/Å^3	1726.9(5)
• Z	4
Abs. Coeff./mm^{-1}	2.510
Abs. Correction	'none'
GOF on F^2	1.023
Final *R* indices [$I > 2\sigma(I)$]	R_1 = 0.0493<br>*wR2* = 0.1183
R indices [all data]	*R1* = 0.0863<br>*wR2* = 0.1373

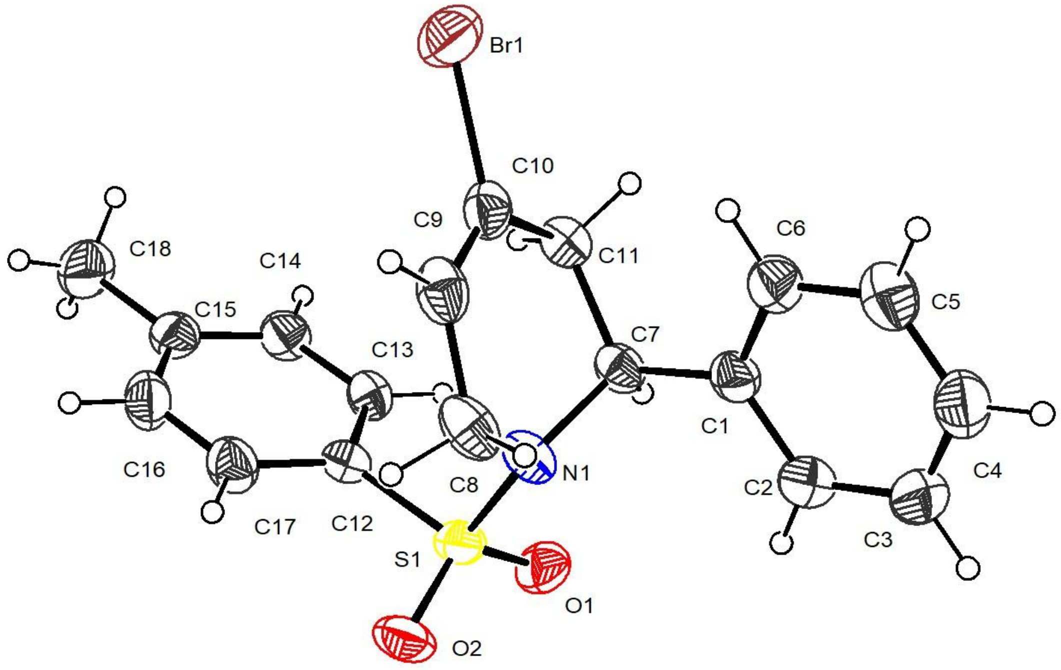

Figure S3: ORTEP diagram of compound 4aa with 30% probability
