# Supplementary material for: Selective synthesis of gem-dihalopiperidines and 4-halo-1,2,3,6-tetrahydropyridines from halogen substituted homoallylic benzenesulfonamides and aldehydes
Source: RSC Adv. 2025 Jun 23;15(27):21257–68. doi: 10.1039/d5ra03630e (PMC12184090; doi:10.1039/d5ra03630e)

Supplementary Information (SI) for RSC Advances.
This journal is © The Royal Society of Chemistry 2025

Selective Synthesis of gem-Dihalopiperidines and 4-halo-1,2,3,6-tetrahydropyridines from halogen substituted homoallylic benzenesulfonamide and aldehyde

Surjya Kumar Bora and Anil K. Saikia*

Department of Chemistry, Indian Institute of Technology Guwahati, Guwahati 781039, India

E-mail: asaikia@iitg.ac.in

Table of Contents:

1. HRMS spectra of all new compounds **S2-S36**

HRMS spectrum of **3aa**

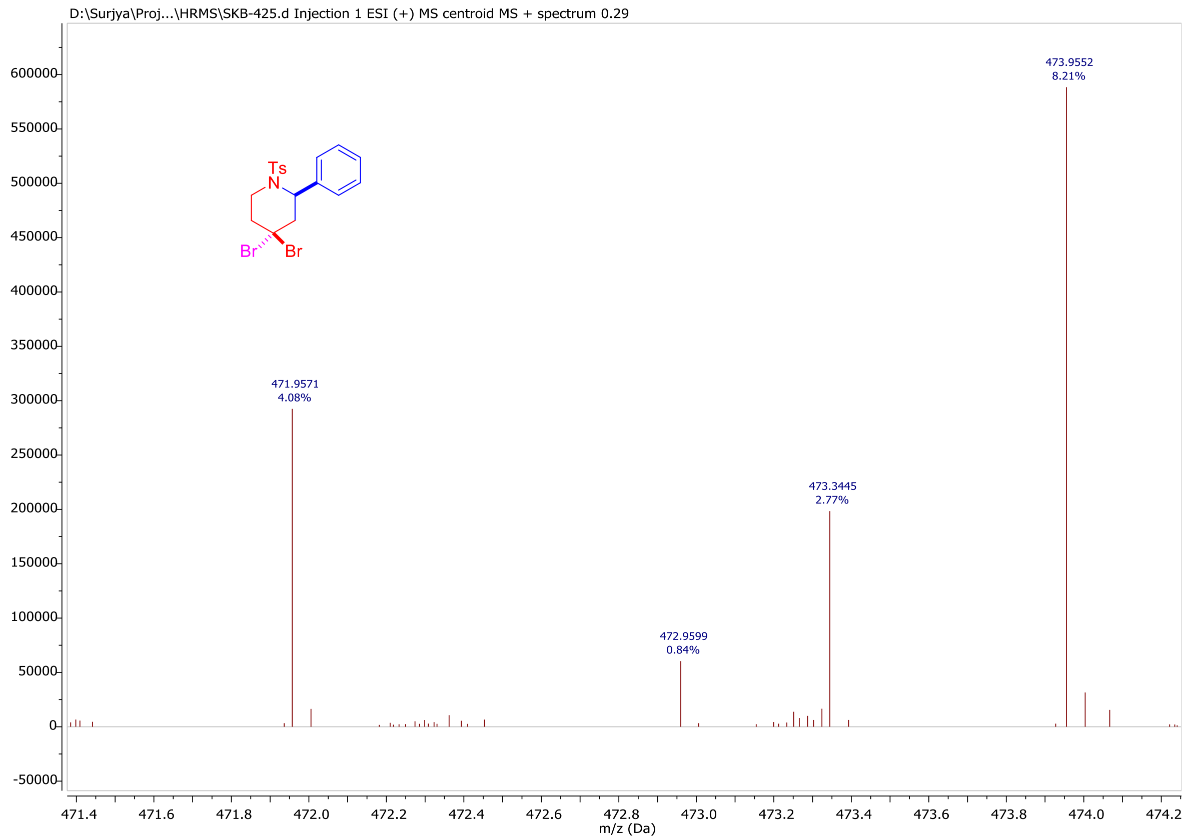

HRMS spectrum of **3ab**

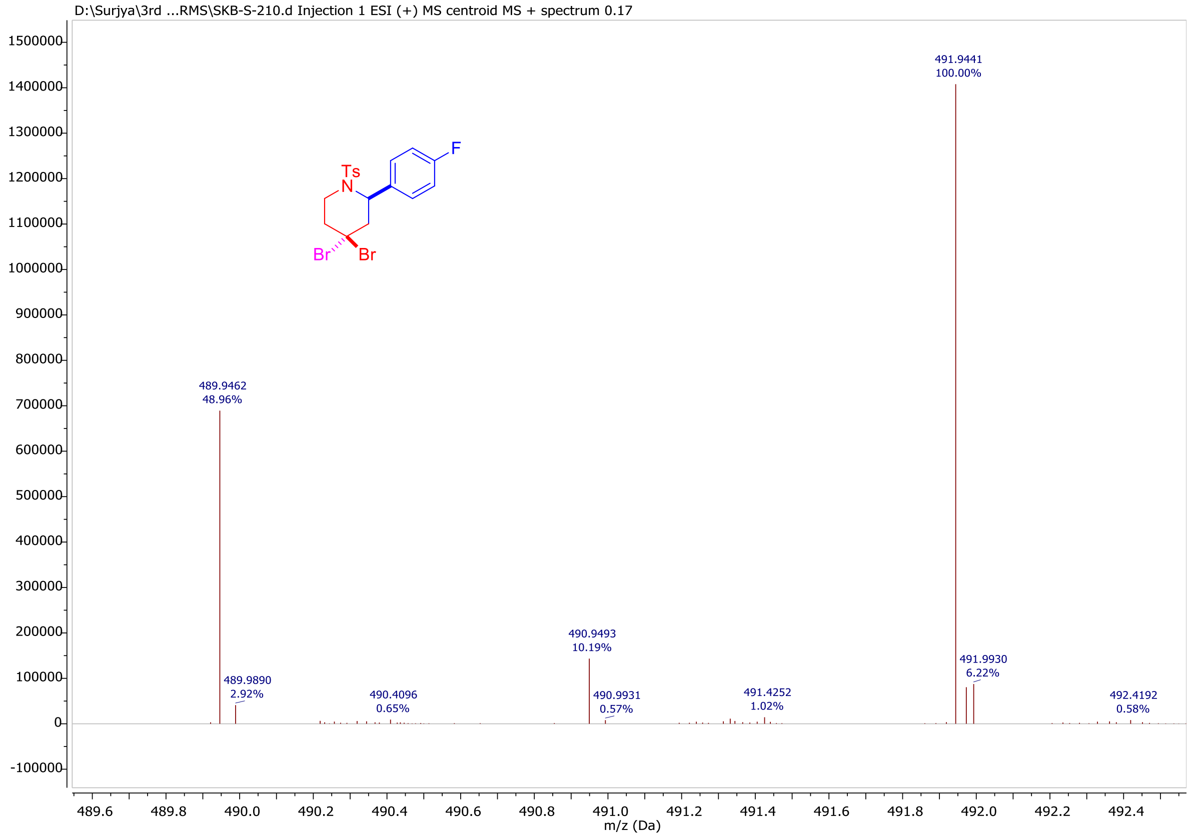

HRMS spectrum of **3ac**

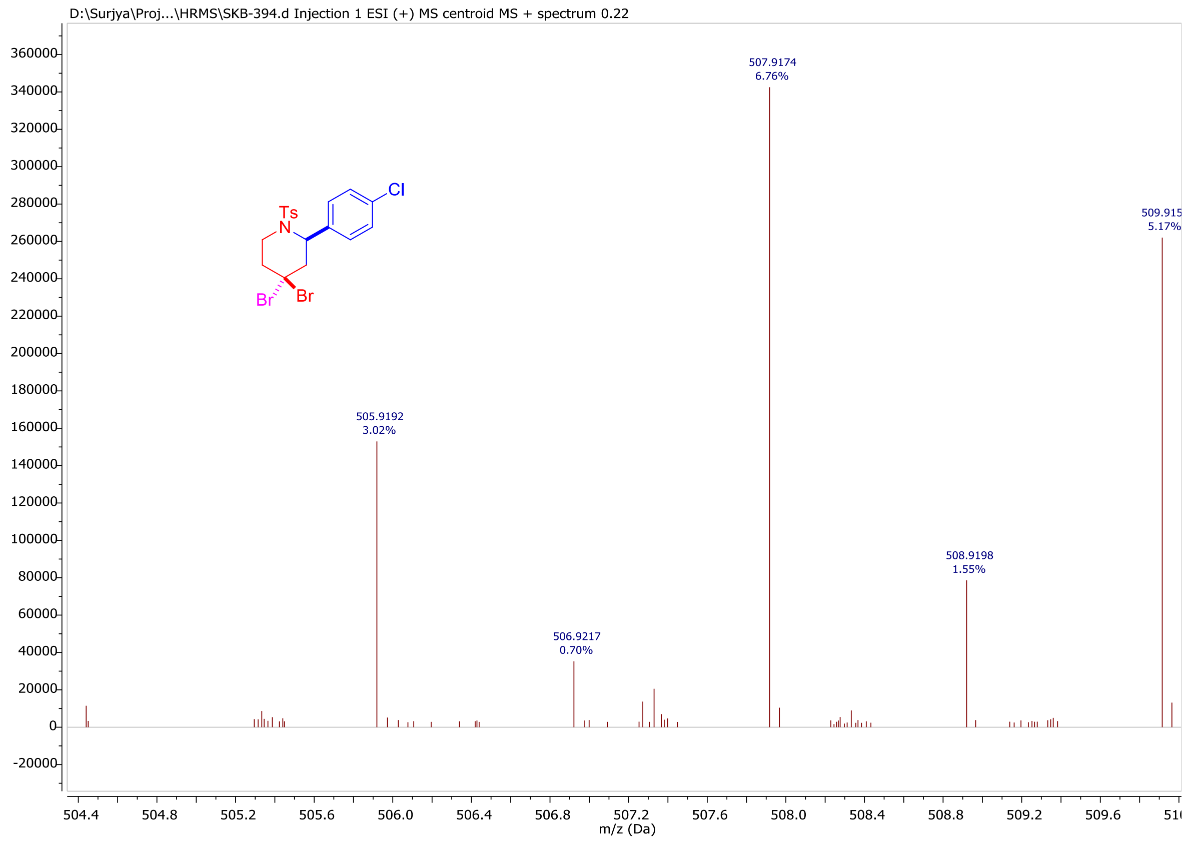

HRMS spectrum of **3ad**

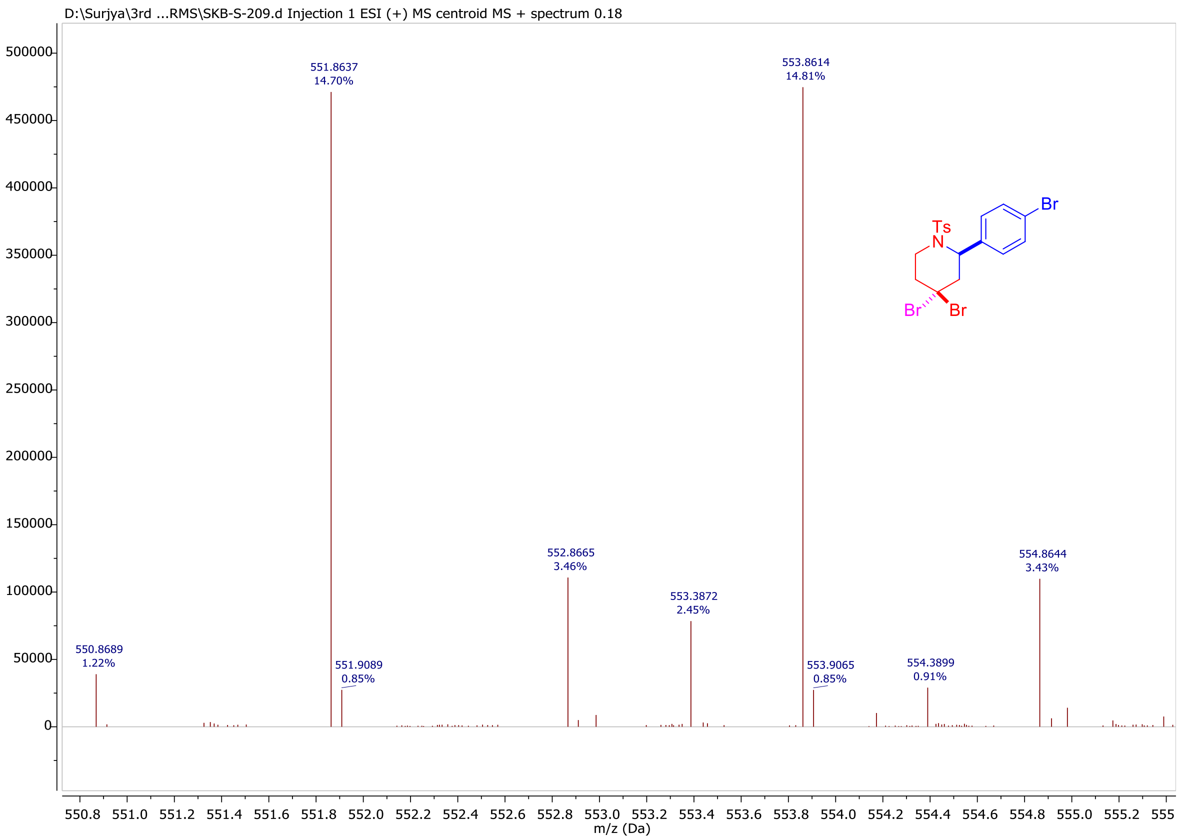

HRMS spectrum of **3ae**

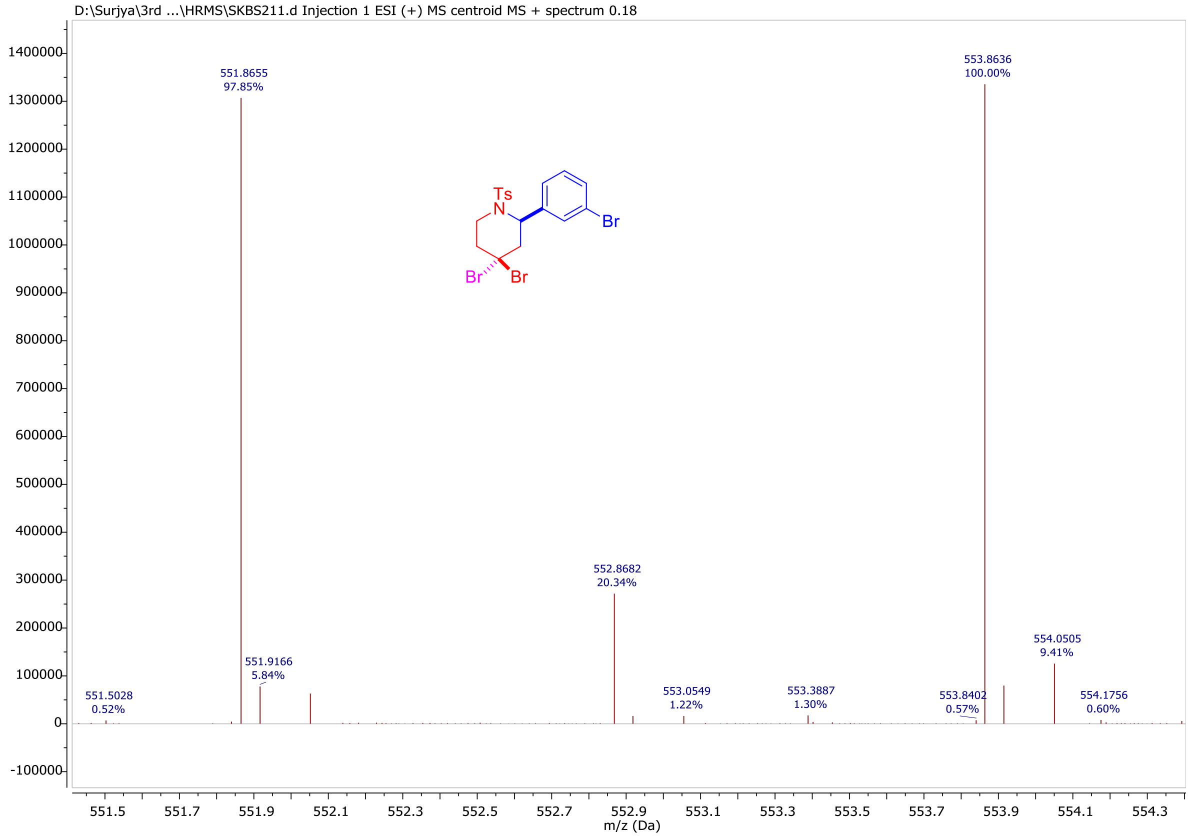

HRMS spectrum of **3af**

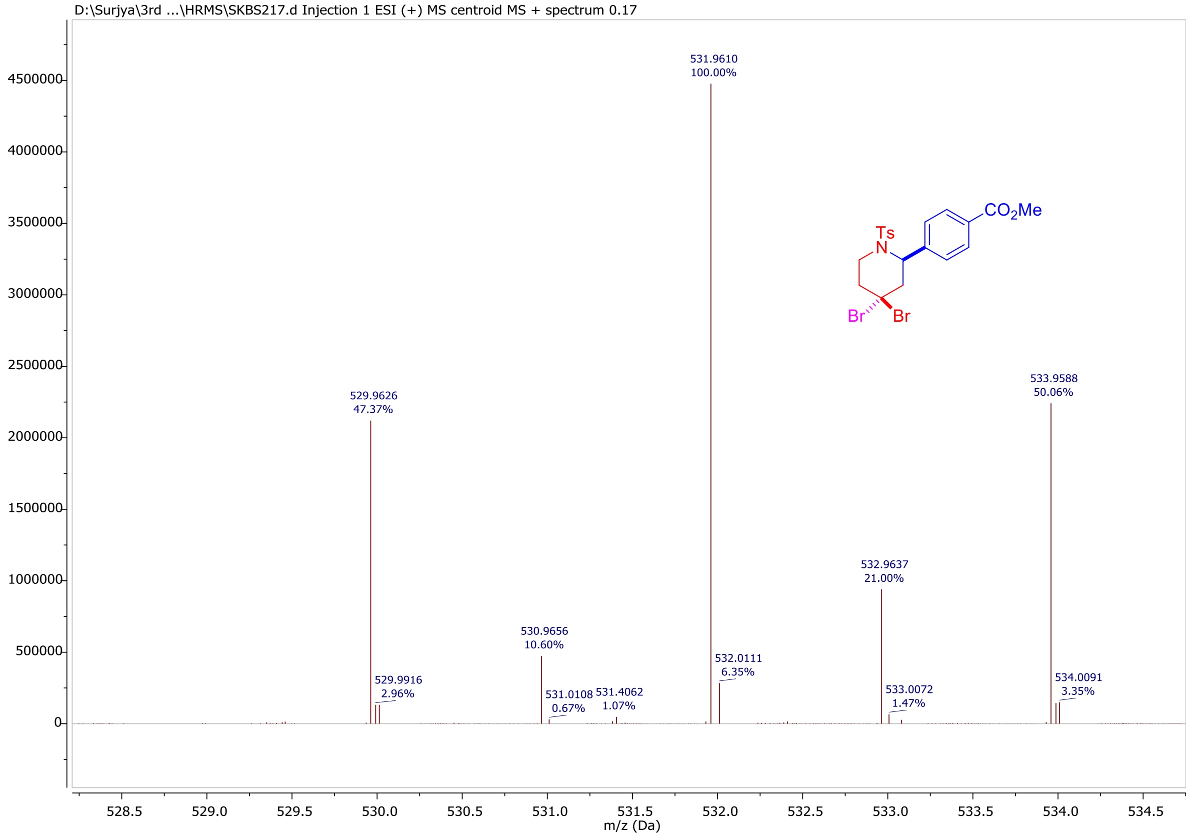

HRMS spectrum of **3ag**

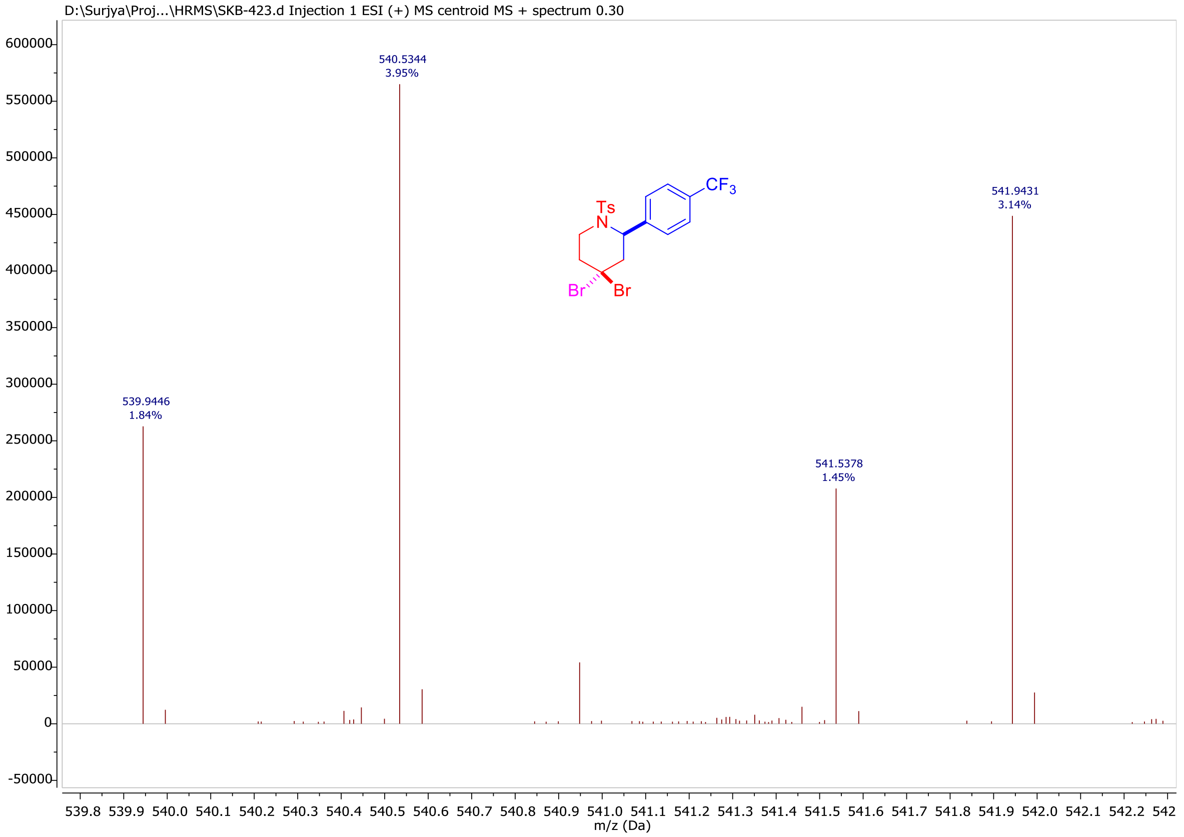

HRMS spectrum of **3ah**

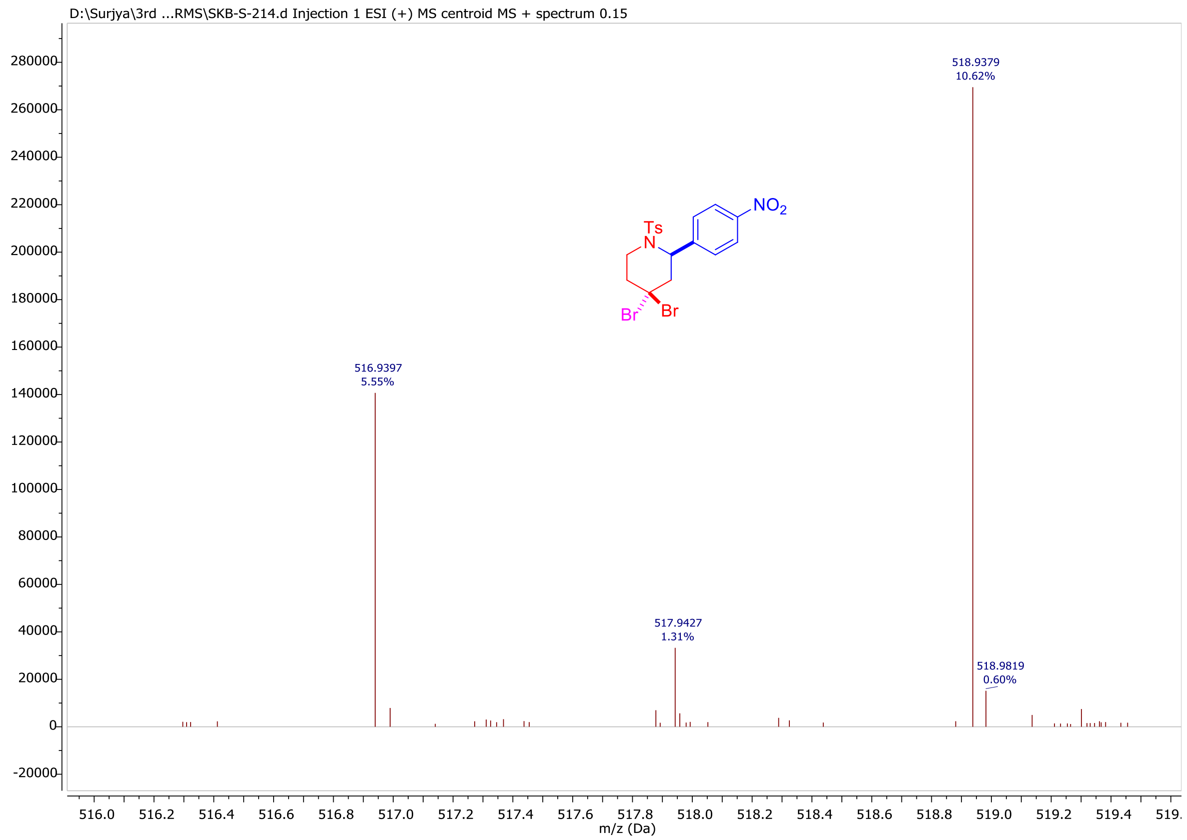

HRMS spectrum of **3ai**

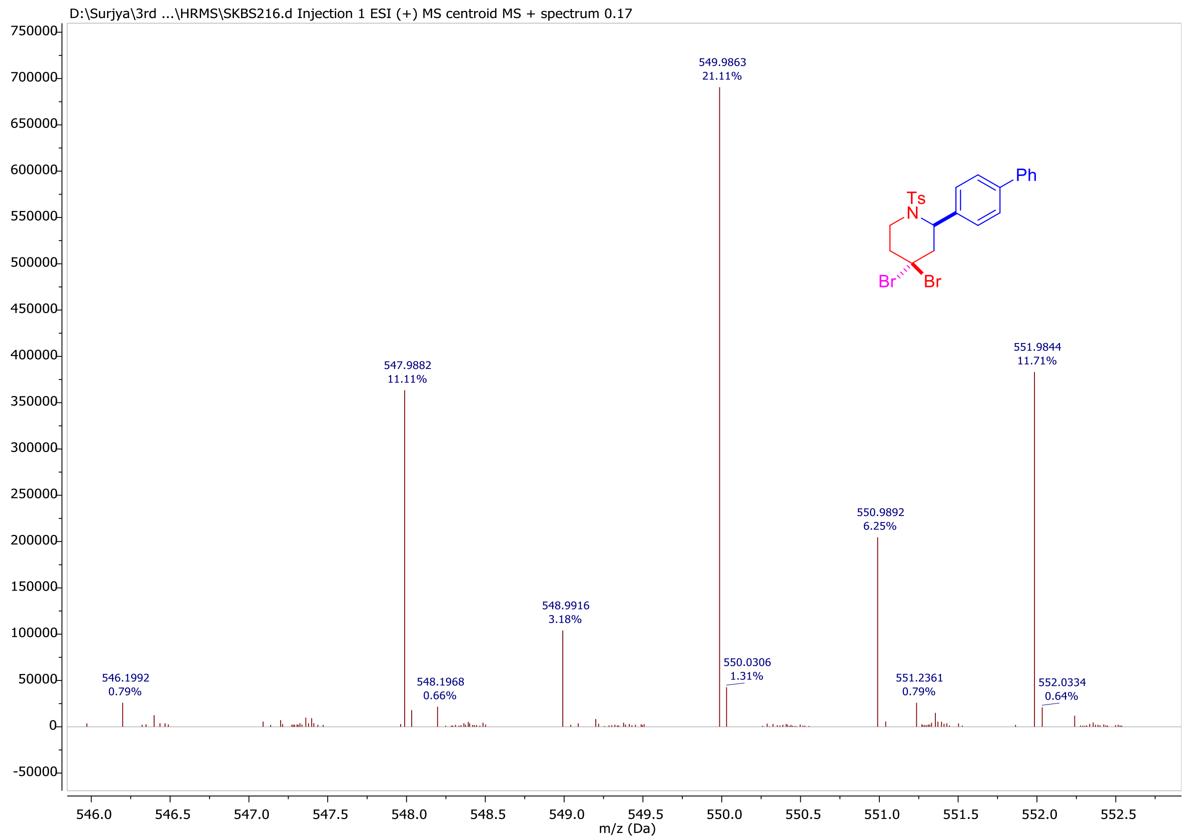

HRMS spectrum of **3aj**

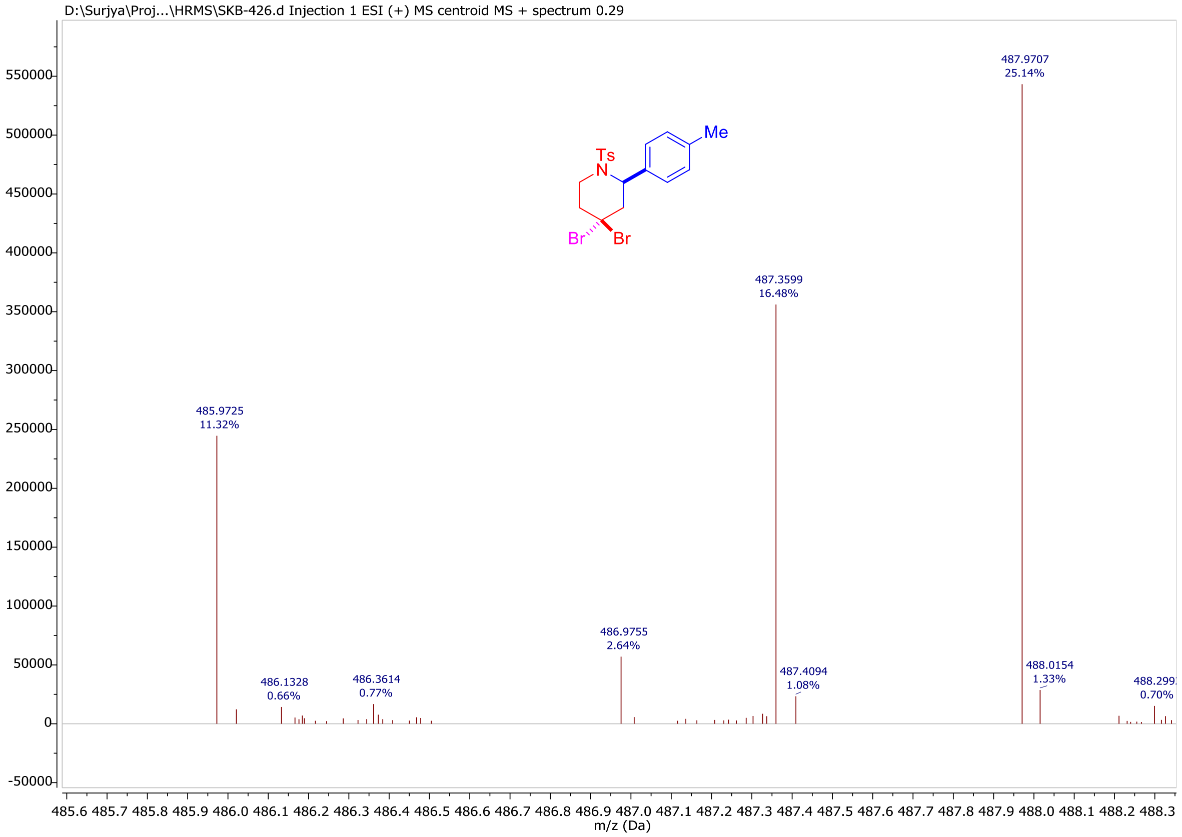

HRMS spectrum of **3al**

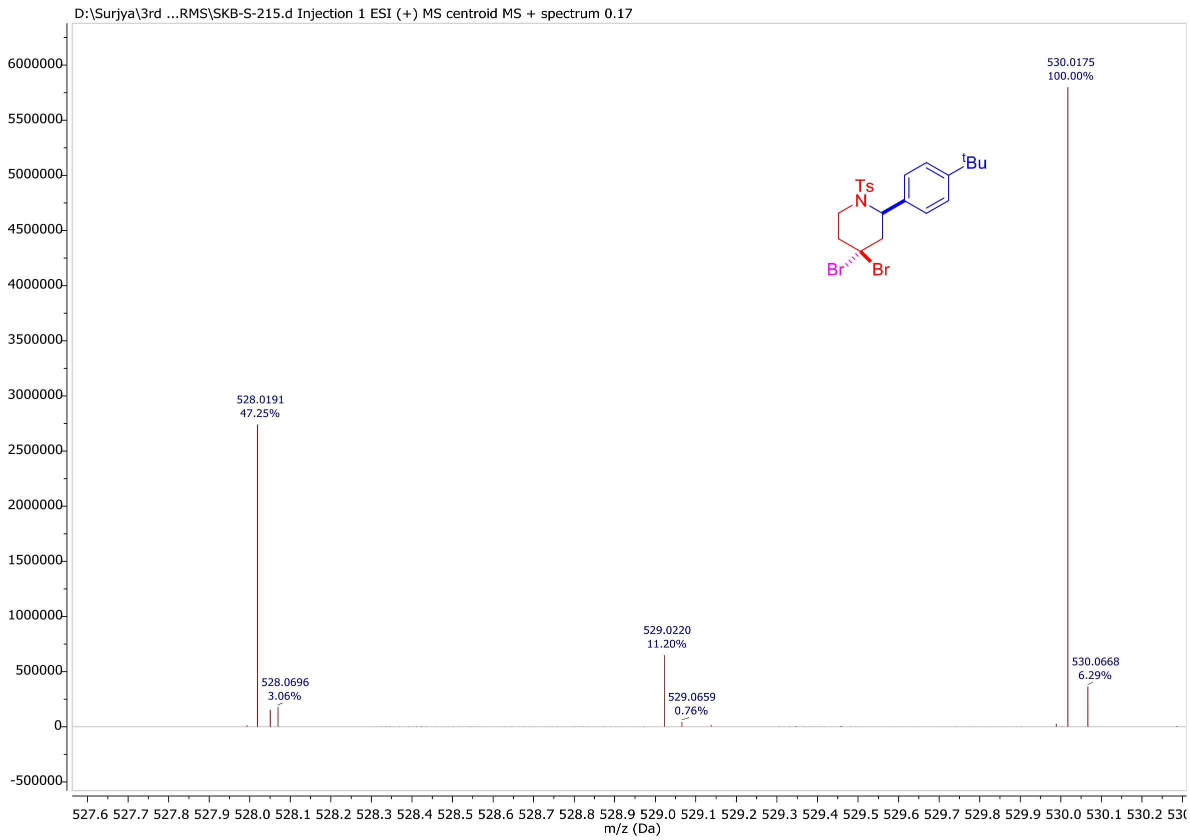

HRMS spectrum of **3am**

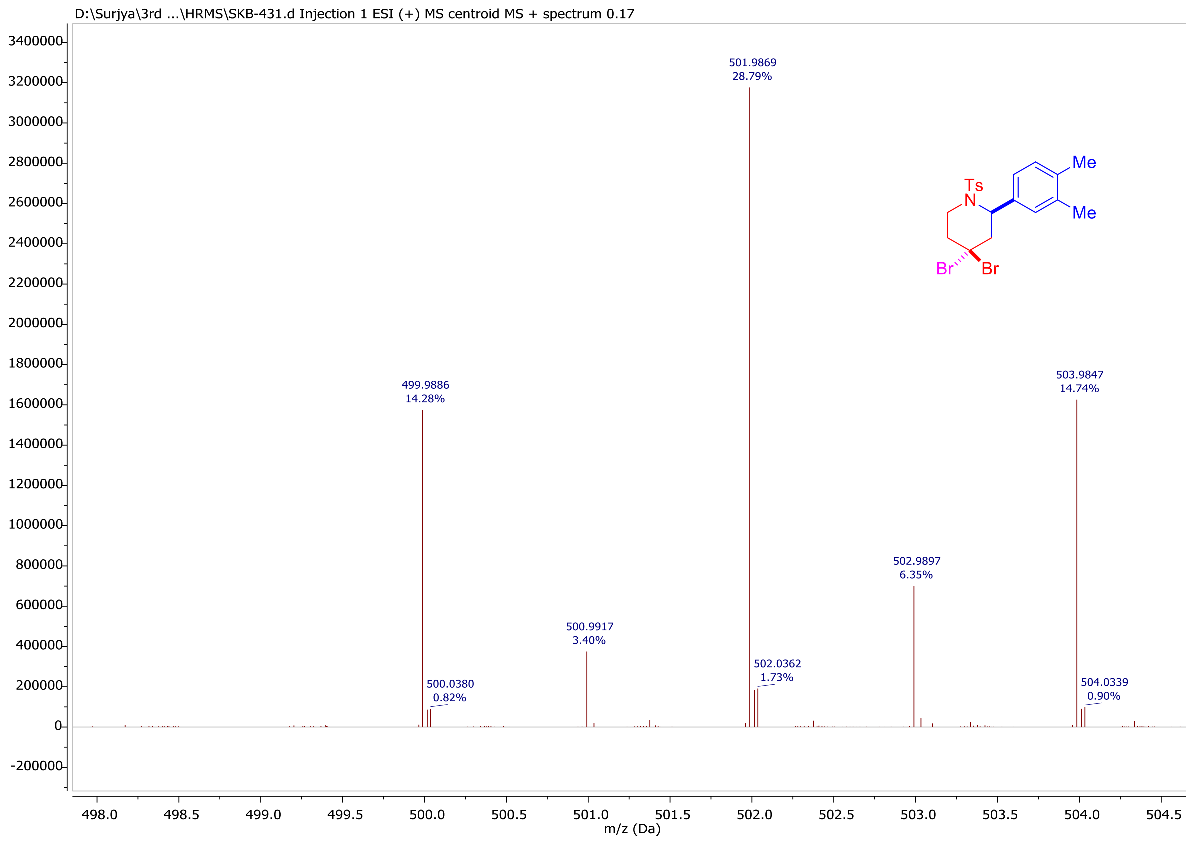

HRMS spectrum of **3an**

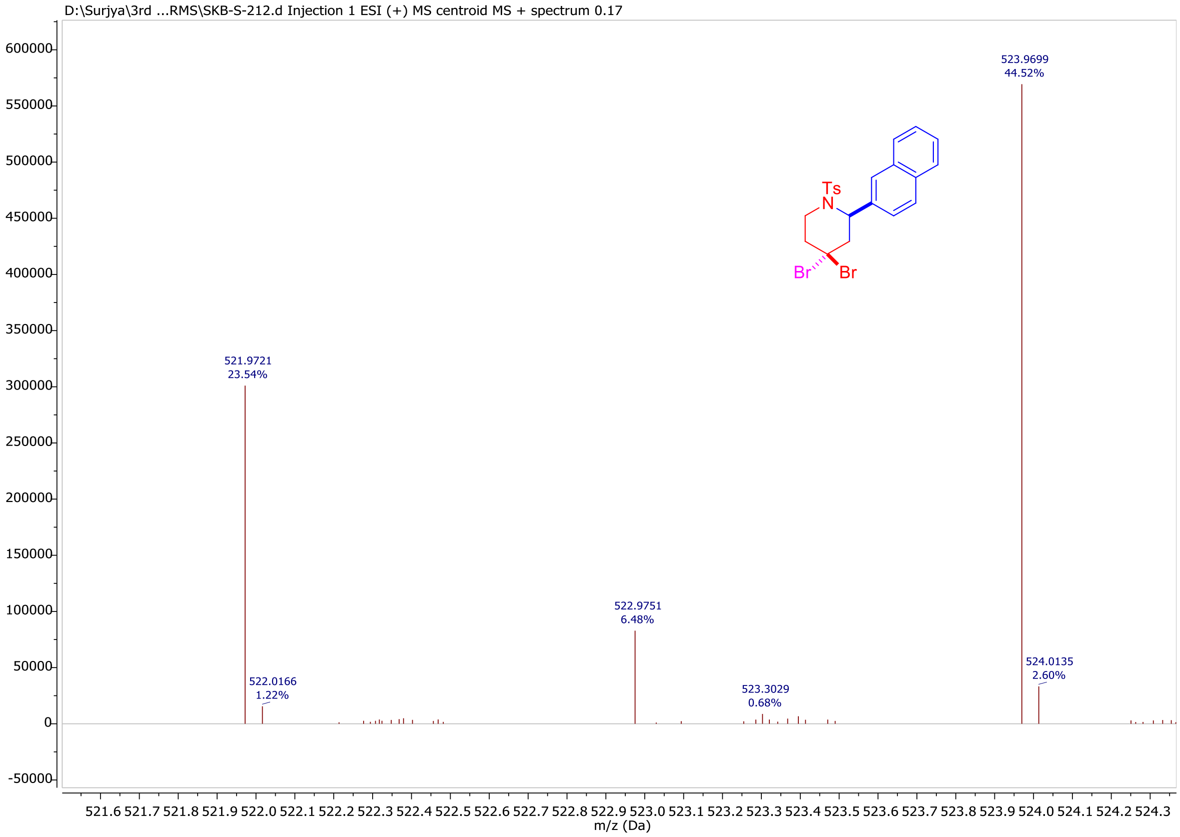

HRMS spectrum of **3ao**

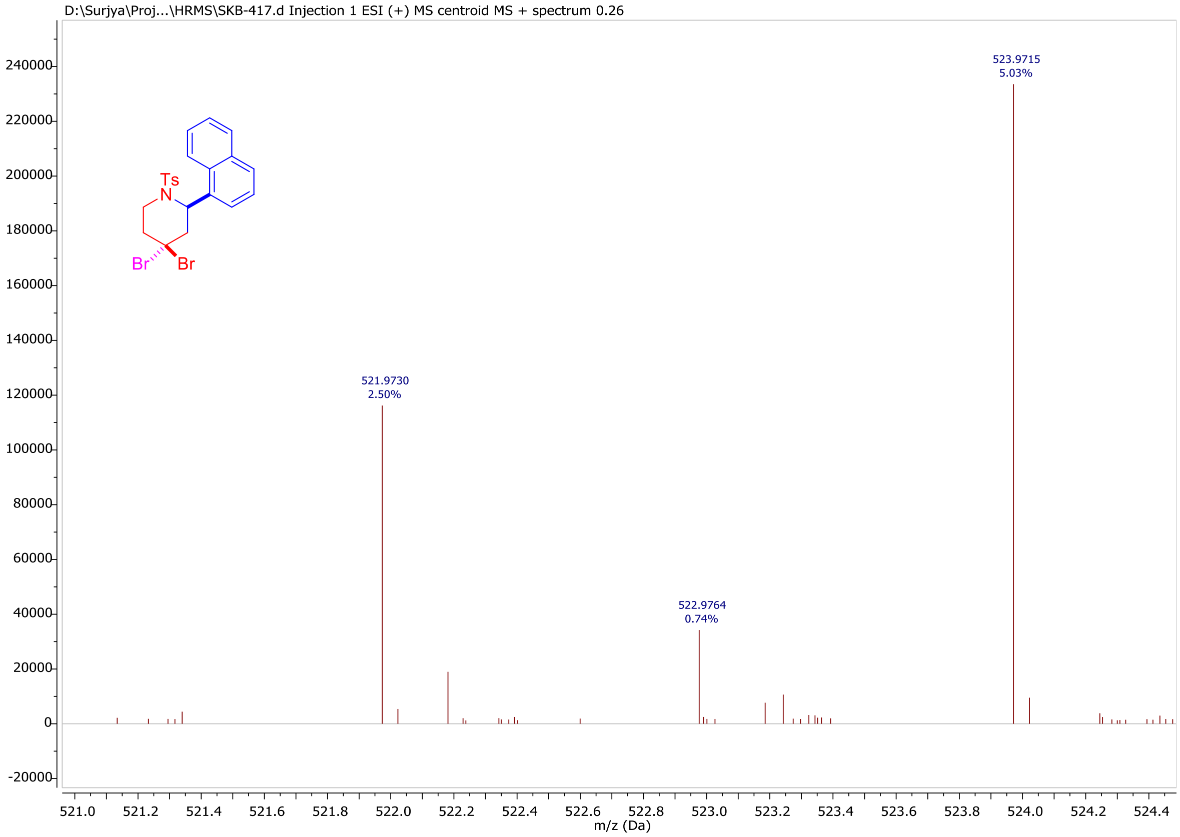

HRMS spectrum of **3ap**

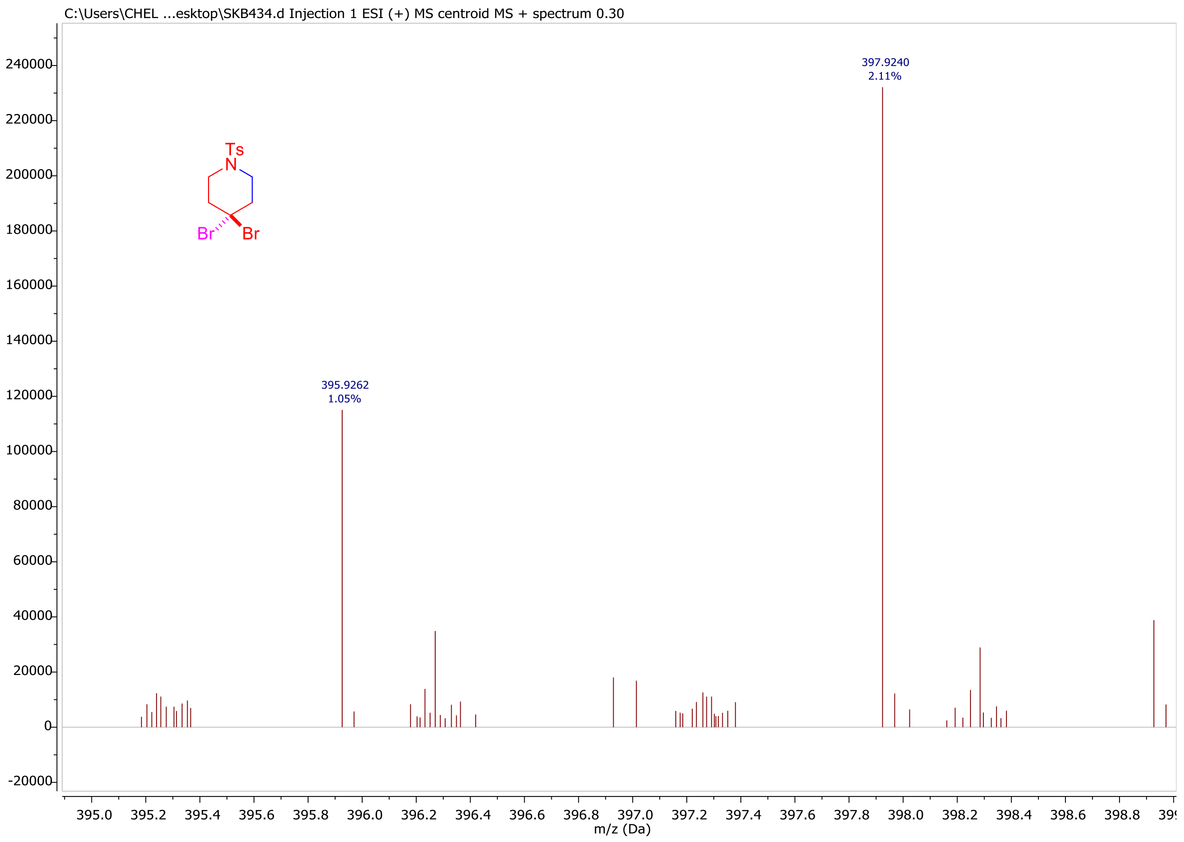

HRMS spectrum of **3aq**

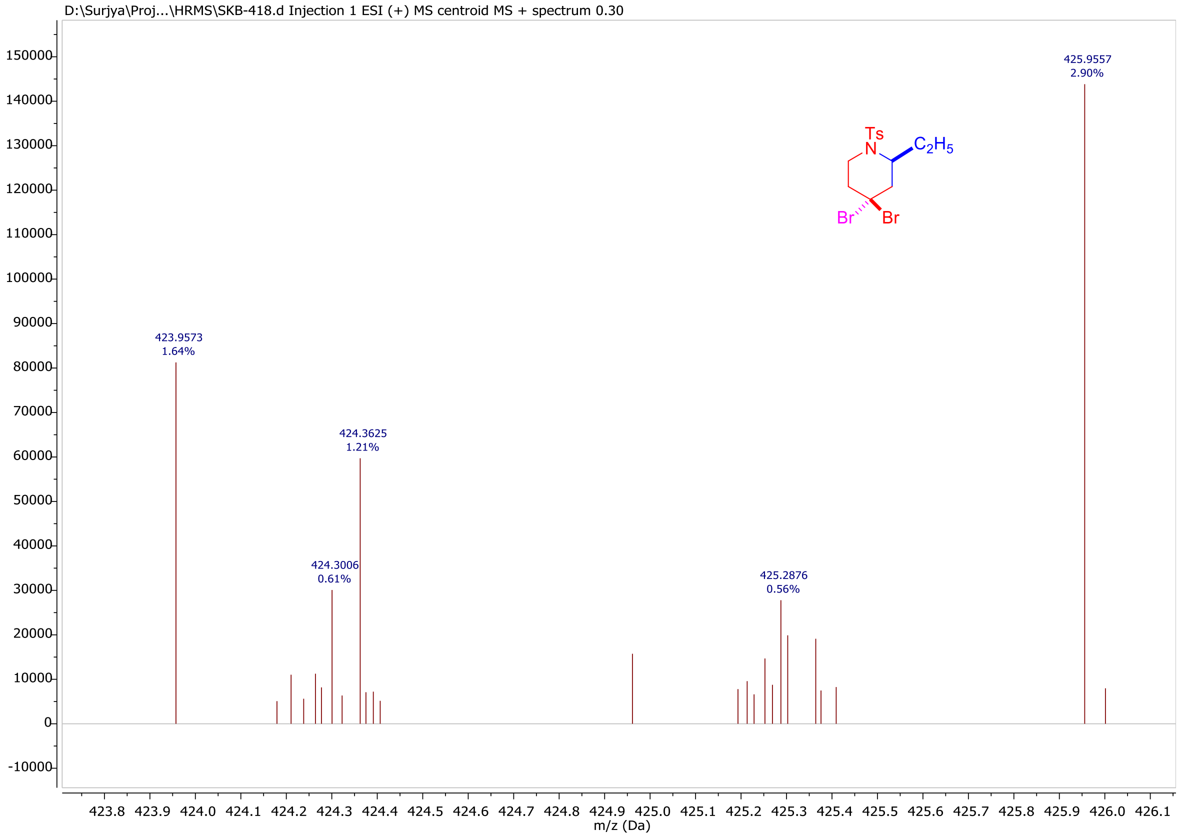

HRMS spectrum of **3ar**

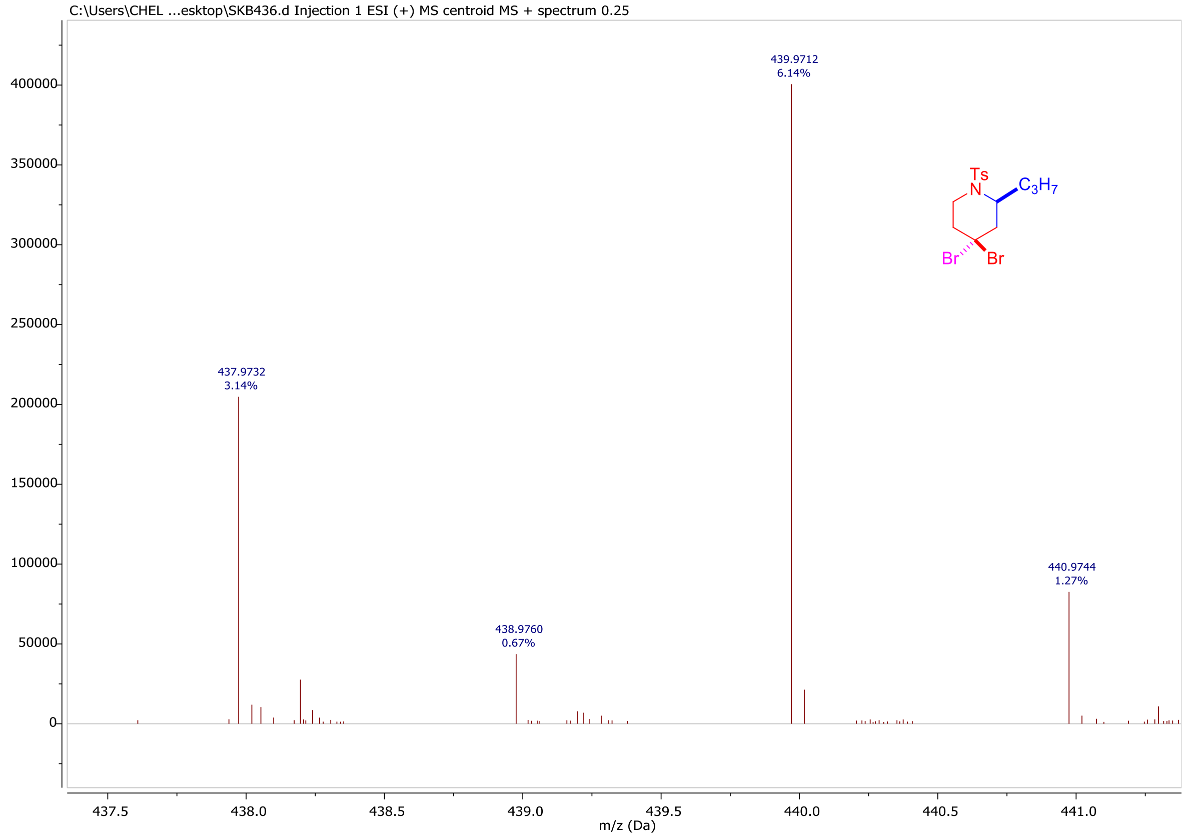

HRMS spectrum of **3as**

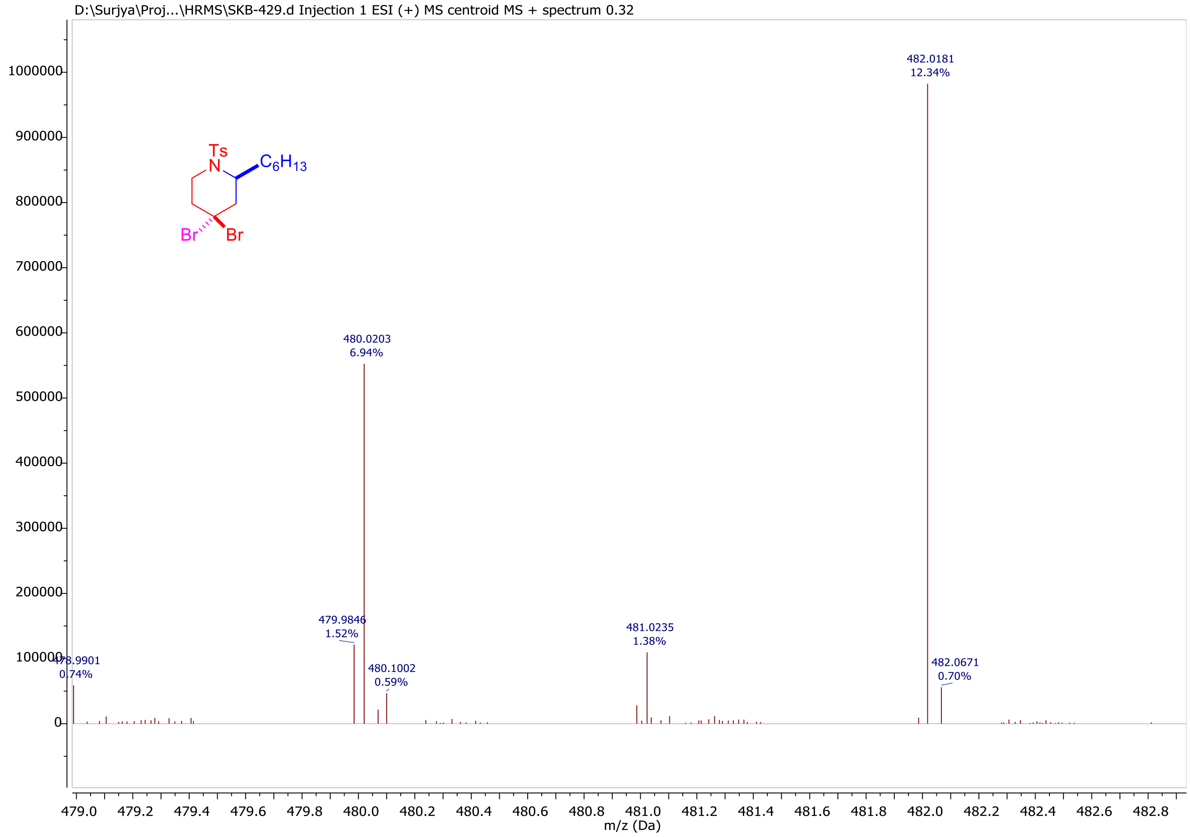

HRMS spectrum of **3at**

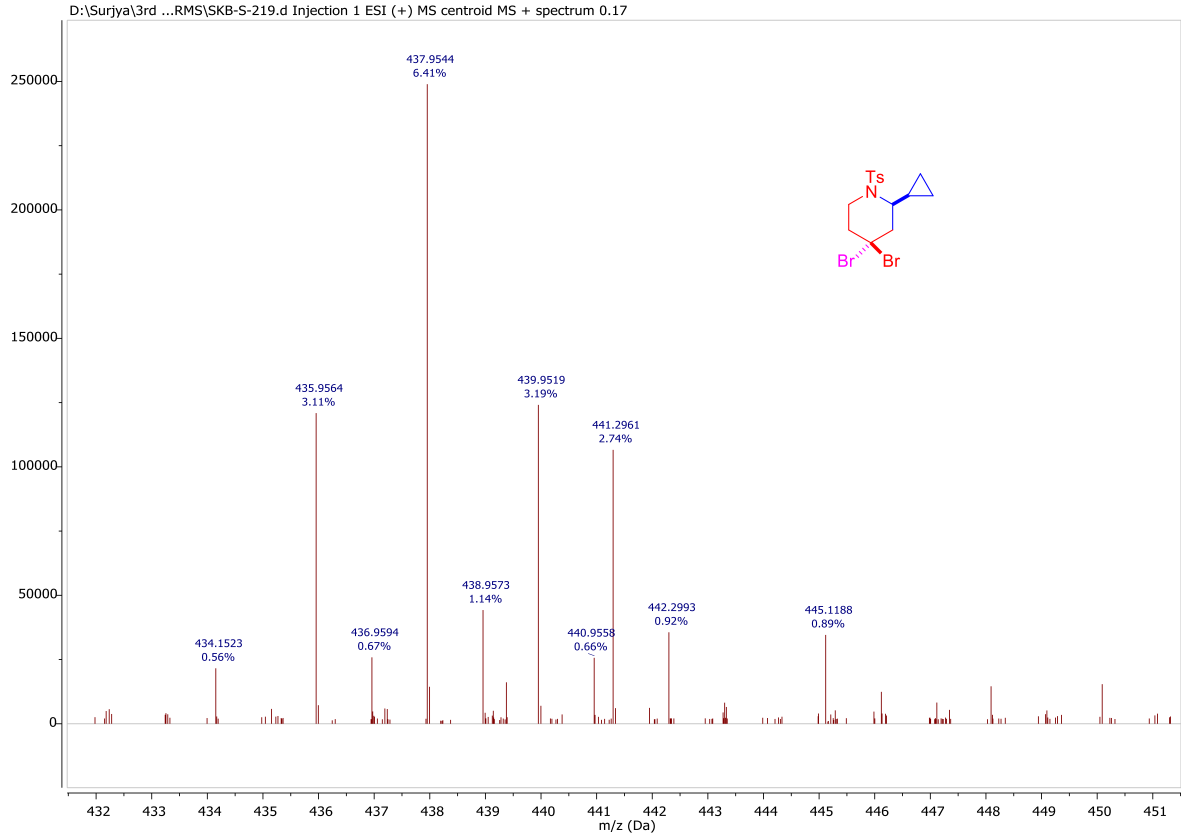

HRMS spectrum of **3au**

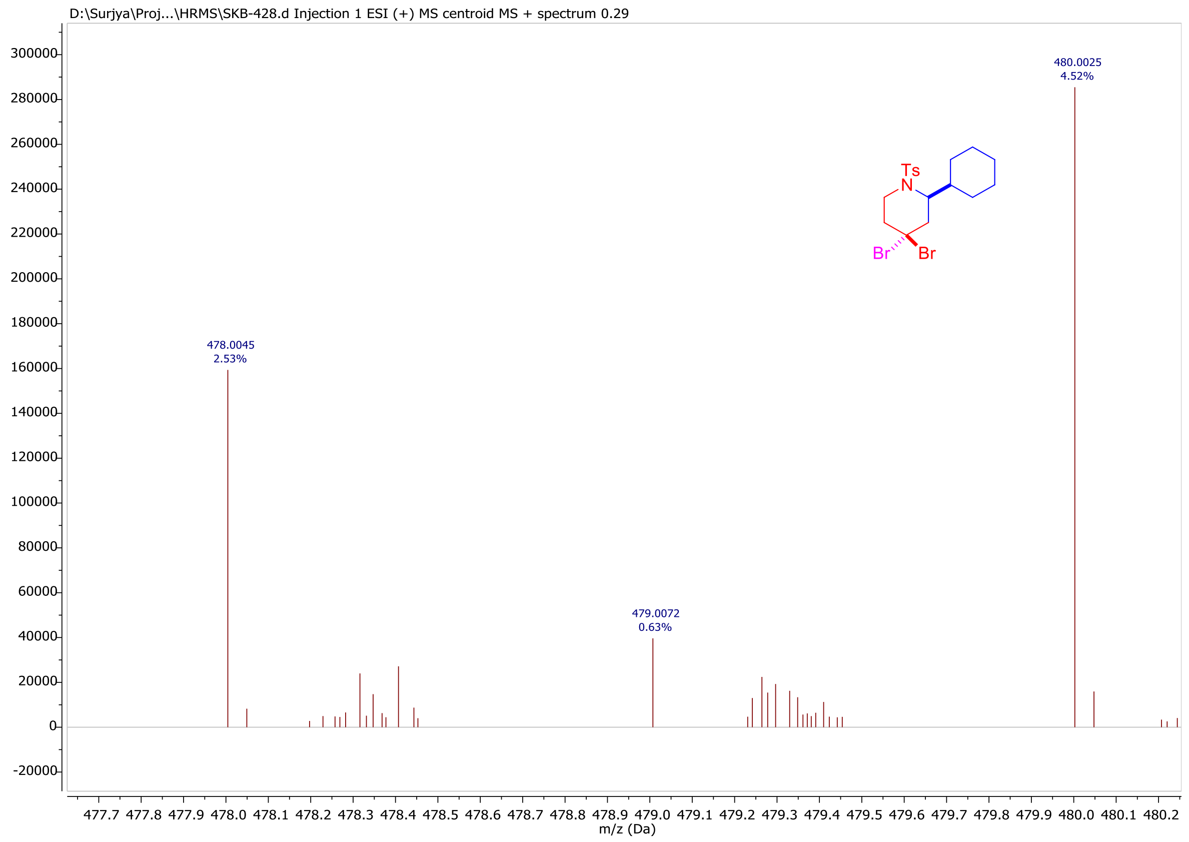

HRMS spectrum of **3dj**

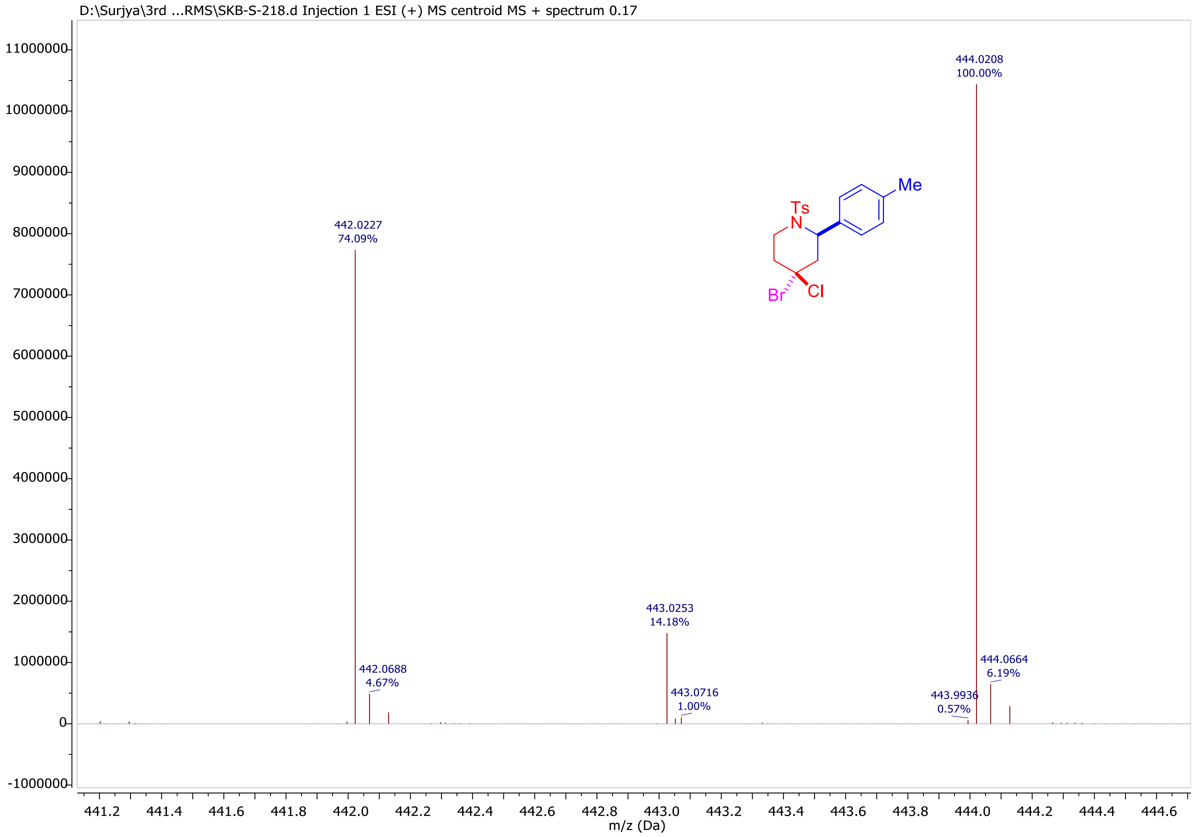

HRMS spectrum of **3ed**

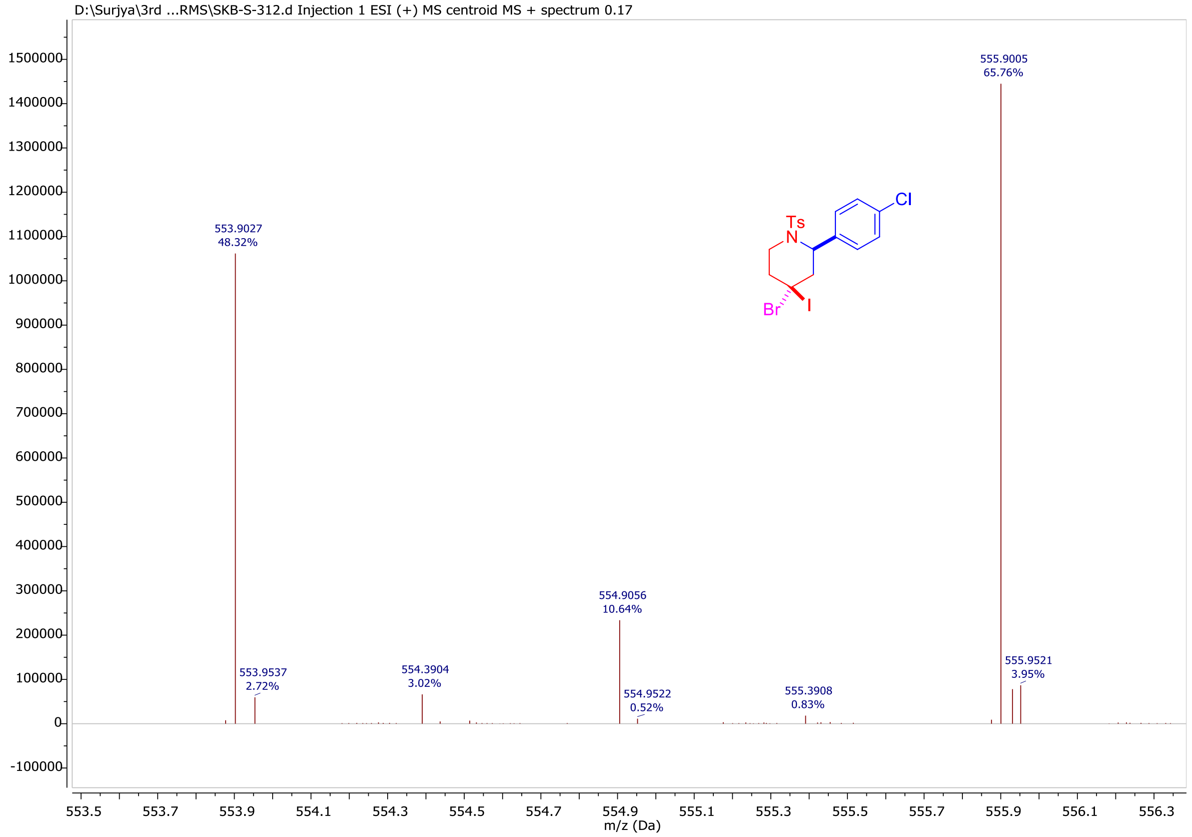

HRMS spectrum of **4aa**

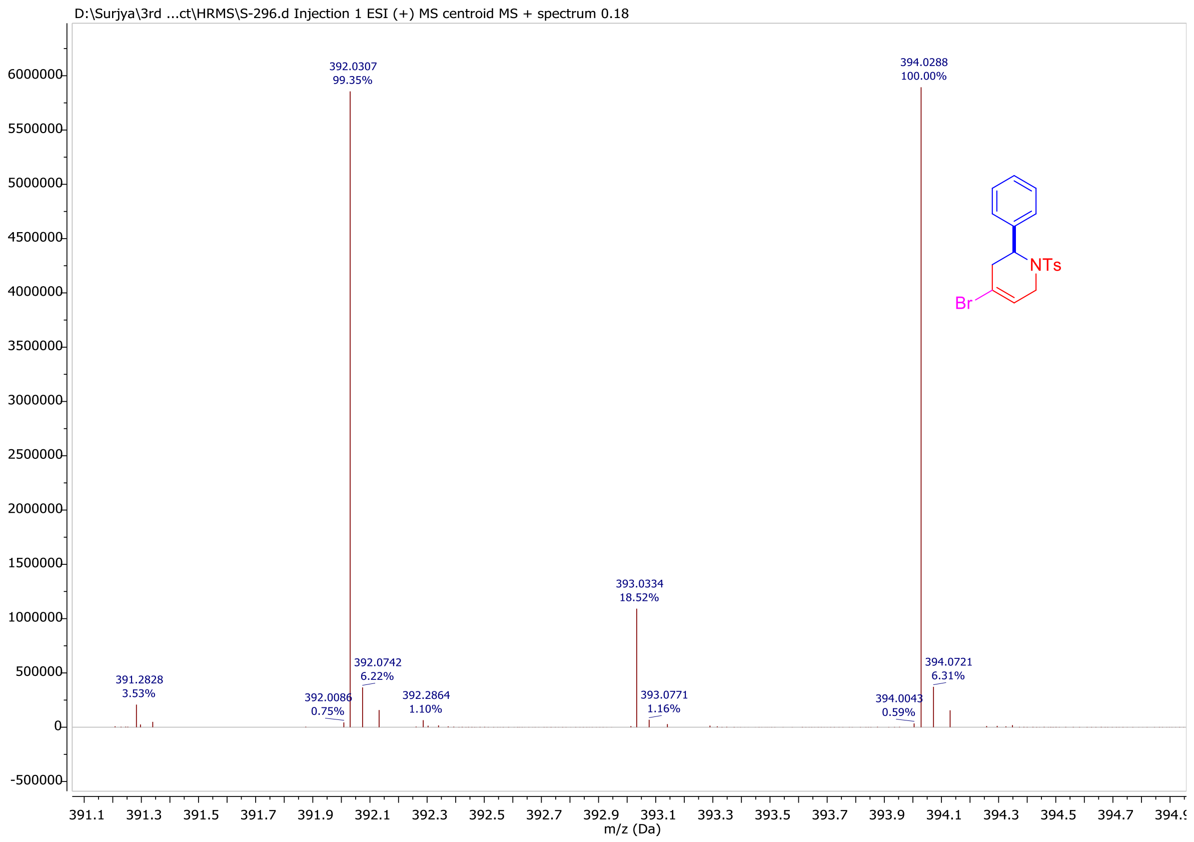

HRMS spectrum of **4ac**

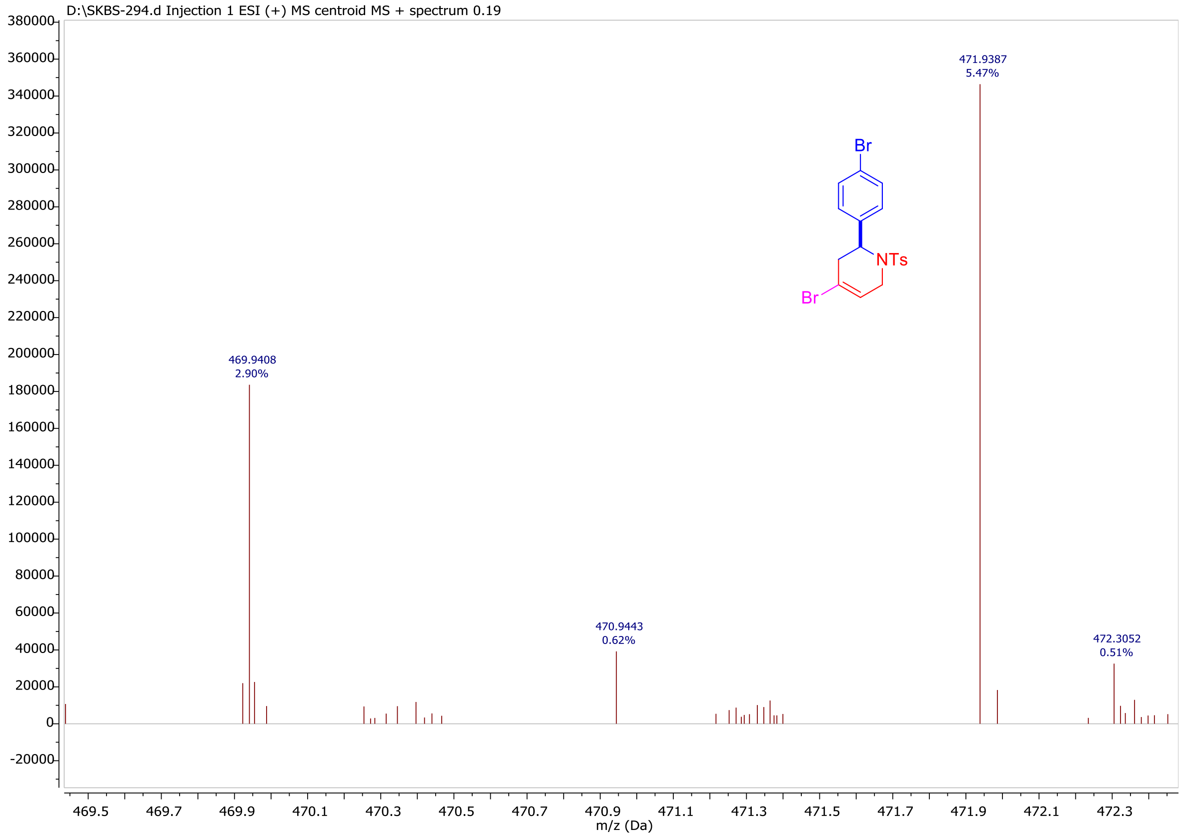

HRMS spectrum of **4ad**

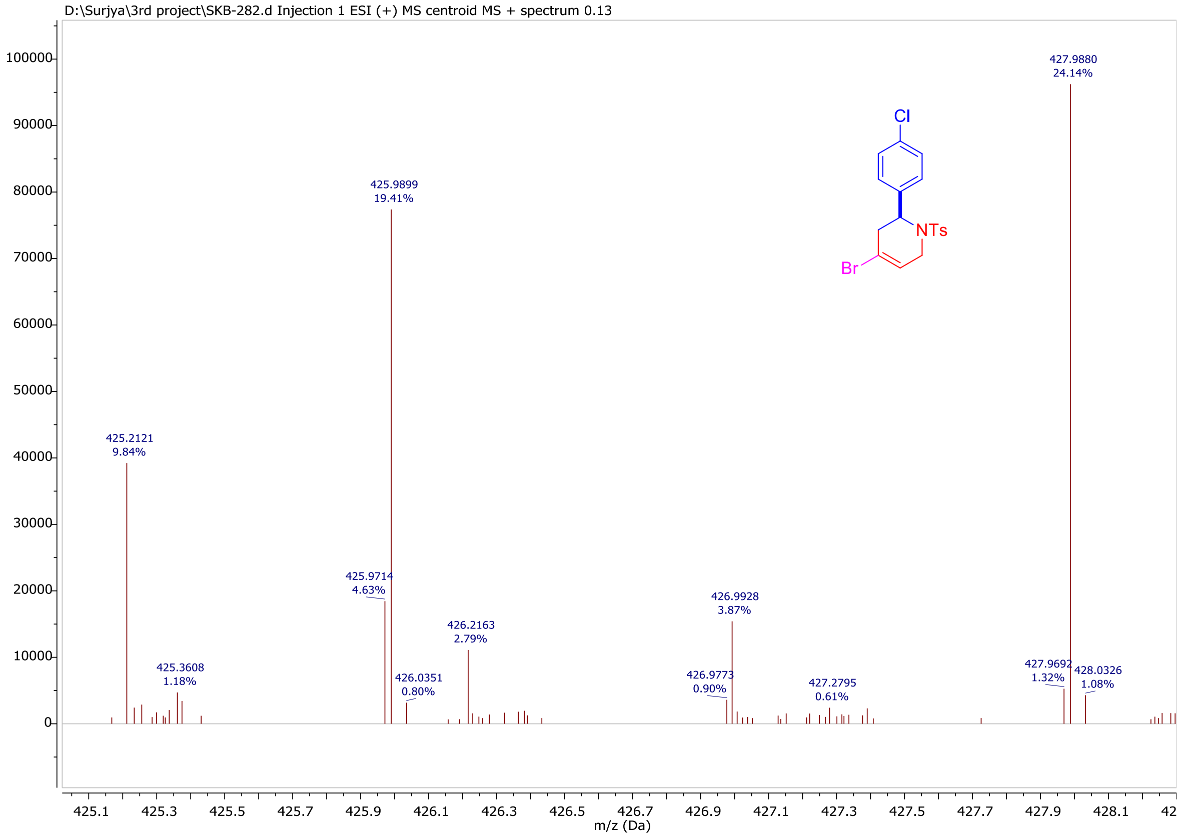

HRMS spectrum of **4ai**

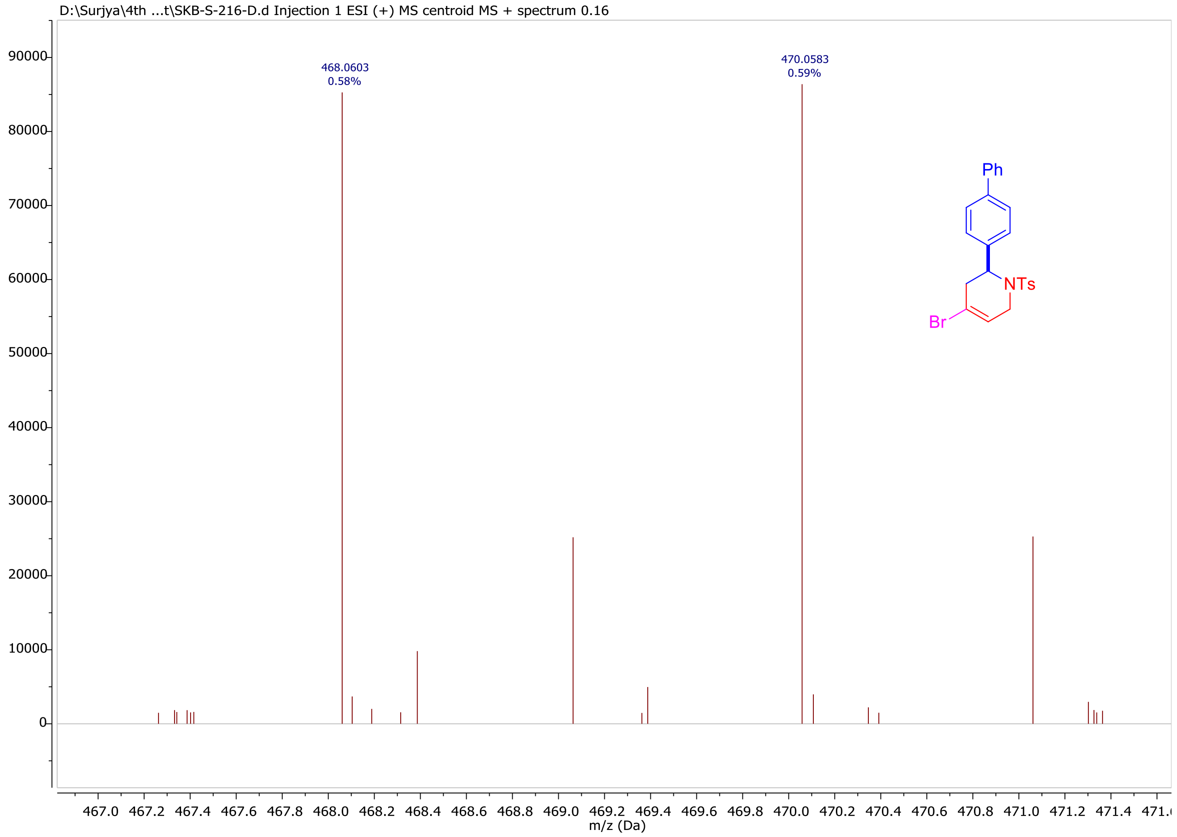

HRMS spectrum of **4aj**

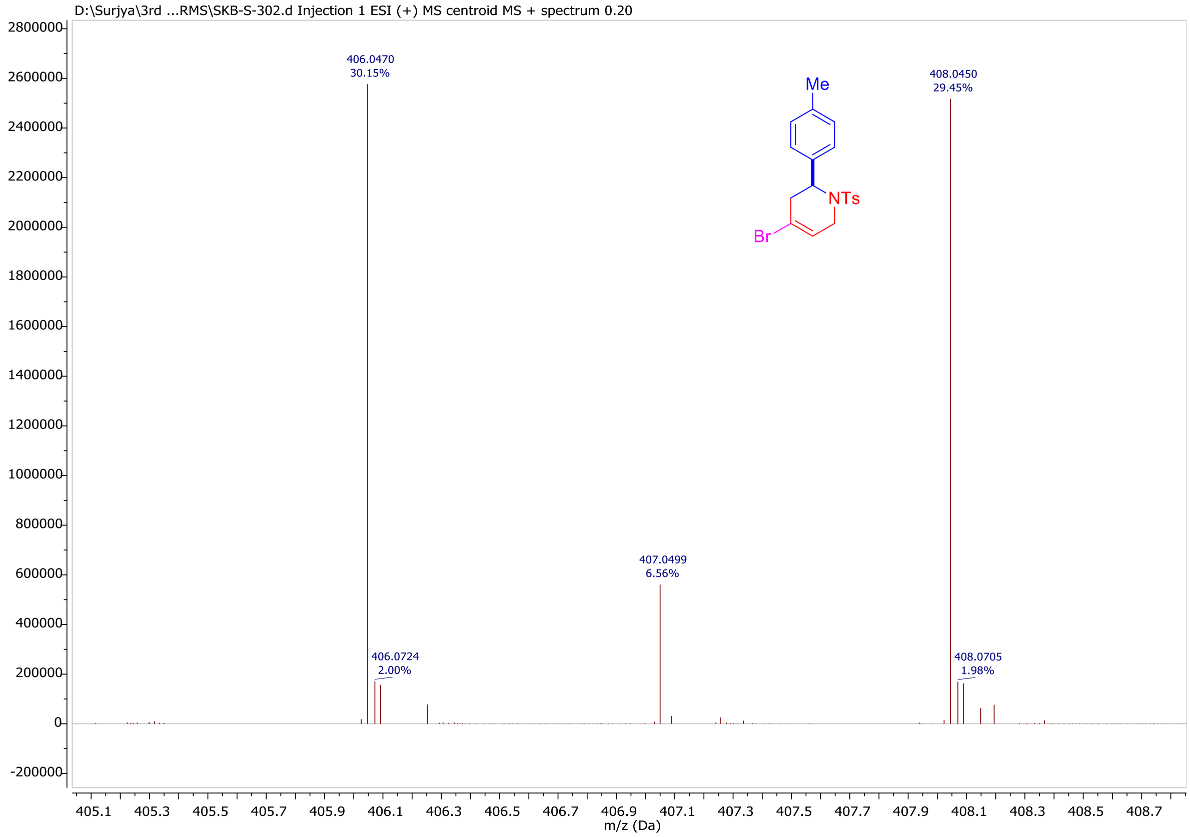

HRMS spectrum of **4an**

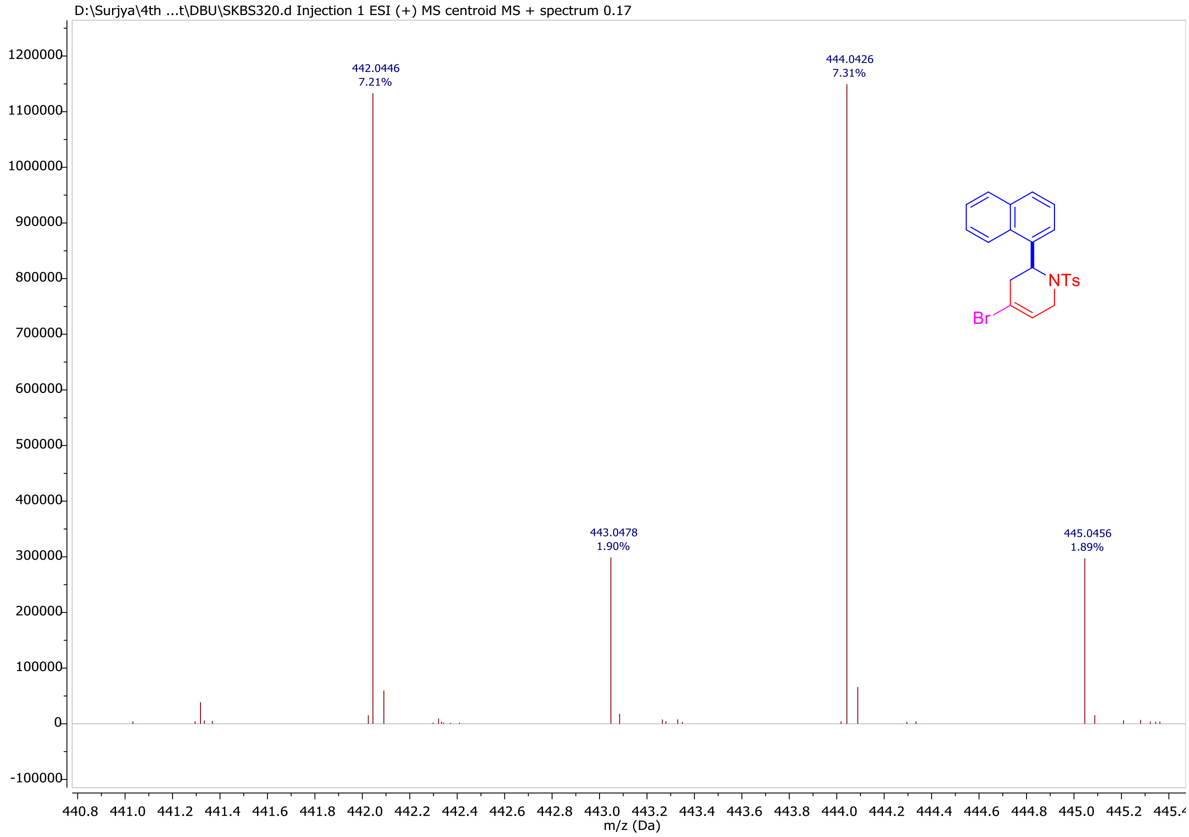

HRMS spectrum of **4aq**

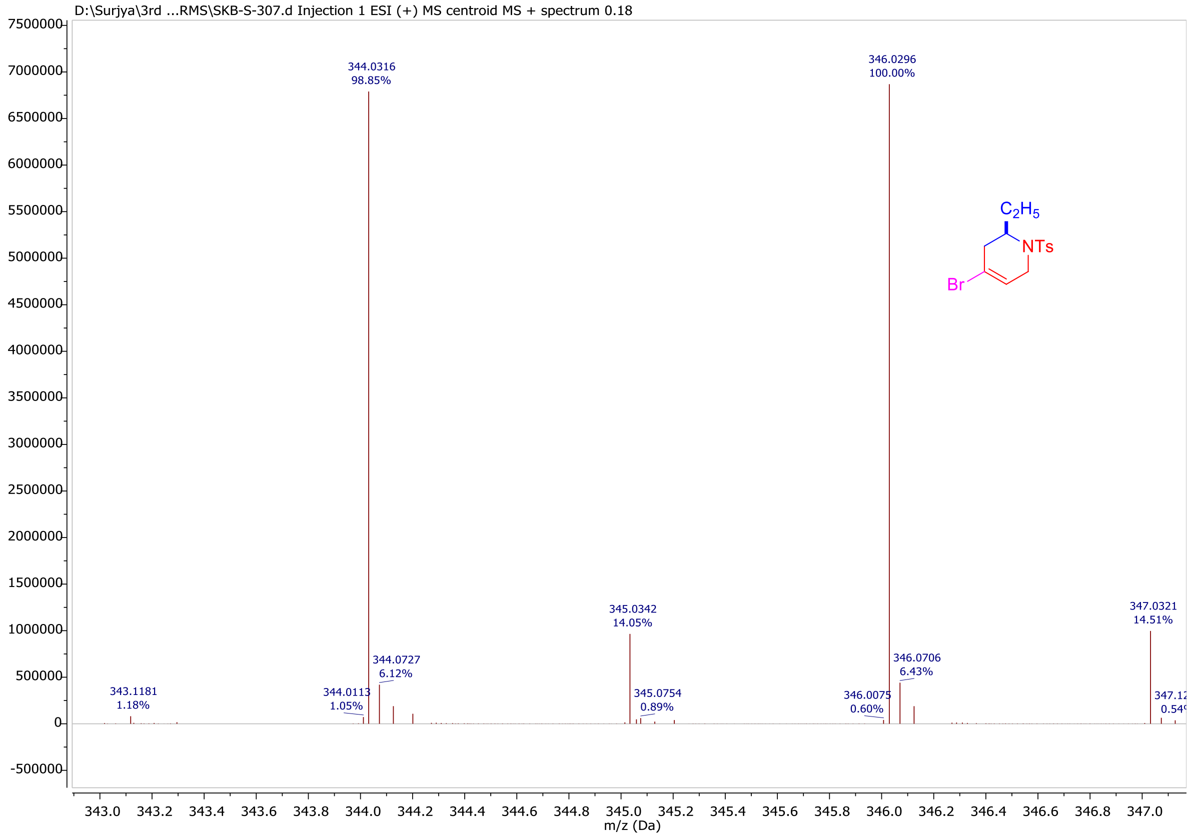

HRMS spectrum of **4db**

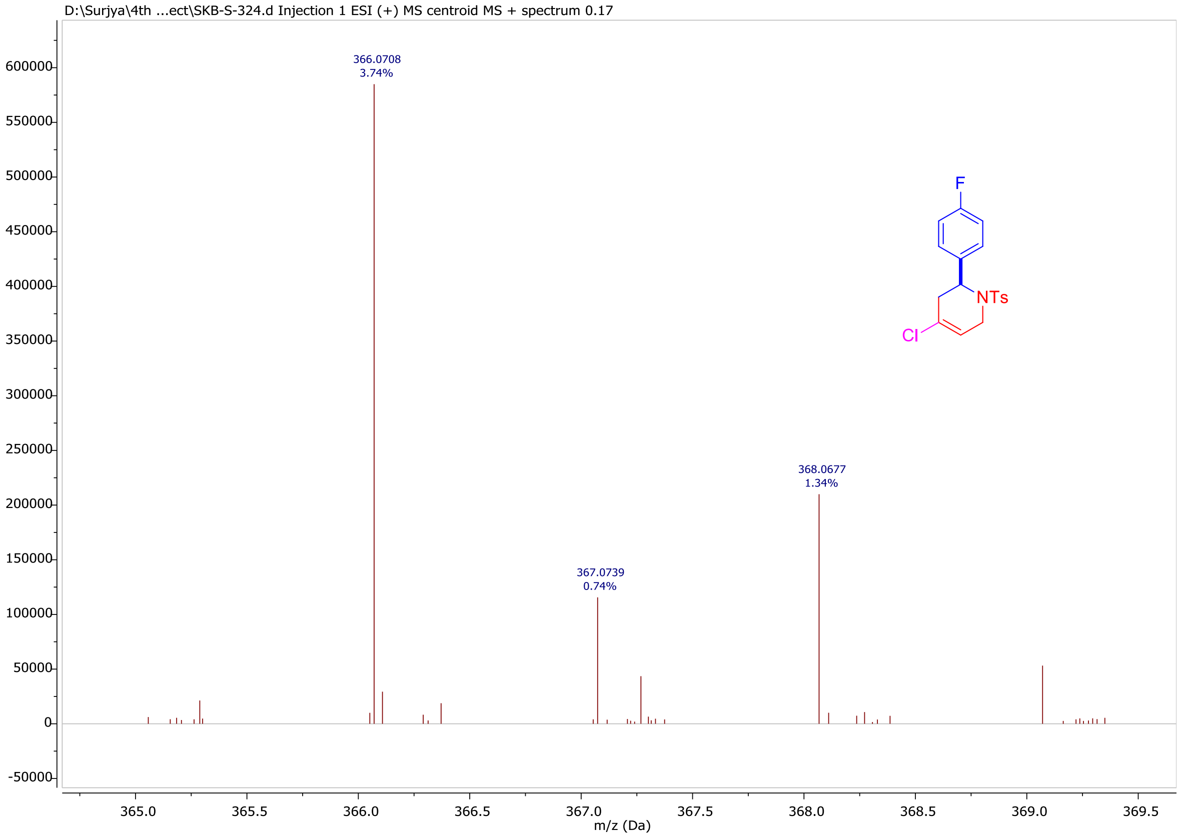

HRMS spectrum of **4de**

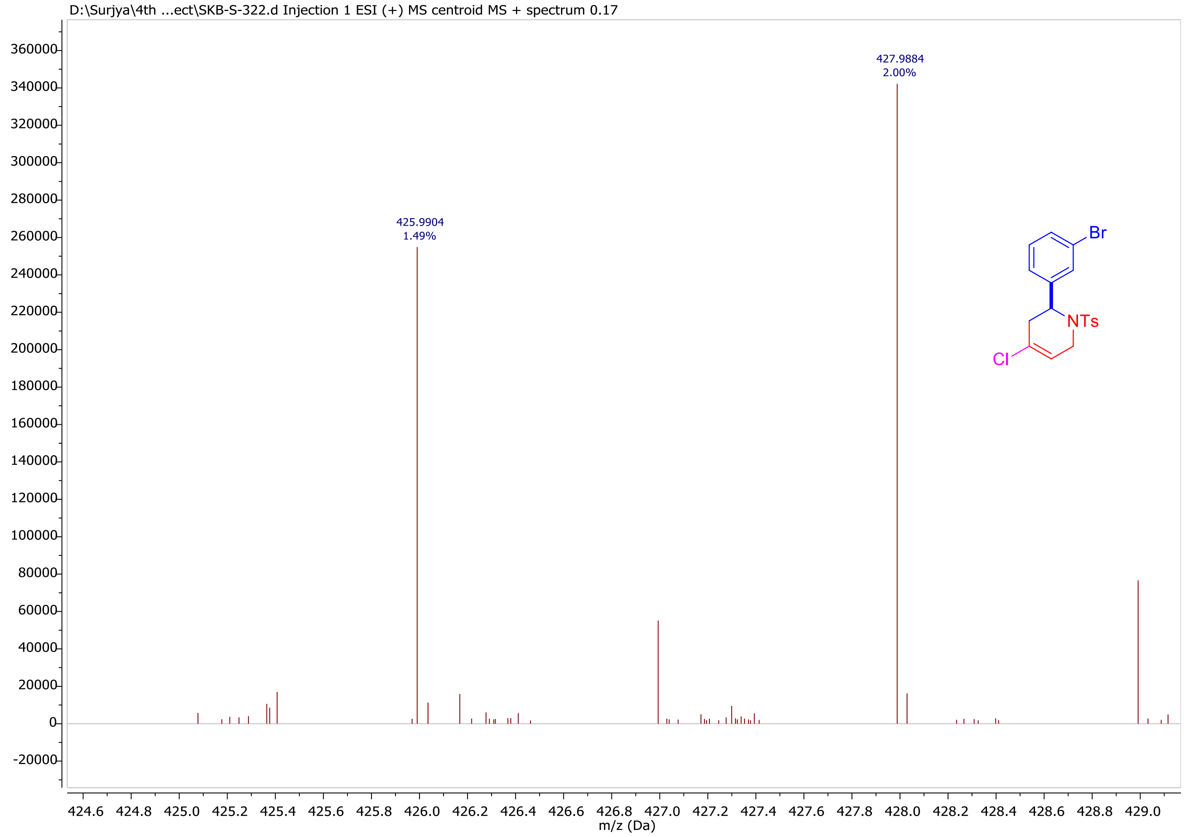

HRMS spectrum of **4dj**

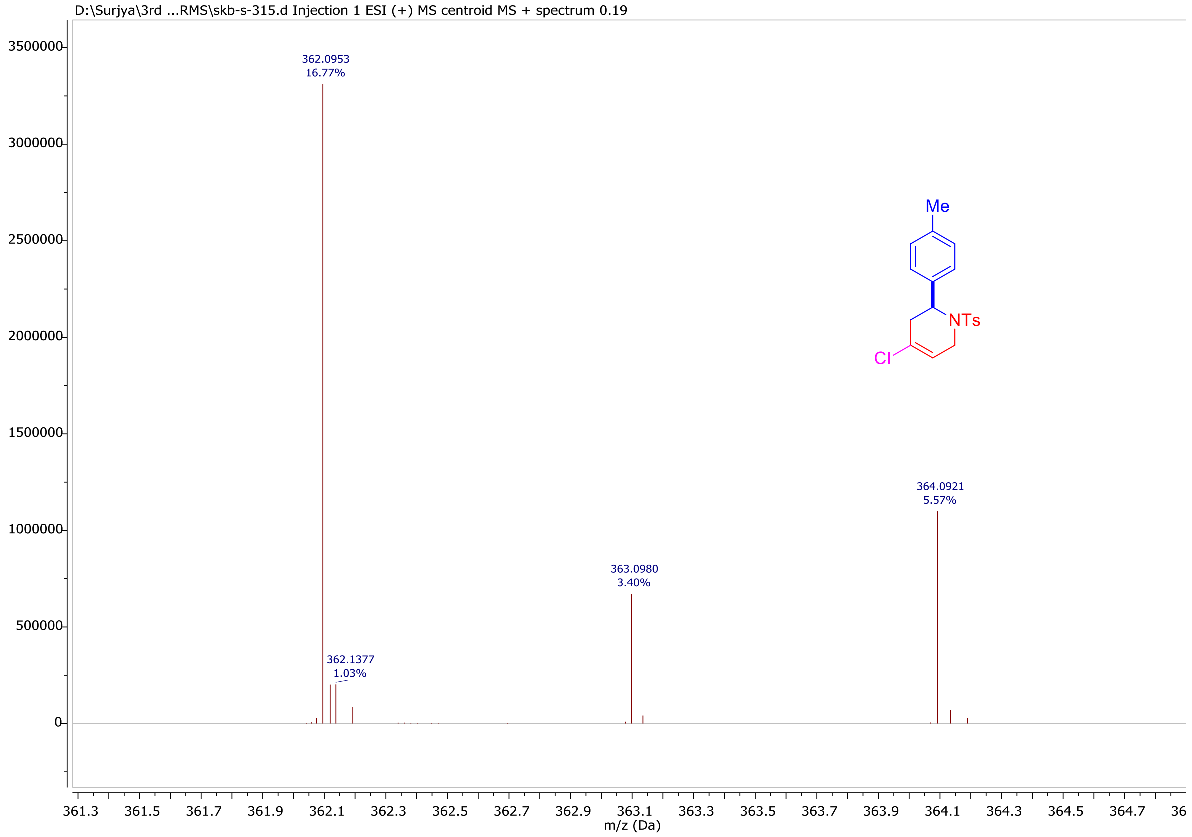

HRMS spectrum of **5a**

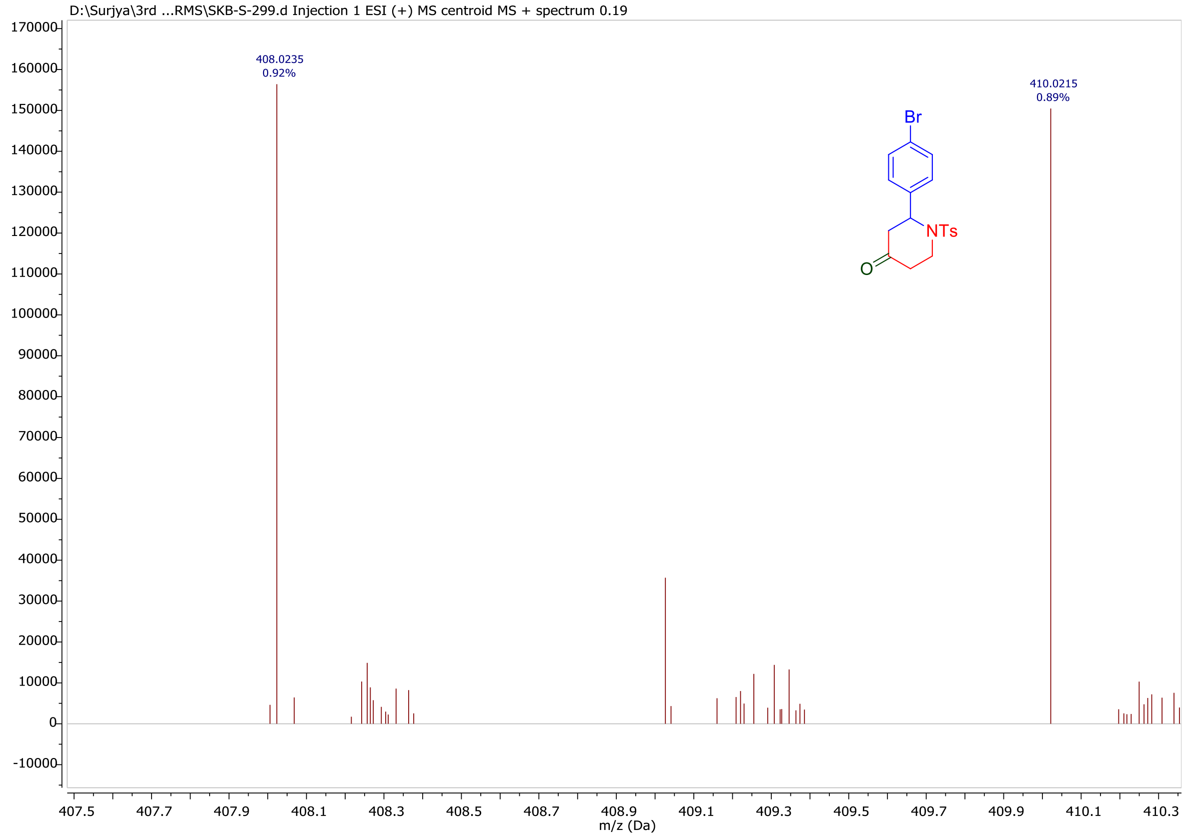

HRMS spectrum of **6a**

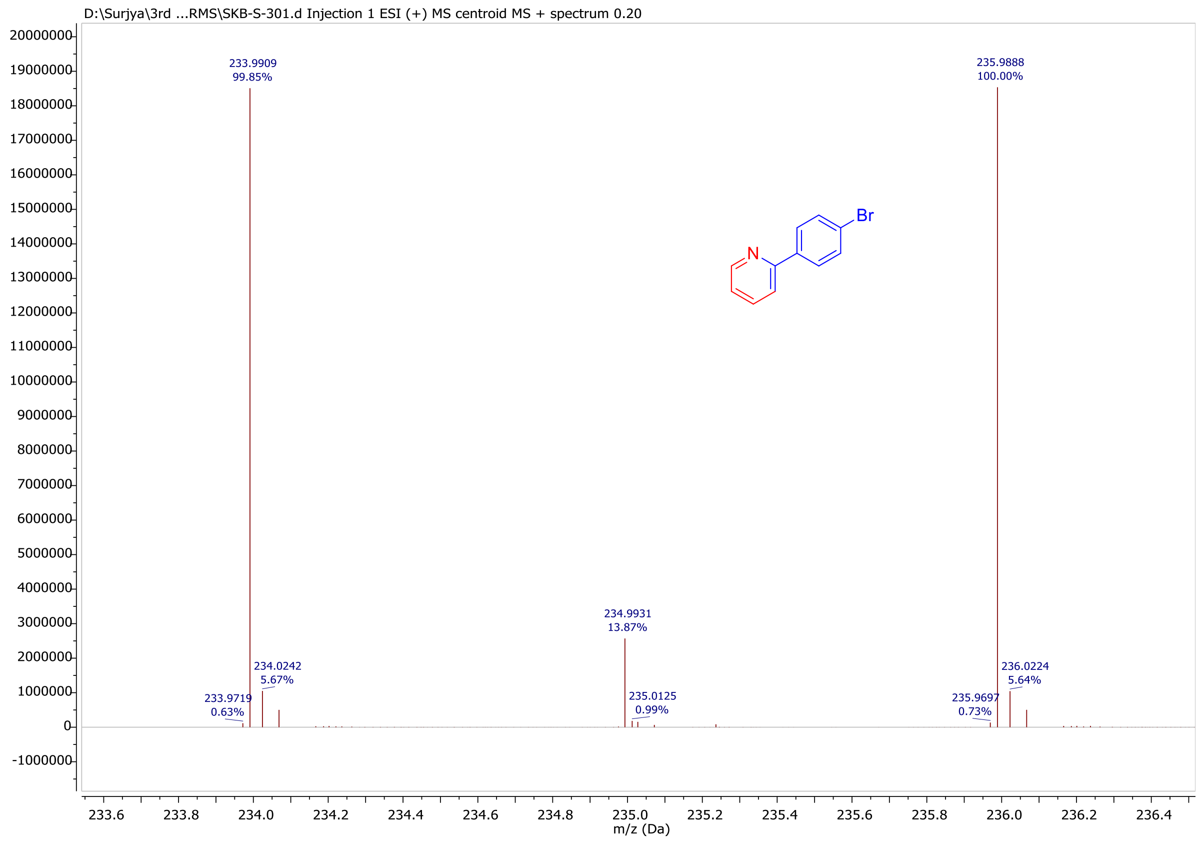

HRMS spectrum of **7**

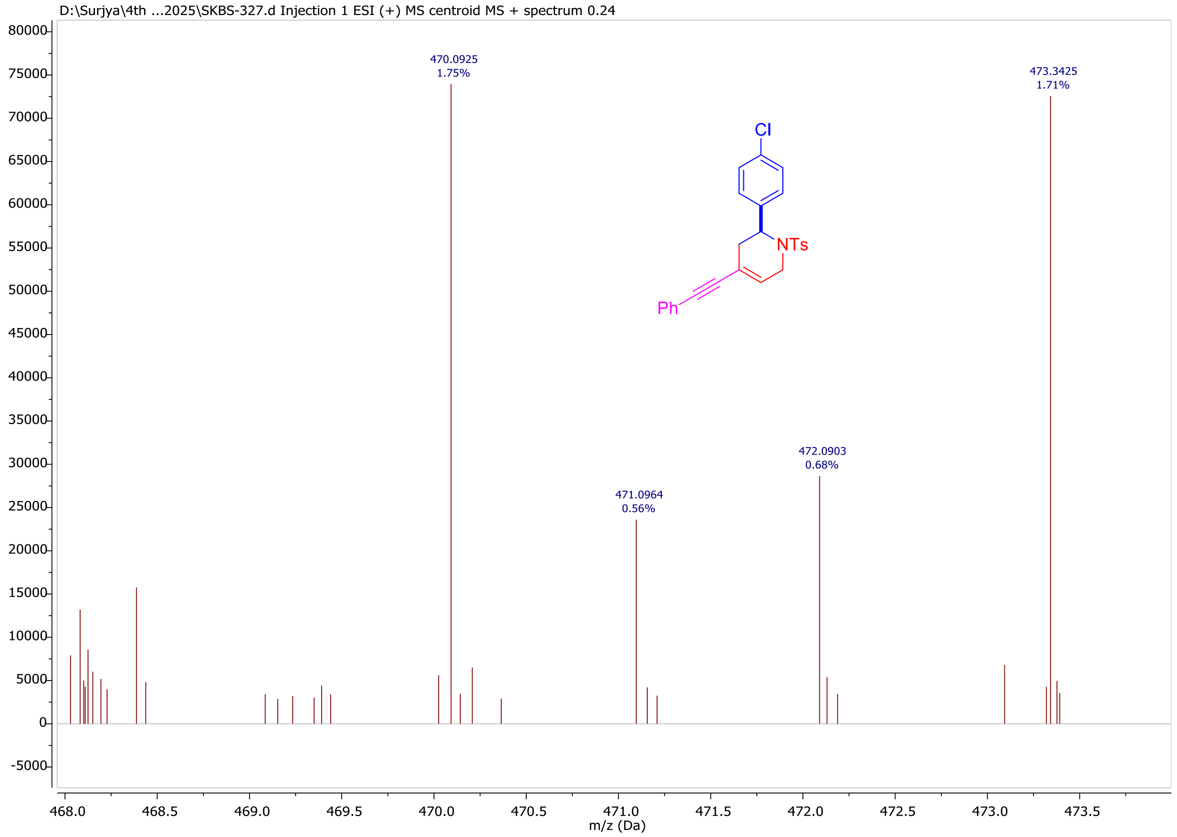

Supplement: RA-015-D5RA03630E-s002 [file RA-015-D5RA03630E-s002.pdf]
